# Ru(II)Porphyrinate-based molecular nanoreactor for carbene insertion reactions and quantitative formation of rotaxanes by active-metal-template syntheses

**DOI:** 10.1038/s41467-020-20046-x

**Published:** 2020-12-11

**Authors:** Liniquer A. Fontana, Marlon P. Almeida, Arthur F. P. Alcântara, Vitor H. Rigolin, Marcos A. Ribeiro, Wdeson P. Barros, Jackson D. Megiatto

**Affiliations:** 1 Institute of Chemistry, University of Campinas (UNICAMP), POBox 6154, 13083-970 Campinas, Brazil; 2 Instituto Federal do Sertão Pernambucano, Estrada do Tamboril, 56200-000 Ouricuri, Brazil; 3 Departamento de Química, Universidade Federal do Espírito Santo, Av. Fernando Ferrari, 514, 29075-910 Vitória, Brazil

**Keywords:** Chemistry, Interlocked molecules

## Abstract

Selectivity in N–H and S–H carbene insertion reactions promoted by Ru(II)porphyrinates currently requires slow addition of the diazo precursor and large excess of the primary amine and thiol substrates in the reaction medium. Such conditions are necessary to avoid the undesirable carbene coupling and/or multiple carbene insertions. Here, the authors demonstrate that the synergy between the steric shielding provided by a Ru(II)porphyrinate-based macrocycle with a relatively small central cavity and the kinetic stabilization of otherwise labile coordinative bonds, warranted by formation of the mechanical bond, enables single carbene insertions to occur with quantitative efficiency and perfect selectivity even in the presence of a large excess of the diazo precursor and stoichiometric amounts of the primary amine and thiol substrates. As the Ru(II)porphyrinate-based macrocycle bears a confining nanospace and alters the product distribution of the carbene insertion reactions when compared to that of its acyclic version, the former therefore functions as a nanoreactor.

## Abstract (web summary)

Selectivity in carbene insertion reactions promoted by Ru(II)porphyrinates is achieved only upon careful control of substrate stoichiometry. Here, the authors demonstrate that endotopic catalysis and formation of mechanical bonds enables carbene insertions to occur selectively and in quantitative yield regardless of substrate stoichiometry.

## Introduction

The rich coordination chemistry of porphyrinates^1–18^ is useful in the design of macrocyclic receptors, in which the porphyrinate subunits are positioned on the top of molecular capsules to create hollow structures with well-defined cavities^19–28^. In such molecular architectures, one of the axial positions of the metallic center is shielded from the environment, while the other has unencumbered reactivity. This asymmetry between the two axial coordination sites of the porphyrinate subunit can be explored to introduce a steric bias in the binding modes of the receptor^18–22^. For example, the porphyrinate’s external axial position can be blocked by selective coordination of bulky and inert monodentate ligands. With such configuration of the ligands around the metal center, the internal coordination site of the porphyrinate-based receptor can bind nonbulky substrates to efficiently and selectively promote their reactions inside the central cavity^23–32^. Such versatile strategy has been explored in the active-metal template synthesis of interlocked molecules^24–27^, asymmetric catalysis^28,29^ and biomimetics^30–32^. However, in those applications, the porphyrinate-based macrocycles has virtually the same reactivity as their acyclic analogues.

To expand the scope of porphyrinate-based molecular capsules, incorporation of nanoreactor concepts^33^ are highly desirable. Nanoreactors are designed to create nanoscale chemical environments partitioned from the bulk in order to change the reactivity of molecules upon binding inside the cavity, therefore altering their behavior in chemical transformations. Congruently, nanoreactors can dramatically change the outcome of chemical reactions ^33^.

The carbene transfer/insertion reactions promoted by Ru(II)porphyrinates from diazo derivatives are versatile synthetic methods as they catalyze a wide range of chemical transformations such as cyclopropanation, 1,3-dipolar cycloadditions, X–H (X = C, N, O, and S) insertions and olefination of aldehydes in the presence of phosphines^34–42^. However, the methodologies based on carbene transfer/insertion processes promoted by Ru(II)porphyrinates suffer from a serious drawback, which is the concurrent dimerization side-reaction of the carbene intermediates. To avoid the undesirable dimerization process, the methods described in the literature require slow addition of the diazo derivative into the reaction medium in order to keep the carbene concentration at low levels^34–42^. Such strategy has had limited success^42^, often requiring long addition times of the diazo derivative. Among the X–H bond carbene insertion reactions, the N–H one is problematic^40–42^ for two reasons. Firstly, competition between the primary amine and the diazo groups on the substrates for the Ru(II)porphyrinate axial coordination sites slows down or even shuts down the Ru(II)porphyrinate capability to generate carbene intermediates. Secondly, selectivity to the secondary amine product is hard to achieve and requires careful control of the diazo concentration and reaction temperature as well as to use a large excess of the amine substrate (10× relative to the diazo derivative)^42^ to avoid double carbenoid insertions that lead to the tertiary amine analog.

Herein, it is reported the molecular design and coordinative properties of a Ru(II)porphyrinate-based molecular capsule that works as a nanoreactor. The molecular capsule quantitatively yields an asymmetrical [2]rotaxanes through the challenging single N–H carbenoid insertion by the active metal template technique using an equimolar mixture of substrates. No signs of dimerization side processes nor double insertions are observed in the rotaxane assembly reaction, even under severe experimental conditions for carbene transfer/insertion reactions^34–42^. Most surprisingly, the excess of substrates added to the reaction medium is completely recovered as unreacted materials after workup. That efficiency in formation of mechanical bonds is hard to achieve in the present case as the Ru(II)porphyrinate subunit on the macrocycle is still active after the rotaxane assembly process. Therefore, it was expected that intercomponent side reactions would plague our methodology as the inherent effective molarity effects of the mechanical bond should have favored such side reactions^32^. Conversely, the acyclic Ru(II)porphyrinate analog yields a product mixture composed of the carbene dimer and the mono/double inserted threads, highlighting the distinct reaction outcomes afforded by the two Ru(II)porphyrinates under the same conditions. A detailed structural investigation of the Ru(II)porphyrinate macrocyclic complex and the resulting [2]rotaxanes reveals the basic steps in which the macrocyclic Ru(II)porphyrinate operates to achieve the striking single N–H bond carbene insertion selectivity. To demonstrate the synthetic generality of the present methodology, we also describe the quantitative active metal template synthesis of the parent thioether-containing rotaxane, which is assembled through the S–H carbene insertion reaction. The findings reported herein demonstrate how the steric features of porphyrinate molecular capsules in combination with formation of mechanical bonds can be explored to change the reaction outcomes of challenging chemical transformations; the fundamental principle of nanoreactors.

## Results

### Molecular design and synthesis of the macrocyclic porphyrin-based ligand

**Fig. 1:**
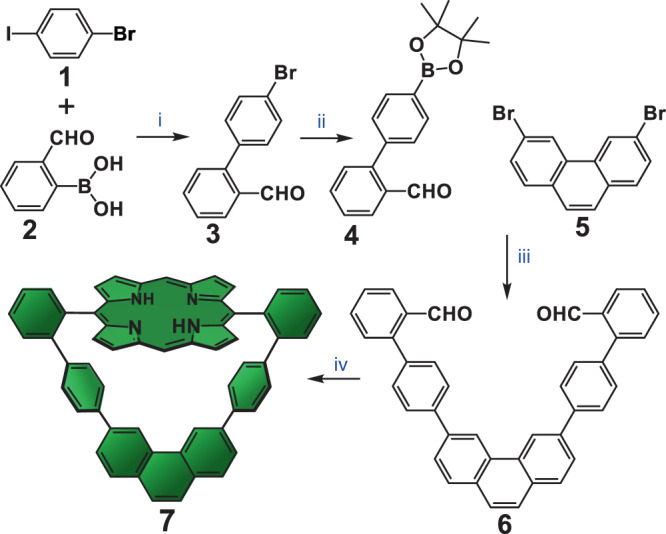
Synthetic strategy for the preparation of macrocycle **7**. Reagents and conditions: (i) Pd(PPh_3_)_4_, Na_2_CO_3(aq),_ MePh/EtOH, reflux, 16 h, N_2_ atmosphere, 80% yield; (ii) Pd(Cl)_2_dppf, B_2_pin_2_, 1,4-dioxane, reflux, 16 h, N_2_ atmosphere, 85% yield; (iii) Pd(PPh_3_)_4_, Na_2_CO_3(aq),_ PPh_3_, MePh/MeOH, reflux, 48 h, N_2_ atmosphere, 95% yield; (iv) dipyrromethane, TFA, CH_2_Cl_2_/CHCl_3_ (3:1, v/v), high dilution (0.213 mM), rt, 20 h, followed by DDQ, CH_2_Cl_2_, reflux, 2 h, 20% yield. Macrocycle **7** is prepared in 13% total yield relative to **2**.

**Fig. 2:**
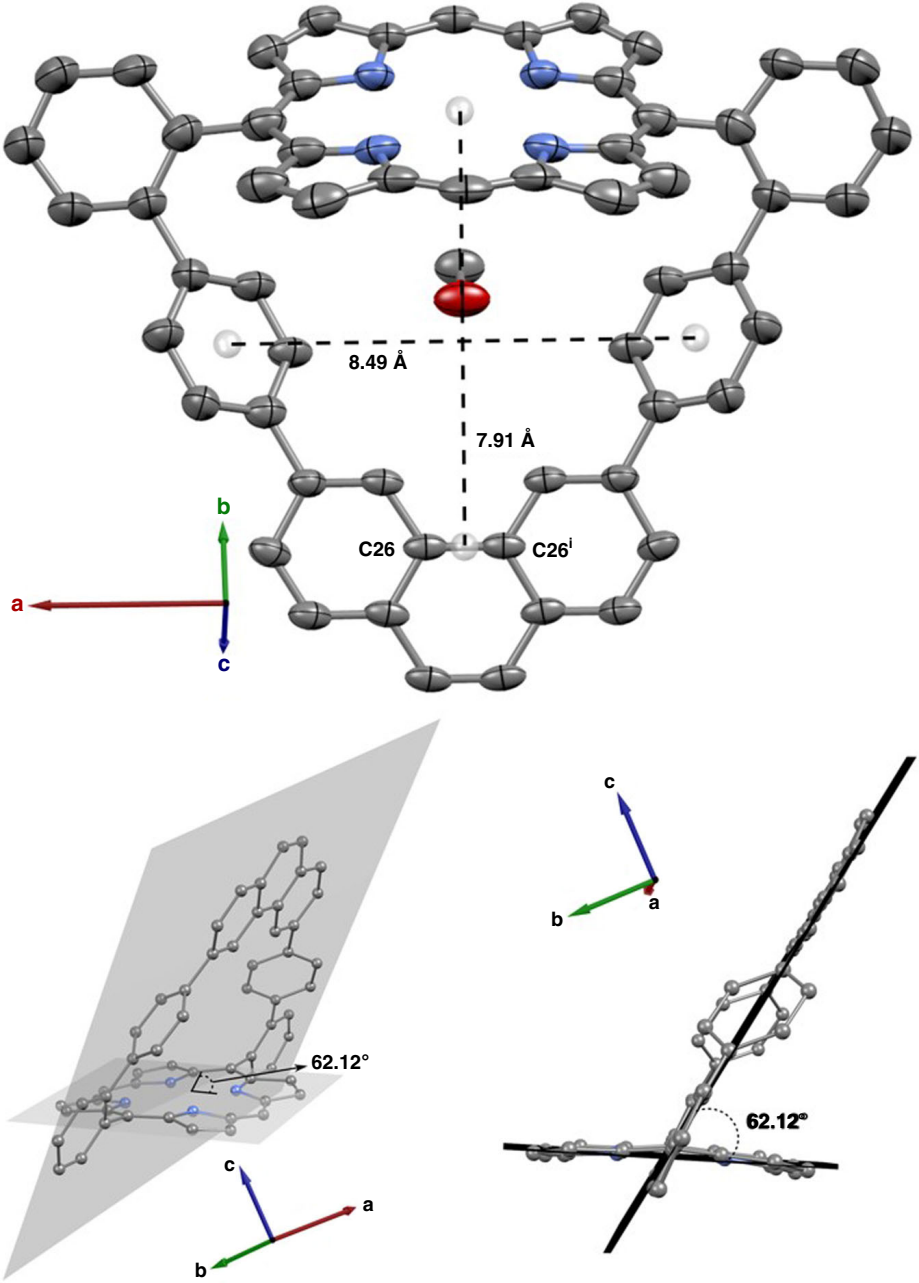
Representation of the crystal structure of macrocycle **7**. Single crystals were grown from a dichloromethane/methanol/tetrahydrofuran saturated solution by slow evaporation. Carbon atoms are shown in gray, nitrogen in blue and oxygen in red. Hydrogen atoms are omitted for clarity purposes. Ellipsoids are drawn at 50% probability levels. Symmetry code: *i* = 1/2-x, y, z. See Supplementary Fig. S1 for complete atom numbering. CCDC ID 1883084.

The molecular design of the macrocyclic receptor **7** (Fig. 1) contemplates the structural requirements for the successful exploration of carbene insertion reactions. The molecular backbone of macrocycle **7** is exclusively composed of aromatic C–H bonds, which are chemically inert to Ru(II)-carbenoids^34–42^. The rigid aromatic backbone inevitably leads to formation of a well-defined and relatively small central cavity in **7**. The crystal structure (CCDC ID 1883084) of **7** (Fig. 2) reveals a virtually flat porphyrin core which together with the aromatic backbone creates a central cavity with dimensions of 8.49 and 7.91 Å (centroid-to-centroid of the phenyl spacers and porphyrin centroid-to-midpoint of C26–C26^i^ bond on the phenanthrene moiety, respectively, with symmetry code: *i* = 1/2-x, y, z; see Supplementary Fig. S1 for complete atom numbering). Such cavity dimensions in **7** are rather smaller than those observed for a similar but semi-rigid porphyrin-based macrocycle reported in our previous works^24,25^ (cavity dimensions 10.30 and 8.40 Å).

The angle between the porphyrin and the molecular aromatic loop mean planes is 62.12°, which is smaller than the expected 90°. Although that indicates that the molecular loop and porphyrin mean planes are not orthogonal in the solid state, NMR investigation informs that the *meso*-aryl rings are rapidly oscillating through their perpendicular orientation to the porphyrin mean plane^43^, which in turn allow the phenanthrene moiety to rapidly swing back-and-forth below the porphyrin centroid in solution. Such fast swinging movement renders the two faces of macrocycle **7** magnetically equivalent. Accordingly, all resonances are sharp and distinct in the ^1^H NMR spectrum of **7** (Supplementary Fig. S2), thus confirming the proposed hollow structure in solution (see Supplementary material for further discussion).

### Structural and coordinative properties of the macrocyclic Ru(II)porphyrinate complexes

**Fig. 3:**
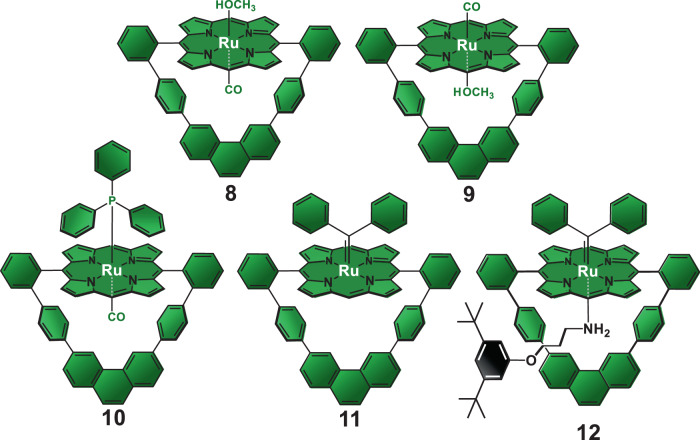
Molecular structures of the Ru(II)porphyrinate complexes. Complex **9** is not observed in our experiments.

With the structural requirements for a porphyrin-based macrocyclic ligand satisfied by **7**, we turned our attention to prepare the corresponding Ru(II)porphyrinate using the classical metalation protocol with Ru_3_(CO)_12_ as the metal source followed by purification in the presence of methanol^36^. In principle, metalation of macrocycle **7** under those conditions should have afforded a mixture composed of Ru(II)porphyrinate **8** and its isomer **9** (Fig. 3) as the central cavity provides no steric shielding for the selective coordination of small axial ligands such as carbonyl and methanol molecules. However, only one porphyrin product is isolated from the crude mixture. The ^1^H NMR spectrum of the isolated product is not consistent with the proposed isomeric mixture and reveals the expected set of signals for a single Ru(II)porphyrinate-based macrocycle (Supplementary Fig. S3)^35^. Congruently, ^13^C NMR and FTIR-ATR analyses (Supplementary Figs. S4 and S5, respectively) show one single resonance at *δ* = 180.1 ppm and one stretching band (${\nu}_{{\mathrm{CO}}_{\mathrm{stretch}}}$ = 1926 cm^−1^) for the carbonyl axial ligand in the isolated product, thus confirming formation of one single complex. Such coordinative properties are not ubiquitous as performing the metalation reaction under the same conditions used for **7** but with our previously reported^24,25^ semi-rigid and larger macrocycle as ligand yields the two expected isomers as revealed by ^1^H NMR spectroscopy (for spectrum and chemical structures, see Supplementary Fig. S6). Therefore, the structural features of **7**, which yield a hollow receptor with a relatively small central cavity, is most likely the reason for such selectivity in the metalation reaction.

To identify which isomer, **8** or **9**, is isolated from the metalation reaction of macrocycle **7**, we took advantage of the steric constraints imposed by the central cavity in **8** and **9**, along with the high affinity of Ru(II) ions for phosphorus-based ligands and the ring current effects provided by the porphyrin subunit. We reasoned that bulky triphenylphosphine species (PPh_3_) would easily displace the loosely bound and labile methanol axial ligand but not the kinetically robust carbonyl one in the coordination sphere of the Ru(II) ions in both isomers^44^. However, the internal axial position in isomer **9** would not be available for the incoming bulky PPh_3_ ligand due to the steric shielding provided by the appended aromatic loop. Conversely, the external position in complex **8** would be unencumbered, thereby allowing the substitution of the PPh_3_ ligand for the methanol one. If such ligand substitution reaction takes place, the ring current effects provided by the porphyrin core would shield the protons of the phenyl groups in the PPh_3_ ligand in the ^1^H NMR spectrum of the product. Therefore, the shielding of the PPh_3_ protons is a probe for the identification of the isolated product as complex **8** or **9** by ^1^H NMR spectroscopy.

Treatment of the unidentified Ru(II)porphyrinate with excess of PPh_3_ in dichloromethane at room temperature followed by precipitation of the crude with petroleum ether lead to the axial ligand substitution reaction to quantitatively yield complex **10** (Fig. 3). ^1^H NMR analysis (Supplementary Fig. S7) clearly reveals the strong shielding of the protons of the PPh_3_ axial ligand, hence giving unmistakable evidence for the exclusive formation of **10**.

For our purposes, complex **8** is useless as the stable and inert axial carbonyl ligand is coordinated to the Ru(II) ion inside the macrocycle’s cavity. Therefore, we investigated several protocols to substitute the internal carbonyl axial ligand in **8** with other ligands. Thermolysis^44^, photolysis^45^, and chemical oxidative decarbonylation reactions^46^ all failed in our hands, suggesting that the cavity provide the carbonyl ligand with great stability. Gratefully, treating **8** with diphenyldiazomethane^34^ in dichloromethane at room temperature followed by purification by column chromatography on neutral alumina affords target complex **11** (Fig. 3).

**Fig. 4:**
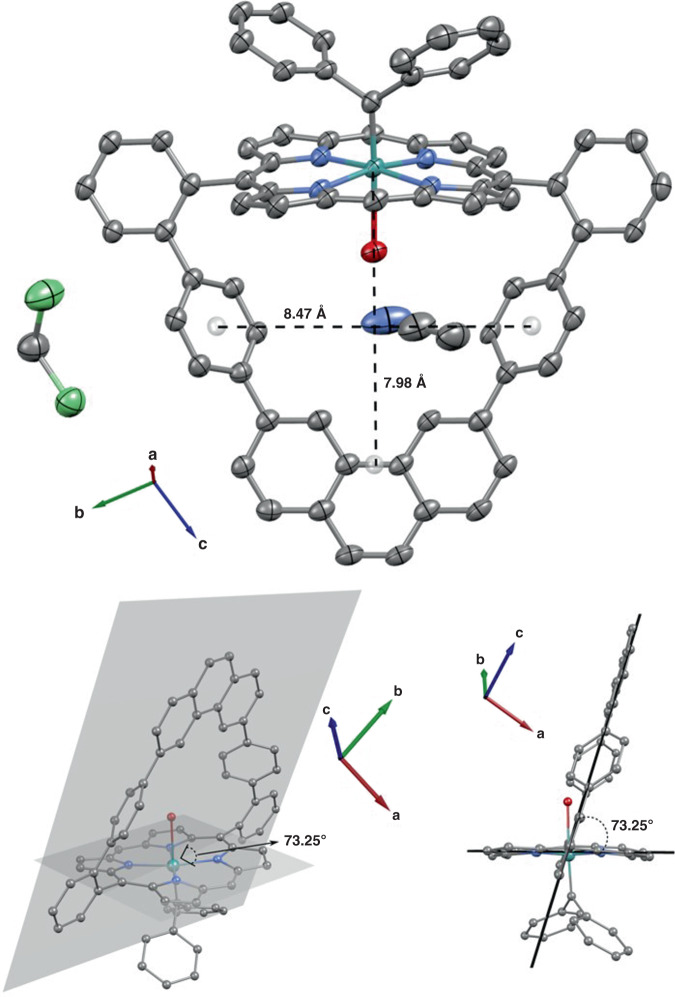
Representation of the crystal structure of macrocycle **11**. Single crystals were grown from a dichloromethane/acetonitrile saturated solution by slow evaporation. Carbon atoms are shown in gray, nitrogen in blue, oxygen in red, ruthenium in turquoise and chlorine in green. Hydrogen atoms are omitted for clarity purposes. Ellipsoids are drawn at 50% probability levels. CCDC ID 1988009.

**Fig. 5:**
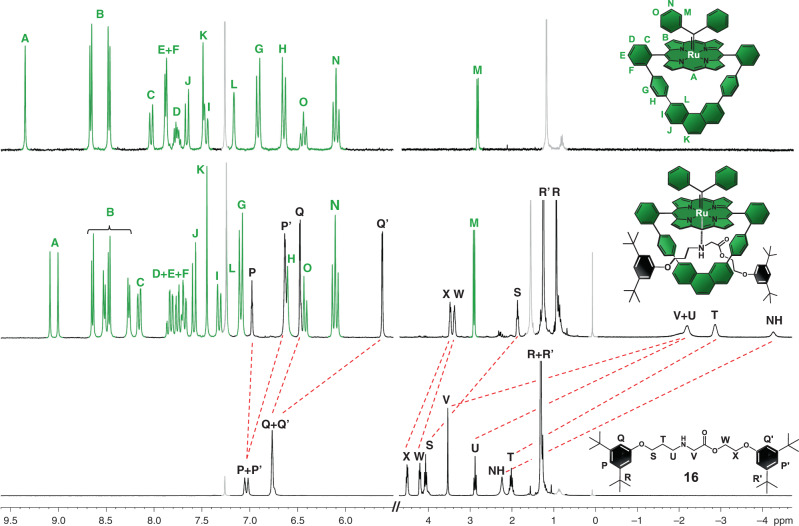
Selected regions of the ^1^H NMR spectra of macrocycle **11** (top), rotaxane **15** (middle) and thread **16** (bottom). Experimental conditions: 250 MHz, CDCl_3_, 298 K. Thread **16** was prepared aside for comparison purposes, using half-threads **13** and **14** as substrates and the acyclic version of **11** as promoter. Unambiguous proton assignments, including the H_L_ signal in rotaxane **15** that overlaps with that of residual chloroform in the deuterated solvent, are based on 2D-NMR spectroscopy. Residual solvent peaks and aliphatic impurities are in gray: chloroform (δ = 7.26 ppm), water (δ = 1.56 ppm), “grease” (δ = 1.26 ppm and δ = 0.88 ppm) and silicone “grease” (δ = 0.07 ppm)^52^.

The proposed molecular structure of complex **11** is confirmed by x-ray diffraction on single crystals grown from slow evaporation of a dichloromethane/acetonitrile saturated solution of **11** (Fig. 4, CCDC ID 1988009). The organic backbone of the complex is virtually identical to that of the free ligand macrocycle **7** (Fig. 2), including the cavity dimensions. The complex has a distorted octahedral symmetry with a residual water molecule bound to the internal axial position and with the Ru(II) ion displaced 0.1449(8) Å from the porphyrin mean plane toward the carbene ligand. The axial Ru–C bond distance in **11** is 1.852(4) Å and thus comparable to those reported for other similar carbene-Ru(II)porphyrinates (1.841–1.876 Å)^38^. However, the Ru–C bond distance in **11** is significantly shorter when compared to the single Ru–C bonds (1.978–2.088 Å)^42,47^ usually observed in ruthenium complexes with N-heterocyclic carbene ligands. The short Ru–C bond distance together with the displacement of the Ru(II) ion from the porphyrin mean plane towards the carbene ligand reveals the formation of a robust axial Ru–C coordinative interaction with partial double bond character in **11**^48^. Accordingly, the carbene carbon atom receives non-negligible electronic density back from the Ru ion (π-back donation)^48,49^. ^1^H NMR analysis on **11** confirms that the solid-state structural features are preserved in solution. The well-defined and sharp signals observed in the ^1^H NMR spectrum of **11** (Fig. 5, top) inform that the axial carbenoid ligand is inert in solution, which is also a consequence of the strong Ru–C axial bond. Such electronic interactions provide **11** with great thermal and chemical stability^47–49^, which in conjunction with the inertness of the carbenoid ligand in solution, yield the structural features required for an excellent endotopic promoter.

### Active-metal-template syntheses of asymmetrical [2]rotaxanes through carbene insertion reactions and the nanoreactor effect

**Fig. 6:**
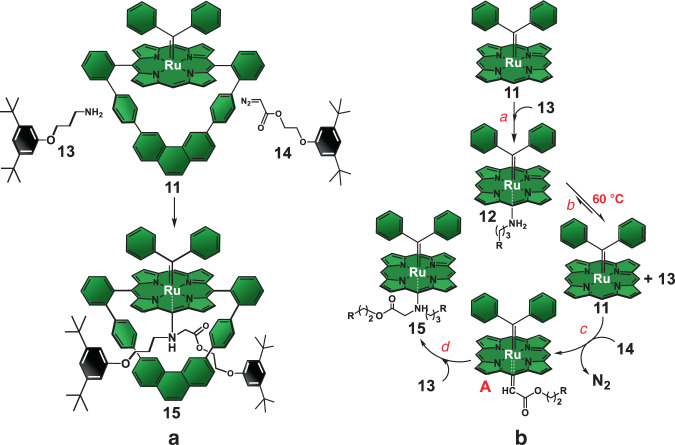
Operation of the nanoreactor. **a** Synthesis of asymmetrical [2]rotaxanes by the active-metal template technique based on the Ru(II)porphyrinate-promoted N-H carbene insertion reactions. Experimental conditions: benzene, 8 h, N_2_ atmosphere, quantitative yield relative to **11**. **b** Conceivable reaction mechanism for the carbenoid N–H insertion process promoted by the Ru(II)porphyrinate subunit in **11** that quantitatively yield asymmetrical [2]rotaxane **15** through the active-metal template technique. The aromatic loop in **11** is not shown in the mechanism for clarity purposes. R = 3,5-di-*tert*-butylphenoxy stopper groups.

To demonstrate the endotopic properties of complex **11**, we developed an active-metal template synthesis^50,51^ of an asymmetrical [2]rotaxanes through the problematic N–H carbene insertions (Fig. 6a)^40,41^ under relatively severe experimental conditions. Accordingly, we decided to add in one portion a relatively large excess of half-threads **13** and **14** (10 equiv) relative to macrocycle **11** (1 equiv). After 3 hours at room temperature, no reaction was observed. That inactivity is due to the coordination of the amino group in **13** to the internal axial position of the Ru(II) ion in **11** to form hexacoordinated complex **12** (Fig. 3), whose structure is unambiguously confirmed by comparing the ^1^H NMR spectra of complex **12**, macrocycle **11** and half-thread **13** (see Supplementary Fig. S8 for spectra and further discussion).

The RH_2_N–Ru coordinative bond in **12** is inert at room temperature and shuts down the *α*-estercarbenoid production by **11** from diazo half-thread **14**^34–42^. However, the interaction becomes labile at higher temperatures as heating the reaction mixture at 60 °C results in smooth and total interlocking of macrocycle **11**. Significantly, the excess of half-threads **13** and **14** added to the reaction medium is completely recovered as unreacted materials after purification of the crude. No signs of noninterlocked threads (from reactions occurring *exo*-to the macrocycle’s cavity) or rotaxane by-products (from intercomponent reactions)^32^ are observed in our experiments. Those findings are surprising and convincingly demonstrate that macrocycle **11** shows chemical selectivity towards promoting a single N–H insertion. Most importantly, the single insertions occur exclusively through the macrocycle’s cavity. Hence, macrocycle **11** is an exceptional endotopic catalyst.

For comparison purposes, we performed the N–H carbene insertion reaction using as promoter the 5,15-*bis*(phenyl)Ru(II)porphyrinate with the diphenylcarbene axial ligand, which functions as the acyclic version of macrocycle **11**, and **13** and **14** as substrates. Under the exact same conditions as those of the rotaxane assembly process, the acyclic Ru(II)porphyrinate leads to total consumption of **14** to afford a small amount of the respective dimer thread along with a mixture composed of the expected thread **16** (26% isolated yield relative to **14**; for structure of **16** see Fig. 5, bottom) and the tertiary amine counterpart (65% isolated yield relative to **14**; for structure see Supplementary Fig. S27), which is formed from the second carbenoid insertion into the secondary amine group of thread **16**. Those findings unequivocally demonstrate that **11** functions as a molecular nanoreactor^33^ as the selectivity towards single N–H carbenoid insertions is unachievable in bulk solution under the conditions investigated.

A complete spectroscopic characterization of the structure of rotaxane **15** by 2D-NMR (see Supplementary Figs. S81–S87) allows unambiguous assignment of all resonances, which along with MALDI-TOF mass spectrometry (Supplementary Fig. S9) provide unequivocal evidence for the proposed interlocked architecture for **15** shown in Fig. 6a. Furthermore, the unique structural features of the interlocked architecture of **15**, revealed by the NMR investigation, provide valuable insights into the nature of the highly chemical selectivity observed in the rotaxane assembly process (vide infra).

In the ^1^H NMR spectrum of **15** (Fig. 5, middle), one observes the duplication of the resonances of the *meso*-nuclei (H_A_) and the pyrrolic protons (H_B_) when compared to that of macrocycle **11**, which is consistent with an asymmetrical [2]rotaxane architecture. The axial coordination of diphenylcarbene ligand is warranted by the observation of the diagnostic shielding of the resonances of protons H_M-O_ as well as by the characteristic strongly de-shielded carbene nuclei resonance at *δ* = 330.3 ppm in the ^13^C NMR spectrum of **15** (Supplementary Fig. S10)^34–42^. Two dimensional NOESY NMR analysis (Supplementary Fig. S11) reveals NOEs between protons H_Q’_, H_S_, H_T_, H_U_ H_V_ and NH on the thread component and H_G_, H_H_, H_L_ and H_B_ on the macrocycle, thus confirming the formation of the mechanical bond in **15**.

Resonances associated with the thread component (Fig. 5, bottom) informs about the structural peculiarities of rotaxane **15**. The secondary N–H moiety is drastically shielded (Δ*δ* = 6.49 ppm) in rotaxane **15** when compared to noninterlocked thread **16**, revealing the coordination of the nitrogen atom of the secondary amine group on the thread to the Ru(II) ion on the macrocycle. As a result of such intercomponent chemical interaction, all nuclei on the thread (H_S-X_), including those on the stoppers (H_P-R_), are also shielded, indicating that the ring virtually covers the whole thread component in rotaxane **15**.

The intercomponent interaction is inert at room temperature and, remarkably, at 333 K (60 °C) as revealed by the variable temperature-NMR analysis of **15** (Supplementary Fig. S12), which is unusual for this type of coordinative bond^40,41^. The R_1_R_2_HN–Ru axial interaction in **15** is kinetically stabilized by the mechanical bond, which constantly keeps the secondary NH binding group on the thread close to the Ru(II) ion on the macrocycle.

To generalize our method, macrocycle **11** was also investigated as promoter for S–H carbene insertions (Supplementary Fig. S13). By mixing thiol half-thread **17** (10 equiv) with macrocycle **11** (1 equiv) followed by one portion addition of half-thread **14** (10 equiv) in benzene at room temperature yields rotaxane **19** (Supplementary Fig. S13) in quantitative yield after 4 hours with complete recovering of the excess of substrates **14** and **17** as starting materials after workup. No signs of by-products were observed in the assembly process of **19** as in the case of the parent rotaxane **15**. Most importantly, quantitative formation of rotaxane **19** informs that the S–H carbene insertion occurs at room temperature rather than at 60°C as necessary for the N–H insertion reaction. A complete structural characterization of rotaxane **19** by 2D-NMR spectroscopy (see Supplementary Figs. S15–S22) allows unambiguous assignment of all resonances and informs that the structural features of rotaxane **19** are very similar to that observed for rotaxane **15** analog. MALDI-TOF mass spectrometry (Supplementary Fig. S23) confirms the interlocked architecture for **19** by revealing the correct ion mass peak at 1624.446 (calculated for C_106_H_98_N_4_O_4_SRu) along with the characteristic fragmentation pattern expected for that type of rotaxanes^24,25,52^.

## Discussion

The endotopic and chemical selectivity observed for **11** under the demanding conditions investigated for the N–H carbene insertions in the rotaxane synthesis along with its distinct reaction outcome when compared to that afforded by the acyclic Ru(II)porphyrinate can be explained by three main factors, which are summarized in the proposed reaction mechanism depicted in Fig. 6b. Firstly, the lack of non-interlocked threads in the crude mixture, being them formed from carbene dimerization and/or mono/double N–H insertions is warranted by the inert and stable diphenylcarbene axial ligand coordinated *exo*-to the macrocycle’s cavity in **11**. Such stability towards potential nucleophilic attack by the amine moiety in **13** and inertness towards ligand dissociation of the diphenylcarbene axial ligand are due to the significant electronic π-back donation from the Ru(II) ion to the carbene carbon as revealed by the short Ru–C bond (1.852(4) Å) measured in the crystal structure of **11** (Fig. 4) and verified in solution by NMR spectroscopy. Accordingly, only the internal axial position of **11** is available for activation of the substrates.

Secondly, coordination of the amino group in **13** to the internal axial position of **11** yields complex **12** (*step a*, Fig. 6b) prior to the initiation of the organometallic cycle. Formation of complex **12**, which is inert at room temperature, informs about the pre-association of **11** and **13**. Heating the reaction solution at 60°C renders complex **12** labile (*step b*, Fig. 6b), which means that half-thread **13** enters and leaves the coordination sphere of the Ru(II) ions **11** rapidly. Therefore, half-thread **13** is held nearby the Ru(II) ion during the rotaxane assembly process. As soon as the *α*-estercarbenoid intermediate **A** is formed^34–42^ from the reaction between half-thread **14** and **11** after N_2_ extrusion (*step c*, Fig. 6b), the relatively stronger nucleophilic power of the amino group in **13** associated with its enforced local concentration near the cavity kinetically favor the N–H insertion (*step d*, Fig. 6b) instead of the deleterious carbene dimerization. Therefore, symmetrical rotaxanes from dimerizations are not observed in our experiments.

Finally, the kinetic stabilization of the axial R_1_R_2_HN–Ru coordinative interaction by the mechanical bond in **15**, in conjunction with the somewhat short molecular linkage between the two stoppers, force the latter to be held in front of the faces of the macrocyclic component in the rotaxane architecture. The steric shielding of both macrocycle’s faces by the two stoppers associated with the small cavity provided by the rigid aromatic backbone completely block the access of exogenous substrates to the Ru(II) ions after rotaxane formation. Accordingly, the synergy between such steric and electronic effects shuts down the Ru(II) ion activity in **15** and explain the selectivity of the process towards the single N–H insertion, even in the presence of a large excess of half-thread **14** at 60 °C. Conversely, the experiment promoted by the acyclic Ru(II)porphyrinate demonstrates that after the first N–H carbene insertion that afford thread **16**, the resulting axial R_1_R_2_HN–Ru interaction is labile due to the lack of the mechanical bond. Congruently, **16** dissociates from the acyclic Ru(II)porphyrinate to allow the turnover of the Ru(II) ions, which then further react with additional **14** molecules present in excess in the reaction medium to generate the *α*-estercarbenoid intermediate. The lack of the steric shielding in the acyclic Ru(II)porphyrinate permits the secondary amine group on **16** to attack the carbene intermediate to mostly afford the double inserted product.

The similar reaction outcome of the S–H insertion when compared to that of the N–H counterpart suggests that the former reaction should take place through the same mechanism proposed in Fig. 6b. In other words, coordination of the thiol group in **17** to the internal axial position of the Ru(II) ion in **11** should occur to form the hexacoordinated intermediate complex **18** (Supplementary Figure S13). However, the RHS–Ru interaction is labile at room temperature, and complex **18** does not require heating to generate carbenes from half-thread **14** as in the case of the parent complex **12** (Fig. 3).

In conclusion, a rigid porphyrin-based hollow receptor with a relatively small and well-defined central cavity revealed unusual coordinative properties as ligand for Ru(II) ions. Capitalizing on that, we developed a macrocyclic Ru(II)porphyrinate receptor bearing a stable and inert diphenylcarbene axial ligand, which functions as a nanoreactor and alters the outcome of N–H bond carbene insertions when compared to that of its acyclic analog. The findings reported herein establish the basis for the synergetic combination between the steric properties of porphyrinate molecular capsules and formation of mechanical bonds as principles for the design of nanoreactors in order to gain control over the product distribution of challenging chemical transformations. The synthetic utility of the present methodology relies on the conversion of the substrates into mechanical bonds with quantitative efficiency via either N–H or S–H carbene insertions even in the presence of substrate excess. As formation of the mechanical bond blocks the turnover of the Ru(II) ions through a combination of steric and electronic effects, each Ru(II)porphyrinate promotes one single carbene insertion, thus no intercomponent nor side-reactions are observed. The opportunity now exists to apply the technique described herein to assemble interlocked polymers with well-defined structures and low polydispersities. Increasingly, we can look forward to the preparation of copolyrotaxanes as we can now use the reaction temperature to selectively control the carbene insertions either in the S-H or N-H bonds. Such interlocked polymers and copolymers will allow a fundamental investigation of the effects of the inherent dynamic processes of mechanical bonds^52^ in the properties of macromolecules. Research along those lines is in progress.

## Methods

### Synthesis of macrocycle 11

In a 100 mL Schlenk flask, macrocycle **8** (0.010 g, 0.011 mmol, 1 equiv) was dissolved in 20 mL of dichloromethane under magnetic stirring at room temperature and inert atmosphere. A dichloromethane solution of freshly prepared diphenyldiazomethane (0.008 g, 0.040 mmol, in 4.00 mL of dichloromethane) was added dropwise to the solution of **8** over 3 h. At the end of the addition, the reaction mixture was magnetically stirred for another 1 h at room temperature. The solvent was removed under reduced pressure and the crude product was purified by chromatography column on neutral alumina using a mixture of petroleum ether/dichloromethane (1:1, v/v) as eluent to afford macrocycle **11** as a red solid in 64% yield (7.4 mg). TLC (petroleum ether:CH_2_Cl_2_, 1:1 v/v): Rf = 0.56.; ^1^H NMR (400 MHz, CDCl_3_): *δ* 9.34 (s, 2H, H_A_); 8.66 (d, *J* = 4.74 Hz, 4H, H_B_); 8.47 (d, *J* = 4.70 Hz, 4H, H_B_); 8.03 (d, *J* = 7.36 Hz, 2H, H_C_); 7.90-7.85 (m, 4H, H_E_ and H_F_); 7.79-7.73 (m, 2H, H_D_); 7.65 (d, *J* = 8.20 Hz, 2H, H_J_); 7.49 (s, 2H, H_K_); 7.46 (d, J = 8.39 Hz, 2H, H_I_); 7.17 (s, 2H, H_L_); 6.91 (d, J = 8.17 Hz, 4H, H_G_); 6.65 (d, *J* = 8.17 Hz, 4H, H_H_); 6.44 (t, *J* = 7.30 Hz, 2H, H_O_); 6.10 (t, *J* = 7.56 Hz, 4H, H_N_); 2.88 (d, *J* = 7.56 Hz, 4H, H_M_). Impurities: 5.30 (dichloromethane); 1.26 and 0.88 (aliphatic impurities); ^13^C NMR (60 MHz, CDCl_3_): Macrocycle **11** was too insoluble to record ^13^C NMR. MALDI-TOF (m/z): [M]^+^ calcd. for C_71_H_44_N_4_Ru, 1054.260; found 1054.191. UV-Vis (CH_2_Cl_2_), 10^-5^ mol/L, *λ*_max_ (nm): 274, 317, 391, 424, 527 and 550.

### Synthesis of rotaxane 15

In a 10 mL Schlenk flask, macrocycle **11** (10 mg, 9.4 μmol, 1.0 equiv) and half-thread **13** (24.7 mg, 94.0 μmol, 10.0 equiv) were dissolved in 1.2 mL of benzene under inert atmosphere at room temperature. The resulting solution was stirred for 15 minutes at room temperature. Compound **14** (29.9 mg, 94.0 μmol, 10.0 equiv) was added in one portion as a solid to the reaction flask and the mixture was heated at 60°C for 8 hours. The crude mixture was evaporated to dryness under reduced pressure. The viscous crude product was dissolved in a minimum amount of petroleum ether. The excess of half-thread **14** was insoluble in the petroleum ether phase and was removed using a pipette as a colorless oil. The petroleum-ether solution was further purified by preparative TLC on silica using petroleum ether/dichloromethane (1:1, v/v) as eluent to afford the target rotaxane **15** as a red solid in quantitative yield relative to **11** (15.0 mg) as the first fraction. The excess of half-thread **13** was isolated as the second fraction as a colorless oil. TLC (petroleum ether:CH_2_Cl_2_, 1:1 v/v): Rf = 0.79. ^1^H NMR (250 MHz, CDCl_3_): *δ* 9.09 (s, 1H, H_A_); 9.00 (s, 1H, H_A_); 8.64 (d, *J* = 4.83 Hz, 2H, H_B_); 8.52 (d, *J* = 4.59 Hz, 2H, H_B_); 8.47 (d, *J* = 4.83 Hz, 4H, H_B_); 8.26 (d, *J* = 4.83 Hz, 4H, H_B_); 8.16 (d, *J* = 7.49 Hz, H_C_); 7.88-7.65 (m, 6H, H_D_, H_E_ and H_F_); 7.58 (d, *J* = 8.29 Hz, 2H, H_J_); 7.44 (s, 2H, H_K_); 7.32 (d, *J* = 8.29, 2H, H_I_); 7.26 (s, 2H, H_L_); 7.09 (d, *J* = 8.29, 4H, H_G_); 6.98 (t, *J* = 1.48 Hz, 1H, H_P’_); 6.66-6.58 (m, 5H, H_P_ and H_H_); 6.47 (d, *J* = 1.43, 2H, H_Q’_); 6.43 (t, *J* = 7.36, 2H, H_O_); 6.10 (t, *J* = 7.73, 4H, H_N_); 5.61 (d, *J* = 1.52 Hz, H_Q_); 3.48 (br, 2H, H_X_); 3.37 (br, 2H, H_W_); 2.91 (d, *J* = 7.10, 4H, H_M_); 1.87 (t, *J* = 5.35 Hz, 2H, H_S_); 1.25 (s, 18H, H_R’_); 0.94 (s, 18H, H_R_); from −1.76 to −2.45 (br, 4H, H_V_ and H_U_); −2.86 (br, 2H, H_T_); −4.25 (br, 1H, NH). Impurities: 1.56 (residual water in the CDCl_3_ solvent); 0.07 (silicon “grease”). ^13^C NMR (60 MHz, CDCl_3_): *δ* 330.3; 168.2; 162.7; 158.0; 157.9; 152.2; 151.3; 144.6; 144.1; 143.9; 143.4; 143.1; 141.3; 140.3; 140.0; 139.7; 134.9; 133.1; 132.8; 131.8; 131.6; 130.8; 129.9; 129.6; 129.5; 128.1; 127.9; 126.2; 125.8; 125.2; 123.4; 123.1; 118.4; 115.2; 113.9; 111.6; 108.8; 108.0; 107.4; 107.0; 64.7; 64.3; 62.4; 53.5; 46.1; 44.2; 35.0; 34.6; 31.5; 31.3; 29.8; 22.8; 22.1; 14.2. Impurities: 53.5 (residual dichloromethane); 29.8; 22.8 and 14.2 (aliphatic impurities). MALDI-TOF (*m/z*): [M]^+^ calcd. for C_106_H_99_N_5_O_4_Ru, 1607.6741; found 1607.603. UV-Vis, *λ*_max_ (nm): 270, 313, 398, 425 and 530. FTIR (ATR), ν (cm^−1^): 1741.

### Synthesis of rotaxane 19

In a 10 mL Schlenk flask, under inert atmosphere, macrocycle **11** (10 mg, 0.0094 mmol, 1.0 equiv) and half-thread **17** (26.0 mg, 0.094 mmol, 10.0 equiv) were dissolved in 1.2 mL of benzene at room temperature. The resulting solution was stirred for 15 minutes at rt. Compound **14** (29.9 mg, 0,094 mmol, 10.0 equiv) was added as a solid to the reaction flask and the mixture was magnetically stirred at room temperature for 4 hours. TLC analyses on silica revealed that macrocycle **11** was completely interlocked after that period. The crude mixture was evaporated to dryness under reduced pressure, dissolved in a minimum amount of petroleum ether and purified by preparative TLC on silica using petroleum ether/dichloromethane (50:50, v/v) as eluent to afford the target rotaxane **19** as a red solid in quantitative yield (91% isolated yield; 0.014 mg) as the second fraction. The excess of half-threads **17** and **14** was completely recovered as the first (colorless oil) and third (yellowish oil) fractions, respectively. TLC (petroleum ether:CH_2_Cl_2_, 1:1 v/v): Rf = 0.79. ^1^H NMR (500 MHz, CDCl_3_): *δ* 9.16 (s, 1H, H_A_); 9.08 (s, 1H, H_A_); 8.63 (d, *J* = 4.55 Hz, 2H, H_B_); 8.53 (d, *J* = 4.73 Hz, 2H, H_B_); 8.48 (d, *J* = 4.55 Hz, 4H, H_B_); 8.36 (d, *J* = 4.77 Hz, 4H, H_B_); 8.23 (d, *J* = 6.57 Hz, H_C_); 7.84 (td, *J* = 7.62 Hz e 1.01 Hz, 2H H_D_); 7.75 (td, *J* = 7.53 Hz e 1.28 Hz, 2H, H_E_); 7.70 (d, *J* = 7.67, 2H, H_F_); 7.58 (d, *J* = 8.26 Hz, 2H, H_J_); 7.44 (s, 2H, H_K_); 7.37 (s, 2H, H_L_); 7.33 (d, *J* = 8.35, 2H, H_I_); 7.03 (d, *J* = 8.26, 4H, H_G_); 6.94 (br, 1H, H_P’_); 6.71 (br, 1H, H_P_); 6.65 (d, *J* = 8.37 Hz, 4H, H_H_); 6.49 (t, *J* = 7.44, 2H, H_O_); 6.39 (s, 2H, H_Q’_); 6.15 (t, *J* = 7.79, 4H, H_N_); 5.85 (s, 2H, H_Q_); 3.18 (br, 2H, H_X_); 2.99 (d, *J* = 7.25, 4H, H_M_); 2.73 (br, 2H, H_W_); 2.38 (br, *J* = 5.35 Hz, 2H, H_S_); 1.23 (s, 18H, H_R’_); 1.00 (s, 18H, H_R_); −1.76 (br, 2H, H_V_); −1.90 (br, 2H, H_U_); −2.05 (s, 2H, H_T_). Impurities: 1.58 (residual water); 1.26 e 0.07 (aliphatic impurities).^13^C NMR (60 MHz, CDCl_3_): *δ* 167.3; 161.4; 158.0; 157.7; 152.2; 151.7; 144.9; 144.5; 143.8; 143.2; 143.1; 141.0; 140.2; 139.7; 139.4; 135.0; 133.0; 132.7; 131.9; 131.7; 130.8; 130.1; 129.8; 129.7; 128.3; 128.2; 128.0; 126.2; 125.7; 125.4; 125.2; 123.9; 123.6; 119.0; 115.2; 114.2; 112.6; 108.6; 108.1; 107.4; 107.0; 65.4; 64.4; 62.5; 35.0; 34.7; 31.5; 31.3; 29.8; 28.6; 28.0; 23.7. Impurities: 29.8; 22.8 e 14.2 (aliphatic impurities). MALDI-TOF (*m/z*): [M]^+^ calcd. for C_106_H_98_N_4_O_4_Ru, 1624.635; found 1624.446.

### NMR experiments

^1^H, ^13^C, ^31^P, COSY, HMBC and HSQC NMR spectra were obtained on either a Bruker AVANCE 250 (250 MHz) or a Bruker AVANCE 500 (500 MHz), in all cases using deuterated solvents as the lock. The spectra were collected at 298 K or 333 K, and chemical shifts reported in parts per million (*δ*, ppm) were referenced to residual solvent peak. Residual solvent peaks and eventual aliphatic impurities were assigned according to literature^53^. Two dimensional NOESY NMR spectra were acquired on a Bruker AVANCE 400 (400 MHz) using CDCl_3_ as deuterated solvent at 298 K and 400 ms mixing time.

### Single-crystal X-ray diffraction measurements

X-Ray quality crystals of macrocycles **7** and **11** were grown from slow evaporation of a dichloromethane/methanol/tetrahydrofuran and dichloromethane/acetonitrile saturated solutions, respectively. Both compounds crystalized as red needle-like single crystals with approximate dimensions of 0.025 mm × 0.01 mm × 0.05 mm for macrocycle **7** and 0.27 × 0.08 × 0.08 mm for macrocycle **11**. The X-ray diffraction experiments were performed at the MX2 beamline at the UVX synchrotron source at the Brazilian Synchrotron Light Source. For further information about the X-ray experiments, see Supplementary Material.

## Supplementary information

Supplementary Information

## Data Availability

All the data generated or analyzed during this study are included in this published article (and its supplementary information files) or available from the authors upon reasonable request. The X-ray crystallographic coordinates for structures reported in this study have been deposited at the Cambridge Crystallographic Data Centre (CCDC), under deposition numbers 1883084 and 1988009. These data can be obtained free of charge from The Cambridge Crystallographic Data Centre via www.ccdc.cam.ac.uk/data_request/cif.
