# Supplementary material for: Ru(II)Porphyrinate-based molecular nanoreactor for carbene insertion reactions and quantitative formation of rotaxanes by active-metal-template syntheses
Source: Nat Commun. 2020 Dec 11;11:6370. doi: 10.1038/s41467-020-20046-x (PMC7733472; doi:10.1038/s41467-020-20046-x)
Supplement: Supplementary file 1 — Supplementary Information [file 41467_2020_20046_MOESM1_ESM.pdf]

Ru(II)Porphyrinate-Based Molecular Nanoreactor for Carbene Insertion Reactions and Quantitative Formation of Rotaxanes by Active-Metal-Template Syntheses

Liniquer A. Fontana,[1] Marlon P. Almeida,[1] Arthur F. P. Alcântara,[1,2] Vitor H. Rigolin,[1] Marcos A. Ribeiro,[3] Wdeson P. Barros[1] and Jackson D. Megiatto, Jr.[1,*]

[1]Institute of Chemistry, University of Campinas (UNICAMP), POBox 6154, 13083-970, Campinas, Brazil.

[2]Instituto Federal do Sertão Pernambucano, Estrada do Tamboril, 56200-000, Ouricuri, Brazil.

[3]Universidade Federal do Espírito Santo, Departamento de Química, Av. Fernando Ferrari, 514, 29075-910, Vitória, Brazil.

SUPPORTING INFORMATION

Table of contents :

General Information.--- S3
Spectroscopic Data and Chemical Structures of Compounds Mentioned in the Manuscript.-- S5
Figure S1 – Estimated cavity size afforded from the crystal structure of macrocycle **7**--- S5
Figure S2 – Comparison of the aromatic region of ^{1}H NMR spectra of compound **6**------ (top) and macrocycle **7** (bottom)--- S6
Figure S3 – ^{1}H NMR spectrum of the porphyrinate product isolated after metalation reaction of macrocycle **7** with $Ru_3(CO)_{12}$-- S7
Figure S4 – ^{13}C NMR spectrum of the porphyrinate product isolated after metalation reaction of macrocycle **7** with $Ru_3(CO)_{12}$-- S8
Figure S5 – Carbonyl stretching frequency region of the FTIR-ATR spectrum of the porphyrinate product isolated after metalation reaction of macrocycle **7** with $Ru_3(CO)_{12}$. S8
Figure S6 – ^{1}H NMR spectrum of the semi-rigid porphyrin-based macrocycle reported in our previous works[S4,S5] after its metalation reaction with $Ru_3(CO)_{12}$--------------------- S9
Figure S7 – Selected region of the ^{1}H NMR spectra of pristine triphenylphosphine and complex **10**--- S10
Figure S8 – Comparison between the ^{1}H NMR spectra of macrocycle **11**, complex **12** and half-thread **13**-- S11
Figure S9 – MALDI-TOF mass spectrum of rotaxane **15**------------------------------------ S13
Figure S10 – ^{13}C NMR spectrum of rotaxane **15**-- S14
Figure S11 – Selected regions of the two-dimensional (^{1}H-^{1}H) NOESY NMR spectrum of rotaxane **15**--- S15
Figure S12 – ^{1}H NMR (500 MHz, C_6D_6) spectra of rotaxane **15** at 333 K----------------- S17
Figure S13 – Synthesis of asymmetrical [2]rotaxanes **19** by the active-metal template technique based on the Ru(II)porphyrinate-promoted S-H carbene insertion reactions--- S18
Figure S14 – ^{1}H NMR spectrum of rotaxane **19** (250 MHz, $CDCl_3$, 298 K)---------------- S19
Figure S15 – Selected region of the two-dimensional (^{1}H-^{1}H) COSY NMR spectrum of rotaxane **19** (500 MHz, $CDCl_3$, 298 K)-- S19
Figure S16 – Selected region of the two-dimensional (^{1}H-^{1}H) COSY NMR spectrum of rotaxane **19** (500 MHz, $CDCl_3$, 298 K)-- S20
Figure S17 – Selected region of the two-dimensional (^{1}H-^{1}H) COSY NMR spectrum of rotaxane **19** (500 MHz, $CDCl_3$, 298 K)-- S21
Figure S18 – Two-dimensional (^{1}H-^{1}H) NOESY NMR spectrum of rotaxane **19** (250 MHz, $CDCl_3$, 298 K)--- S22
Figure S19 – Selected region of the two-dimensional (^{1}H-^{1}H) NOESY NMR spectrum of rotaxane **19** (250 MHz, $CDCl_3$, 298 K)-- S23
Figure S20 – Selected region of the two-dimensional (^{1}H-^{13}C) HSQC NMR spectrum of rotaxane **19** (500 MHz, $CDCl_3$, 298 K)-- S24
Figure S21 – Selected region of the two-dimensional (^{1}H-^{13}C) HSQC NMR spectrum of rotaxane **19** (500 MHz, $CDCl_3$, 298 K)-- S25
Figure S22 – Two-dimensional (^{1}H-^{13}C) HMBC NMR spectrum of rotaxane **19**---------- S26
Figure S23 – Low-resolution MALDI-TOF mass spectrum of rotaxane **19**---------------- S27
Syntheses-- S28
Spectral Data-- S49
Crystal Data--- S97
References-- S104

1. General Information

1.1. Materials

All chemicals were purchased from Sigma-Aldrich and Labsynth and used without further purification unless otherwise noted. For moisture-sensitive reactions, solvents were freshly distilled. Dichloromethane (DCM), benzene and toluene (MePh) were dried over calcium hydride, whereas 1,4-dioxane and tetrahydrofuran (THF) were dried using the sodium/benzophenone system. Chloroform was dried over calcium chloride. Anhydrous dimethylformamide (DMF) was used as received. Acetone, 1,2,4-trichlorobenzene, dimethyl sulfoxide (DMSO), petroleum ether, methanol (MeOH) and ethanol (EtOH) were P.A. ACS grade reagents and were used as received. Technical grade ethyl acetate (EtOAc) and hexanes were used as received in extractions and as eluent in column chromatography purifications. Epoxidized soybean oil was prepared from commercially available refined soybean oil and performic acid [generated *in situ* from aqueous hydrogen peroxide (50% w/w) and formic acid (assay 85%)] as oxidizing agent.[S1] The final product had an oxirane oxygen content of about 6% (w/w) as determined according to the American Oil Chemical Society Official Method Cd-9-57. All syntheses were carried out using Schlenk line techniques. Moisture-sensitive liquids were transferred by cannula or syringe. The progress of the reactions was monitored by thin-layer chromatography (TLC) whenever possible. TLC was performed using pre-coated aluminum plates (SiliaPlate from Silicycle with 200 μm thickness) containing a 254 nm fluorescent indicator. Preparative TLC was carried out on glass plates coated with silica gel 60 PF_{254} containing gypsum ($CaSO_4$•0.5 H_2O, 28-32%, w/w) with particle size 90% ≤ 55 μm from Merck EMD Millipore Corporation. Column chromatography was carried out using Fluka Silica Gel 60 (230-400 mesh particle size). Melting points were determined using a PF1500 Farma Capillary Melting Point apparatus from Gehaka and were uncorrected. Diphenyldiazomethane was prepared according to literature methods.[S2]

1.2. Nuclear Magnetic Resonance – NMR

^{1}H, ^{13}C, ^{31}P, COSY, HMBC and HSQC NMR spectra were obtained on either a Bruker AVANCE 250 (250 MHz), a Bruker AVANCE 400 (400 MHz) or a Bruker AVANCE 500 (500 MHz), in all cases using deuterated solvents as the lock. The spectra were collected at 298 K or 333 K, and chemical shifts reported in parts per million (δ, ppm) were referenced to residual solvent peak. In the assignments, the chemical shift (in ppm) is given first, followed, in parentheses, by multiplicity (s, singlet; d, doublet; t, triplet; q,

quartet and quintet; m, multiplet; br, broad), the values of the *J*-coupling constants in Hz (if applicable), the number of protons implied and finally the assignment. Residual solvent peaks and eventual aliphatic impurities were assigned according to literature.[S3] Two-dimensional NOESY NMR spectra were acquired on a Bruker AVANCE 400 (400 MHz) using $CDCl_3$ as deuterated solvent at 298 K and 400 ms mixing time.

1.3. Mass Spectrometry

Low resolution mass spectra (LRMS) were afforded from an Agilent GC-MS piece of equipment, model 5975C, equipped with a monoquadrupole detector in positive mode and electron impact (EI) source. High-resolution mass spectra (HRMS) were recorded on a Q-TOF (ESI-QTOF) equipment operating in positive mode. Low-resolution MALDI-TOF mass spectra were recorded in a Bruker Daltonics Microflex LT MALDI-TOF MS, while the high-resolution spectra were acquired in a Bruker Daltonics Autoflex III Smartbean equipped with LIFT TOF/TOF technology spectrometers. The mass spectra represent an average over 512 consecutive laser shots in linear mode using (1E,3E)-1,4-diphenylbuta-1,3-diene (DPB) as matrix (unless otherwise noted), which was purchased from Aldrich. The mass scale was calibrated using the peptide calibration standard purchase from Bruker and α-cyano-4-hydroxycinnamic acid (HCCA) as matrix. Data processing was carried out using the software package Compass for Flex series available from Bruker. Mentioned *m/z* values correspond to monoisotopic masses.

1.4. Fourier-Transform Infrared Spectroscopy – FTIR

FTIR spectra were obtained from an Agilent Cary 630 spectrometer equipped with Attenuated Total Reflection (ATR) accessory and 4 cm^{-1} resolution. All samples were analyzed in the solid state.

1.5. Steady-State Ultraviolet-Visible Absorption Spectroscopy – UV-Vis

UV-Vis spectra were obtained from an Agilent Cary 50 spectrometer with 1.0 nm resolution. All samples were analyzed in dichloromethane solutions (10^{-5} mol/L) at room temperature in quartz cuvettes with 1 cm width.

2. Spectroscopic Data and Chemical Structures of Compounds Mentioned in the Manuscript

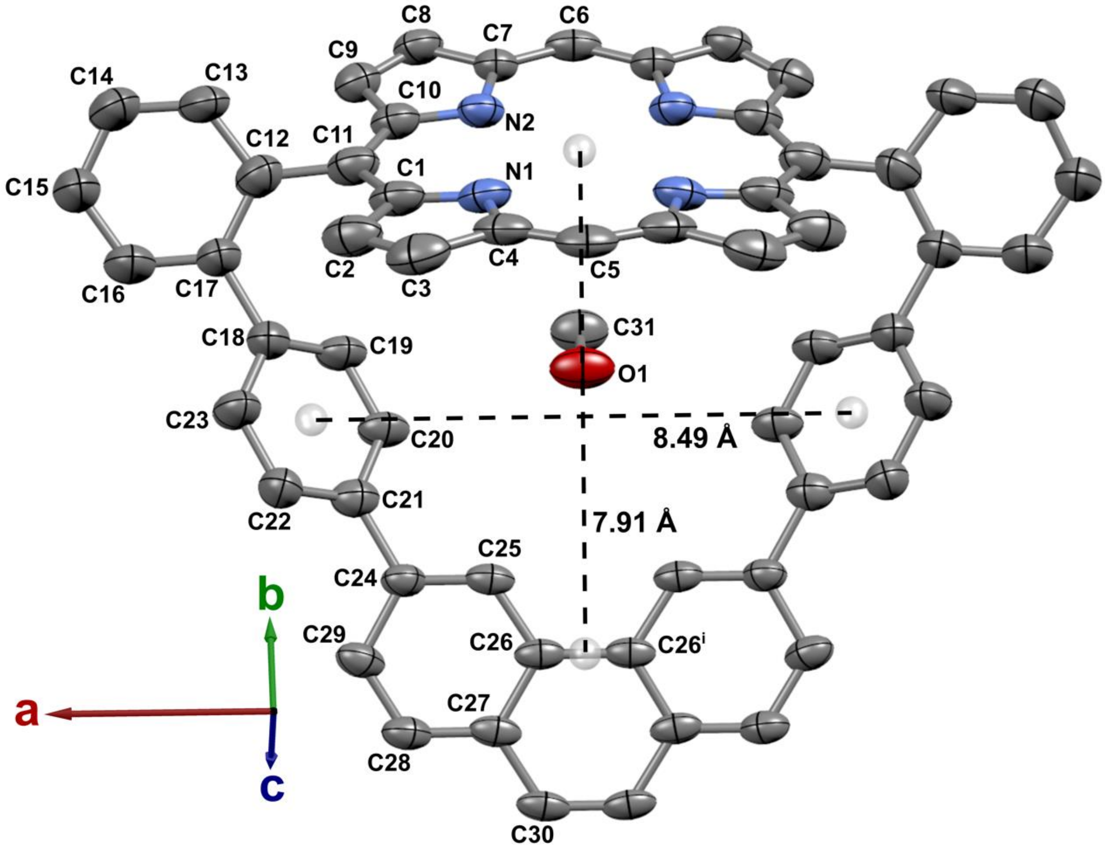

Figure S1 – Estimated cavity size afforded from the crystal structure of macrocycle 7. Using Mercury software, a centroid at the C26–C26^{i} bond on the phenanthrene moiety was calculated, which along with the centroid calculated from the 24 porphyrin atoms and the two centroids calculated from the 6 carbon atoms of the two phenyl spacers allowed the estimation of the cavity size in **7**. Carbon atoms are shown in grey, nitrogen in blue and oxygen in red. Hydrogen atoms are omitted for clarity purposes. Ellipsoids are drawn at 50% probability levels. Symmetry code: i = 1/2-x, y, z.

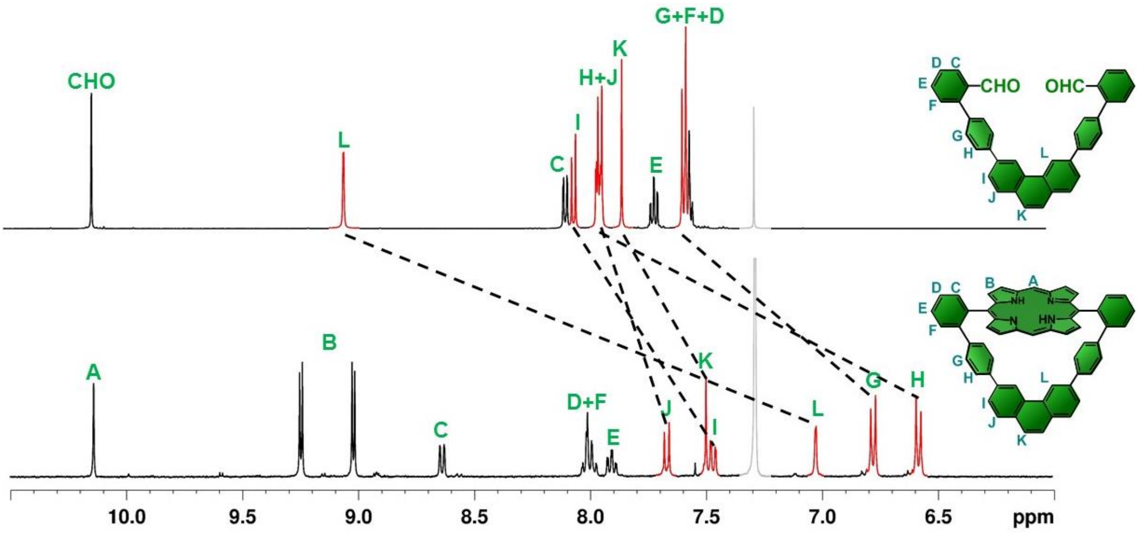

Figure S2 – Aromatic region of ^{1}H NMR spectra of compound 6 (top) and macrocycle 7 (bottom) (500 MHz, $CDCl_3$, 298 K). Proton assignments are based on 2D-NMR spectroscopy (*vide infra*). Residual solvent is in grey: chloroform (δ = 7.26 ppm). All resonances in the spectrum of macrocycle **7** are sharp and distinct and confirm the proposed hollow structure in solution. Furthermore, comparison between ^{1}H NMR spectra of **6** and **7** reveals that the phenanthrene protons facing the cavity (labelled as H_L) are unusually shielded. That significant shielding ($\Delta\delta_L$ = 2.03 ppm) informs that the H_L protons are at reach of the strong magnetic anisotropy of the porphyrin aromatic system in **7**. In fact, all protons on the aromatic loop in **7** are affected by the porphyrin ring current effects (dotted lines). The shielding of the nuclei on the aromatic loop confirms the well-defined and relatively small central cavity in **7**. Therefore, the aromatic loop provides the internal axial position of the resulting porphyrinate with effective steric shielding against upcoming bulky ligands in solution.

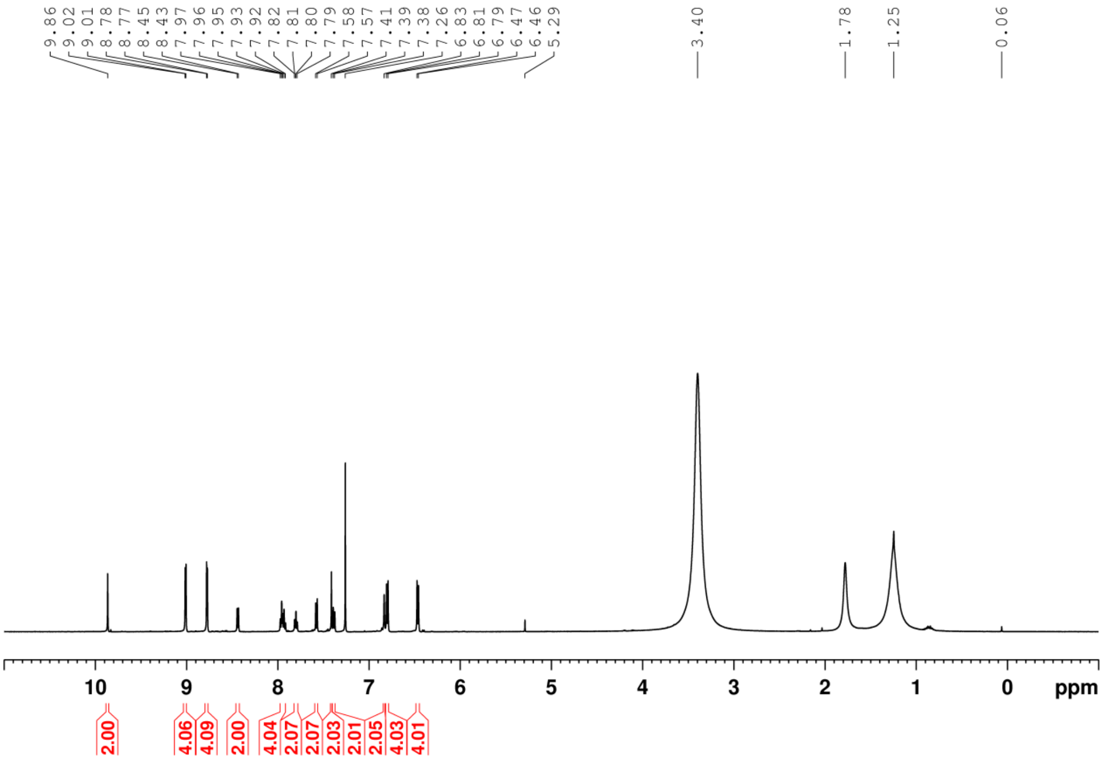

Figure S3 – ^{1}H NMR spectrum of the porphyrinate product isolated after metalation reaction of macrocycle 7 with $Ru_3(CO)_{12}$ (500 MHz, $CDCl_3$, 298 K). To improve the solubility of the complex in the $CDCl_3$ solvent, 0.5% (v/v) of regular methanol was added to the NMR sample, which appears as large singlets at δ = 3.39 ($C\underline{H}_3OH$) and δ = 1.24 ($CH_3O\underline{H}$). Residual solvent and aliphatic impurities: chloroform (δ = 7.26 ppm), dichloromethane (δ = 5.29 ppm), residual water in $CDCl_3$/CH_3OH solvent mixture (δ = 1.77 ppm). After the metalation reaction, only one porphyrin product is isolated from the crude, despite all our efforts to identify the expected isomers **8** and **9** by TLC. The spectrum of the isolated porphyrin product is not consistent with a mixture of isomeric complexes as no duplication of signals are observed. As described in the manuscript, it turned out that the isolated product was complex **8**.

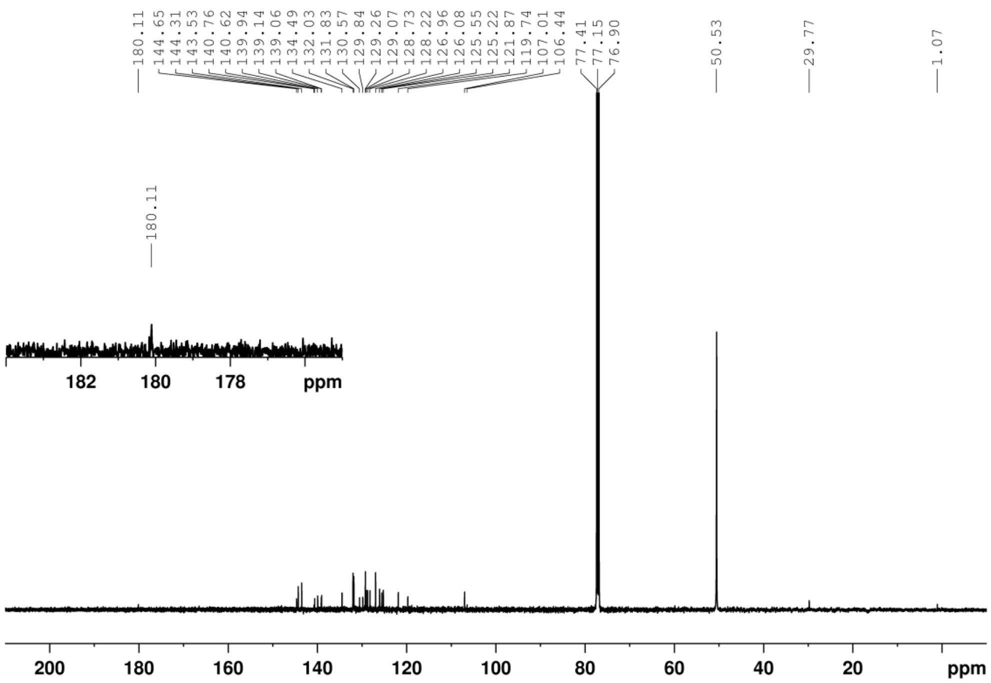

Figure S4 – ^{13}C NMR spectrum of the porphyrinate product isolated after metalation reaction of macrocycle 7 with $Ru_3(CO)_{12}$ (125 MHz, $CDCl_3$, 298 K). To improve the solubility of the complex in the $CDCl_3$ solvent, 0.5% (v/v) of regular methanol was added to the NMR sample, which appears at δ = 50.53 ($\underline{C}H_3OH$). Inset: resonance of the $\underline{C}O$ axial ligand. It turned out that this product was complex **8**.

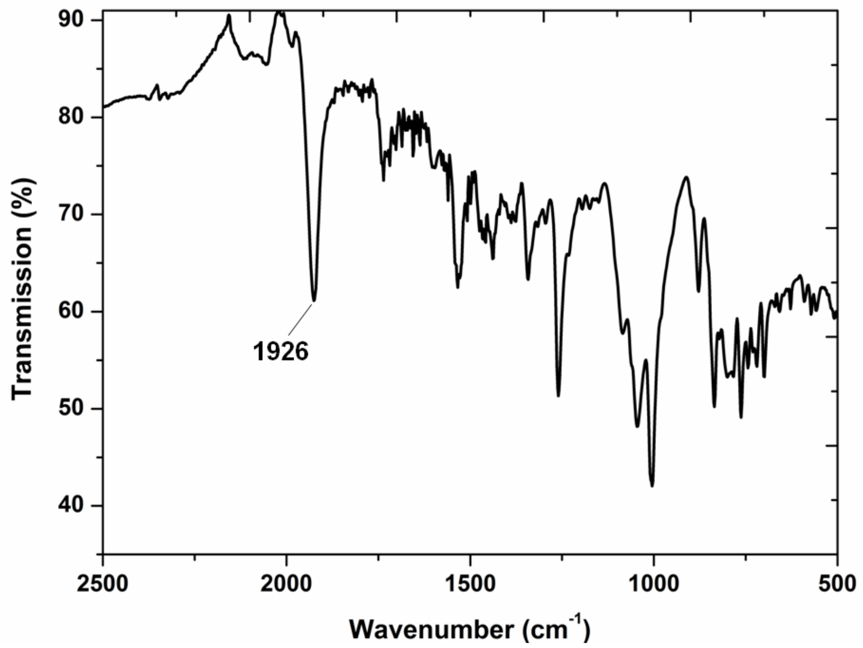

Figure S5 – Carbonyl stretching frequency region of the FTIR-ATR spectrum of the porphyrinate product isolated after metalation reaction of macrocycle 7 with $Ru_3(CO)_{12}$. It turned out that this product was complex 8.

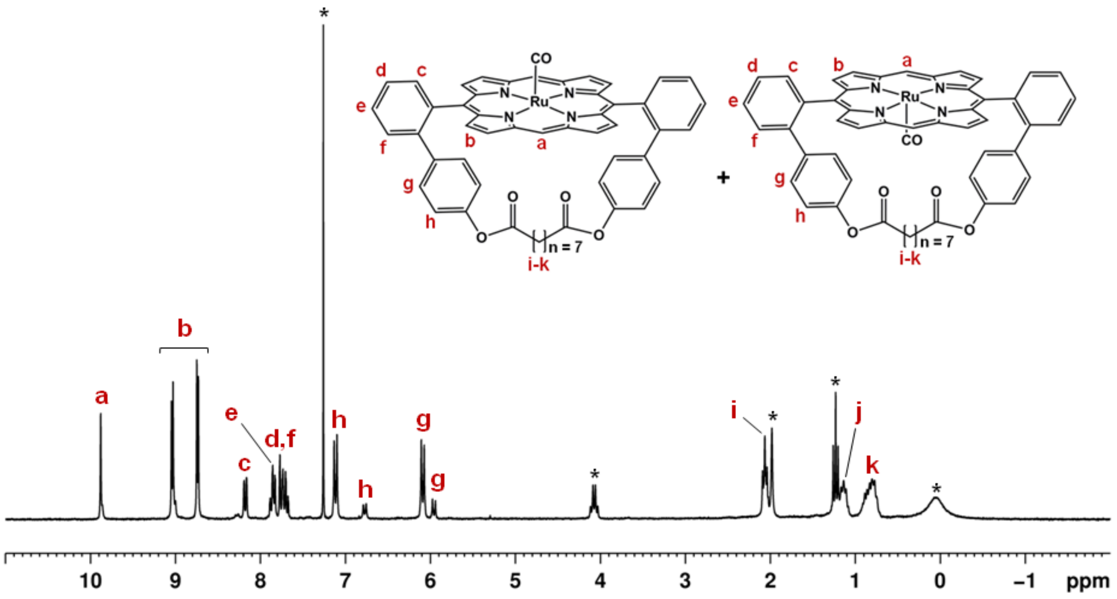

Figure S6 – ^{1}H NMR spectrum of the semi-rigid porphyrin-based macrocycle reported in our previous work[S4] after its metalation reaction with $Ru_3(CO)_{12}$ (250 MHz, $CDCl_3$, 298 K). Residual solvent and aliphatic impurities are marked with an asterisk: chloroform (δ = 7.26 ppm); ethyl acetate (δ = 4.12, 2.05 and 1.26 ppm); silicone "grease" (δ = 0.07 ppm). After workup, the crude product showed two porphyrin spots on TLC. Duplication of the resonances for protons H_g and H_h reveals formation of the isomeric mixture of Ru(II)porphyrinates with the carbonyl axial ligand coordinated *exo*- and *endo*-to the macrocycle's cavity. It should be noted that, contrary to the shielding observed for all protons on the appended aromatic loop in the ^{1}H NMR spectrum of **7** (Figure S2), the protons on the aliphatic linker of the semi-rigid macrocycle appear in the expected region of the spectrum. Accordingly, those aliphatic nuclei do not fell the porphyrin magnetic anisotropy and confirms that macrocycle **7** has a significant smaller central cavity than that of the semi-rigid macrocycle in solution.

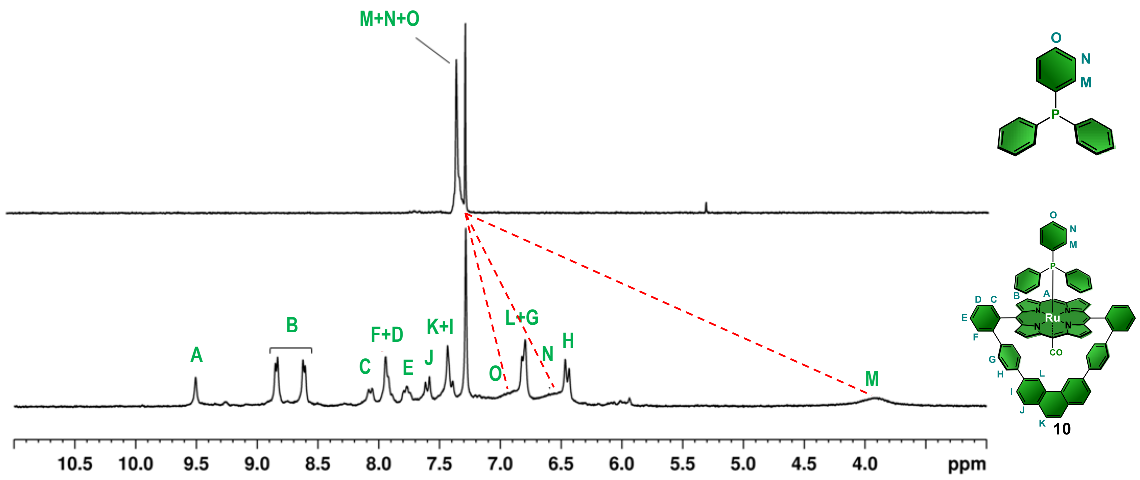

Figure S7 – Selected region of the ^{1}H NMR spectra of pristine triphenylphosphine (top) and complex 10 (bottom) (250 MHz, $CDCl_3$, 298 K). The shielding of the $H_{M\text{-}O}$ protons on the triphenylphosphine axial ligand (red dashed-lines) due to the porphyrin ring current effects confirms the quantitative formation of complex **10**. The PPh_3 proton resonances are broad, suggesting rapid exchange between free and coordinated PPh_3 species at a rate comparable to the NMR time scale at 298 K.

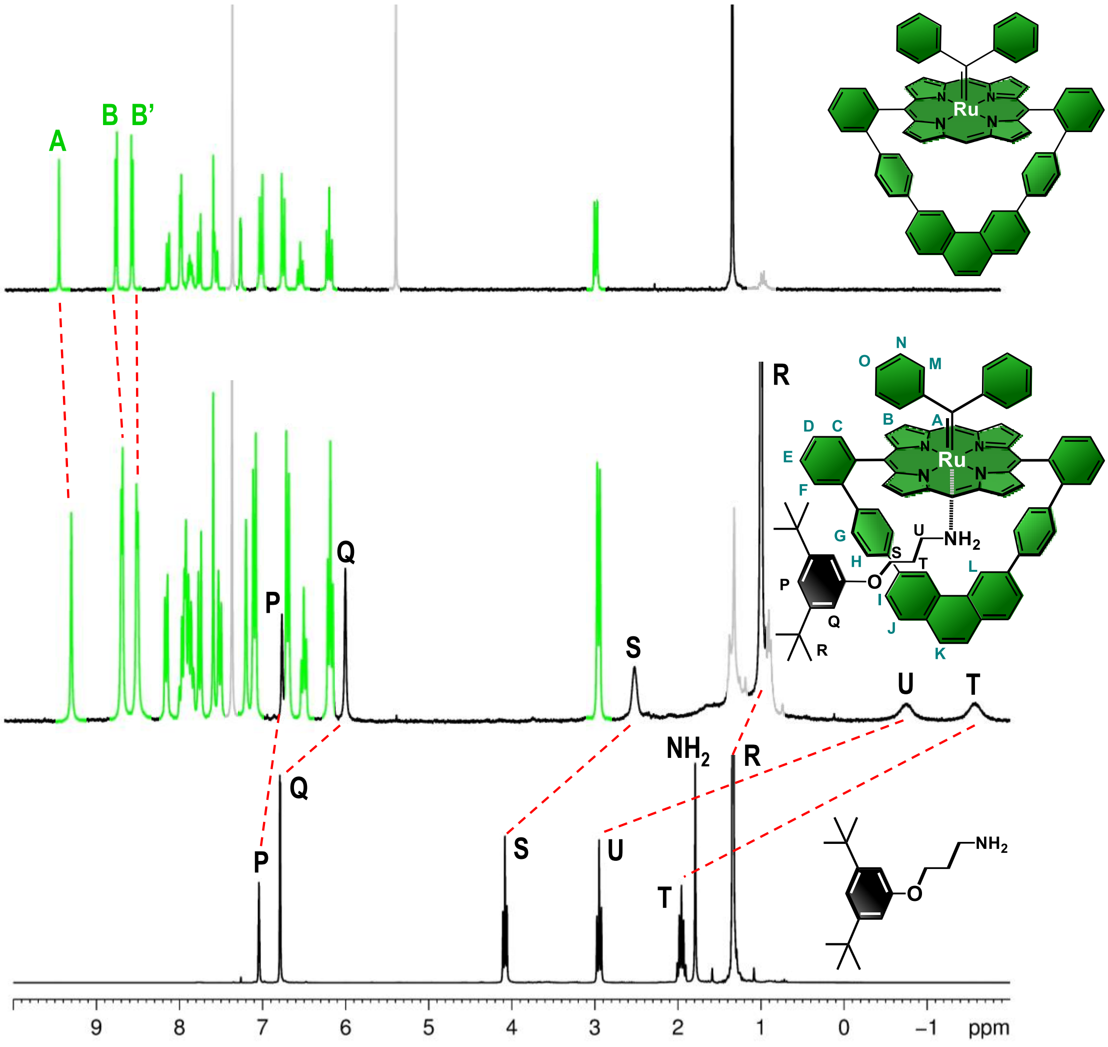

Figure S8 – Comparison between the ^{1}H NMR spectra (250 MHz, $CDCl_3$, 298 K) of macrocycle 11 (top), complex 12 (middle), which is afforded by just mixing an equimolar amount of macrocycle 11 and half-thread 13 in $CDCl_3$, and pristine half-thread 13 (bottom). Shielding of the assigned protons (dashed red lines) confirms coordination of the amine group on **13** to the Ru(II) ion in macrocycle **11** at 298 K. Residual solvent and aliphatic impurities are in grey: chloroform (δ = 7.26 ppm), dichloromethane (δ = 5.29 ppm), "grease" (δ = 1.26 and 0.88 ppm). Such comparative analysis reveals the diagnostic slightly shielding of the *meso*- and pyrrolic protons (H_A and H_B, respectively) of the porphyrin core when compared to those in pristine **11**. Such shielding is due to donation of electronic density from the amino group in **13** to the Ru(II) ion in **11**, thereby confirming formation of **12**. Furthermore, all proton resonances on the axial half-thread ligand are shielded in **12**, particularly the methylene $H_{S\text{-}U}$ nuclei, when compared to the respective resonances in the spectrum of pristine **13**. The strong shielding of the methylene protons, which is due

to the porphyrin magnetic anisotropy, informs that those groups are held close to the Ru(II)porphyrinate core in **12**, and confirms the axial coordination of the amino group to the Ru(II) ion. However, the Ru–N<u>H</u>$_2$ resonance is not observed in the ^{1}H NMR spectrum of **12** in $CDCl_3$. This lack of the Ru–N<u>H</u>$_2$ signal is due to rapid hydrogen-deuterium exchange reactions between the amino group and $CDCl_3$ solvent molecules. Accordingly, the expected broad and strongly shielded Ru–N<u>H</u>$_2$ signal is clearly observed in the spectrum of **12** in C_6D_6 at 298 K as shown below in Figure S8.1.

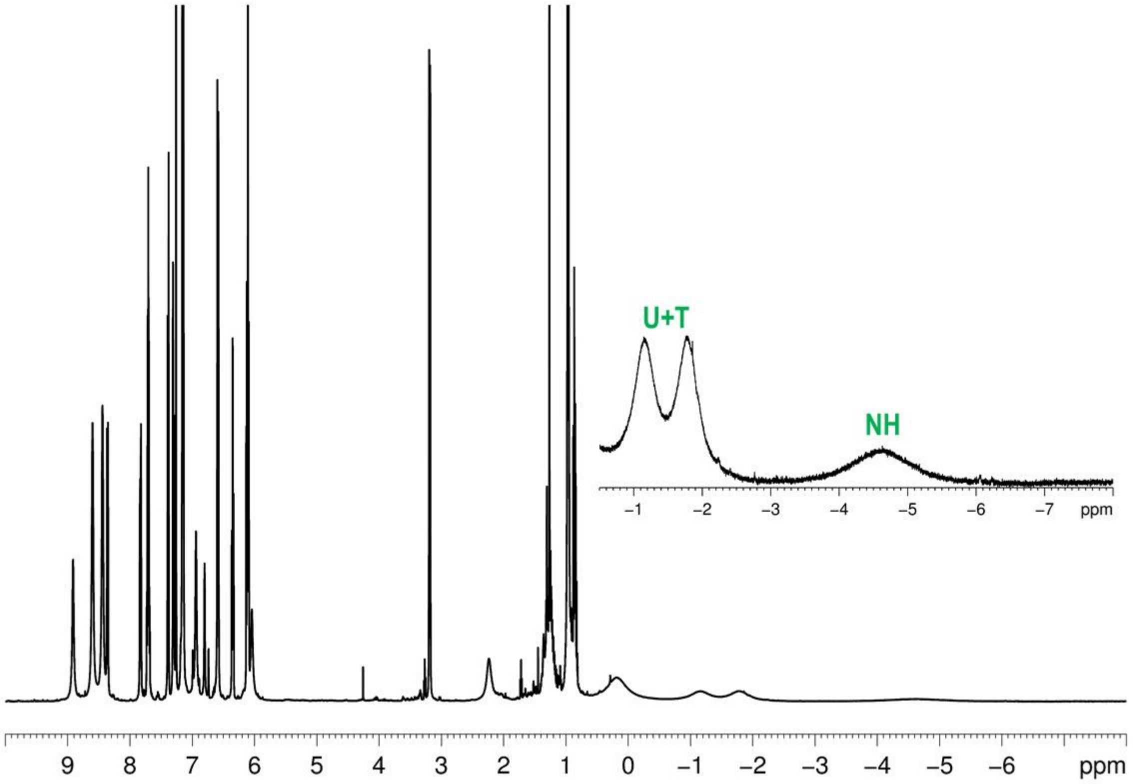

Figure S8.1 – ^{1}H NMR spectrum of complex 12 in C_6D_6 at 298 K (500 MHz). The inset shows the selected region of the spectrum with magnified intensity in which the Ru–N<u>H</u>$_2$ resonance appears as a broad and strongly shielded signal. Therefore, the ^{1}H NMR spectrum in C_6D_6 provides strong evidence for the hydrogen-deuterium exchange reactions between the amino group and $CDCl_3$ solvent molecules and the consequent absence of the NH_2 signal in the spectrum of **12** in that solvent (Figure S8 above).

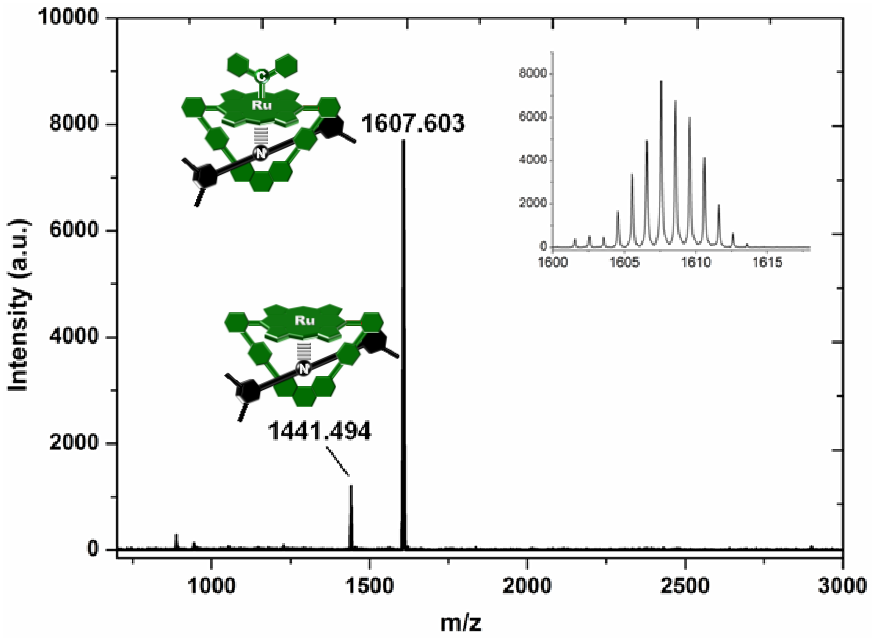

Figure S9 – High-resolution MALDI-TOF mass spectrum of rotaxane 15. *Inset*: isotopic distribution for $C_{106}H_{99}N_5O_4Ru$. The molecular ion peak for **15** is observed at *m/z* 1607.603 $[M]^+$ (*m/z* 1607.674 calculated for $C_{106}H_{99}N_5O_4Ru$), along with a molecular ion peak at *m/z* 1441.494 [M – diphenylcarbene ligand]$^+$, which correspond to a rotaxane species with no diphenylcarbene axial ligand. The latter signal informs rupture of some Ph_2C–Ru coordinative bond in **15** during the MALDI-TOF ionization process. Such rupture of the Ph_2C–Ru bond upon irradiation is common in carbenoid complexes as the coordinative bond is light-sensitive.[S5]

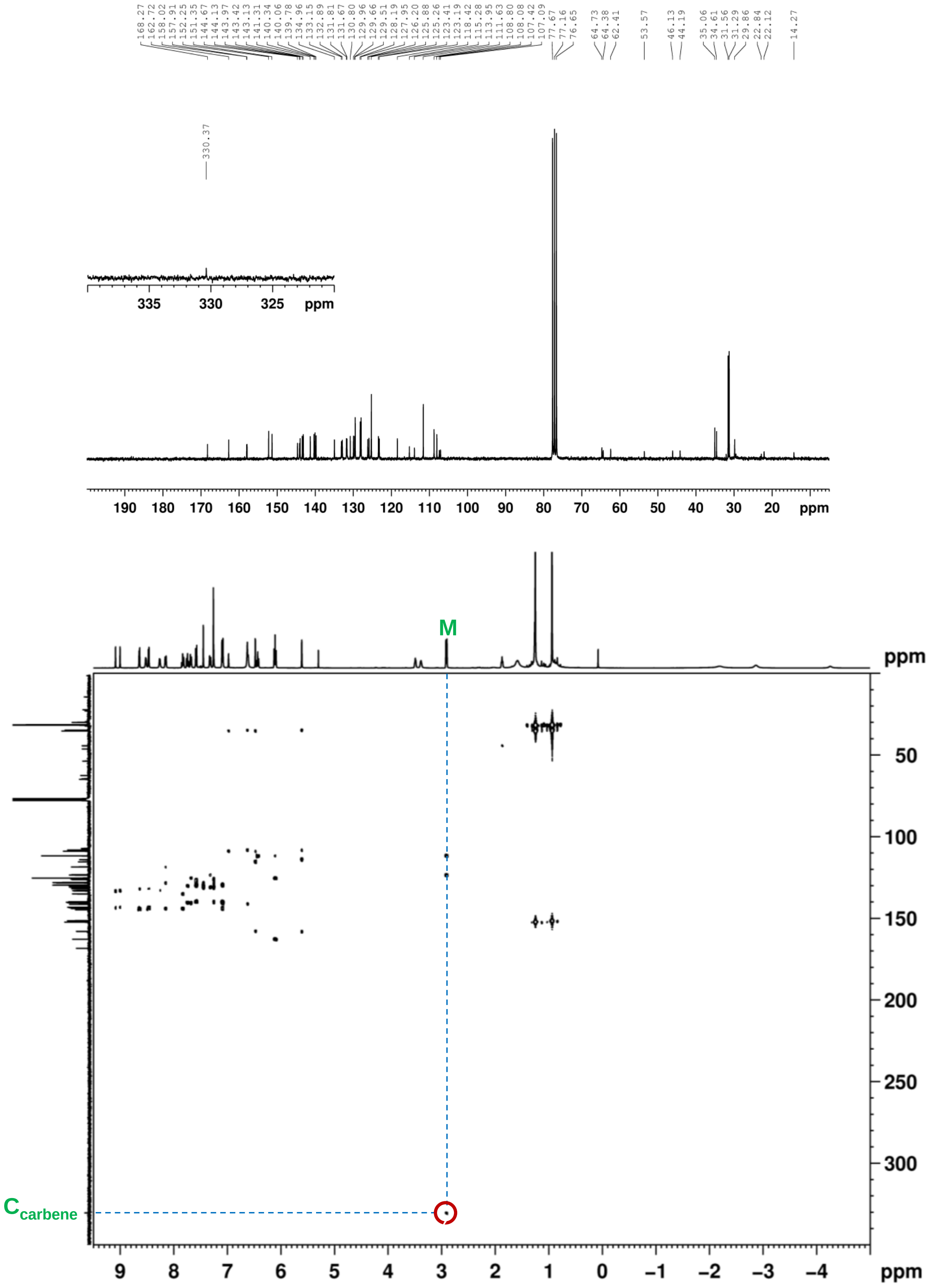

Figure S10 – Top: ^{13}C NMR spectrum of rotaxane **15** (125 MHz, $CDCl_3$, 298 K) with the inset showing the highly de-shielded carbene resonance (δ = 330.4 ppm) region. Bottom: two-dimensional (^{1}H-^{13}C) HMBC NMR spectrum of rotaxane **15** showing the correlation between the H_M and the carbene nuclei resonances, which confirms the axial coordination of diphenyl carbenoid in the rotaxane structure.

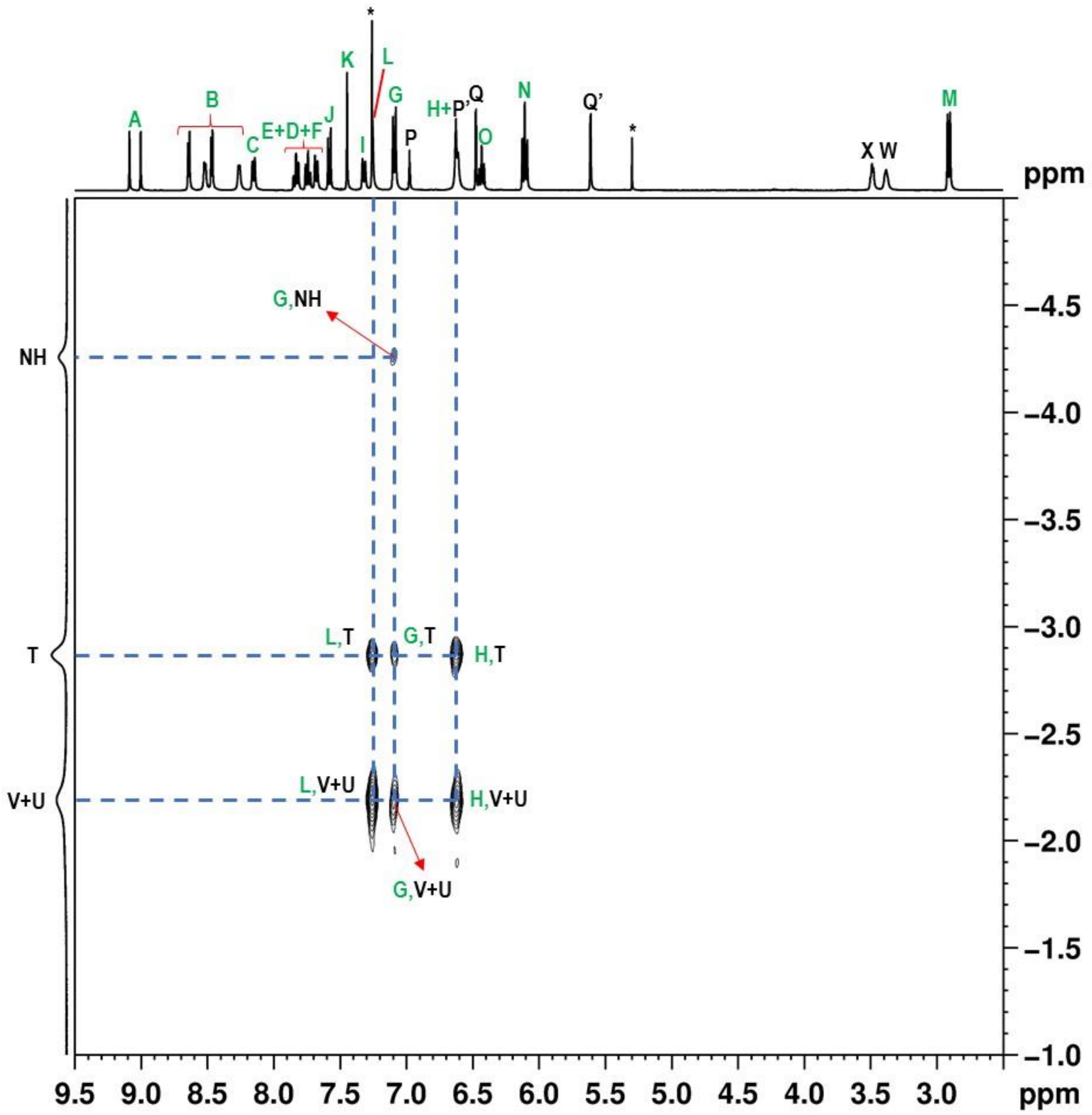

Figure S11 – Selected region of the two-dimensional (^1H-^1H) NOESY NMR spectrum of rotaxane 15 recorded in $CDCl_3$ at 298 K (400 MHz). Proton labelling corresponds to that shown in Figure 5 of the manuscript. The dashed lines designate the spatial cross signals between protons on the thread component (labelled in black) and nuclei on the macrocycle (labelled in green), which confirm the interlocked structure proposed for rotaxane **15**.

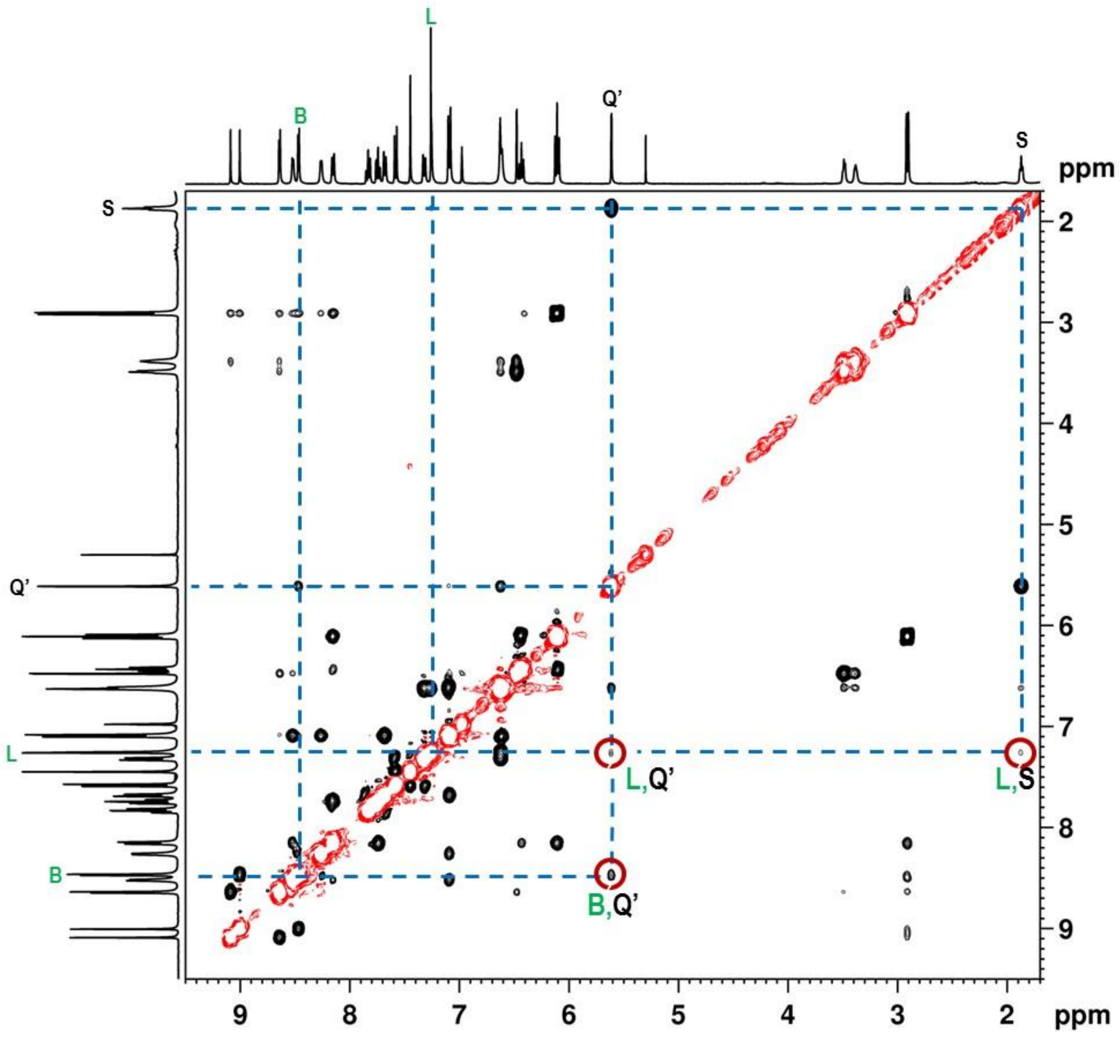

Figure S11.1 – Selected region of the two-dimensional (^1H-^1H) NOESY NMR spectrum of rotaxane 15 recorded in $CDCl_3$ at 298 K (400 MHz). Proton labelling corresponds to that shown in Figure 5 of the manuscript. The dashed lines designate the spatial cross signals between protons on the thread component (labelled in black) and nuclei on the macrocycle (labelled in green), which confirm the interlocked structure proposed for rotaxane **15**.

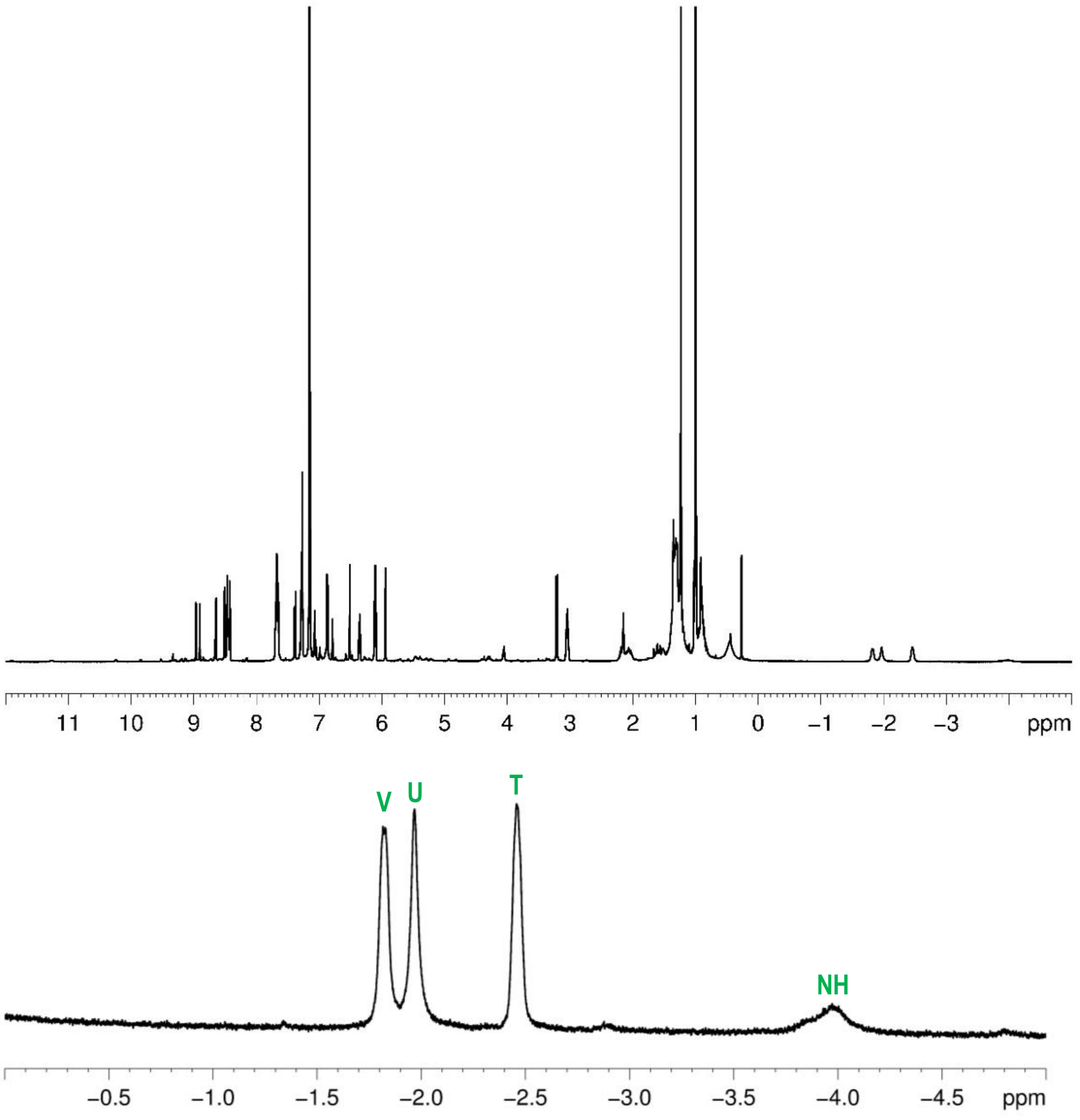

Figure S12 – ^{1}H NMR (500 MHz, C_6D_6) spectra of rotaxane 15 at 333 K. Top: full spectrum. Bottom: selected region of the ^{1}H NMR spectrum highlighting that the $R_1R_2\underline{H}N$–Ru coordinative bond in **15** is still observed at 333 K. The shielding of the N–H, H_T, H_U and H_V resonances due to the porphyrin ring current effect confirms that the ring spends most of its time over that region of the thread component even at 333 K. Accordingly, rotaxane **15** adopts the single co-conformation illustrated in Figure 6A of the manuscript in C_6D_6 at 333 K. Therefore, as described in the manuscript, the steric shielding and kinetic stabilization of the R_1R_2HN–Ru coordinative bond, which are warranted by the short molecular link between the stoppers and by the mechanical bond, respectively, act in conjunction to prevent the Ru(II) ions to promote deleterious intercomponent double insertions and/or dimerization side-reactions upon further reactions between rotaxane **15** and half-thread **14**.

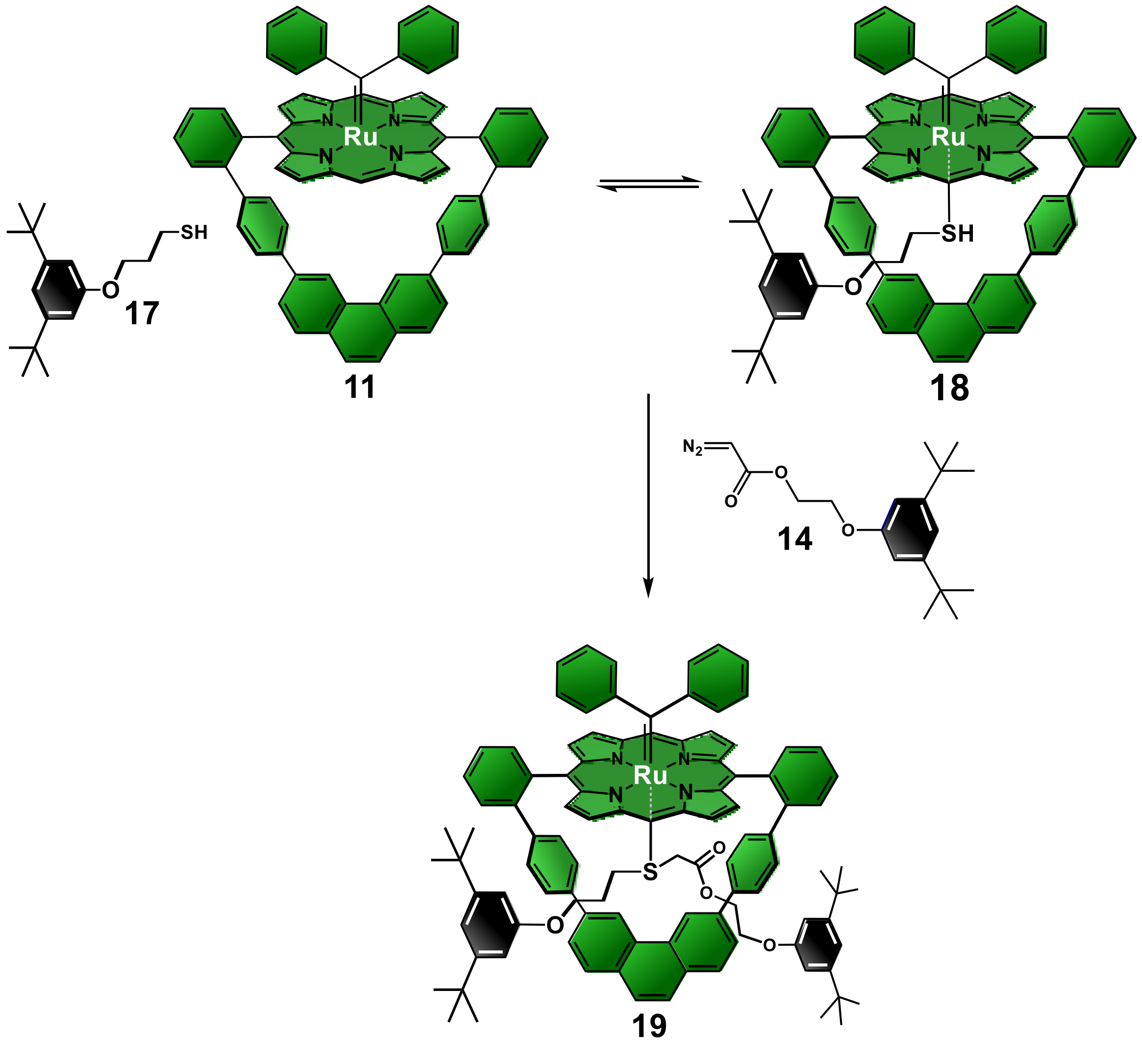

Figure S13 – Synthesis of asymmetrical [2]rotaxanes 19 by the active-metal template technique based on the Ru(II)porphyrinate-promoted S-H carbene insertion reactions. Experimental conditions: benzene, 4 h, N_2 atmosphere, room temperature, quantitative yield relative to **11**.

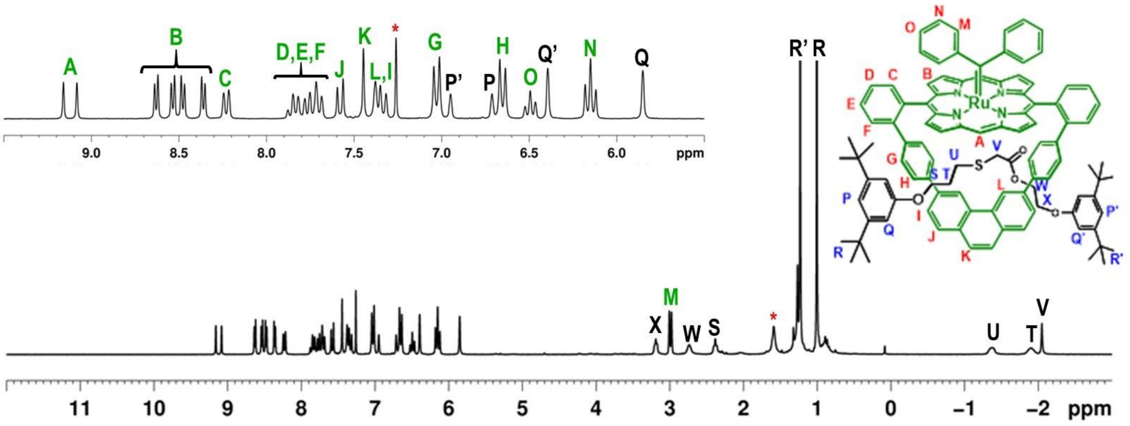

Figure S14 – ^{1}H NMR spectrum of rotaxane 19 (250 MHz, $CDCl_3$, 298 K). Red asterisk denotates residual C<u>H</u>Cl_3 (δ = 7.26 ppm) and water (δ = 1.58 ppm) in the deuterated solvent.

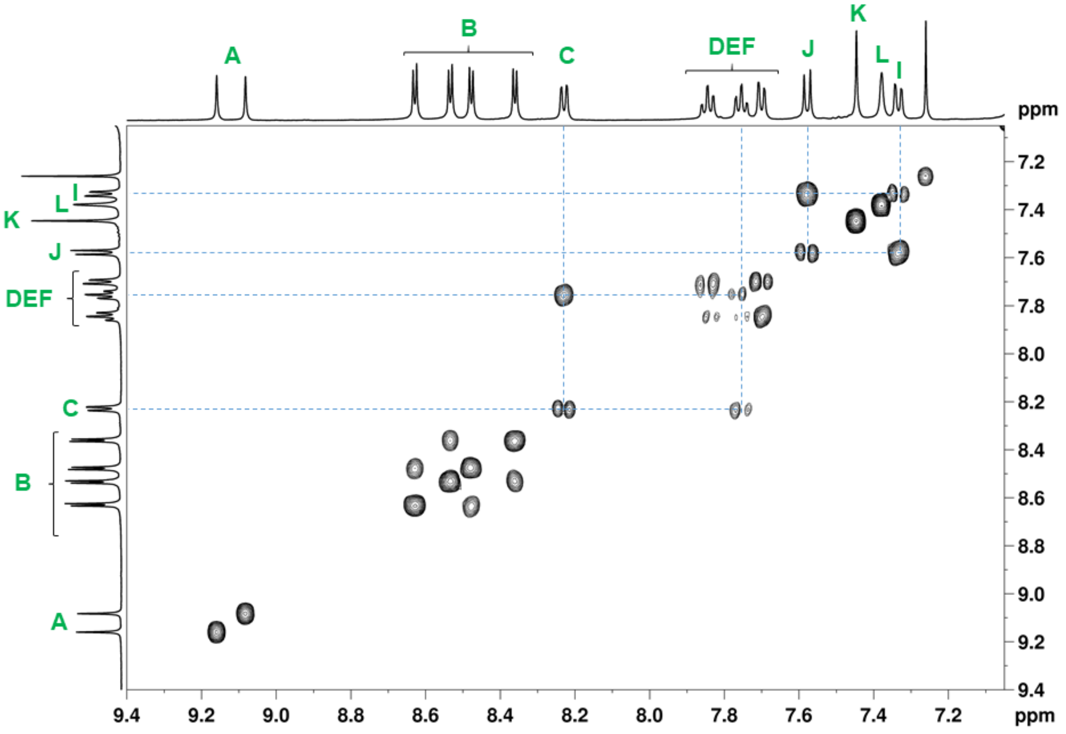

Figure S15 – Selected region of the two-dimensional (^{1}H-^{1}H) COSY NMR spectrum of rotaxane 19 (500 MHz, $CDCl_3$, 298 K). Proton labelling corresponds to that shown in Figure S14.

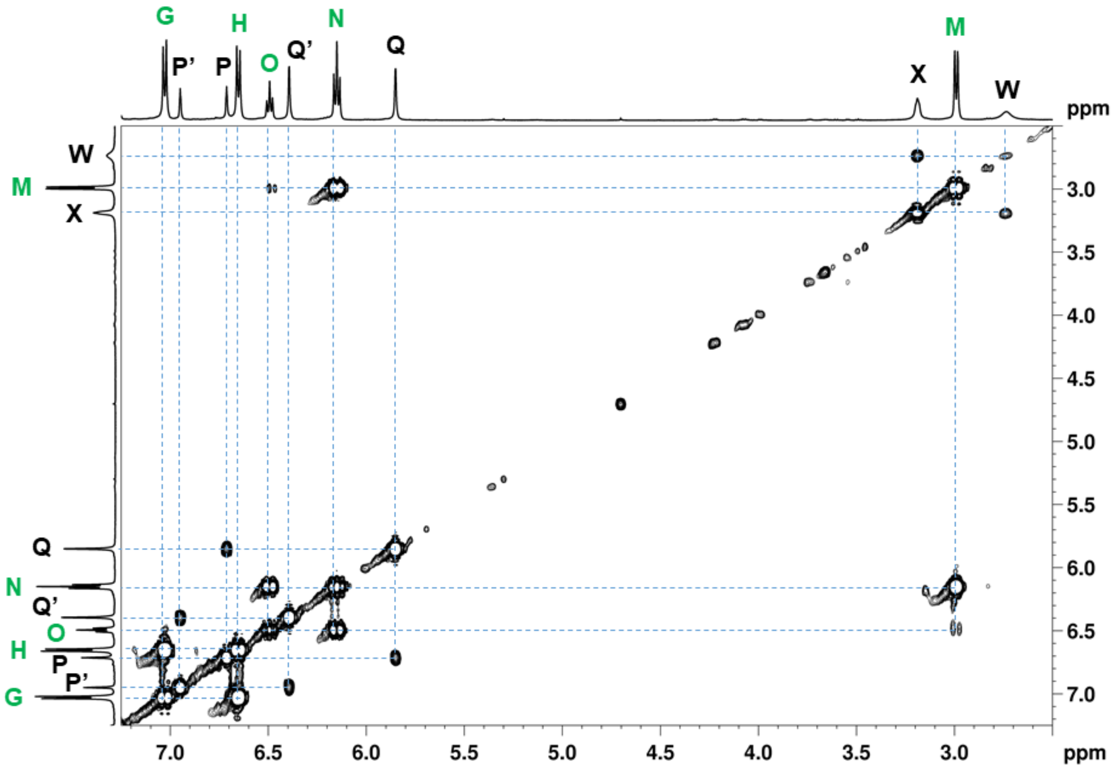

Figure S16 – Selected region of the two-dimensional (^1H-^1H) COSY NMR spectrum of rotaxane 19 (500 MHz, $CDCl_3$, 298 K). Proton labelling corresponds to that shown in Figure S14.

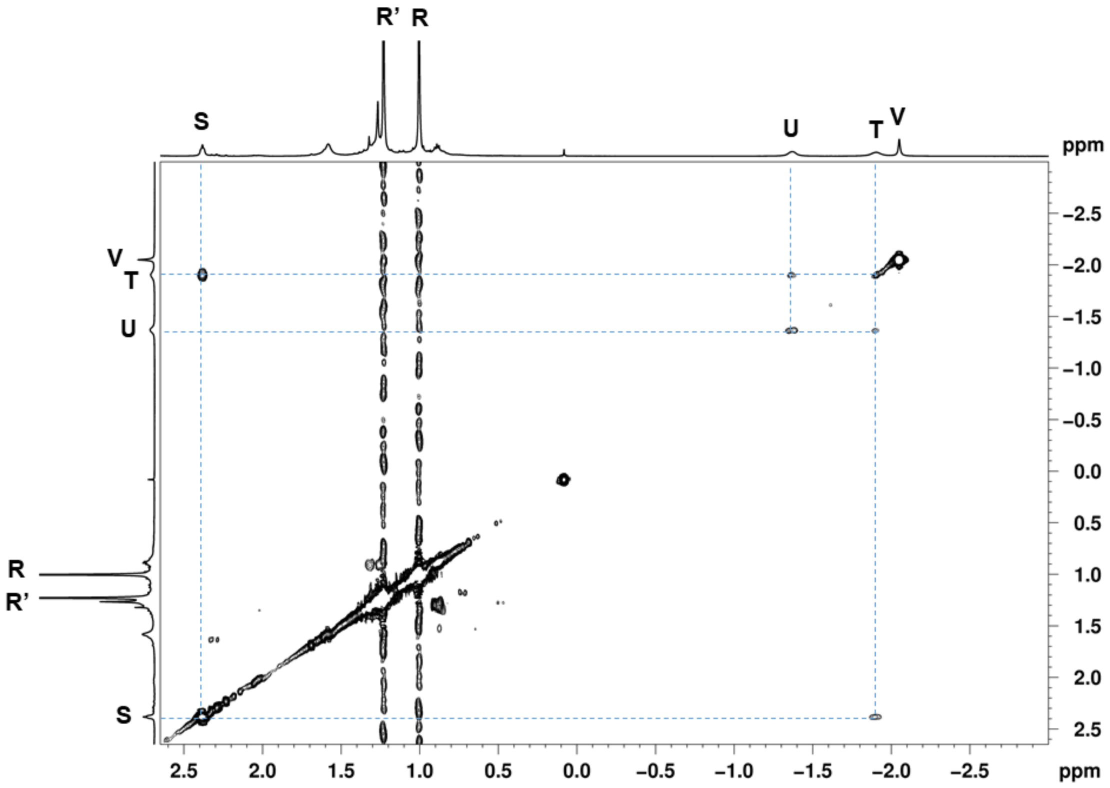

Figure S17 – Selected region of the two-dimensional (^1H-^1H) COSY NMR spectrum of rotaxane 19 (500 MHz, $CDCl_3$, 298 K). Proton labelling corresponds to that shown in Figure S14.

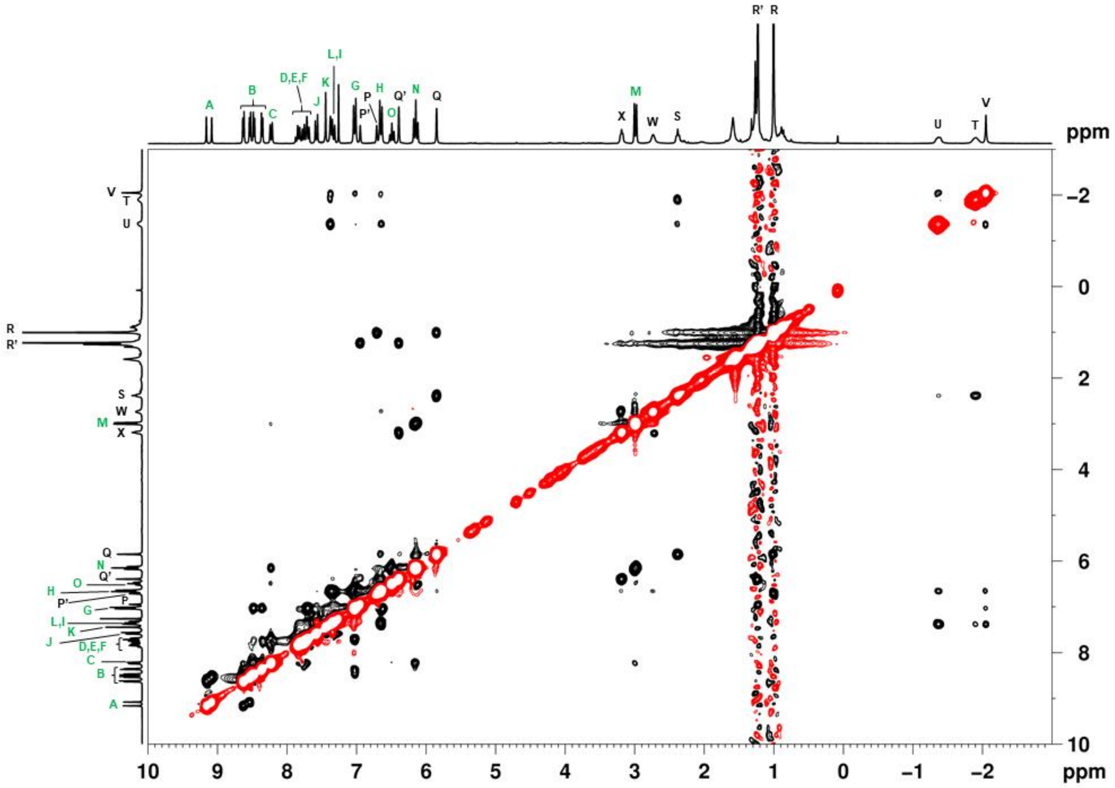

Figure S18 – Two-dimensional (^{1}H-^{1}H) NOESY NMR spectrum of rotaxane 19 (250 MHz, $CDCl_3$, 298 K). Proton labelling corresponds to that shown in Figure S14.

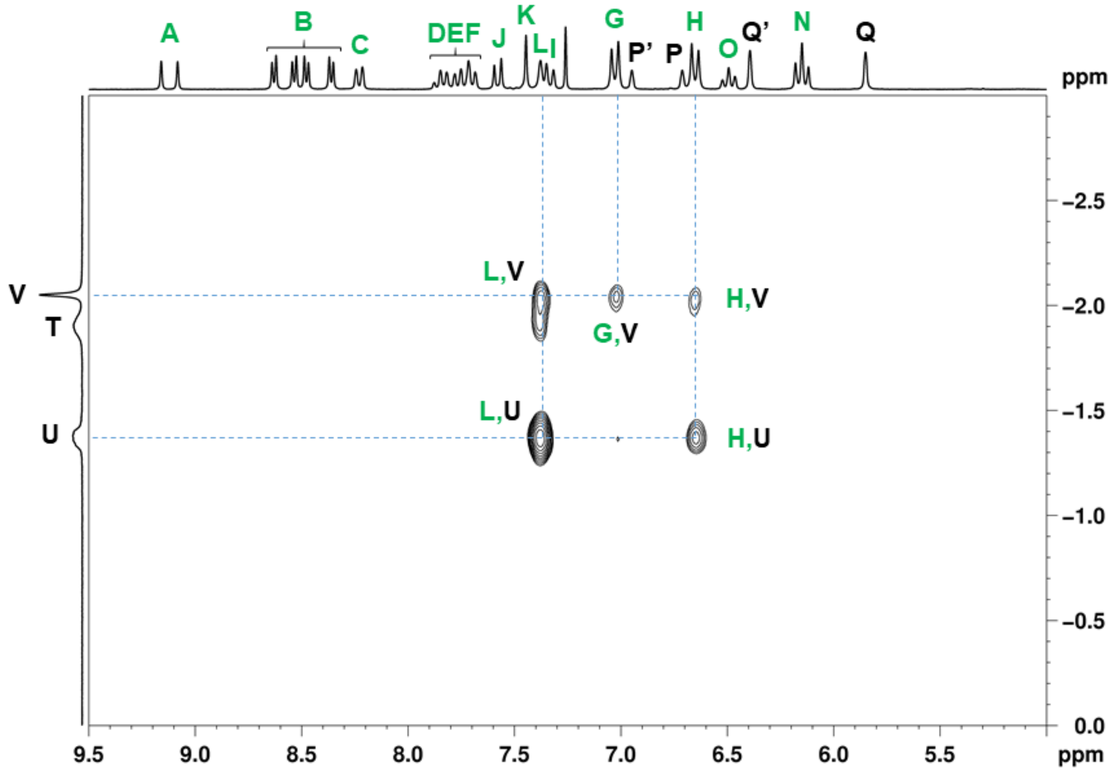

Figure S19 – Selected region of the two-dimensional (^{1}H-^{1}H) NOESY NMR spectrum of rotaxane 19 (250 MHz, $CDCl_3$, 298 K).

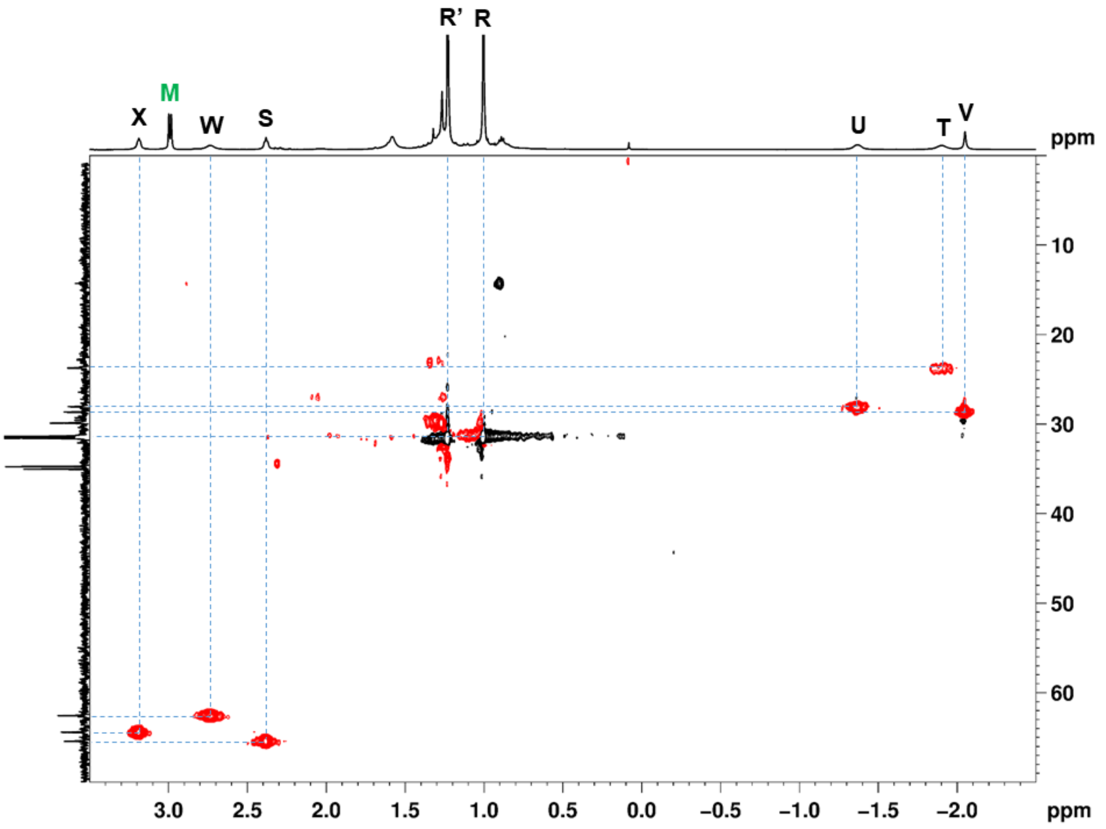

Figure S20 – Selected region of the two-dimensional (^{1}H-^{13}C) HSQC NMR spectrum of rotaxane 19 (500 MHz, $CDCl_3$, 298 K). The CH_3 and CH groups are in black, while the CH_2 moieties are in red. Proton labelling corresponds to that shown in Figure S14.

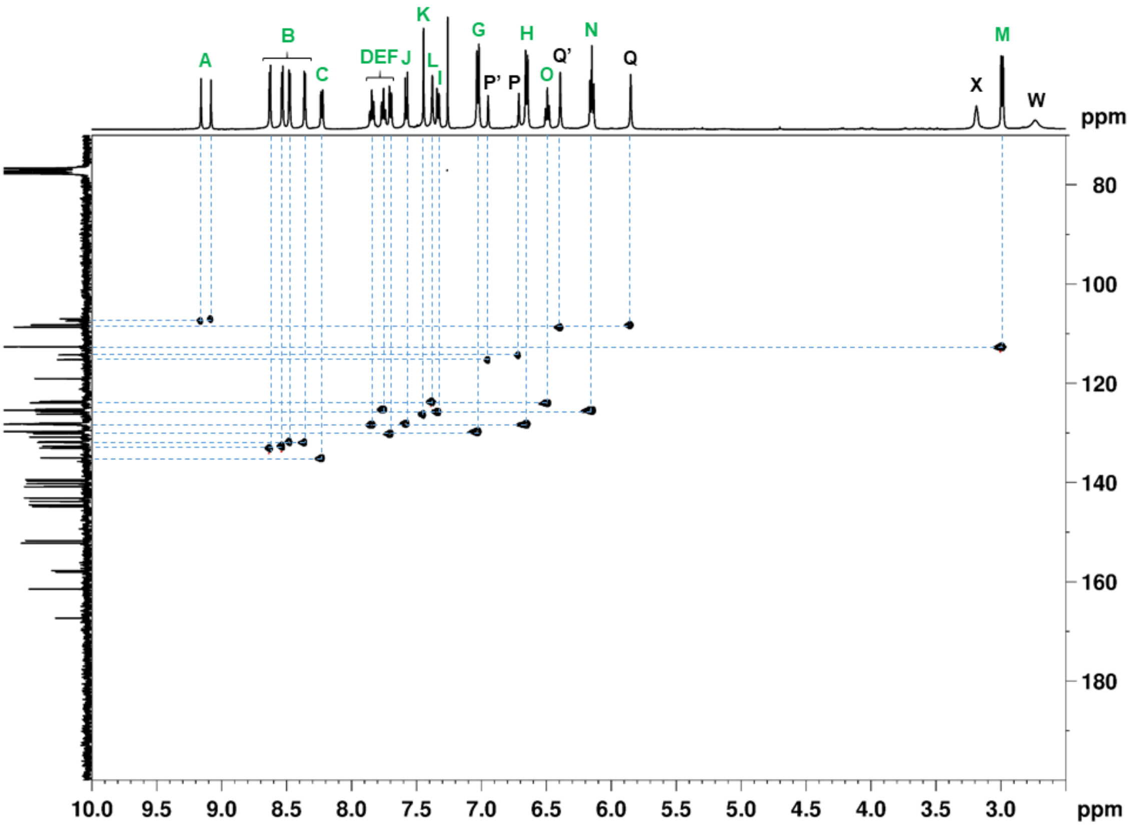

Figure S21 – Selected region of the two-dimensional (^1H-^{13}C) HSQC NMR spectrum of rotaxane 19 (500 MHz, $CDCl_3$, 298 K). The CH_3 and CH groups are in black, while the CH_2 moieties are in red. Proton labelling corresponds to that shown in Figure S14.

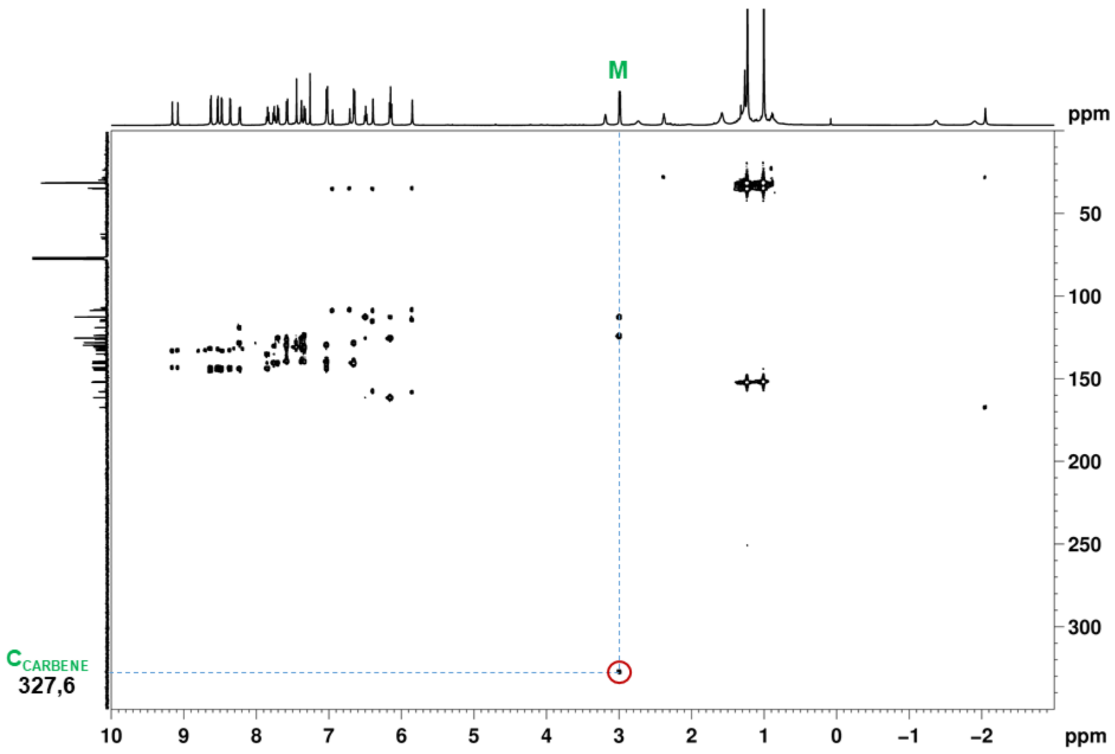

Figure S22 – Two-dimensional (^{1}H-^{13}C) HMBC NMR spectrum of rotaxane 19 showing the correlation between the H_M and the carbene nuclei resonances.

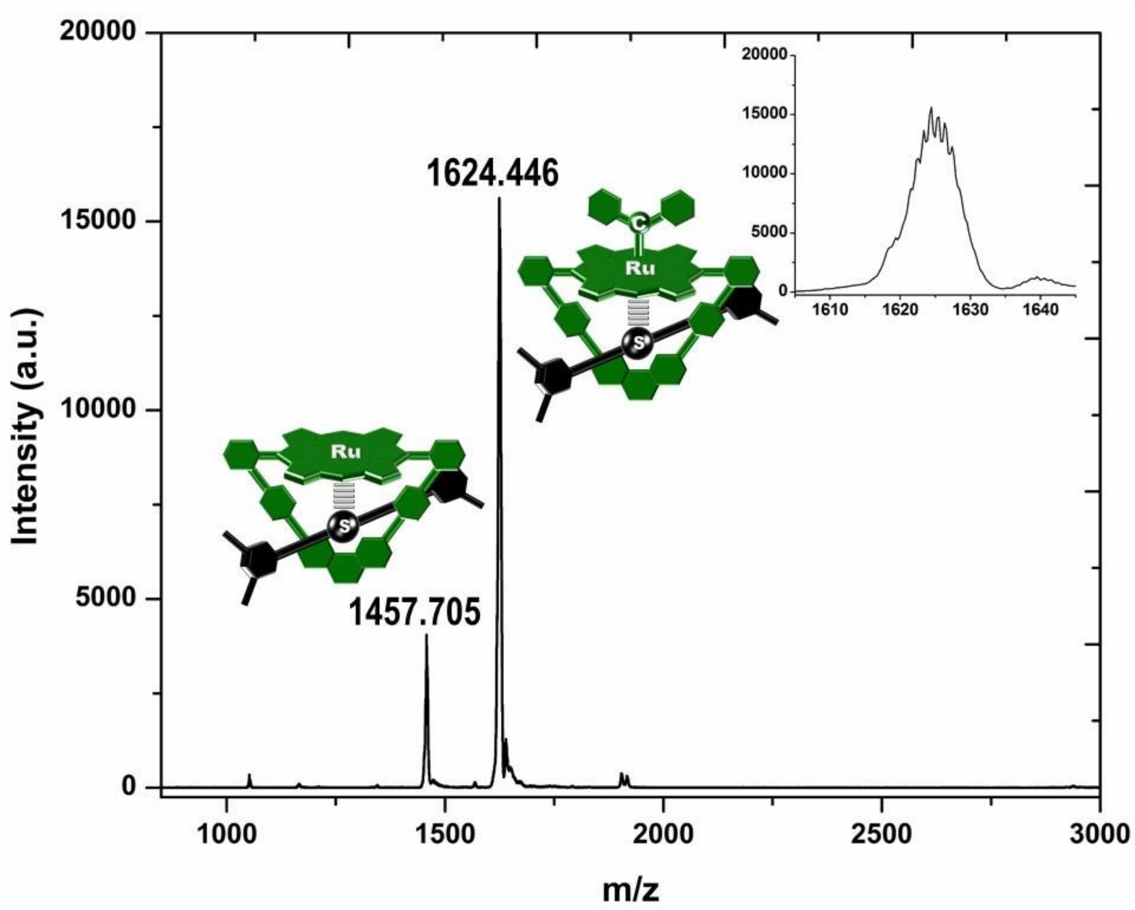

Figure S23 – Low-resolution MALDI-TOF mass spectrum of rotaxane 19. *Inset*: isotopic distribution for $C_{106}H_{98}N_4O_4SRu$. The molecular ion peak for **19** is observed at *m/z* 1624.446 $[M]^+$ (*m/z* 1624.635 calculated for $C_{106}H_{98}N_4O_4SRu$), along with a molecular ion peak at *m/z* 1457.705 [M – diphenylcarbene ligand]$^+$, which correspond to a rotaxane species with no diphenylcarbene axial ligand. The latter signal informs rupture of some Ph_2C–Ru coordinative bonds in **19** during the MALDI-TOF ionization process. Such rupture of the Ph_2C–Ru bond upon irradiation is common in carbenoid complexes as the coordinative bond is light-sensitive.[S5]

3. Synthesis

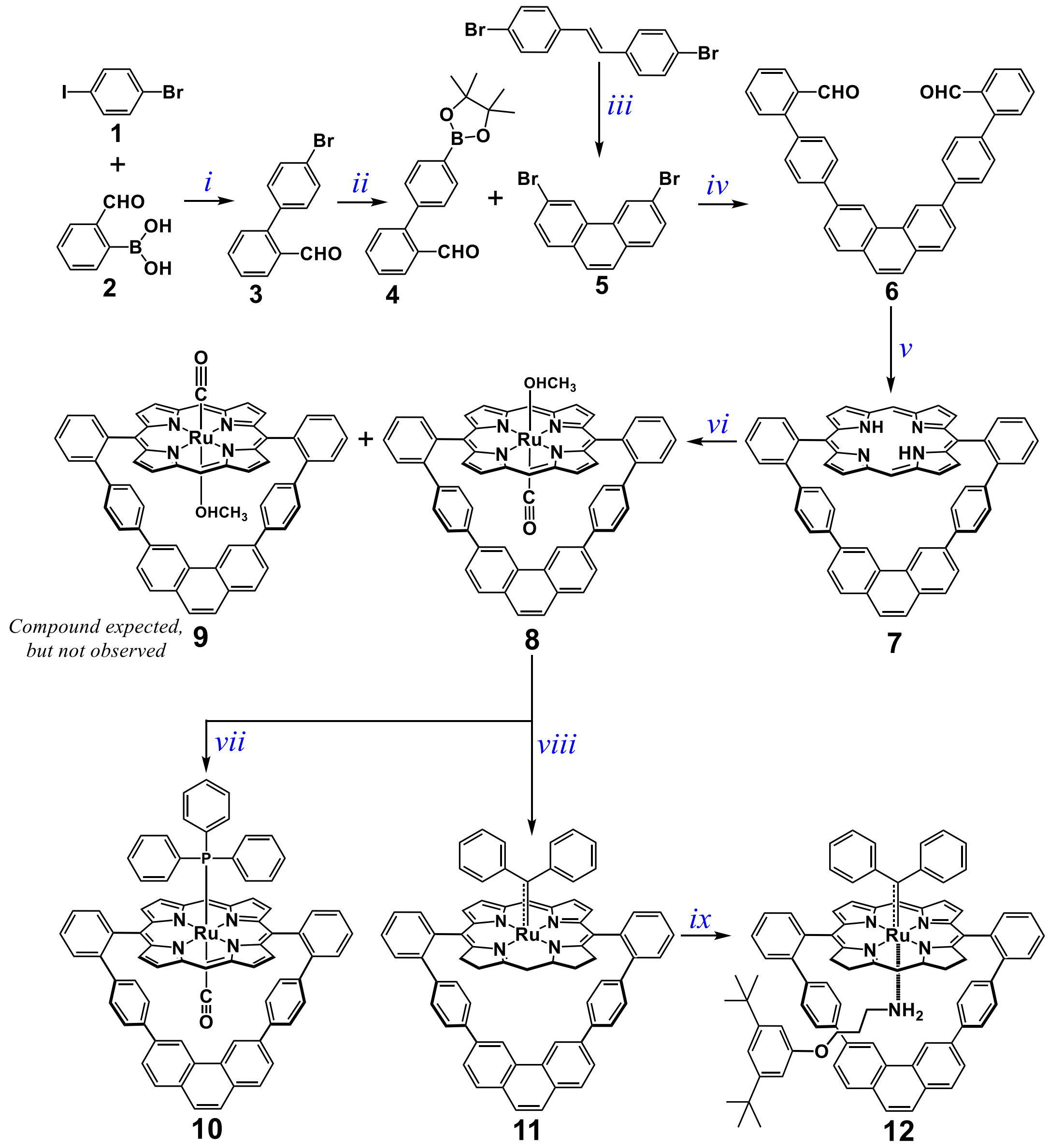

Figure S24 – Overview of the synthetic strategy used to prepare macrocycles and complexes. Reagents and conditions: *i*) $Pd(PPh_3)_4$, $Na_2CO_{3(aq)}$, MePh/EtOH (1:1, v/v), N_2 atmosphere, reflux, 16 h, 80%; *ii*) $PdCl_2dppf$, B_2pin_2, 1,4-dioxane, N_2 atmosphere, reflux, 16 h, 85%; *iii*) *hv* (medium-pressure Hg-lamp, 450 W, emission range 250-400 nm), I_2, epoxidized soybean oil, MePh, high dilution ($1x10^{-3}$ M), N_2 atmosphere, 35°C, 18 h, 80%; *iv*) $Pd(PPh_3)_4$, $Na_2CO_{3(aq)}$, PPh_3, MePh/MeOH (4:1, v/v), N_2 atmosphere, reflux, 48 h, 95%; *v*) (a) dipyrromethane, TFA, DCM/chloroform (3:1, v/v), high dilution ($2.13x10^{-4}$ M), N_2 atmosphere, rt, 20 h. (b) DDQ, reflux, 2 h, 20%; *vi*) Ru_3CO_{12}, 1,2,4-trichlorobenzene, N_2 atmosphere, 180°C, 2.5 h, 73%; *vii*) PPh_3, DCM, N_2 atmosphere, rt, 1 h, quantitative; *viii*) diphenyldiazomethane (dropwise addition), DCM, N_2 atmosphere, rt, 5 h, 64%; *ix*) compound **13**, $CDCl_3$, N_2 atmosphere, rt, 1 h, quantitative.

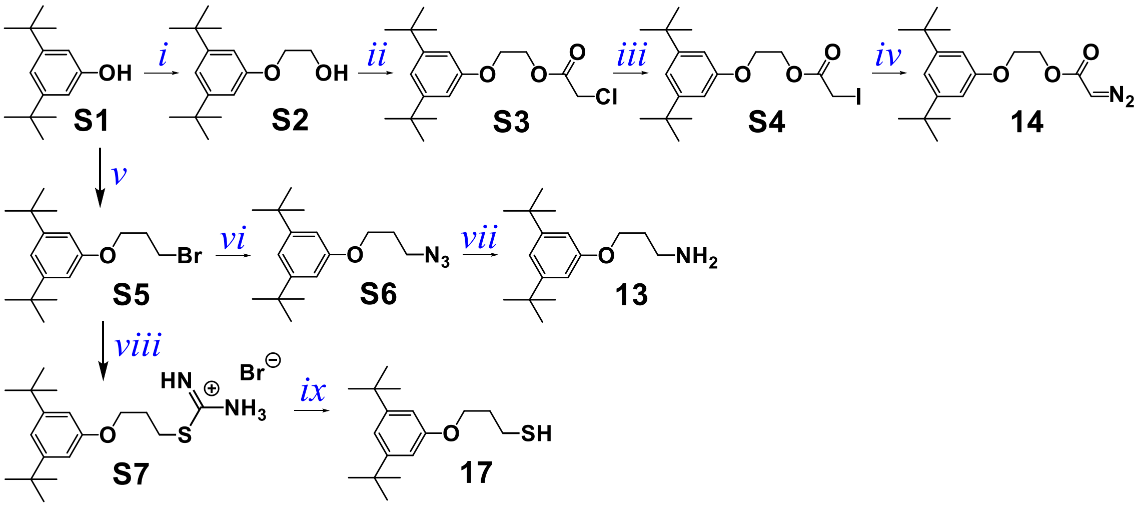

Figure S25 – Overview of the synthetic strategy used to prepare half-threads. Reagents and conditions: *i*) K_2CO_3, DMF, 2-bromoethanol, 90ºC, 30 h, 75%; *ii*) chloroacetic acid, CH_2Cl_2, BOP-Cl, Et_3N, N_2 atmosphere, rt, 12 h, 79%; *iii*) NaI, acetone, 3.5 h, rt, 86%; *iv*) (a) THF, *N,N'-bis*(*p*-toluenesulfonyl)hydrazine, DBU, N_2 atmosphere, 0ºC, 1 h. (b) $NaHCO_{3(aq)}$, N_2 atmosphere, 10 min, rt, 60%; *v*) K_2CO_3, 1,3-dibromopropane, EtOH, reflux, 5 h, 58%; *vi*) NaN_3, DMSO, rt, 20 h, 89%; *vii*) THF/H_2O (4:1, v/v), PPh_3, 24 h, rt, 67%; *viii*) thiourea, EtOH, 80ºC, 8h, quantitative; *ix*) KOH, EtOH, 80ºC, 6h, 80%.

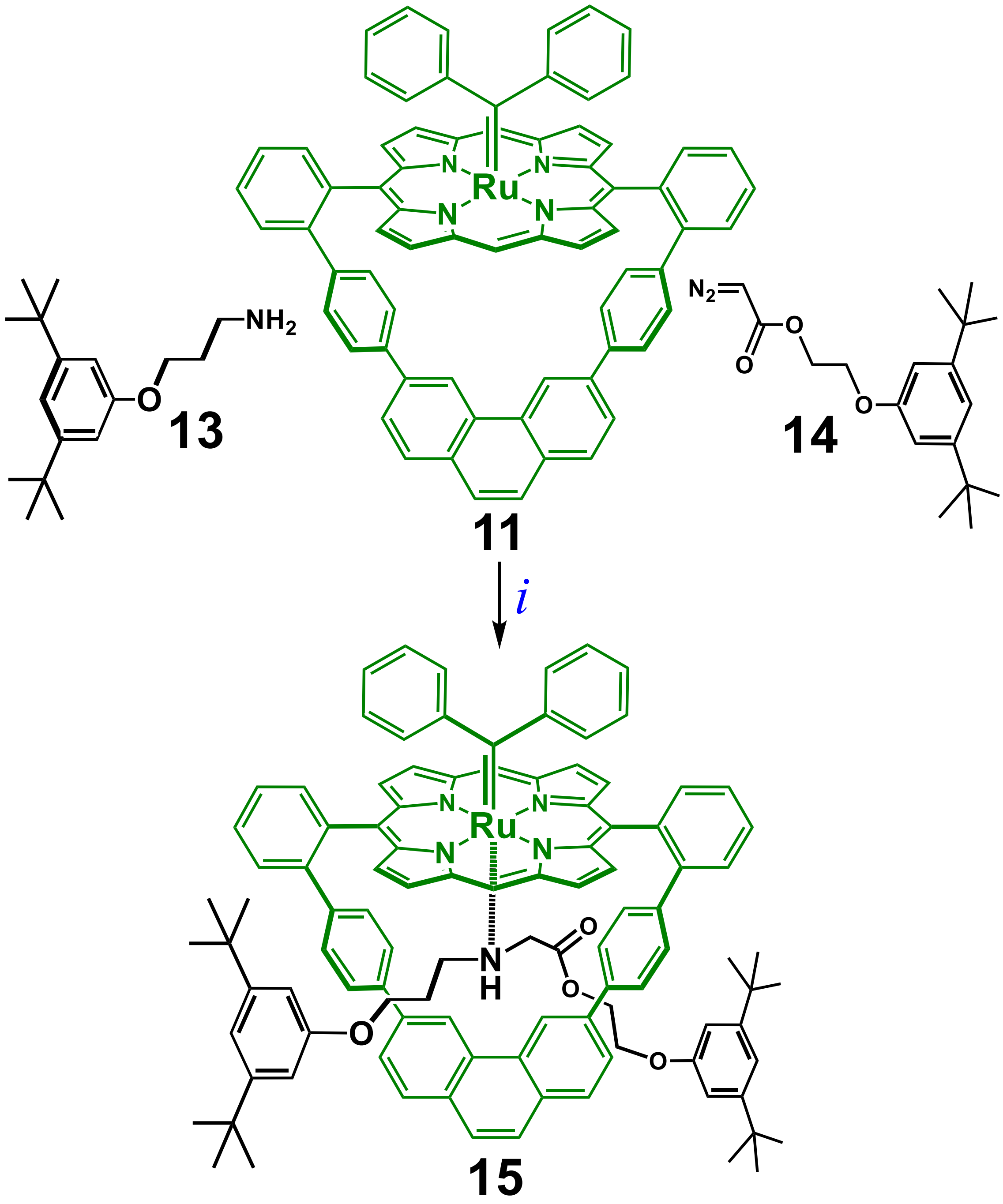

Figure S26 – Synthetic strategy used to prepare rotaxane 15. Reagents and conditions:
i) benzene, 60ºC, 8 h, quantitative relative to **11**.

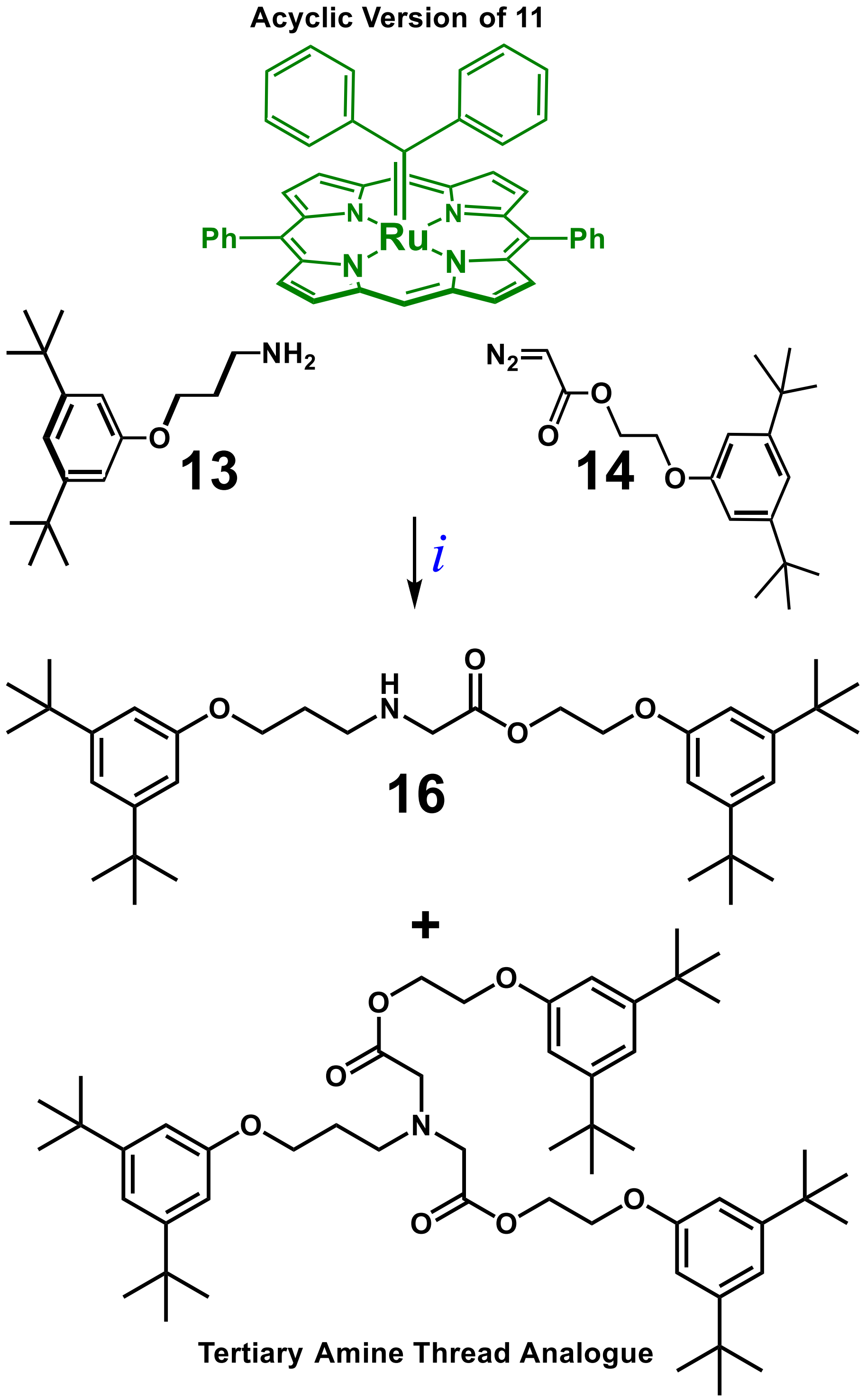

Figure S27 – Synthetic strategy used to prepare thread 16 using the Ru(II)porphyrinate acyclic version of macrocycle 11 as promoter. Reagents and conditions: *i*) benzene, 60°C, 8 h, quantitative.

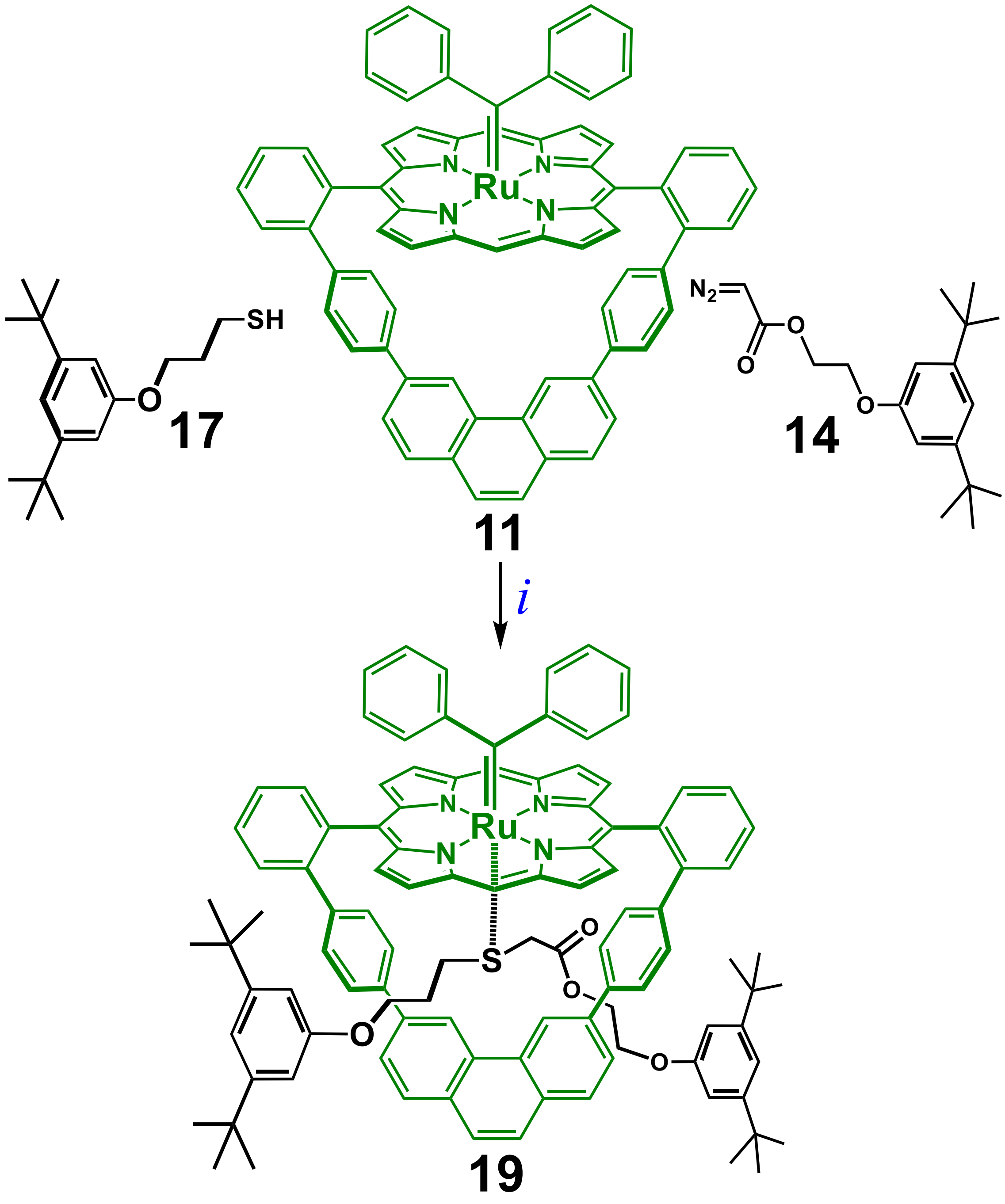

Figure S28 – Synthetic strategy used to prepare rotaxane 19. Reagents and conditions: *i*) benzene, rt, 4 h, quantitative relative to **11**.

Synthesis of 4'-bromo-[1,1'-biphenyl]-2-carbaldehyde **3**[S6]

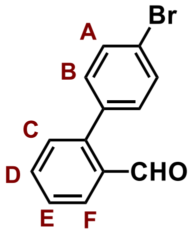

In a 250 mL Schlenk flask, commercially available 2-formylphenylboronic acid **2** (1.005 g, 6.70 mmol, 1.0 equiv), 1-bromo-4-iodobenzene **1** (2.086 g, 7.37 mmol, 1.1 equiv) and [Pd(PPh$_3$)$_4$] (0.387 g, 0.335 mmol, 5% relative to **2**) were dissolved in 28 mL of an oxygen-free solvent mixture composed of toluene/ethanol (1:1, v/v) at room temperature, under magnetic stirring and inert atmosphere. After complete dissolution, Na_2CO_3 (2.841 g, 26.80 mmol, 4.0 equiv), previously dissolved in 14 mL of oxygen-free distilled water, was added and the reaction mixture was heated at reflux under magnetic stirring and inert atmosphere for 16 h. The crude product was concentrated under reduced pressure and extracted with EtOAc (3 x 50 mL). The organic phase was washed with distilled water (3 x 50 mL), dried over Na_2SO_4, filtered through paper and concentrated under reduced pressure to afford a dark yellow solid. Final purification was achieved by column chromatography (SiO_2), using a hexanes/EtOAc gradient (9:1, v/v) as eluent to afford compound **3** as a white solid in 80% yield (1.397 g).

R.F. (hexanes/EtOAc, 4:1) = 0.70.

^{1}H NMR (500 MHz, $CDCl_3$), δ (ppm): 9.97 (s, 1H, CHO); 8.02 (dd, J = 0.96 and 7.80 Hz, 1H, H_F); 7.64 (td, J = 1.30 and 7.58 Hz, 1H, H_D); 7.61 (d, J = 8.36 Hz, 2H, H_A); 7.52 (t, J = 7.60 Hz, 1H, H_E); 7.40 (dd, J = 0.50 and 7.68 Hz, 1H, H_C); 7.25 (d, 2H, J = 8.37 Hz, H_B). Impurities: 1.56 (residual water in the $CDCl_3$ solvent); 0.07 (residual silicone grease).

^{13}C NMR (125 MHz, $CDCl_3$) δ (ppm): 191.9; 144.6; 136.8; 133.8; 133.7; 131.7; 131.6; 130.7; 128.3; 128.1; 122.8.

HRMS (ESI) (+): Calculated for $[C_{13}H_{10}BrO]^+$, 260.9910. Found *m/z* 260.9915.

FTIR (ATR), ν (cm^{-1}): 2842 and 2741 (C–H aldehyde); 1691 (C=O aldehyde); 760 (C–Br).

The spectroscopic and mass spectrometry data were consistent with those reported in the literature.[S6]

Synthesis of 4'-(4,4,5,5-tetramethyl-1,3,2-dioxaborolan-2-yl)-[1,1'-biphenyl]-2-carbaldehyde **4**

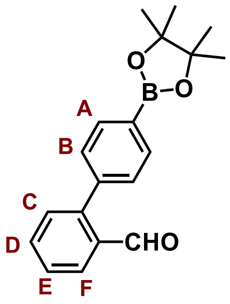

In a 50 mL Schlenk flask, compound **3** (0.63 g, 2.41 mmol, 1.0 equiv), *bis*(pinacolato)diboron (0.736 g, 2.91 mmol, 1.2 equiv), potassium acetate (1.183 g, 12.05 mmol, 5 equiv) and [1,1'-*bis*(diphenylphosphino)ferrocene]dichloropalladium(II) (0.099 g, 0.121 mmol, 5% relative **3**) were dissolved in 4 mL of oxygen-free 1,4-dioxane at room temperature and inert atmosphere. The reaction mixture was heated at reflux under magnetic stirring for 16 h. The crude was concentrated under reduced pressure and dissolved in 100 mL of EtOAc, washed with distilled water (3 x 50 mL), dried over Na_2SO_4, filtered through paper and concentrated under reduce pressure to afford a brown paste. Final purification was achieved by column chromatography (SiO_2), using a hexanes/EtOAc gradient (4:1, v/v) as eluent to afford compound **4** as a light-yellow solid in 85% yield (0.631 g).

R.F. (hexanes/EtOAc, 4:1) = 0.73.

Melting point: 142-143°C.

^{1}H NMR (500 MHz, $CDCl_3$), δ (ppm): 9.97 (s, 1H, CHO); 8.03 (d, J = 7.90 Hz, 1H, H_F); 7.91 (d, J = 8.00 Hz, 2H, H_A); 7.64 (td, J = 1.25 and 7.50 Hz 1H, H_D); 7.50 (t, J = 7.52 Hz, 1H, H_E); 7.44 (d, J = 7.76 Hz, 1H, H_C); 7.40 (d, J = 8.00 Hz, 2H, H_B); 1.38 (s, 12H, CH_3). Impurities: 1.59 (residual water in the $CDCl_3$ solvent); 0.07 (residual silicone grease).

^{13}C NMR (125 MHz, $CDCl_3$) δ (ppm): 192.4; 146.0; 140.6; 134.9; 133.8; 133.7; 130.8; 129.6; 128.0; 127.7; 84.1; 25.02; C–B not seen. Impurities: 1.15 (residual silicone grease).

HRMS (ESI) (+): Calculated for $[C_{19}H_{22}BO_3]^+$, 309.1657. Found *m/z* 309.1659.

FTIR (ATR), (cm^{-1}): 2862 and 2762 (C–H aldehyde stretching); 1691 (C=O aldehyde stretching); 1356 and 1325 ($C_2B_2O_2$ ring "breathing").[S6]

Synthesis of 3,6-dibromophenanthrene **5**[S7]

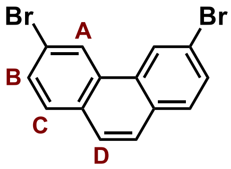

This reaction was carried out in a homemade photochemical reactor with 3.5 L capacity. The set up includes a triple jacketed immersion well, medium-pressure mercury lamp (450 W, emission range from 250 nm to 400nm, with peaks at 254 nm, 356 nm, and 365 nm), lamp power supply, a digital thermometer, a chiller and a magnetic stirrer. The quartz inner double jacketed well is equipped with inlet and outlet for cold water circulation to roughly

control the reaction temperature. The outer vessel is made of boronsilicate glass and is equipped with two ports (one for feeding and the other for the thermometer insertion) and gas inlet and outlet. In a typical run, *trans*-4,4′-dibromostilbene (0.845 g, 2.50 mmol, 1 equiv), iodine (0.698 g, 2.75 mmol, 1.1 equiv), epoxidized soybean oil with oxirane oxygen content of 6.0% (4.00 g) were dissolved in 2.5 L of oxygen-free toluene (previously degassed with N_2 for 20 min) at room temperature and magnetic stirring. Under a continuous nitrogen gas flow, the lamp was put in the inner double jacketed well and turned on. Ice-cold water circulation and the heat produced by the lamp led the reaction mixture to reach thermal equilibrium at about 35°C. Under those steady-state conditions, the reaction mixture was irradiated for 18 h, when TLC showed total conversion of the starting material. Toluene was evaporated under reduced pressure to yield a light orange oil. Successive washing of the crude with diethyl ether to remove the excess of epoxidized soybean oil yielded **5** as a light beige solid in 80% yield (0.672 g). An analytical sample can be afforded from recrystallization using slow diffusion of diethyl ether into a saturated dichloromethane solution.

R.F. (hexanes) = 0.48.

Melting Point = 194°C.

^{1}H NMR (500 MHz, $CDCl_3$), δ (ppm): 8.69 (d, J = 1.45 Hz, 2H, H_A); 7.76-7.66 (m, 6H, H_B+H_C+H_D). Impurities: 5.30 (residual dichloromethane); 1.55 (residual water in the $CDCl_3$ solvent).

^{13}C NMR (125 MHz, $CDCl_3$) δ (ppm): 130.9; 130.7; 130.6; 130.2; 126.9; 125.7; 121.3.

LRMS (EI) (+): Calculated for $C_{14}H_8Br_2$, 333.889. Found m/z 336.0 $[M]^+$ (90%) and 176.0 $[M – 2HBr]^+$ (100%).

The spectroscopic and mass spectrometry data were consistent with those reported in the literature.[S7]

Synthesis of 4',4'''-(phenanthrene-3,6-diyl)bis(([1,1'-biphenyl]-2-carbaldehyde)) **6**

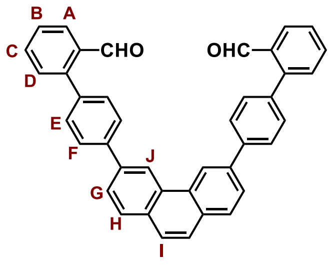

In a 250 mL Schlenk flask, compounds **5** (0.350 g, 1.04 mmol, 1 equiv), **4** (0.769 g, 2.49 mmol, 2.4 equiv), $[Pd(PPh_3)_4]$ (0.131 g, 0.113 mmol, 20% relative to **5**) and triphenylphosphine (0.100 g, 0.38 mmol, 36.5% relative to **5**) were dissolved in 25 mL of an oxygen-free solvent mixture composed of toluene/methanol (4:1, v/v) at room temperature, under magnetic stirring and inert atmosphere. After dissolution, Na_2CO_3 (2.00 g, 18.86 mmol, 18 equiv),

previously dissolved in 10 mL of oxygen-free distilled water, was added and the reaction mixture was heated at reflux for 48 h. The solvents were removed under reduced pressure and the remaining brown paste was dissolved in 50 mL of dichloromethane, washed with distilled water (3 x 50 mL), dried over Na_2SO_4, filtered through paper and concentrated under reduced pressure to yield a light brown solid. Flash chromatography on SiO_2 using DCM as eluent removed the residual black palladium. The solvent was evaporated under reduced pressure to yield a light-yellow solid. Successive washing of the light-yellow solid with diethyl ether to remove residual triphenylphosphine and excess of **4** afforded target compound **6** as a white solid in 95% yield (0.532 g).

R.F. (hexanes/EtOAc, 4:1) = 0.43.

Melting Point = 258-259°C.

^{1}H NMR (250 MHz, $CDCl_3$), *δ* (ppm): 10.12 (s, 2H, CHO); 9.03 (d, *J* = 1.40 Hz, 2H, H_L); 8.11-8.00 (m, 4H, H_C and H_I); 7.96-7.89 (m, 6H, H_H and H_J); 7.83 (s, 2H, H_K); 7.69 (t, *J* = 7.33 Hz, 2H, H_E); 7.60-7.50 (m, 8H, H_G, H_D and H_F). Impurities: 5.30 (residual dichloromethane); 3.48 and 1.21 (residual diethyl ether); 1.57 (residual water in the $CDCl_3$ solvent).

^{13}C NMR (60 MHz, $CDCl_3$), δ (ppm): 192.6; 145.6; 141.5; 138.8; 137.1; 134.0; 133.8; 131.9; 130.9; 130.8; 130.7; 129.6; 128.1; 128.0; 127.8; 127.1; 126.3; 121.3. Impurity: 53.4 (residual dichloromethane).

MALDI-TOF (pristine) (+): Calculated for $C_{40}H_{26}O_2$, 538.1933. Found *m/z* 538.1936 $[M]^+$.

FTIR (ATR), ν (cm^{-1}): 2853 and 2762 (C–H aldehyde); 1687 (C=O aldehyde).

Synthesis of macrocycle **7**

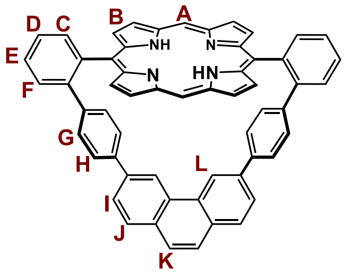

In a two-necked round bottom flask of 2 L capacity, compound **6** (0.115 g, 0.213 mmol, 1 equiv) and dipyrromethane[S8] (0.063 g, 0.43 mmol, 2 equiv) were dissolved in 1 L of an oxygen-free solvent mixture composed of 750 mL of dichloromethane and 250 mL of chloroform at room temperature and inert atmosphere. TFA (0.745 g, 6.53 mmol, 30 equiv, 0.50 mL) was added and the red reaction mixture was stirred at room temperature for 20 h. DDQ (0.420 g, 1.85 mmol, 8.5 equiv) was added as a solid and the resulting black mixture was heated at reflux for 2 h. After cooling, the crude was neutralized with Et_3N (5 mL). The solvent was removed under reduced

pressure and the resulting black paste was directly loaded into a column chromatography (SiO_2) with dichloromethane. Flash chromatography using DCM as eluent removed black tar material. The crude product was concentrated under reduced pressure. Washing the crude with diethyl ether to remove impurities followed by filtration through paper yielded target macrocycle **7** as a purple solid in 20% (0.034 g).

R.F. (hexanes/DCM, 1:1) = 0.62.

^{1}H NMR (500 MHz, $CDCl_3$), δ (ppm): 10.11 (s, 2H, H_A); 9.22 (d, J = 4.50 Hz, 4H, H_B); 8.99 (d, J = 4.50 Hz, 4H, H_B); 8.61 (d, J = 7.26 Hz, 2H, H_C); 8.01-7.94 (m, 4H, H_D and H_F); 7.88 (td, J = 1.81 and 7.23 Hz, 2H, H_E); 7.64 (d, J = 8.06 Hz, 2H, H_J); 7.47 (s, 2H, H_K); 7.44 (dd, J = 1.40 and 8.30 Hz, 2H, H_I); 7.00 (s, 2H, H_L); 6.75 (d, J = 8.40 Hz, 4H, H_G); 6.55 (d, J = 8.40 Hz, 4H, H_H); -2.98 (s, 2H, N–H). Impurities: 5.30 (residual dichloromethane); 3.48 and 1.21 (residual diethyl ether); 1.54 (residual water in the $CDCl_3$ solvent); 1.26 and 0.88 (residual "grease"); 0.07 (residual silicone "grease").

^{13}C NMR (125 MHz, $CDCl_3$) δ (ppm): Macrocycle **7** was too insoluble to record ^{13}C NMR.

MALDI-TOF (pristine) (+): Calculated for $C_{58}H_{36}N_4$, 788.294. Found *m/z* 788.282 $[M]^+$.

UV-Vis (CH_2Cl_2), 10^{-5} mol/L, λ_{max} (nm): 274 and 322 (aromatic molecular loop); 413 (Soret), 507, 541, 580 (Q-bands).

Synthesis of macrocycle **8**

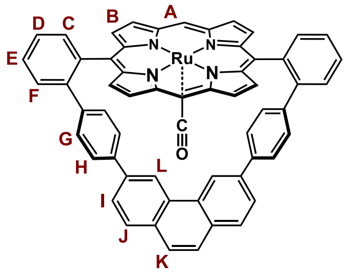

In a 50 mL Schlenk flask, macrocycle **7** (0.030 g, 0.038 mmol, 1 equiv) and $Ru_3(CO)_{12}$ (0.030 g, 0.047 mmol, 1.25 equiv) were dissolved in 9.0 mL of 1,2,4-trichlorobenzene under inert atmosphere. The reaction mixture was heated at 180°C and stirred for 2.5 hours. The crude product was cooled to room temperature and directly loaded on a neutral alumina chromatography column. The 1,2,4-trichlorobenzene solvent was removed using hexanes (100%) as eluent. The target macrocycle was then eluted using a mixture of dichloromethane/methanol (99:1) as eluent. The resulting red solid was dissolved in 20 mL of a mixture composed of dichloromethane/methanol (80:20) and concentrated under reduced pressure to afford a red solid, which was washed with methanol (3 x 10 mL) and petroleum ether (3 x 10 mL). Macrocycle **8** was afforded in 73% yield as a red solid (0.025 g).

R.F. (DCM/Methanol, 99.5:0.5) = 0.91.

^{1}H NMR (500 MHz, $CDCl_3$), δ (ppm): 9.86 (s, 2H, H_A); 9.01 (d, J = 4.67 Hz, 4H, H_B); 8.77 (d, J = 4.67 Hz, 4H, H_B); 8.44 (d, J = 7.20 Hz, 2H, H_C); 7.97-7.91 (m, 4H, H_E and H_F); 7.80 (t, J = 7.24 Hz, 2H, H_D); 7.57 (d, J = 8.14 Hz, 2H, H_J); 7.41 (s, 2H, H_K); 7.38 (d, J = 8.17 Hz, 2H, H_I); 6.83 (s, 2H, H_L); 6.80 (d, J = 8.28 Hz, 4H, H_G); 6.46 (d, J = 8.30 Hz, 4H, H_H). Impurities: 5.29 (dichloromethane); 3.39 and 1.24 (methanol); 1.77 (residual water); 0.06 (residual silicone "grease"). In order to improve the solubility of **8**, 0.5% (v/v) of regular methanol was added to the $CDCl_3$ solvent for the NMR analyses.

^{13}C NMR (125 MHz, $CDCl_3$) δ (ppm): 180.1; 144.6; 144.3; 143.5; 140.7; 140.6; 139.9; 139.1; 139.0; 134.5; 132.0; 131.8; 130.5; 129.8; 129.2; 129.0; 128.7; 128.2; 126.9; 126.0; 125.5; 125.2; 121.8; 119.7; 107.0; 106.4; 1.07. Impurities: 50.5 (methanol); 29.7 (silicon grease).

MALDI-TOF (pristine) (+): Calculated for $C_{59}H_{34}N_4ORu$, 916.177. Found *m/z* 887.921 $[M - CO]^+$.

UV-Vis (CH_2Cl_2), 10^{-5} mol/L, λ_{max} (nm): 275 and 312 (phenanthrene moiety); 406 (Soret), 523, 555 (Q-bands).

FTIR (ATR), ν (cm^{-1}): 1926 (CO).

Synthesis of macrocycle **10**

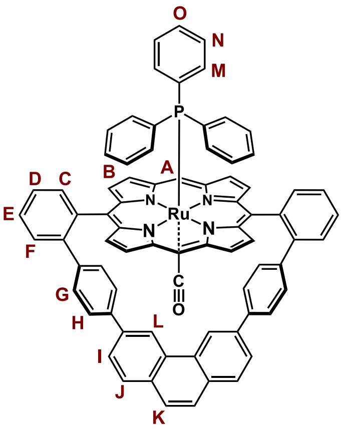

In a 50 mL Schlenk flask under inert atmosphere, macrocycle **8** (0.010 g, 0.011 mmol, 1 equiv) and triphenylphosphine (PPh_3) (0.024 g, 0.088 mmol, 8 equiv) were dissolved in dichloromethane (15 mL) and stirred at room temperature for 1 h. Petroleum ether (50 mL) was added and the solvent was evaporated under reduced pressure to precipitate target complex **10** as a red solid. The resulting material was washed with petroleum ether (3 x 10 mL) and dried under reduce pressure to afford macrocycle **10** in quantitative yield (0.012 g).

^{1}H NMR (250 MHz, $CDCl_3$), δ (ppm): 9.48 (s, 2H, H_A); 8.82 (d, J = 4.57 Hz, 4H, H_B); 8.59 (d, J = 4.57 Hz, 4H, H_B); 8.04 (d, J = 6.63 Hz, 2H, H_C); 7.96-7.82 (m, 4H, H_D and H_F); 7.79-7.69 (m, 2H, H_E); 7.57 (d, J = 7.95 Hz, 2H, H_J); 7.43-7.34 (m, 4H, H_K and H_I); 6.97-6.73 (m, 9H, H_G, H_L and H_O); 6.69-6.34 (m, 10H, H_H and H_N); 3.88 (br, 6H, H_M). Impurities: 1.46 residual water; 1.26, 0.88 and 0.07 (aliphatic impurities).

^{13}C NMR (125 MHz, $CDCl_3$) δ (ppm): Macrocycle **10** was too insoluble to record ^{13}C NMR.

MALDI-TOF (pristine) (+): Calculated for $C_{77}H_{49}N_4OPRu$, 1178.268. Found *m/z* 1149.630 $[M - CO]^+$ and *m/z* 887.724 $[M - CO - PPh_3]^+$.

UV-Vis (CH_2Cl_2), 10^{-5} mol/L, λ_{max} (nm): 274 and 313 (phenanthrene moiety); 407 (Soret), 523, 552 (Q-bands).

FTIR (ATR), ν (cm^{-1}): 1946 (CO).

Synthesis of macrocycle **11**

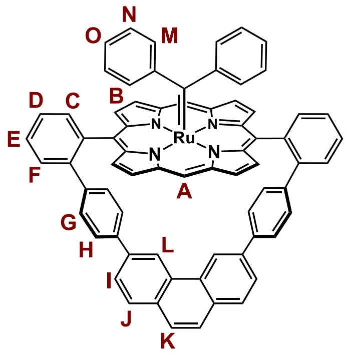

In a 100 mL Schlenk flask under inert atmosphere, macrocycle **8** (0.010 g, 0.011 mmol, 1 equiv) was dissolved in 20 mL of dichloromethane under magnetic stirring at room temperature A dichloromethane solution of freshly prepared diphenyldiazomethane[S2] (0.008g, 0.040 mmol, in 4.00 mL of dichloromethane) was added dropwise to the solution of **8** over 3 hours. At the end of the addition, the reaction mixture was magnetically stirred for another 1 h. The solvent was removed under reduced pressure and the crude product was purified by neutral alumina chromatography column, using a mixture of petroleum ether/DCM (1:1, v/v) as eluent to afford macrocycle **11** as a red solid in 64% yield (7.4 mg). Increasing the polarity of the eluent to DCM/MeOH (99:1, v/v) allows the recovering of macrocycle **8** as starting material in 36% yield (3.5 mg), which can be reused in another batch.

R.F. (Petroleum ether/DCM, 1:1) = 0.56.

^1H NMR (400 MHz, $CDCl_3$), δ (ppm): 9.34 (s, 2H, H_A); 8.66 (d, J = 4.74 Hz, 4H, H_B); 8.47 (d, J = 4.70 Hz, 4H, H_B); 8.03 (d, J = 7.36 Hz, 2H, H_C); 7.90-7.85 (m, 4H, H_E and H_F); 7.79-7.73 (m, 2H, H_D); 7.65 (d, J = 8.20 Hz, 2H, H_J); 7.49 (s, 2H, H_K); 7.46 (d, J= 8.39 Hz, 2H, H_I); 7.17 (s, 2H, H_L); 6.91 (d, J= 8.17 Hz, 4H, H_G); 6.65 (d, J = 8.17 Hz, 4H, H_H); 6.44 (t, J = 7.30 Hz, 2H, H_O); 6.10 (t, J = 7.56 Hz, 4H, H_N); 2.88 (d, J = 7.56 Hz, 4H, H_M). Impurities: 5.30 (dichloromethane); 1.26 and 0.88 (aliphatic impurities).

^{13}C NMR (60 MHz, $CDCl_3$) δ (ppm): Macrocycle **11** was too insoluble to record ^{13}C NMR.

MALDI-TOF (pristine) (+): Calculated for $C_{71}H_{44}N_4Ru$, 1054.260. Found *m/z* 1054.191 $[M]^+$ and *m/z* 888.121 $[M – diphenylcarbene\ ligand]^+$

UV-Vis (CH_2Cl_2), 10^{-5} mol/L, λ_{max} (nm): 274 and 317 (phenanthrene moiety); 391, 424, 527, 550.

Synthesis of 2-(3,5-di-tert-butylphenoxy)ethan-1-ol **S2**.

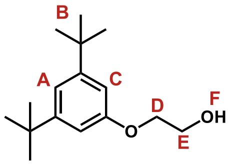

In a 50 mL Schlenk flask, under inert atmosphere, 3,5-di-tert-butylphenol **S1** (0.20 g, 1,00 mmol, 1 equiv) was dissolved in anhydrous dimethylformamide (10 mL). To this solution were added potassium carbonate (1.40 g, 10 mmol, 10 equiv) and 2-bromoethan-1-ol (0.49 g, 4.00 mmol, 4 equiv). The resulting reaction mixture was heated at 90 ºC and magnetically stirred for 30 hours. The dimethylformamide was removed under reduced pressure and the crude was dissolved in diethyl ether (80 mL), washed with water (3 x 50 mL), dried over Na_2SO_4, filtered through paper and concentrated under reduced pressure to give a yellowish oil. Final purification was achieved by column chromatography (SiO_2), using a hexanes/ethyl acetate gradient (95:5, v/v) as eluent to afford compound **S2** as a colorless oil, in 75 % yield (0.19 g).

R.F. (hexanes/ethyl acetate 4:1, v/v) = 0.72

^{1}H NMR (250 MHz, $CDCl_3$), δ (ppm): 7.06 (t, J = 1.63 Hz, 1H, H_A); 6.79 (d, J = 1.63 Hz, 2H, H_C); 4.11 (m, 2H, H_D); 3.97 (m, 2H, H_E); 2.16 (t, J = 6.04 Hz, 1H, H_F); 1.32 (s, 18H, H_B). Impurities: 1.68 (residual water in the $CDCl_3$ solvent).

^{13}C NMR (60 MHz, $CDCl_3$) δ (ppm): 158.3; 152.5; 115.5; 109.07; 69.1; 61.7; 35.1; 31.5.

HRMS (ES) (+): Calculated for $C_{16}H_{26}O_2$, 250.1933. Found *m/z* $[M + H]^+$ 251.2030.

Synthesis of 2-(3,5-di-tert-butylphenoxy)ethyl 2-chloroacetate **S3**.

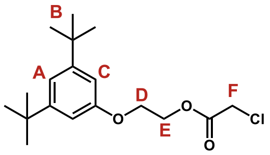

In a 50 mL Schlenk flask, under inert atmosphere, compound **S2** (0.21g, 0.84 mmol, 1 equiv) and chloroacetic acid (0.098g, 1.08 mmol, 1.30 equiv) were dissolved in 15 mL of dichloromethane. To this solution were added *bis*(2-oxo-3-oxazolidinyl)phosphinic chloride (BOP-Cl) (0.43 g, 1.68 mmol, 2 equiv) and triethylamine (0.14 mL). The reaction mixture was stirred at room temperature for 12 hours. The solvent was removed under reduced pressure and the crude was dissolved in diethyl ether (100 mL), washed with water (3 x 50 mL), dried over Na_2SO_4, filtered through paper and concentrated under reduced pressure. Final purification was achieved by column chromatography (SiO_2), using a hexanes/ethyl acetate gradient (96:4, v/v) as eluent to afford compound **S3** as a light-yellow oil, in 79 % yield (0.22 g).

R.F. (hexane/ethyl acetate 4:1, v/v) = 0.65

^{1}H NMR (250 MHz, $CDCl_3$), δ (ppm): 7.06 (t, J = 1.63 Hz, 1H, H_A); 6.77 (d, J = 1.63 Hz, 2H, H_C); 4.56 (t, J = 4.80 Hz, 2H, H_D); 4.22 (t, J= 4.82 Hz, 2H, H_E); 4.12 (s, 2H, H_F); 1.32 (s, 18H, H_B). Impurities: 5.30 (dichloromethane) and 1.59 (residual water in the $CDCl_3$ solvent).

^{13}C NMR (60 MHz, $CDCl_3$) δ (ppm): 167.5; 158.0; 152.5; 115.7; 109.0; 65.6; 64.8; 40.9; 35.1; 31.5.

HRMS (ES) (+): Calculated for $C_{18}H_{27}ClO_3$, 326.1649. Found *m/z* $[M + H]^+$ 327.1740.

FTIR (ATR), ν (cm^{-1}): 1760.

Synthesis of 2-(3,5-di-tert-butylphenoxy)ethyl 2-iodoacetate **S4**.

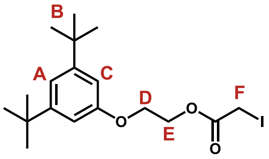

In a 50 mL Schlenk flask, under inert atmosphere, compound **S3** (0.20 g, 0.61 mmol, 1 equiv) was dissolved in 10 mL of acetone. Sodium iodide (0.27 g, 1.83 mmol, 3 equiv) was added to the reaction flask and the solution was stirred at room temperature for 3.5 hours. Acetone was removed under reduced pressure and the crude was dissolved in diethyl ether (100 mL), washed with a 5% (w/w) sodium thiosulfate aqueous solution to remove iodine impurities, followed by washing with water (3 x 50 mL), dried over sodium sulfate, filtered through paper and evaporated to dryness under reduced pressure to afford compound **S4** as a colorless oil in 86 % yield (0.22 g).

R.F. (hexanes/ethyl acetate 4:1, v/v) = 0.78

^{1}H NMR (250 MHz, $CDCl_3$), δ (ppm): 7.06 (s, 1H, H_A); 6.78 (d, J = 1.26 Hz, 2H, H_C); 4.51 (t, J = 4.80 Hz 2H, H_D); 4.21 (t, J = 4.83 Hz 2H, H_E); 3.75 (s, 2H, H_F); 1.32 (s, 18H, H_B). Impurities: 1.58 (residual water in the $CDCl_3$ solvent).

^{13}C NMR (60 MHz, $CDCl_3$) δ (ppm): 169.0; 158.0; 152.5; 115.7; 109.1; 65.6; 64.7; 35.1; 31.5; -5.6.

HRMS (ES) (+): Calculated for $C_{18}H_{27}O_3I$, 418.1005. Found *m/z* $[M + H]^+$ 419.1065.

FTIR (ATR), ν (cm^{-1}): 1736.

Synthesis of 2-(3,5-di-tert-butylphenoxy)ethyl 2-diazoacetate **14**.

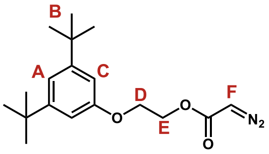

In a 50 mL Schlenk flask, under inert atmosphere, compound **S4** (0.10 g, 0.24 mmol, 1.0 equiv) was dissolved in tetrahydrofuran (8.0 mL) and *N,N'-bis*(*p*-toluenesulfonyl)hydrazine (0.09 g, 0.26 mmol, 1.08 equiv) was added. The resulting mixture was cooled to 0ºC in an ice bath and

stirred at this temperature for 10 minutes. Then, 1,8-diazabicyclo[5.4.0]undec-7-ene (0.15 g, 0.96 mmol, 4.0 equiv) was added dropwise to this solution and the resulting mixture was stirred for 45 min in the ice bath. A saturated aqueous solution of sodium bicarbonate (10 mL) was added and the reaction mixture was stirred for 10 minutes and allowed to cool to room temperature. The crude product was extracted with diethyl ether (3 x 50 mL), dried over Na_2SO_4, filtered through paper and concentrated under reduced pressure. Final purification was achieved by column chromatography (SiO_2), using a hexanes/ethyl acetate gradient (96:4, v/v) as eluent to afford compound **14** as a yellowish oil, in 60 % yield (0.046 g).

R.F. (hexane/ethyl acetate 4:1, v/v) = 0.62

^{1}H NMR (250 MHz, $CDCl_3$), δ (ppm): 7.06 (t, J = 1.58 Hz, 1H, H_A); 6.79 (d, J = 1.58 Hz, 2H, H_C); 4.82 (br, 1H, H_F); 4.53 (t, J = 4.60 Hz, 2H, H_D); 4.21 (t, J = 4.43 Hz; 2H, H_E); 1.33 (s, 18H, H_B). Impurities: 1.69 (residual water in the $CDCl_3$ solvent).

^{13}C NMR (60 MHz, $CDCl_3$) δ (ppm): 166.9; 158.2; 152.5; 115.6; 109.0; 66.0; 63.4; 46.5; 35.1; 31.5.

HRMS (ES) (+): Calculated for $C_{18}H_{26}N_2O_3$, 318.1943. Found *m/z* 341.1835 [M + Na^+].

FTIR (ATR), ν (cm^{-1}): 2106, 1698, 1678.

Synthesis of 1-(3-bromopropoxy)-3,5-di-tert-butylbenzene **S5**.

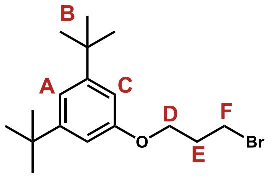

In a 100 mL Schlenk flask, under inert atmosphere, 3,5-di-tert-butylphenol **S1** (0.20 g, 1.00 mmol, 1.0 equiv) was dissolved in ethanol (20 mL). Potassium carbonate (0.28 g, 2.00 mmol, 2.0 equiv) and 1,3-dibromopropane (1.00 g, 5.00 mmol, 5.0 equiv) were added and the solution was stirred at reflux for 5 hours. The solvent was removed under reduced pressure and the resulting oil was dissolved in diethyl ether (100 mL), washed with water (3 x 50 mL), dried over sodium sulfate, filtered through paper and concentrated under reduced pressure. Final purification was achieved by column chromatography (SiO_2), using a hexanes/ethyl acetate gradient (96:4, v/v) as eluent to afford compound **S5** as a colorless oil, in 58 % yield (0.19 g).

R.F. (hexanes/ethyl acetate 96:4, v/v) = 0.76.

^{1}H NMR (250 MHz, $CDCl_3$), δ (ppm): 7.04 (t, J = 1.60 Hz, 1H, H_A); 6.77 (d, J = 1.60 Hz, 2H, H_C); 4.12 (t, J = 6.02 Hz, 2H, H_D); 3.63 (t, J = 6.51 Hz, 2H, H_F); 2.33 (q, J = 6.00 Hz; 2H, H_E); 1.32 (s, 18H, H_B). Impurities: 1.56 (residual water in the $CDCl_3$ solvent).

^{13}C NMR (60 MHz, $CDCl_3$) δ (ppm): 158.4; 152.4; 115.4; 108.9; 65.2; 35.1; 32.8; 31.60; 30.3.

HRMS (ES) (+): Calculated for $C_{17}H_{27}BrO$, 326.1245. Found *m/z* $[M + H]^+$ 327.1348.

Synthesis of 1-(3-azidopropoxy)-3,5-di-tert-butylbenzene **S6**.

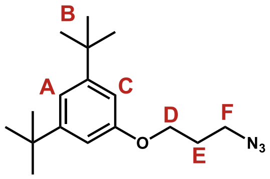

In a 50 mL Schlenk flask, under inert atmosphere, compound **S5** (0.17 g, 0.52 mmol, 1.00 equiv) and sodium azide (0.051 g, 0.78 mmol, 1.50 equiv) were dissolved in DMSO (4.0 mL) and stirred at room temperature for 20 hours. Water (20 mL) was added to the reaction flask and the crude extracted with diethyl ether (3 x 50 mL). The organic fractions were combined, dried over Na_2SO_4, filtered through paper and concentrated under reduced pressure. Final purification was achieved by column chromatography (SiO_2), using hexanes as eluent to afford compound **S6** as a colorless oil, in 89 % yield (0.14 g).

R.F. (hexane) = 0.18.

^{1}H NMR (250 MHz, $CDCl_3$), δ (ppm): 7.04 (t, J = 1.52 Hz, 1H, H_A); 6.76 (d, J = 1.52 Hz, 2H, H_C); 4.07 (t, J = 5.90 Hz, 2H, H_D); 3.54 (t, J = 6.64 Hz, 2H, H_F); 2.06 (q, J = 6.26 Hz; 2H, H_E); 1.33 (s, 18H, H_B). Impurities: 1.57 (residual water in the $CDCl_3$ solvent).

^{13}C NMR (60 MHz, $CDCl_3$) δ (ppm): 158.4; 152.4; 115.4; 109.0; 64.5; 48.5; 35.1; 31.6; 29.1.

HRMS (ES) (+): Calculated for $C_{17}H_{27}N_3O$, 289.2154. Found *m/z* $[M + H]^+$ 290.2277.

FTIR (ATR), ν (cm^{-1}): 2097.

Synthesis of 3-(3,5-di-tert-butylphenoxy)propan-1-amine **13**.

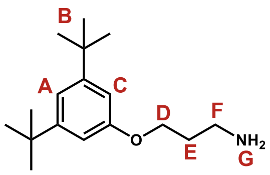

In a 50 mL Schlenk flask, under inert atmosphere, compound **S6** (0.10 g, 0.36 mmol, 1.0 equiv) was dissolved in a solvent mixture of tetrahydrofuran/H_2O (10 mL, 8:2 v/v). Triphenylphosphine (0.14 g, 0.54 mmol, 1.5 equiv) was added to the reaction flask and the mixture was stirred at room temperature for 24 hours. An aqueous solution of sodium hydroxide (10 mL, 1.0 M) was added to the reaction flask and the mixture was stirred for another 1 h at room temperature. The solvent was removed under reduced pressure and the crude was dissolved in diethyl ether (50 mL), washed with water (3 x 25 mL), dried with Na_2SO_4, filtered through paper and concentrated under reduced pressure. Final purification was achieved by column chromatography (SiO_2), using a

dichloromethane/methanol gradient (95:5, v/v) as eluent to afford compound **13** as a colorless oil, in 67 % yield (0.064 g).

R.F. (dichloromethane/methanol 97:3, v/v) = 0.21.

^{1}H NMR (250 MHz, $CDCl_3$), δ (ppm): 7.04 (br, 1H, H_A); 6.79 (d, J = 1.50 Hz, 2H, H_C); 4.08 (t, J = 6.03 Hz, 2H, H_D); 2.94 (t, J = 6.75 Hz, 2H, H_F); 1.95 (q, J = 6.30 Hz; 2H, H_E); 1.79 (br, 2H, H_G); 1.33 (s, 18H, H_B).

^{13}C NMR (60 MHz, $CDCl_3$) δ (ppm): 158.6; 152.3; 115.1; 108.9; 65.7; 39.4; 35.1; 33.09; 31.5. Impurities: 53.5 (residual dichloromethane); 14.2; 22.8; 29.8 (aliphatic impurities).

HRMS (ES) (+): Calculated for $C_{17}H_{29}NO$, 263.2249. Found *m/z* $[M + H]^+$ 264.2279.

Synthesis of 2-(3-(3,5-di-*tert*-butylphenoxy)propyl)isothiouronium bromide **S7.**

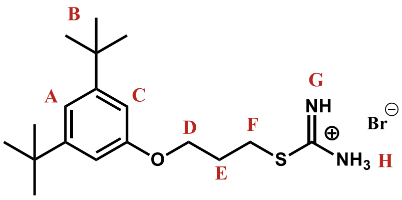

In a 100 mL Schlenk flask, under inert atmosphere, compound **S5** (0.17 g, 0.52 mmol, 1.00 equiv) and thiourea (0.05 g, 0.65 mmol, 1.25 equiv) were dissolved in ethanol (20.0 mL) and stirred at 80 ºC for 8 hours. The solvent was removed under reduced pressure and the resulting solid was washed with diethyl ether (3 x 15 mL) and dried under reduced pressure to afford compound **S7** as a white solid, in 86 % yield (0,18 g).

^{1}H NMR (250 MHz, DMSO-d_6), δ (ppm): 9.01 (br, 3H, H_G); 7.30-6.85 (m, 5H, H_H and H_A); 6.71 (d, J = 1.46 Hz, 2H, H_C); 4.04 (t, J = 5.80 Hz, 2H, H_D); 3.30 (overlap with residual water, H_F); 2.05 (q, J = 6.65 Hz; 2H, H_E); 1.26 (s, 18H, H_B).

Synthesis of 3-(3,5-di-tert-butylphenoxy)propane-1-thiol **17.**

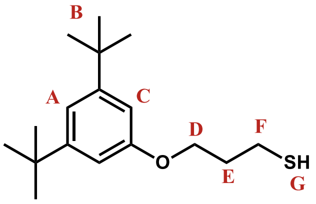

In a 100 mL Schlenk flask, under inert atmosphere, compound **S7** (0.16 g, 0.40 mmol, 1.0 equiv) and sodium hydroxide (0.040 g, 1.00 mmol, 2.5 equiv) were dissolved in ethanol (30.0 mL) and stirred at 80 ºC for 8 hours. The reaction mixture was cooled to room temperature and neutralized with a H_2SO_4 aqueous solution (1 mol/L, 4.0 mL). The solvents were removed under reduced pressure and water (20 mL) was added to the crude, which was extracted with diethyl ether (3 x 50 mL). The combined organic fractions were dried over Na_2SO_4, filtered through paper, and concentrated under reduced pressure. Final purification was achieved by column chromatography (SiO_2), using an hexanes/dichloromethane gradient (80:20, v/v) as eluent to afford compound **17** as a colorless oil, in 61 % yield (0.068 g).

R.F. (petroleum ether/dichloromethane 60:40, v/v) = 0.86.

^{1}H NMR (250 MHz, $CDCl_3$), δ (ppm): 7.03 (t, J = 1.58 Hz, 1H, H_A); 6.76 (d, J = 1.58 Hz, 2H, H_C); 4.09 (t, J = 5.92 Hz, 2H, H_D); 2.76 (q, J = 7.19 Hz, 2H, H_F); 2.09 (q, J = 6.40 Hz; 2H, H_E); 1.42 (t, J = 8.16 Hz, 1H, H_G); 1.31 (s, 18H, H_B).

^{13}C NMR (60 MHz, $CDCl_3$) δ (ppm): 158.5; 152.3; 115.2; 108.9; 65.5; 35.1; 33.7; 31.6; 21.5.

HRMS (ES) (+): Calculated for $C_{17}H_{28}OS$, 280.1861. Found *m/z* $[M + Na]^+$ 303.1464.

Synthesis of rotaxane **15**.

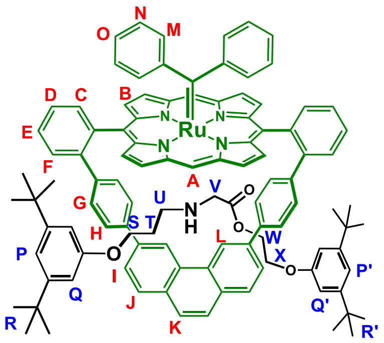

In a 10 mL Schlenk flask, under inert atmosphere, macrocycle **11** (10 mg, 0.0094 mmol, 1.0 equiv) and half-thread **13** (24.7 mg, 0.094 mmol, 10.0 equiv) were dissolved in 1.2 mL of benzene at room temperature. The resulting solution was stirred for 15 minutes at rt. Compound **14** (29.9 mg, 0,094 mmol, 10.0 equiv) was added as a solid to the reaction flask and the mixture was heated at 60ºC for 8 hours. The crude mixture was evaporated to dryness under reduced pressure. The crude product was dissolved in a minimum amount of petroleum ether. The excess of half-thread **14** was insoluble in the petroleum-ether phase and was removed using a pipette as a colorless oil. The petroleum-ether solution was purified by preparative TLC on silica using petroleum ether/dichloromethane (50:50, v/v) as eluent to afford the target rotaxane **15** as a red solid in quantitative yield (15.0 mg) as the first fraction. The excess of half-thread **13** was isolated as the second fraction as a yellowish oil.

R.F. (Petroleum ether/DCM, 1:1) = 0.79.

^{1}H NMR (250 MHz, $CDCl_3$), *δ* (ppm): 9.09 (s, 1H, H_A); 9.00 (s, 1H, H_A); 8.64 (d, J = 4.83 Hz, 2H, H_B); 8.52 (d, J = 4.59 Hz, 2H, H_B); 8.47 (d, J = 4.83 Hz, 4H, H_B); 8.26 (d, J = 4.83 Hz, 4H, H_B); 8.16 (d, J = 7.49 Hz, H_C); 7.88-7.65 (m, 6H, H_D, H_E and H_F); 7.58 (d, J = 8.29 Hz, 2H, H_J); 7.44 (s, 2H, H_K); 7.32 (d, J = 8.29, 2H, H_I); 7.26 (s, 2H, H_L); 7.09 (d, J = 8.29, 4H, H_G); 6.98 (t, J = 1.48 Hz, 1H, $H_{P'}$); 6.66-6.58 (m, 5H, H_P and H_H); 6.47 (d, J = 1.43, 2H, $H_{Q'}$); 6.43 (t, J = 7.36, 2H, H_O); 6.10 (t, J = 7.73, 4H, H_N); 5.61 (d, J = 1.52 Hz, H_Q); 3.48 (br, 2H, H_X); 3.37 (br, 2H, H_W); 2.91 (d, J = 7.10, 4H, H_M); 1.87 (t, J = 5.35 Hz, 2H, H_S); 1.25 (s, 18H, $H_{R'}$); 0.94 (s, 18H, H_R); from -1.76 to -2.45 (br, 4H, H_V and H_U); -2.86 (br, 2H, H_T); -4.25 (br, 1H, NH).

Impurities: 1.56 (residual water in the $CDCl_3$ solvent); 0.07 (silicon “grease”).

^{13}C NMR (60 MHz, $CDCl_3$) δ (ppm): 330.3; 168.2; 162.7; 158.0; 157.9; 152.2; 151.3; 144.6; 144.1; 143.9; 143.4; 143.1; 141.3; 140.3; 140.0; 139.7; 134.9; 133.1; 132.8; 131.8; 131.6; 130.8; 129.9; 129.6; 129.5; 128.1; 127.9; 126.2; 125.8; 125.2; 123.4; 123.1; 118.4; 115.2; 113.9; 111.6; 108.8; 108.0; 107.4; 107.0; 64.7; 64.3; 62.4; 53.5; 46.1; 44.2; 35.0; 34.6; 31.5; 31.3; 29.8; 22.8; 22.1; 14.2. Impurities: 53.5 (residual dichloromethane); 29.8; 22.8 and 14.2 (aliphatic impurities).

MALDI-TOF (pristine) (+): Calculated for $C_{106}H_{99}N_5O_4Ru$, 1607.6741. Found *m/z* 1607.603 $[M]^+$ and 1441.494 $[M – \text{diphenylcarbene ligand}]^+$.

UV-Vis (CH_2Cl_2), 10^{-5} mol/L, λ_{max} (nm): 270; 313; 398, 425 and 530.

FTIR (ATR), ν (cm^{-1}): 1741.

Synthesis of thread **16**

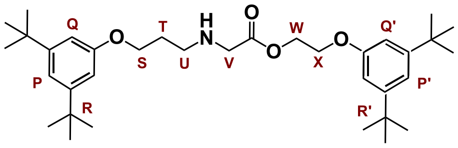

This compound was isolated from the reaction between half-threads **13** and **14** using Ru(II)diphenylporphyrinate with the diphenyl carbene axial ligand, the acyclic version of **11**, as promoter under the exact same conditions employed for the rotaxane **15** assembly process. This experiment was performed for comparison purposes, which highlighted the special endotopic features of **11**. In a 10 mL Schlenk flask, Ru(II)diphenylporphyrinate with the diphenyl carbene axial ligand (6.8 mg, 9.4 μmol, 1.0 equiv) and half-thread **13** (24.7 mg, 94.0 μmol, 10.0 equiv) were dissolved in 1.2 mL of benzene under inert atmosphere at room temperature. The resulting solution was stirred for 15 minutes at room temperature. Compound **14** (29.9 mg, 94.0 μmol, 10.0 equiv) was added in one portion as a solid to the reaction flask and the mixture was heated at 60°C for 8 hours. The crude mixture was evaporated to dryness under reduced pressure. The crude was purified by preparative TLC on silica using chloroform as eluent. A first fraction was isolated as a light-yellow oil in 65% yield relative to **14** (25.7 mg), which corresponded to the tertiary amine analogue that was formed from the second carbenoid insertion into the secondary amine group of thread **16**. The second more polar fraction corresponded to thread **16**, which was isolated as a colorless oil in 26% yield relative to **14** (13.5 mg).

Thread **16**: R.F. thread **16** (silica, chloroform) = 0.19.

^1H NMR (250 MHz, $CDCl_3$), δ (ppm): 7.05 and 7.02 (s, 2H, H_P and $H_{P'}$); 6.77 (br, 4H, H_Q and $H_{Q'}$); 4.51 (t, *J* = 5.00 Hz, 2H, H_X); 4.21 (t, *J* = 5.00 Hz, 2H, H_W); 4.07 (t, *J* = 6.00

Hz, 2H, H_S); 3.53 (s, 2H, H_V); 2.88 (t, *J* = 6.80 Hz, 2H, H_U); 2.24 (br, 1H, N<u>H</u>); 2.01 (q, *J* = 6.50 Hz, 2H, H_T); 1.31 (s, 36H, H_R and $H_{R'}$). Impurities: 1.26 (residual "grease"); 0.08 (residual silicone "grease").

<u>HRMS (ESI) (+)</u>: Calculated for [$C_{35}H_{55}NO_4$] 553.4131. Found *m/z* 553.4119.

<u>Tertiary amine thread analogue:</u>

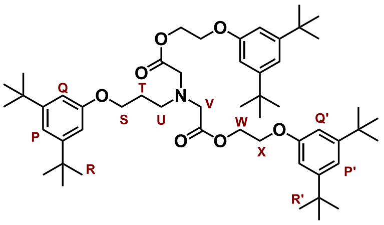

<u>R.F. (silica, chloroform)</u> = 0.58.

<u>^{1}H NMR (250 MHz, $CDCl_3$), *δ* (ppm)</u>: 7.04 (br, 2H, $H_{P'}$), 7.02 (s, 1H, H_P); 6.80-6.70 (m, 6H, H_Q and $H_{Q'}$); 4.46 (t, *J* = 5.00 Hz, 4H, H_X); 4.18 (t, *J* = 5.00 Hz, 4H, H_W); 4.03 (t, *J* = 6.00 Hz, 2H, H_S); 3.67 (s, 4H, H_V); 2.97 (t, *J* = 6.80 Hz, 2H, H_U); 2.01 (q, *J* = 6.50 Hz, 2H, H_T); 1.30 (s, 54H, H_R and $H_{R'}$). Impurities: 1.26 (residual "grease"); 0.08 (residual silicone "grease").

<u>HRMS (ESI) (+)</u>: Calculated for [$C_{53}H_{81}NO_7$] 843.6013. Found *m/z* 843.6019.

<u>Synthesis of rotaxane **19**.</u>

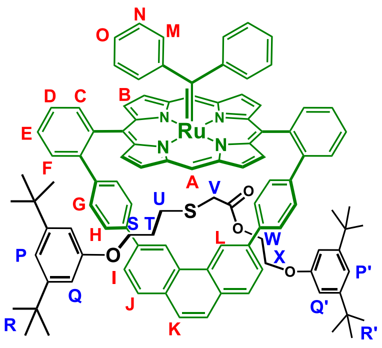

In a 10 mL Schlenk flask, under inert atmosphere, macrocycle **11** (10 mg, 0.0094 mmol, 1.0 equiv) and half-thread **17** (26.0 mg, 0.094 mmol, 10.0 equiv) were dissolved in 1.2 mL of benzene at room temperature. The resulting solution was stirred for 15 minutes at rt. Compound **14** (29.9 mg, 0,094 mmol, 10.0 equiv) was added as a solid to the reaction flask and the mixture was magnetically stirred at room temperature for 4.0 hours. TLC analyses on silica revealed that macrocycle **11** was completely interlocked after that period. The crude mixture was evaporated to dryness under reduced pressure. The crude product was dissolved in a minimum amount of petroleum ether and purified by preparative TLC on silica using petroleum ether/dichloromethane (50:50, v/v) as eluent to afford the target rotaxane **19** as a red solid in quantitative yield (91% isolated yield; 14.0 mg) as the second fraction. The excess of half-threads **17** and **14** was completely recovered as the first (colorless oil) and third (yellowish oil) fractions, respectively.

<u>R.F. (silica, petroleum ether/dichloromethane, 1:1)</u> = 0.79.

<u>^{1}H NMR (500 MHz, $CDCl_3$), δ (ppm):</u> 9.16 (s, 1H, H_A); 9.08 (s, 1H, H_A); 8.63 (d, J = 4.55 Hz, 2H, H_B); 8.53 (d, J = 4.73 Hz, 2H, H_B); 8.48 (d, J = 4.55 Hz, 4H, H_B); 8.36 (d, J

= 4.77 Hz, 4H, H_B); 8.23 (d, J = 6.57 Hz, H_C); 7.84 (td, J= 7.62 Hz e 1.01 Hz, 2H H_D); 7.75 (td, J= 7.53 Hz e 1.28 Hz, 2H, H_E); 7.70 (d, J = 7.67, 2H, H_F); 7.58 (d, J = 8.26 Hz, 2H, H_J); 7.44 (s, 2H, H_K); 7.37 (s, 2H, H_L); 7.33 (d, J = 8.35, 2H, H_I); 7.03 (d, J = 8.26, 4H, H_G); 6.94 (br, 1H, $H_{P'}$); 6.71 (br, 1H, H_P); 6.65 (d, J = 8.37 Hz, 4H, H_H); 6.49 (t, J = 7.44, 2H, H_O); 6.39 (s, 2H, $H_{Q'}$); 6.15 (t, J = 7.79, 4H, H_N); 5.85 (s, 2H, H_Q); 3.18 (br, 2H, H_X); 2.99 (d, J = 7.25, 4H, H_M); 2.73 (br, 2H, H_W); 2.38 (br, J = 5.35 Hz, 2H, H_S); 1.23 (s, 18H, $H_{R'}$); 1.00 (s, 18H, H_R); -1.38 (br, 2H, H_V); -1.90 (br, 2H, H_U); -2.05 (s, 2H, H_T). Impurities: 1.58 (residual water); 1.26 e 0.07 (aliphatic impurities).

^{13}C NMR (60 MHz, $CDCl_3$) δ (ppm): 167.3; 161.4; 158.0; 157.7; 152.2; 151.7; 144.9; 144.5; 143.8; 143.2; 143.1; 141.0; 140.2; 139.7; 139.4; 135.0; 133.0; 132.7; 131.9; 131.7; 130.8; 130.1; 129.8; 129.7; 128.3; 128.2; 128.0; 126.2; 125.7; 125.4; 125.2; 123.9; 123.6; 119.0; 115.2; 114.2; 112.6; 108.6; 108.1; 107.4; 107.0; 65.4; 64.4; 62.5; 35.0; 34.7; 31.5; 31.3; 29.8; 28.6; 28.0; 23.7. Impurities: 29.8; 22.8 e 14.2 (aliphatic impurities).

MALDI-TOF (+): Calculado para $C_{106}H_{98}N_4O_4SRu$, 1624.6352. Experimental *m/z* 1624.446 $[M]^+$.

4. Spectral Data

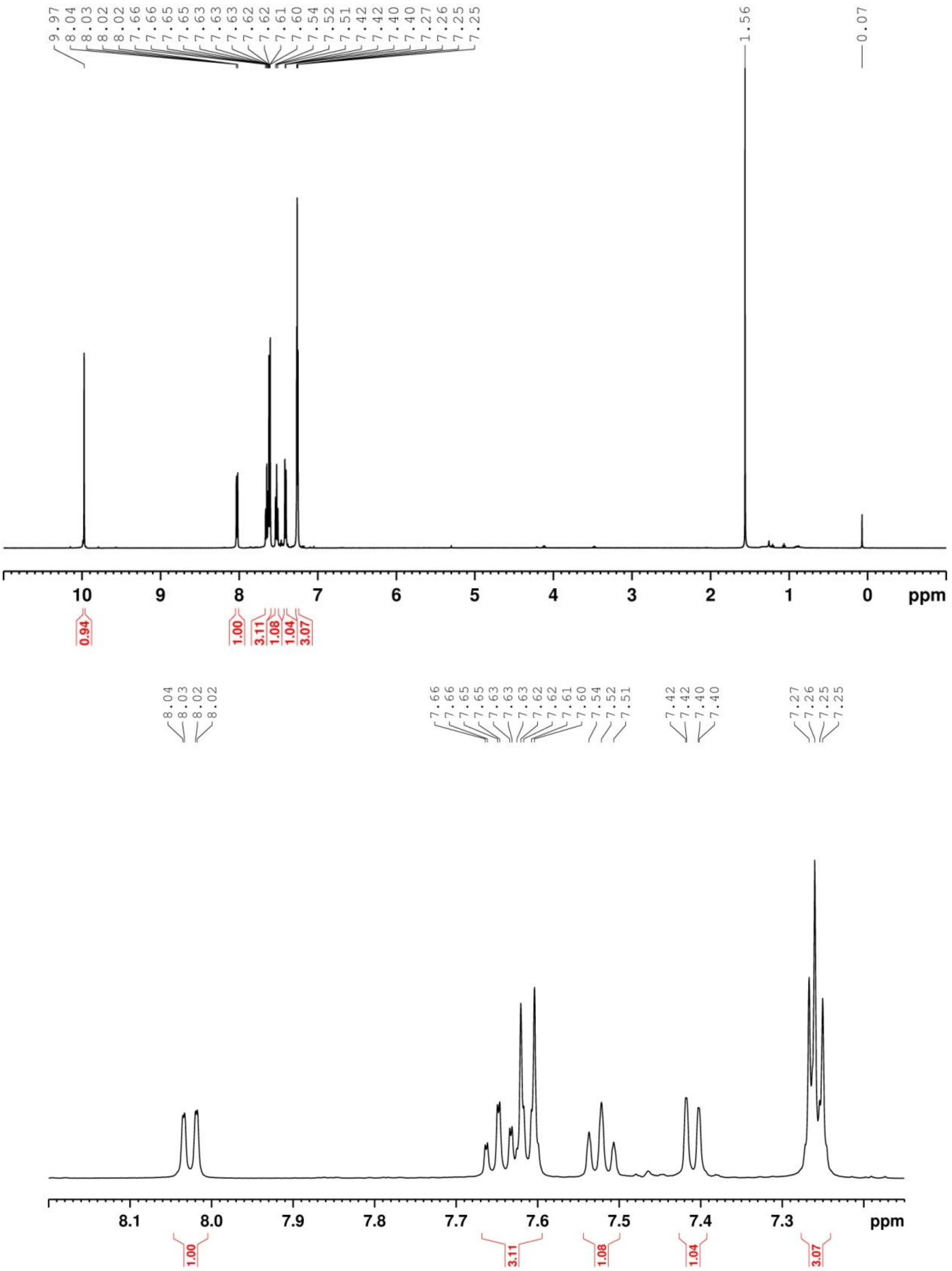

Figure S29 – ^{1}H NMR spectrum of compound **3** (500 MHz, $CDCl_3$, 298 K).

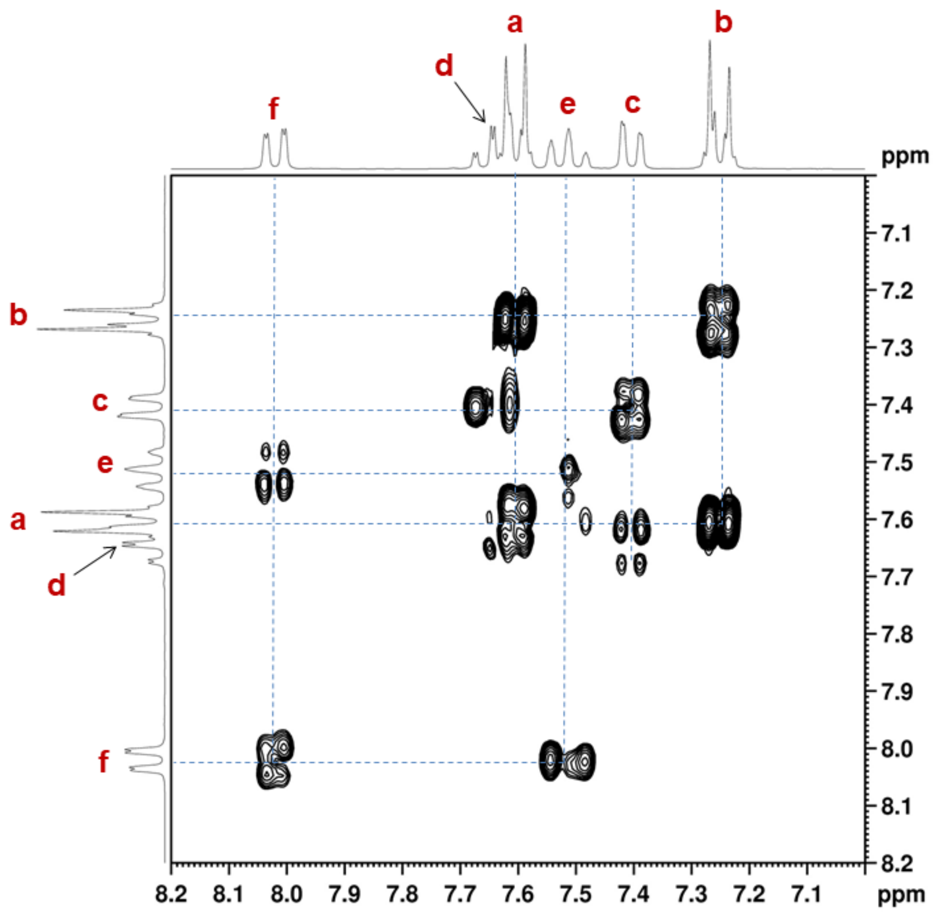

Figure S30 – Tow dimensional (^1H-^1H) COSY NMR spectrum of compound **3** (250 MHz, $CDCl_3$, 298 K).

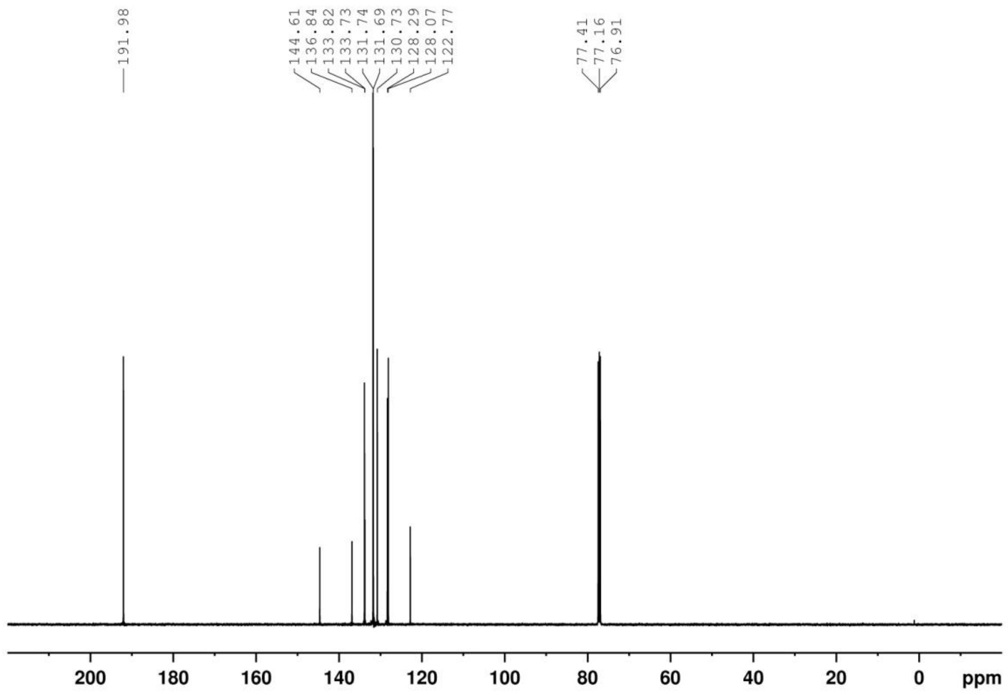

Figure S31 – ^{13}C NMR spectrum of compound **3** (125 MHz, $CDCl_3$, 298 K).

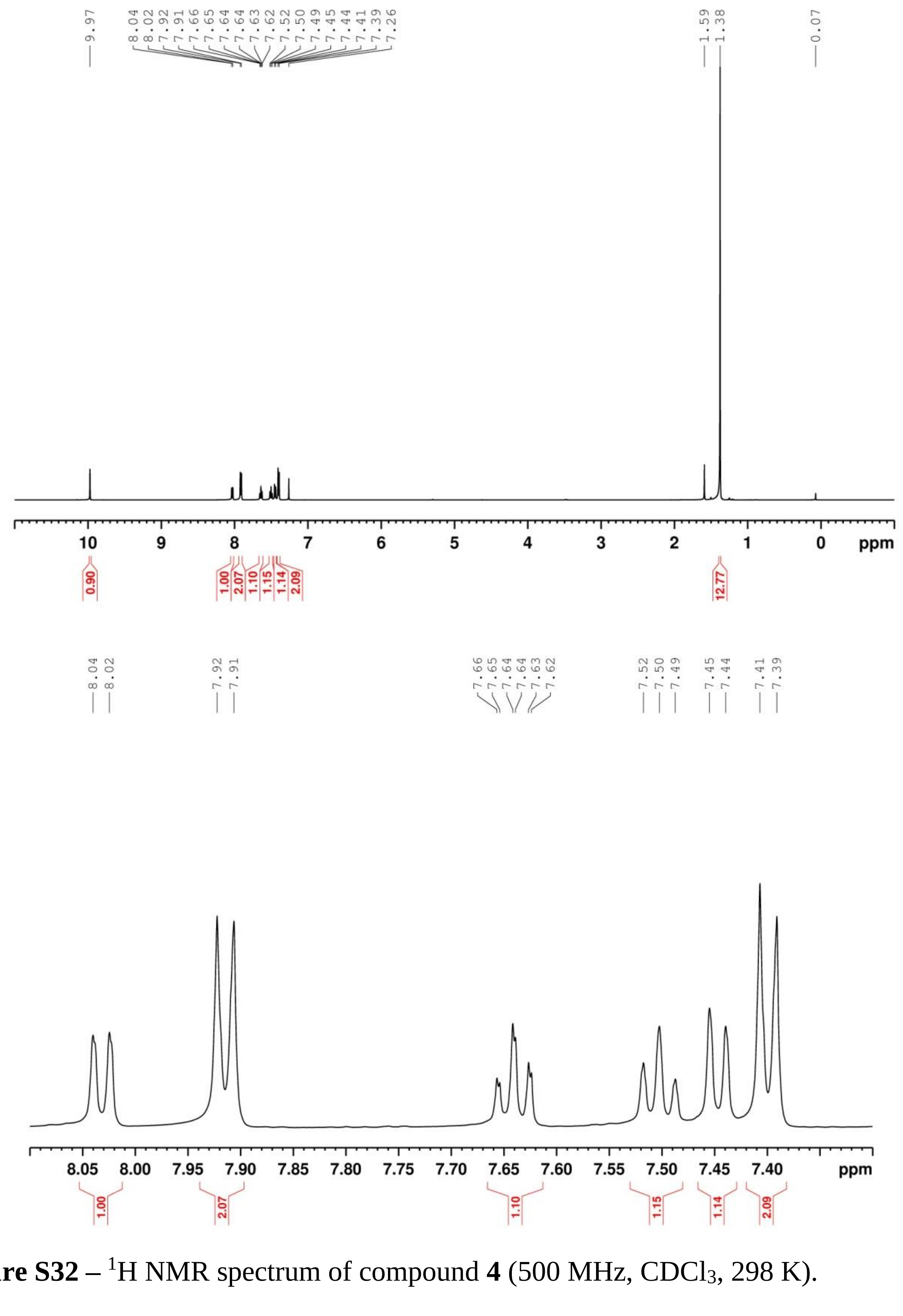

Figure S32 – ^{1}H NMR spectrum of compound **4** (500 MHz, $CDCl_3$, 298 K).

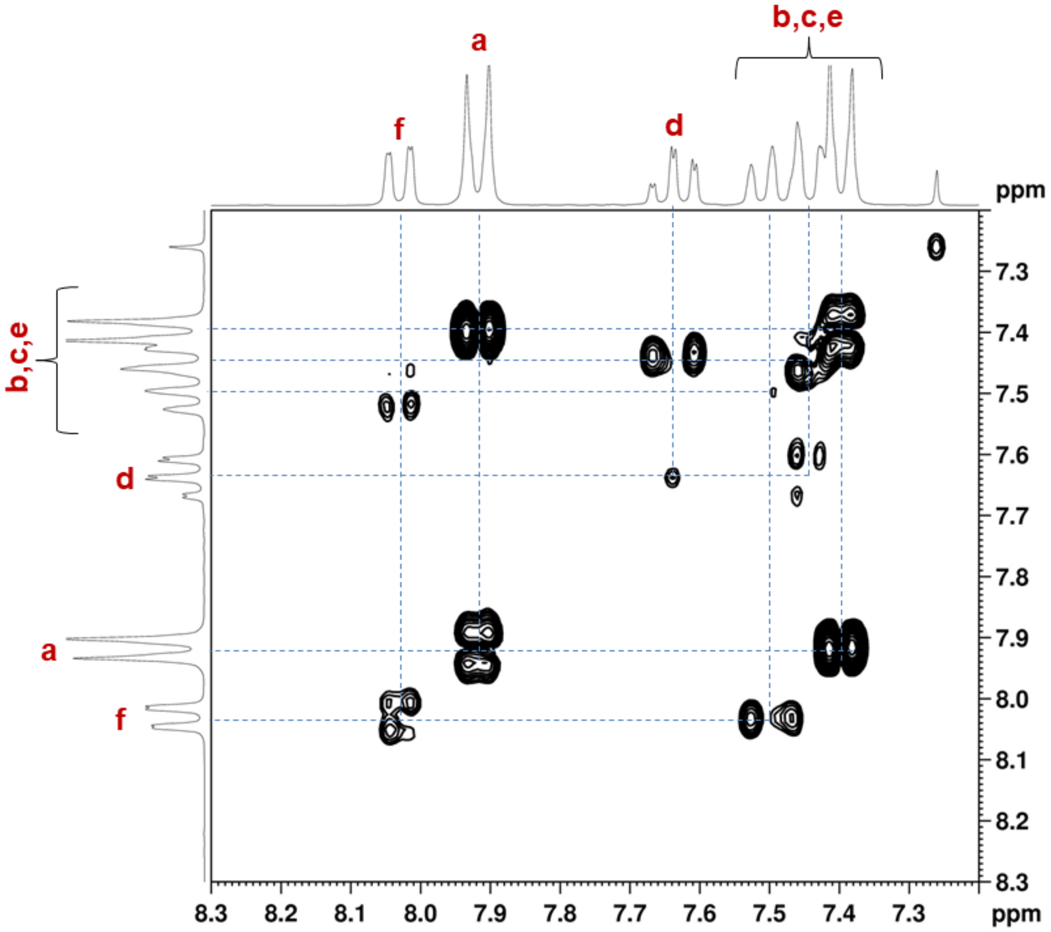

Figure S33 – Two-dimensional (^1H-^1H) COSY NMR spectrum of compound **4** (250 MHz, $CDCl_3$, 298 K).

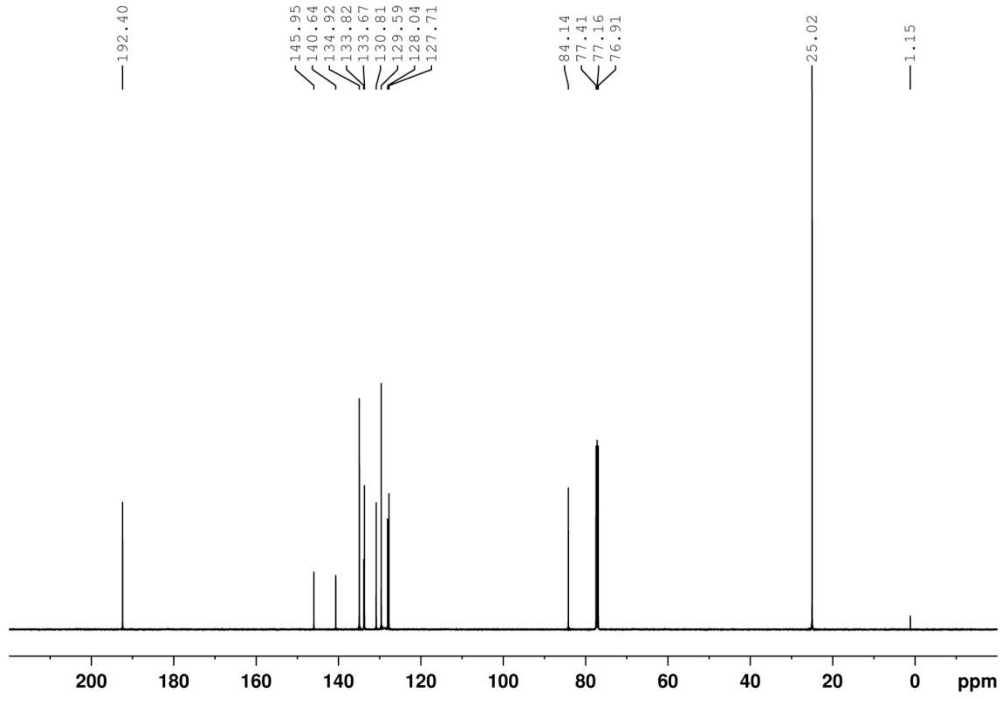

Figure S34 – ^{13}C NMR spectrum of compound **4** (125 MHz, $CDCl_3$, 298 K).

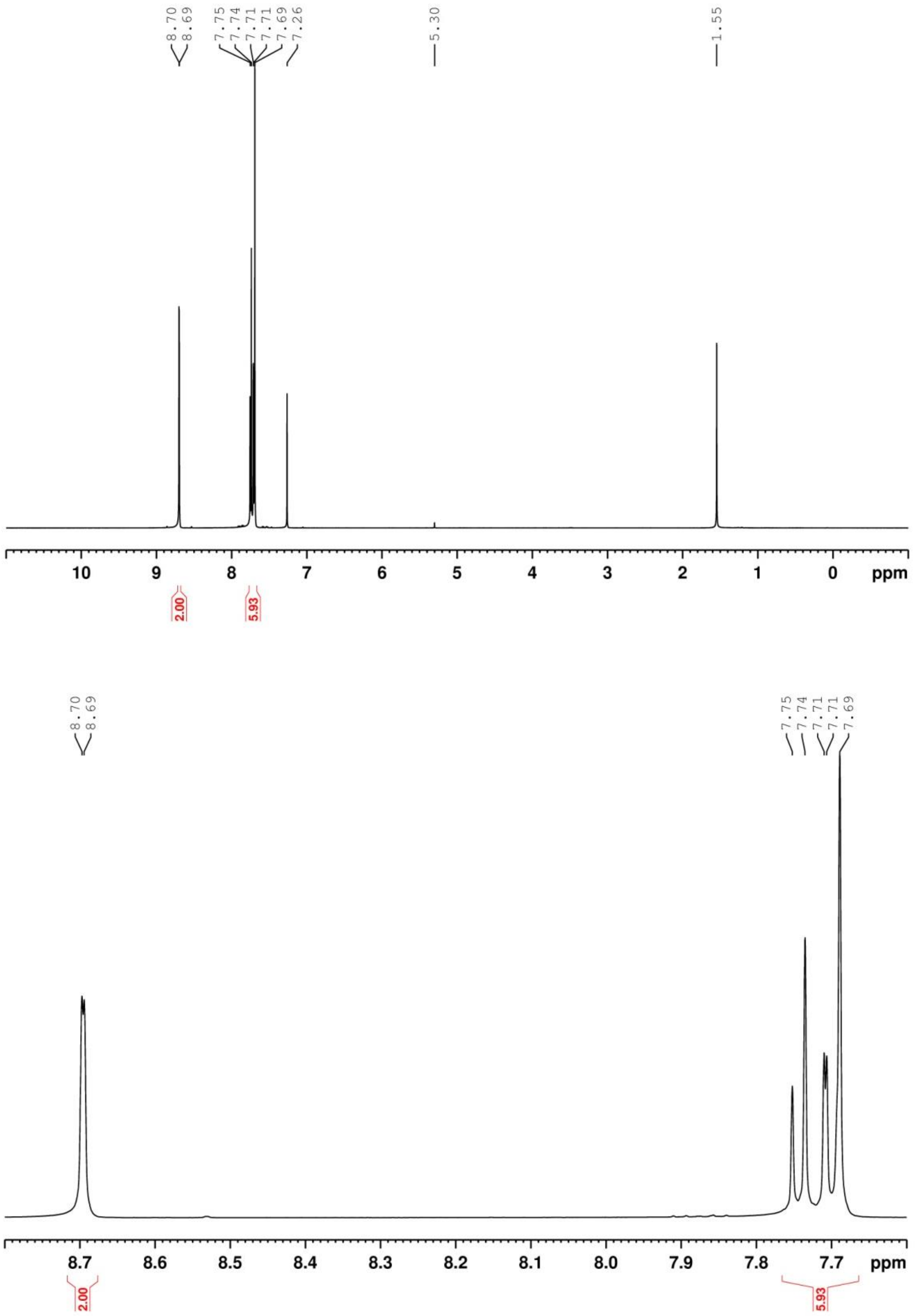

Figure S35 – ^{1}H NMR spectrum of compound **5** (500 MHz, $CDCl_3$, 298 K).

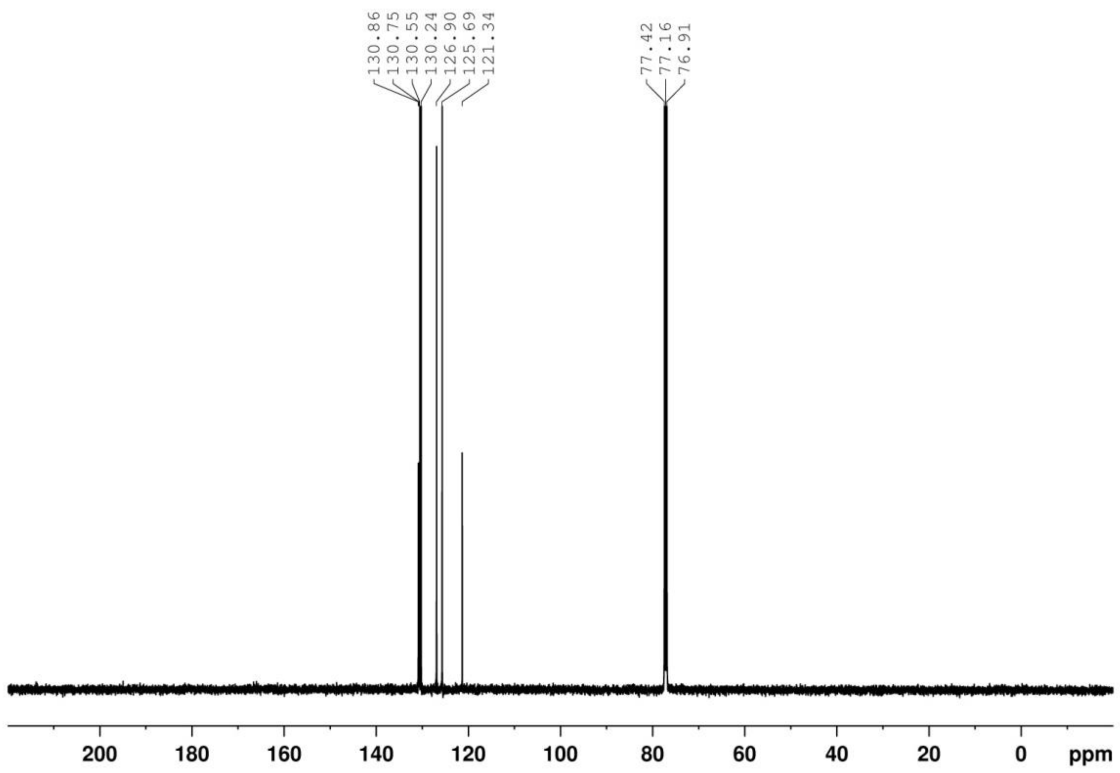

Figure S36 – ^{13}C NMR spectrum of compound **5** (125 MHz, $CDCl_3$, 298 K).

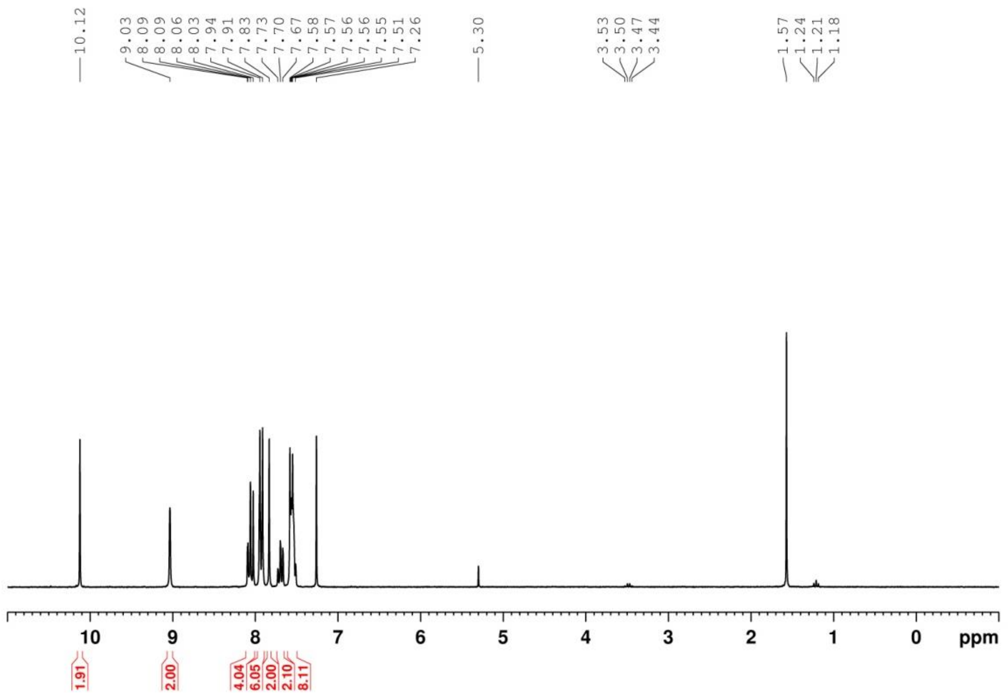

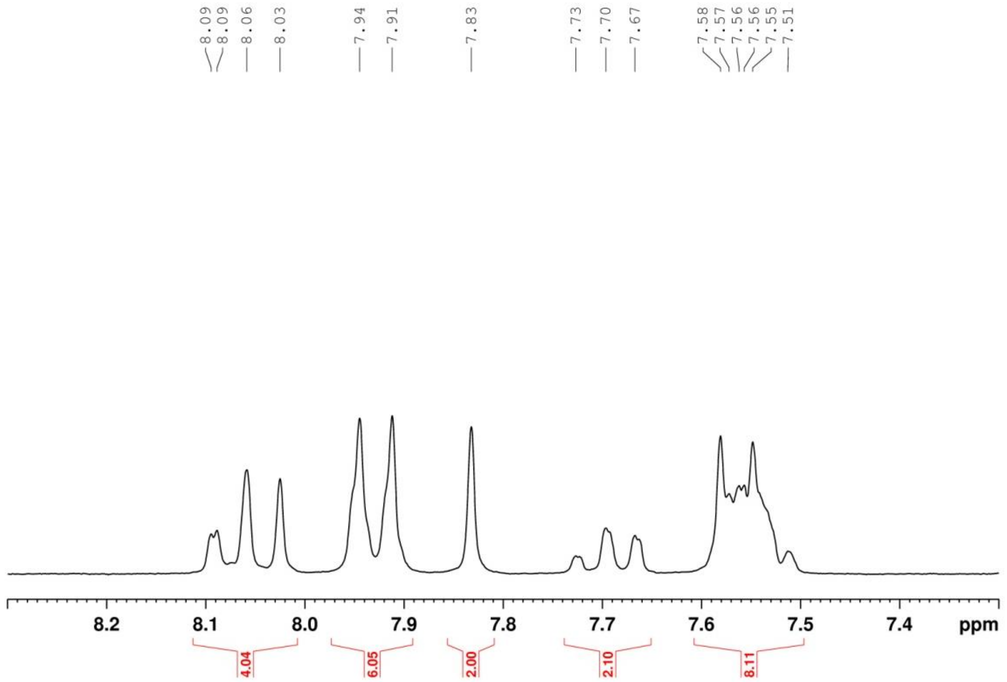

Figure S37 – ^{1}H NMR spectrum of compound **6** (250 MHz, $CDCl_3$, 298 K).

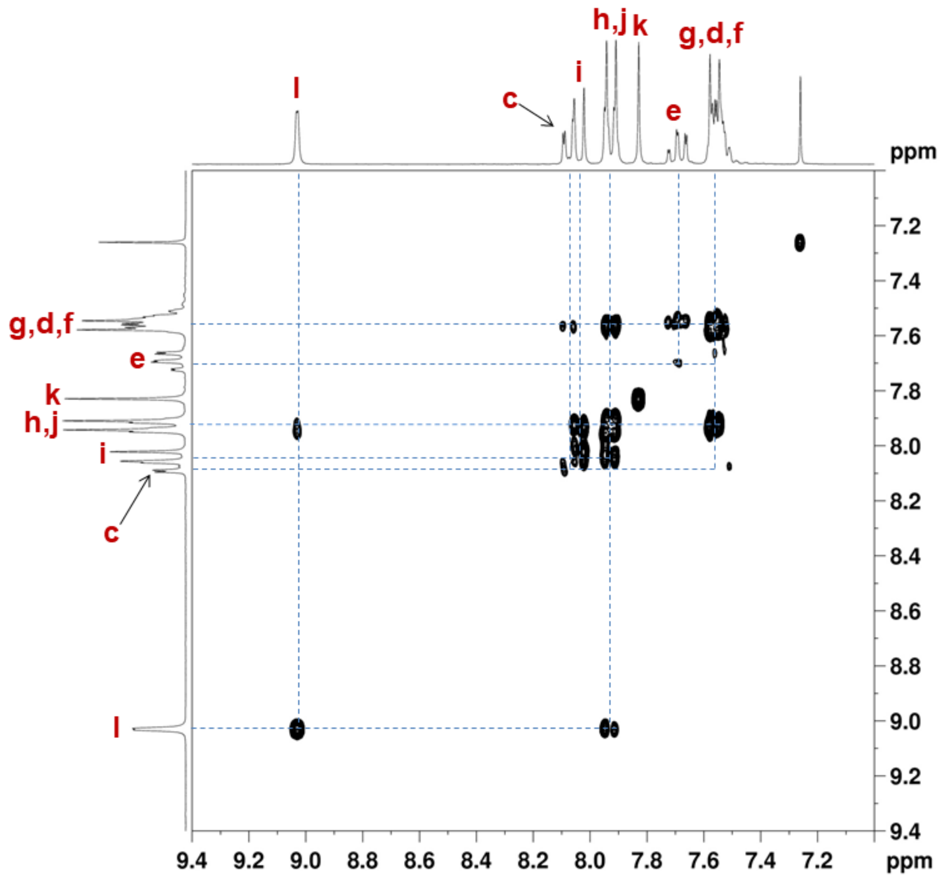

Figure S38 – Two-dimensional (^{1}H-^{1}H) COSY NMR spectrum of compound **6** (250 MHz, $CDCl_3$, 298 K).

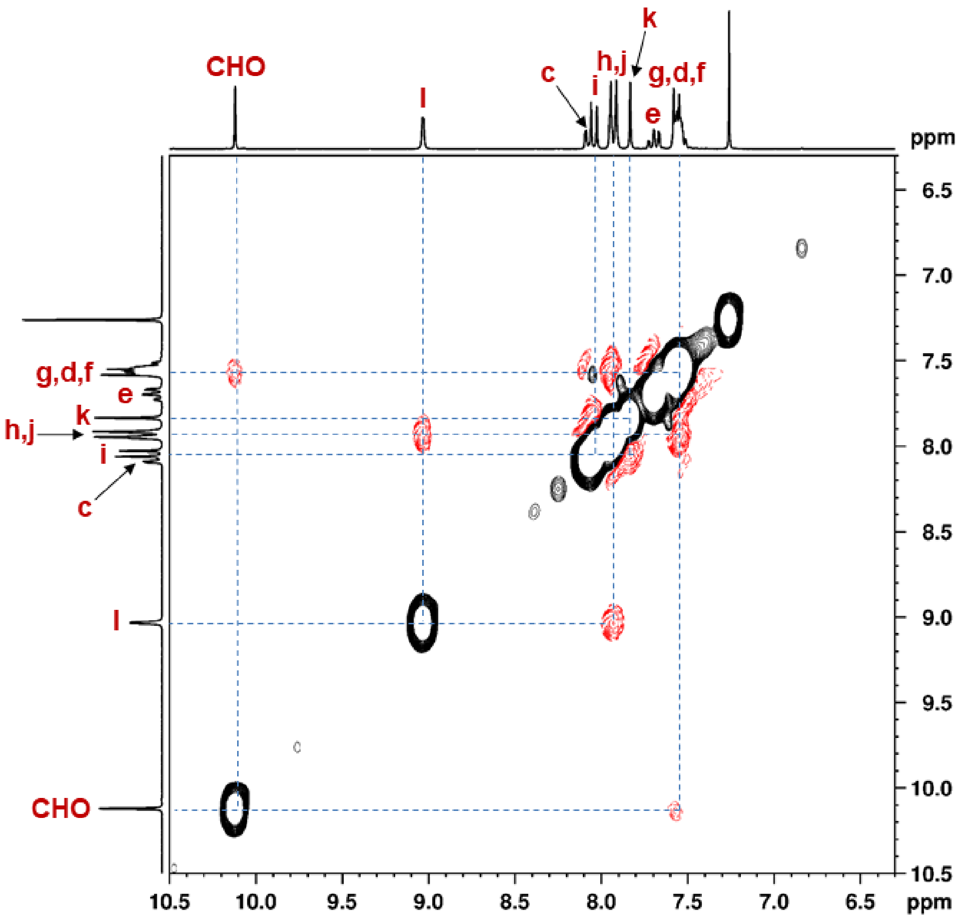

Figure S39 – Two-dimensional (^{1}H-^{1}H) NOESY NMR spectrum of compound **6** (250 MHz, $CDCl_3$, 298 K).

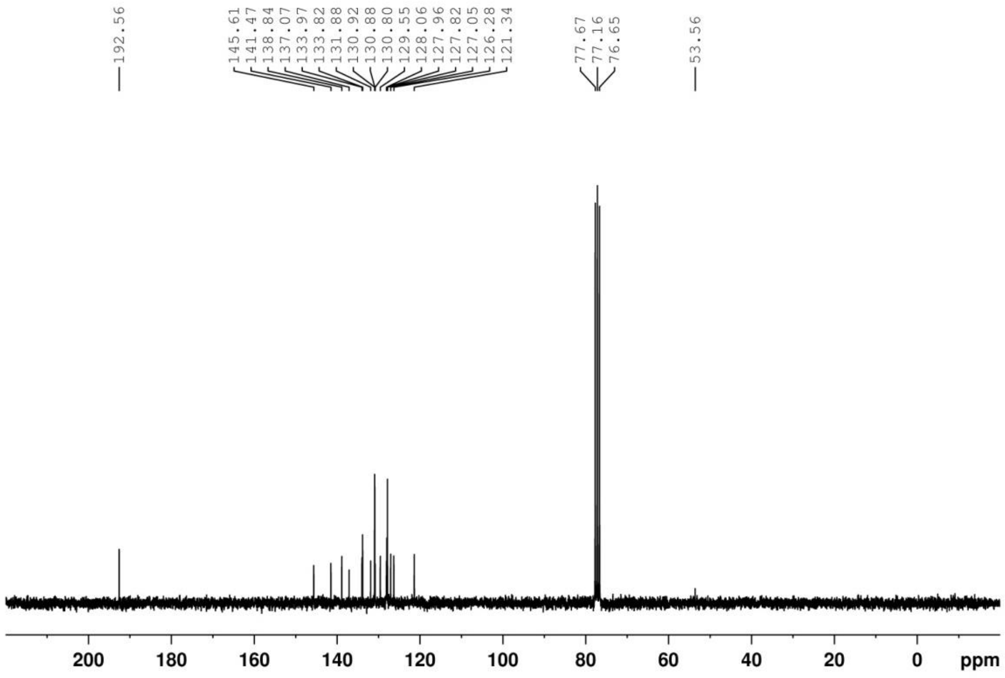

Figure S40 – ^{13}C NMR spectrum of compound **6** (60 MHz, $CDCl_3$, 298 K).

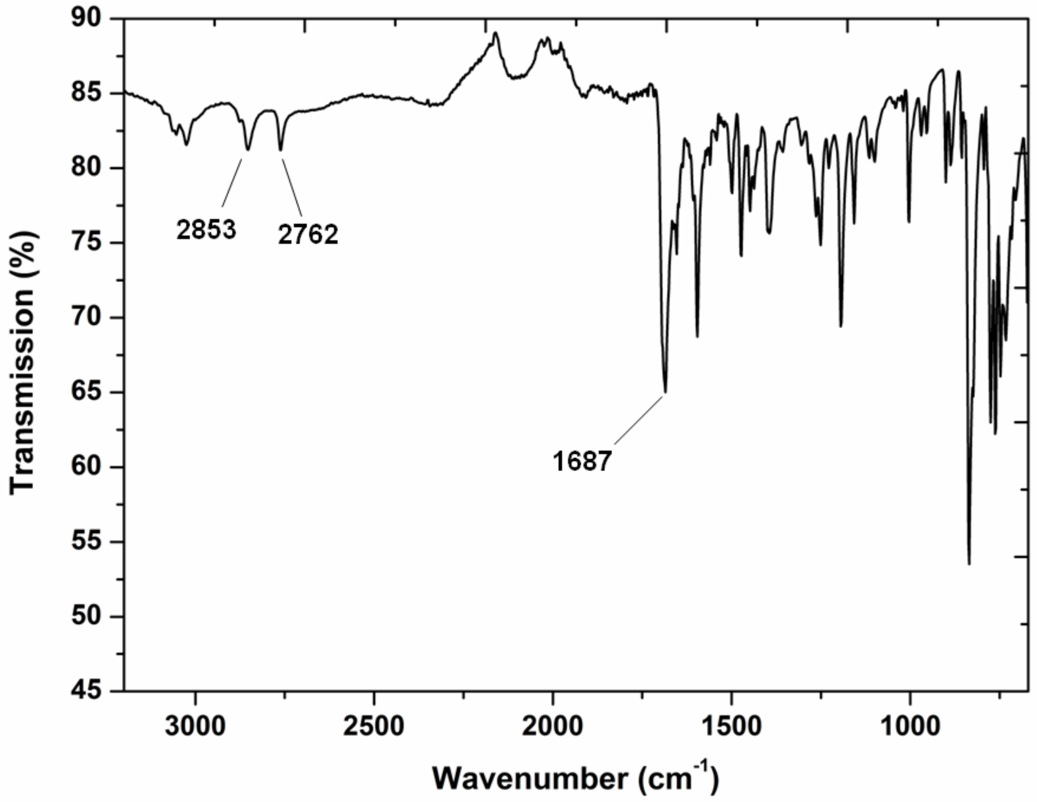

Figure S41 – FTIR spectrum of compound **6**.

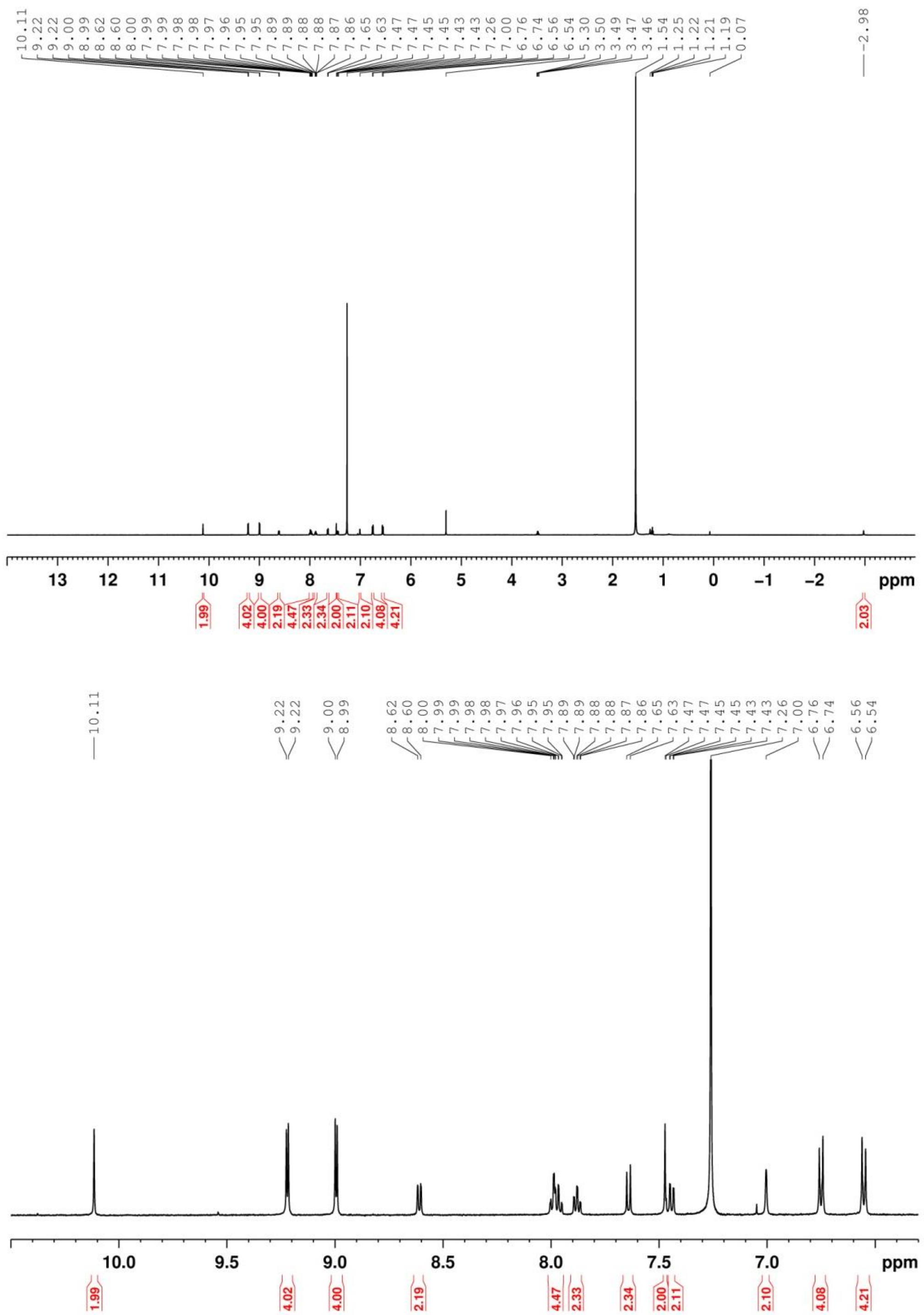

Figure S42 – ^{1}H NMR spectrum of macrocycle **7** (500 MHz, $CDCl_3$, 298 K).

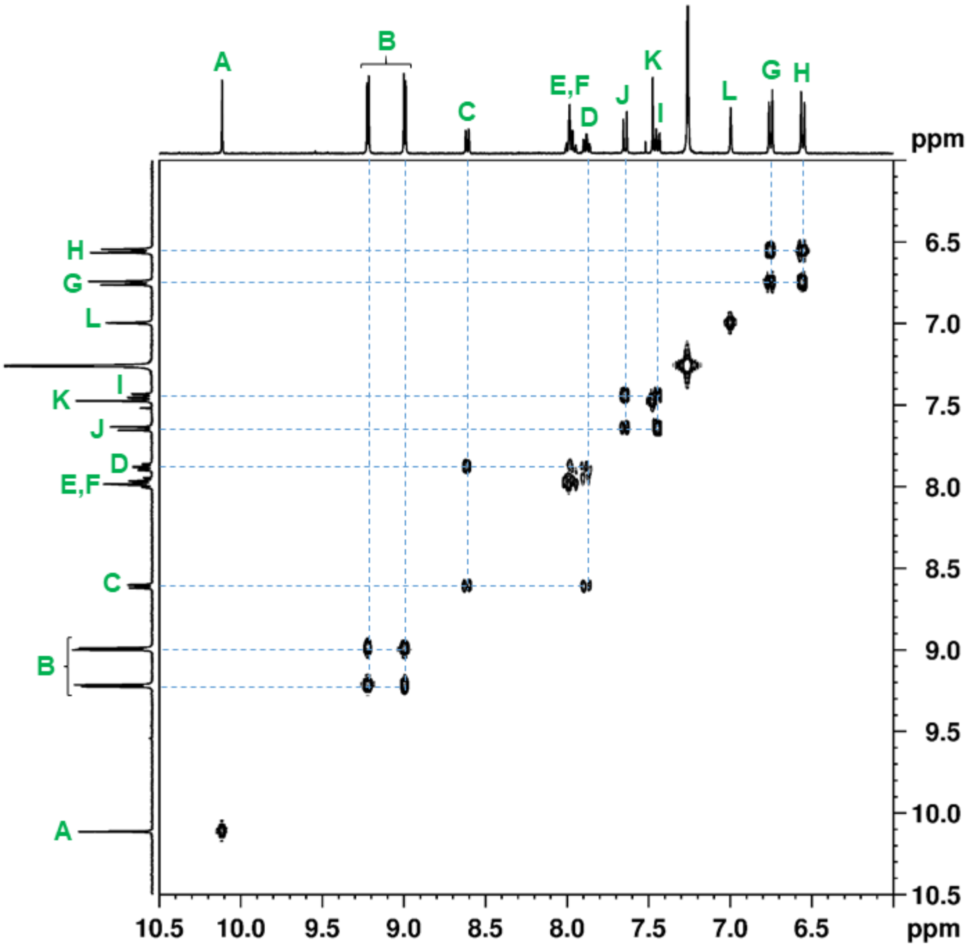

Figure S43 – Two-dimensional (^1H-^1H) COSY NMR spectrum of macrocycle **7** (500 MHz, $CDCl_3$, 298 K).

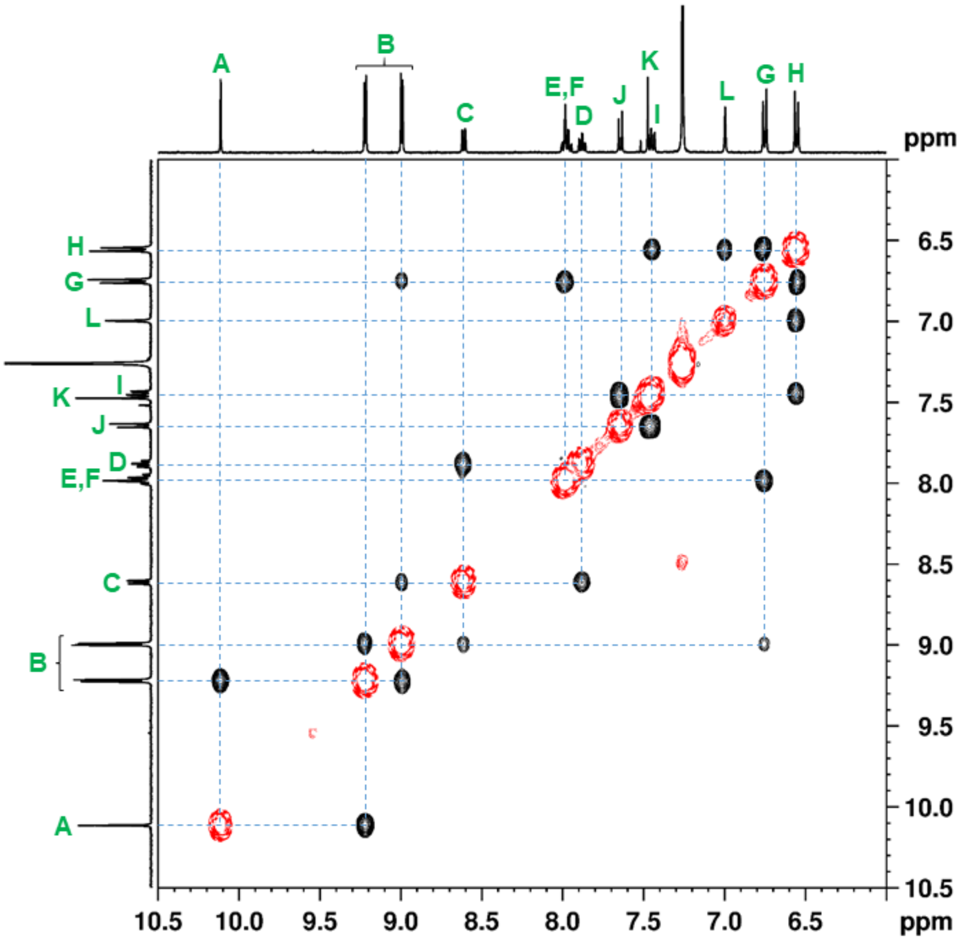

Figure S44 – Two-dimensional (^1H-^1H) NOESY NMR spectrum of macrocycle **7** (500 MHz, $CDCl_3$, 298 K).

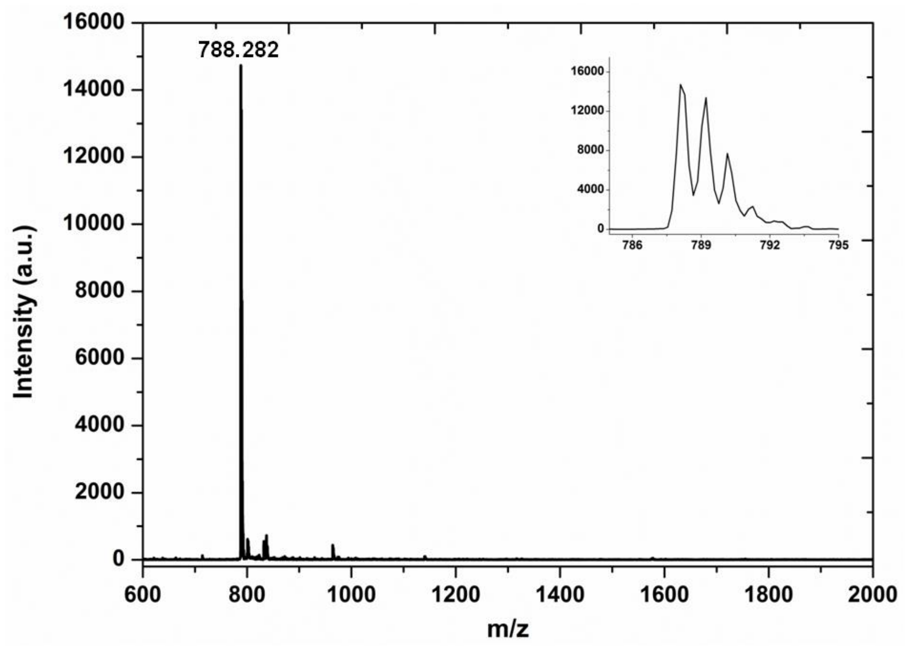

Figure S45 – MALDI-TOF mass spectrum of macrocycle **7**.

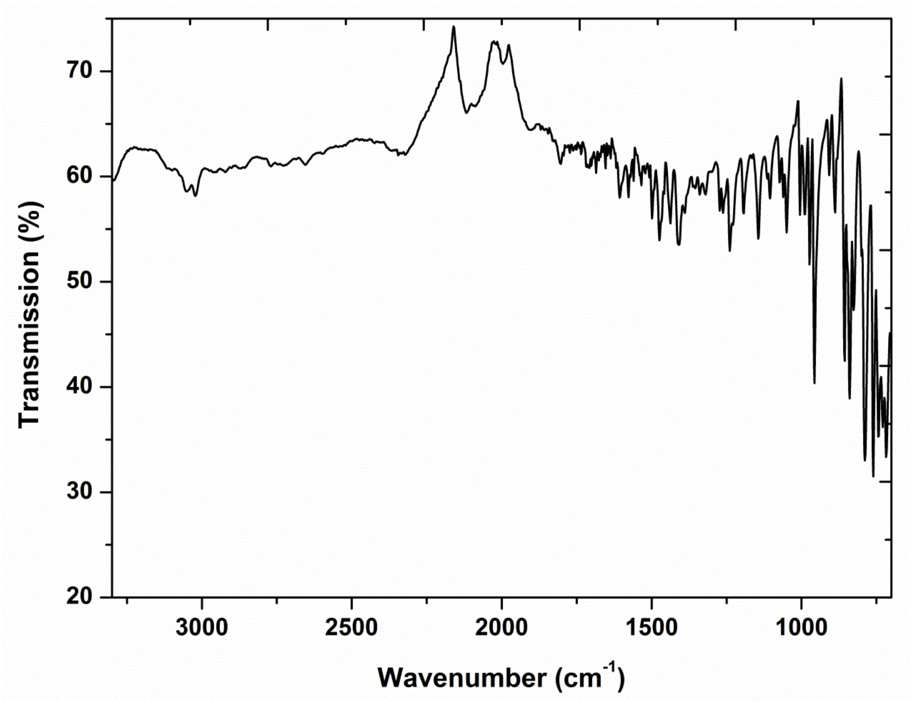

Figure S46 – FTIR spectrum of macrocycle **7**.

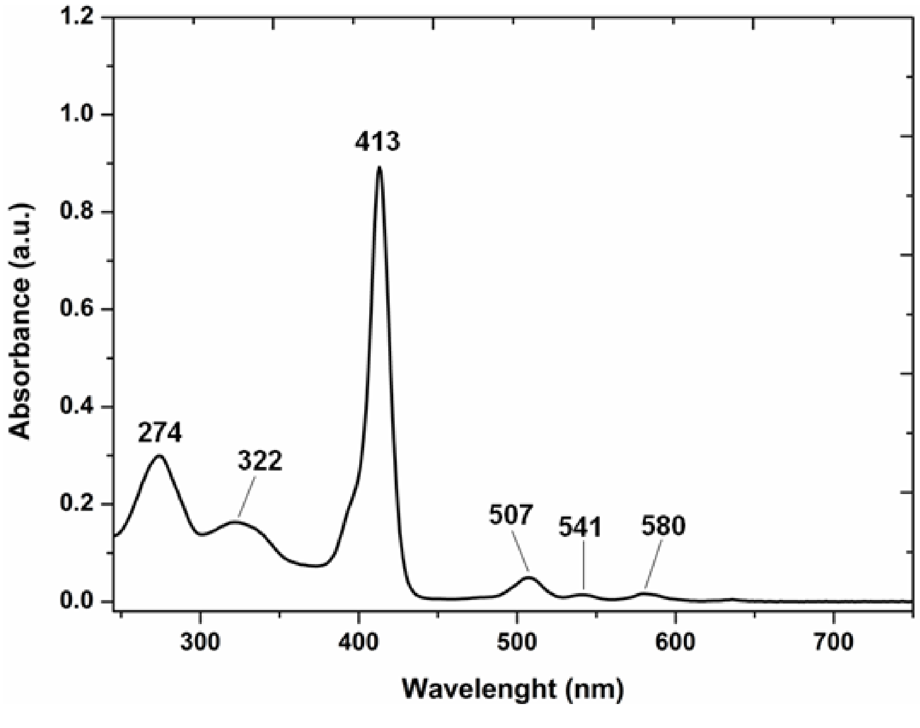

Figure S47 – Ground State UV-Vis absorption spectrum of macrocycle **7**. Conditions: DCM solution at 10^{-5} M concentration, room temperature.

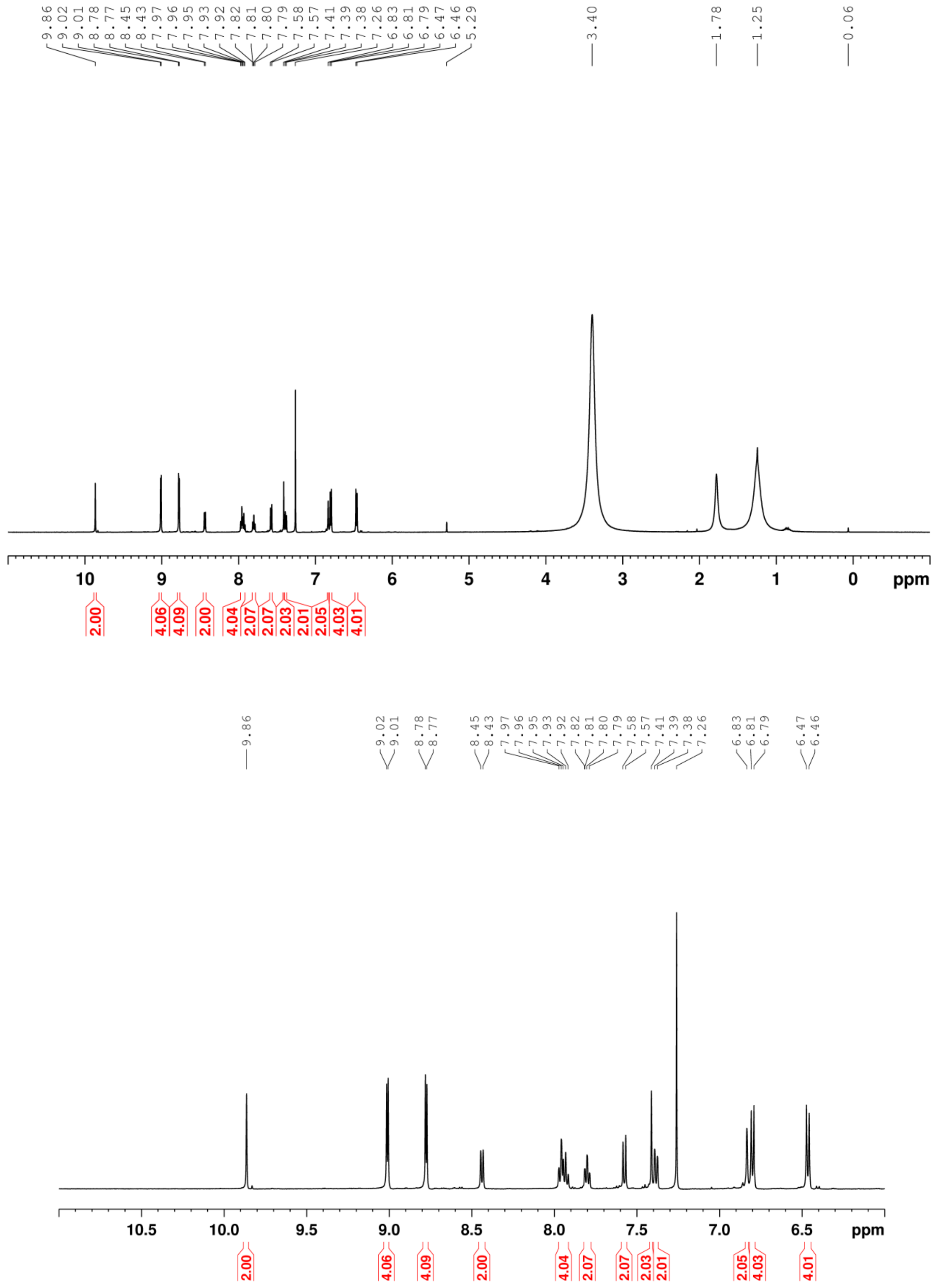

Figure S48 – ^{1}H NMR spectrum of macrocycle **8** (500 MHz, $CDCl_3$, 298 K).

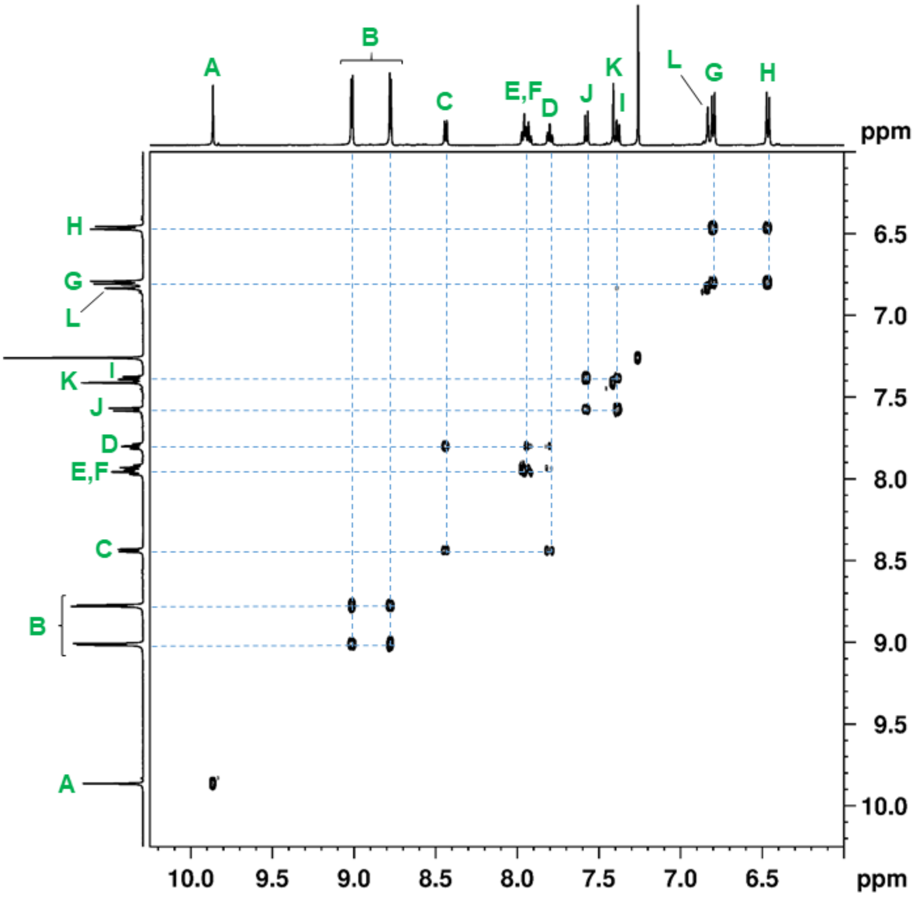

Figure S49 – Two-dimensional (^{1}H-^{1}H) COSY NMR spectrum of macrocycle **8** (500 MHz, $CDCl_3$, 298 K).

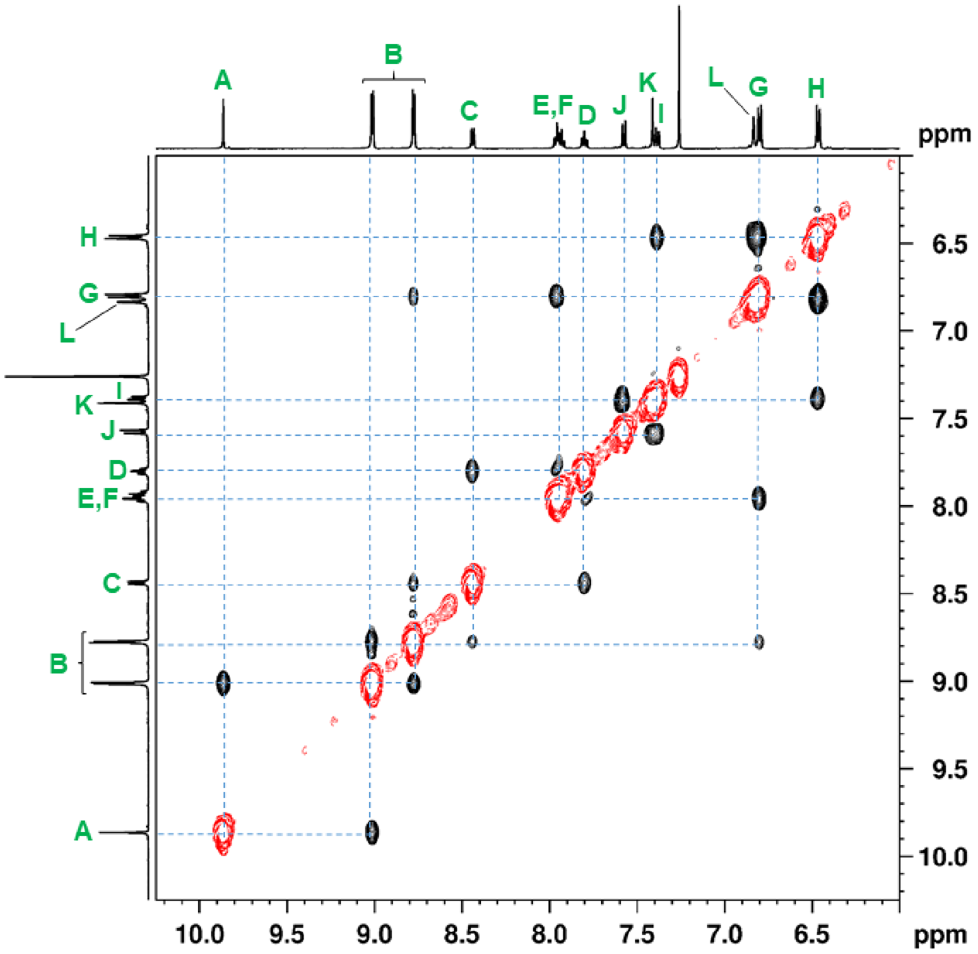

Figure S50 – Two-dimensional (^1H-^1H) NOESY NMR spectrum of macrocycle **8** (500 MHz, $CDCl_3$, 298 K).

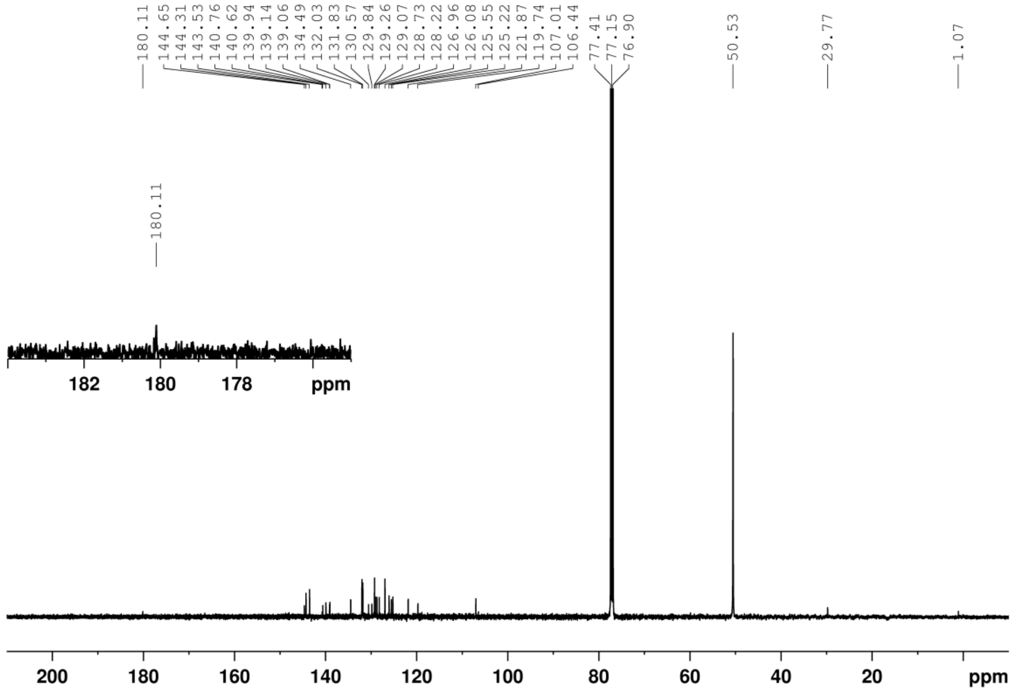

Figure S51 – ^{13}C NMR spectrum of macrocycle **8** (125 MHz, $CDCl_3$, 298 K).

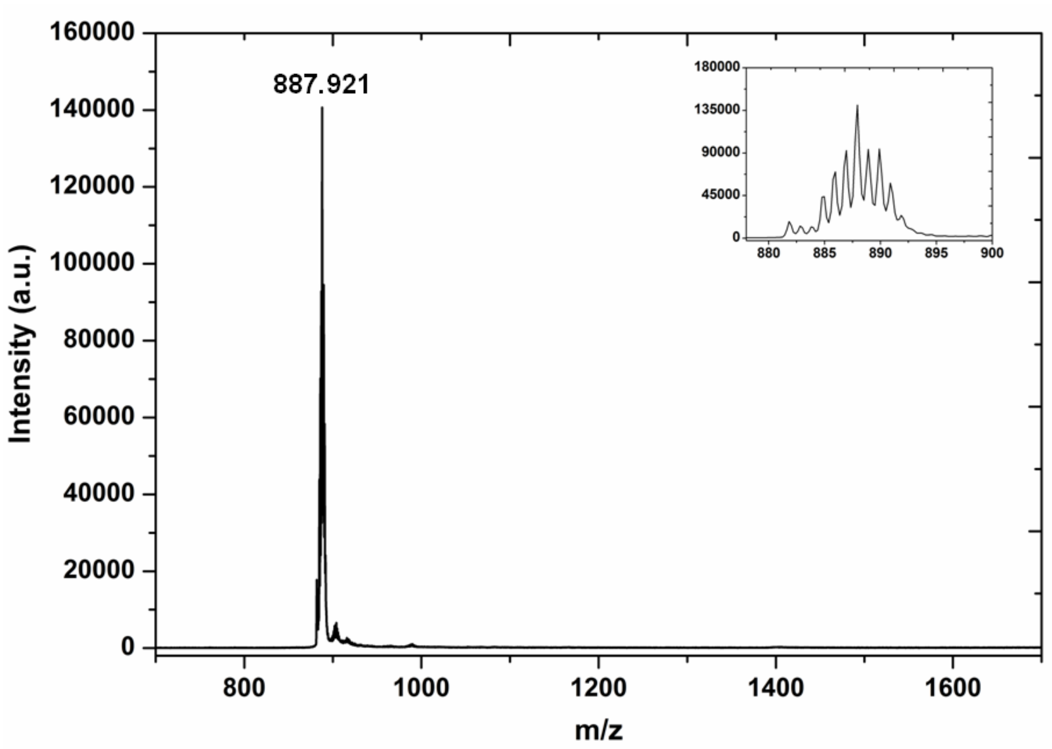

Figure S52 – MALDI-TOF mass spectrum of macrocycle **8**.

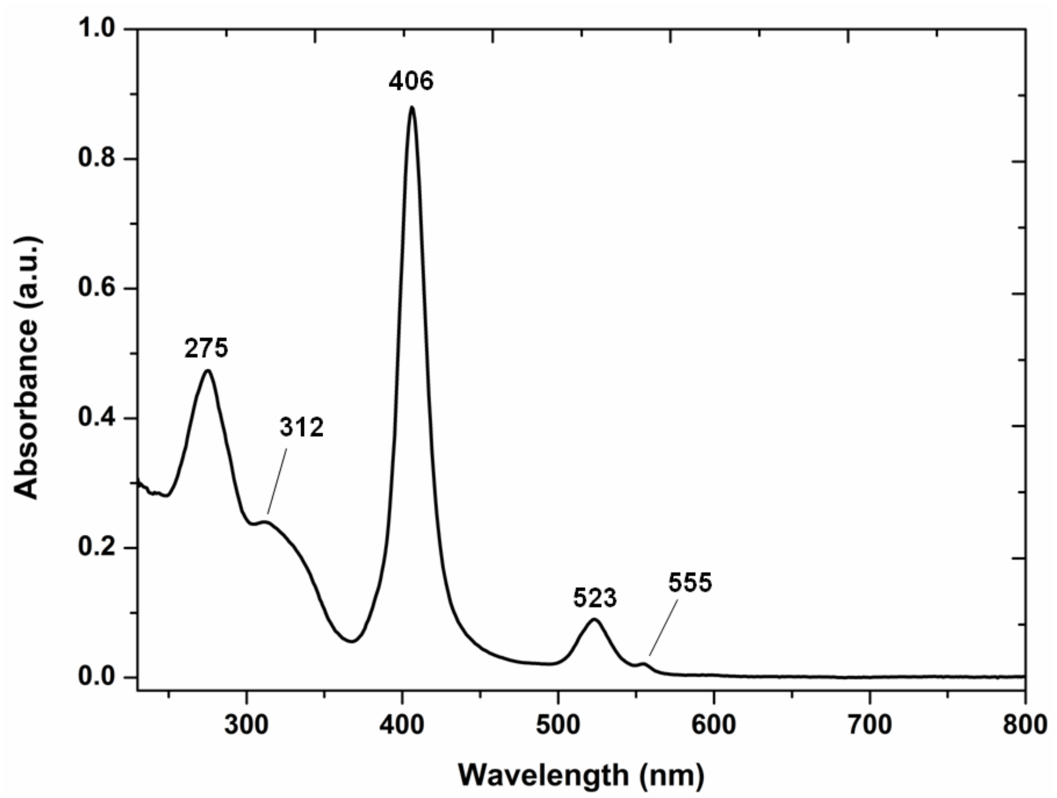

Figure S53 – Ground State UV-Vis absorption spectrum of macrocycle **8**. Conditions: DCM solutions at 10^{-5} M concentration, room temperature.

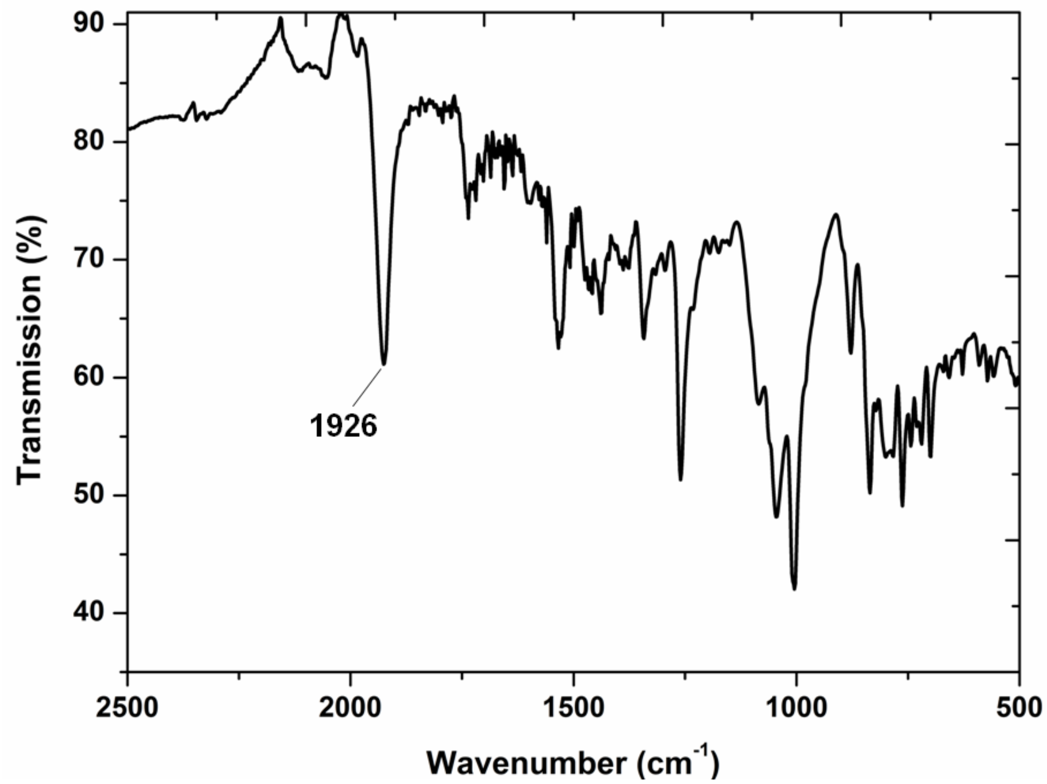

Figure S54 – FTIR spectrum of macrocycle **8**.

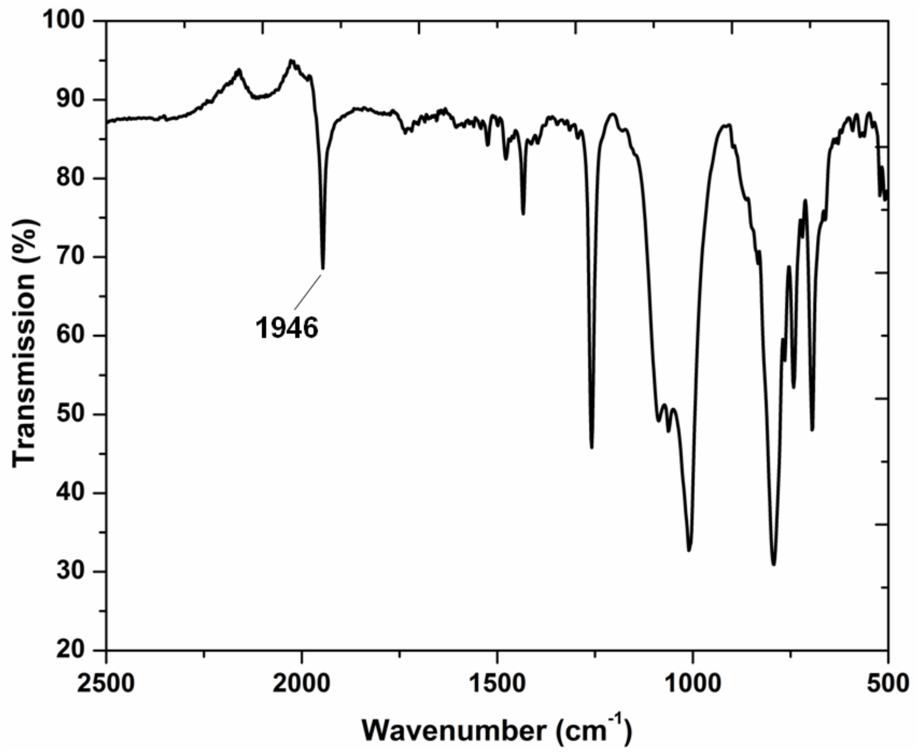

Figure S55 – Carbonyl stretching frequency region of the FTIR-ATR spectrum of complex **10**.

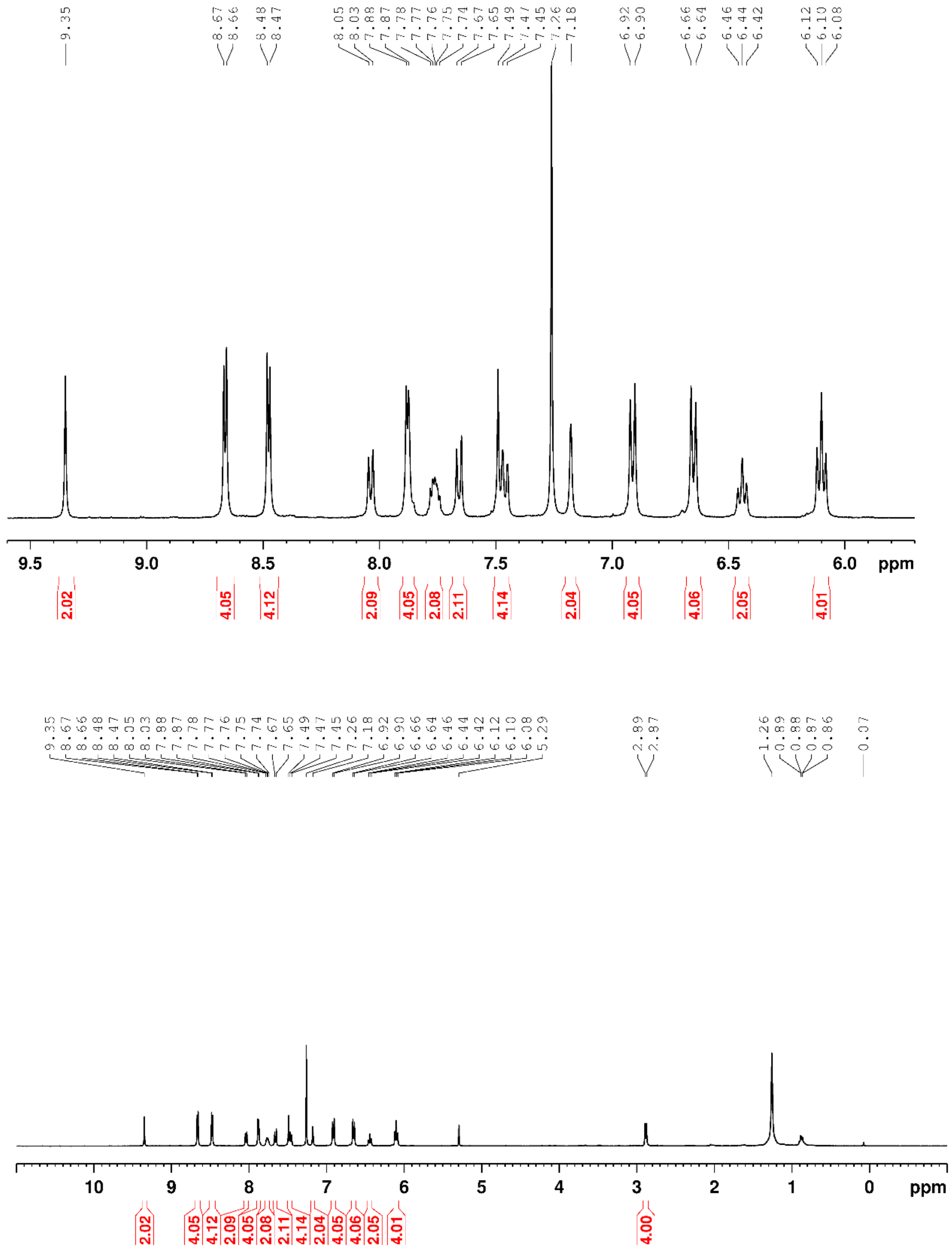

Figure S56 – ^{1}H NMR spectra of macrocycle **11** (400 MHz, $CDCl_3$, 298 K).

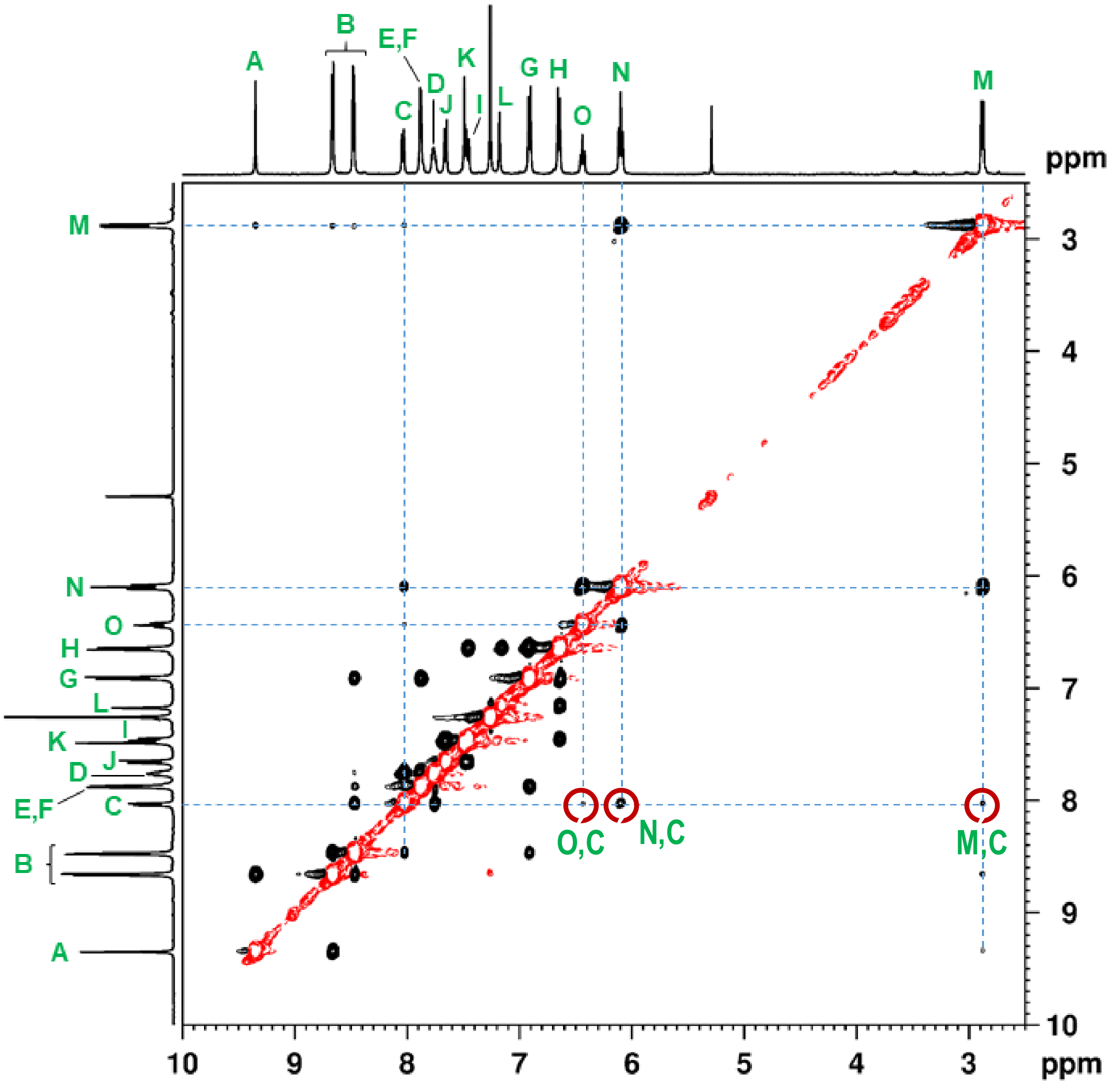

Figure S57 – Two-dimensional (^{1}H-^{1}H) NOESY NMR spectrum of macrocycle **11** recorded in $CDCl_3$ at 298 K (400 MHz). Proton labelling corresponds to that shown in Figure 5 of the manuscript. The dashed lines designate spatial cross signals between protons $H_{M\text{-}O}$ on the axial ligand and H_C on the phenyl rings at the *5,15-meso* positions of the porphyrin core, which confirm the axial coordination of the carbene ligand *exo*-to the macrocycle's cavity. Those findings provide unequivocal evidence for the effective steric bias of the aromatic appended loop in **8** against bulky diphenyldiazomethane to afford **11**.

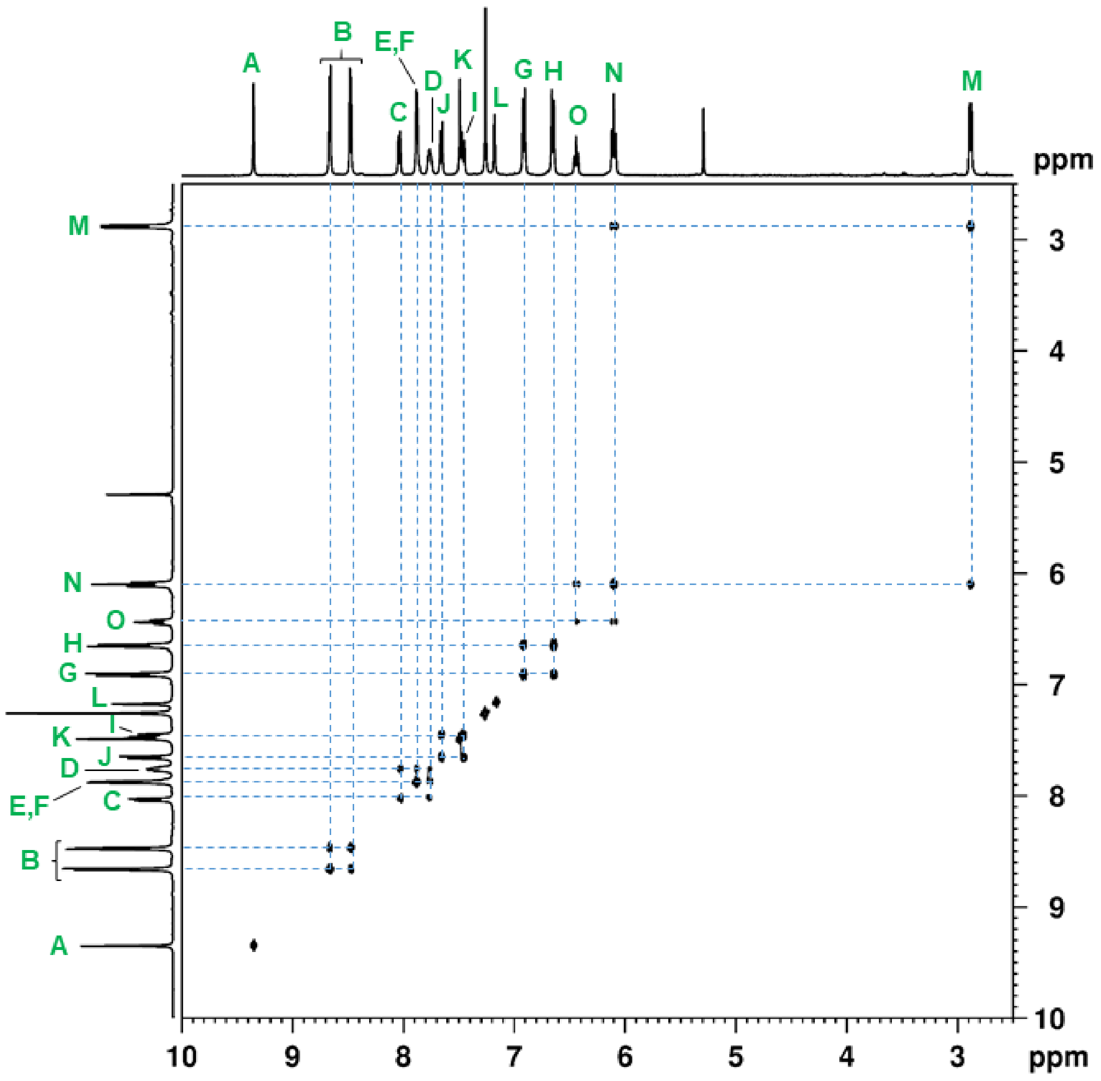

Figure S58 – Two-dimensional (^1H-^1H) COSY NMR spectrum of macrocycle **11** (400 MHz, $CDCl_3$, 298 K).

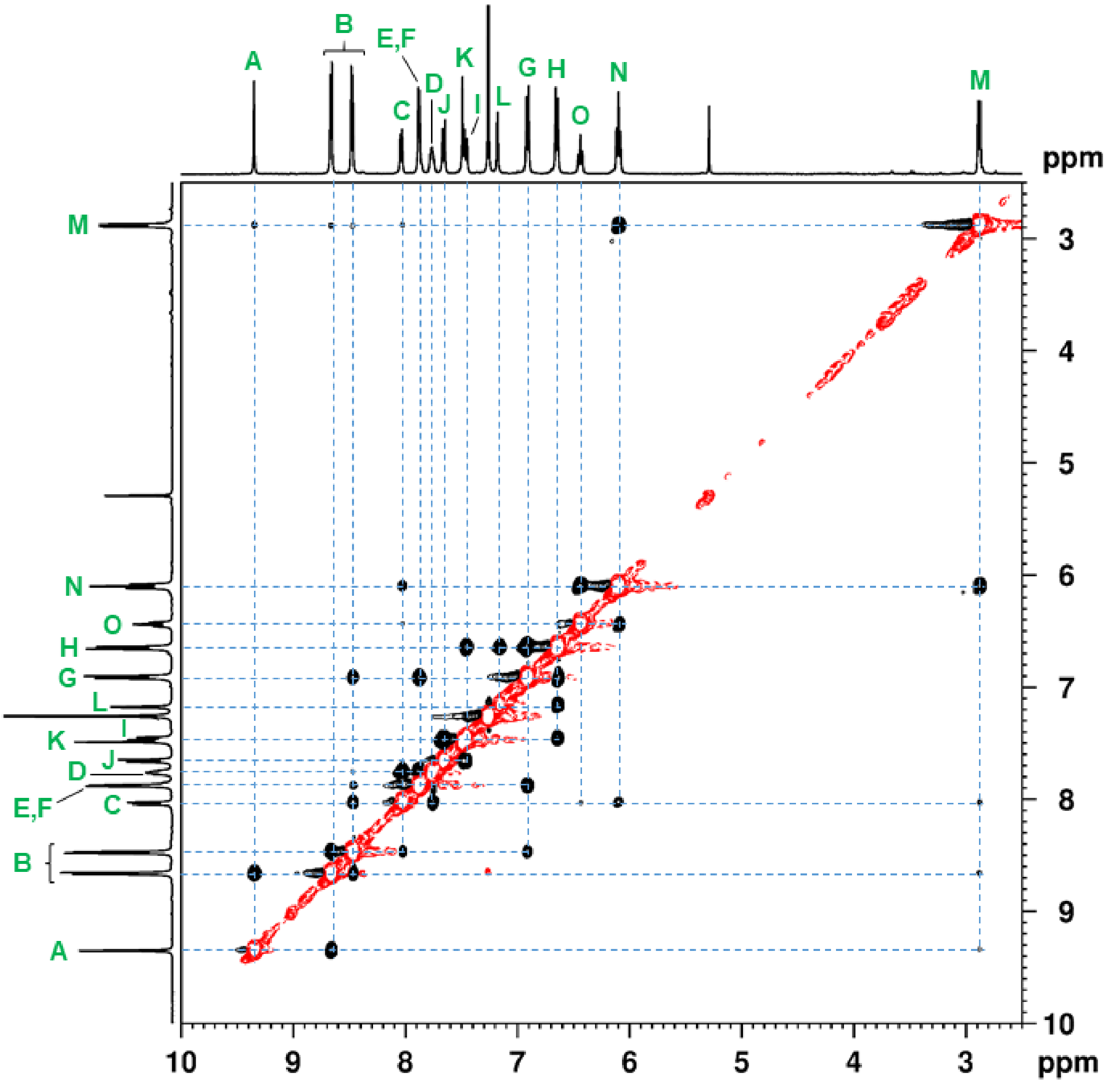

Figure S59 – Two-dimensional (^{1}H-^{1}H) NOESY NMR spectrum of macrocycle **11** (400 MHz, $CDCl_3$, 298 K).

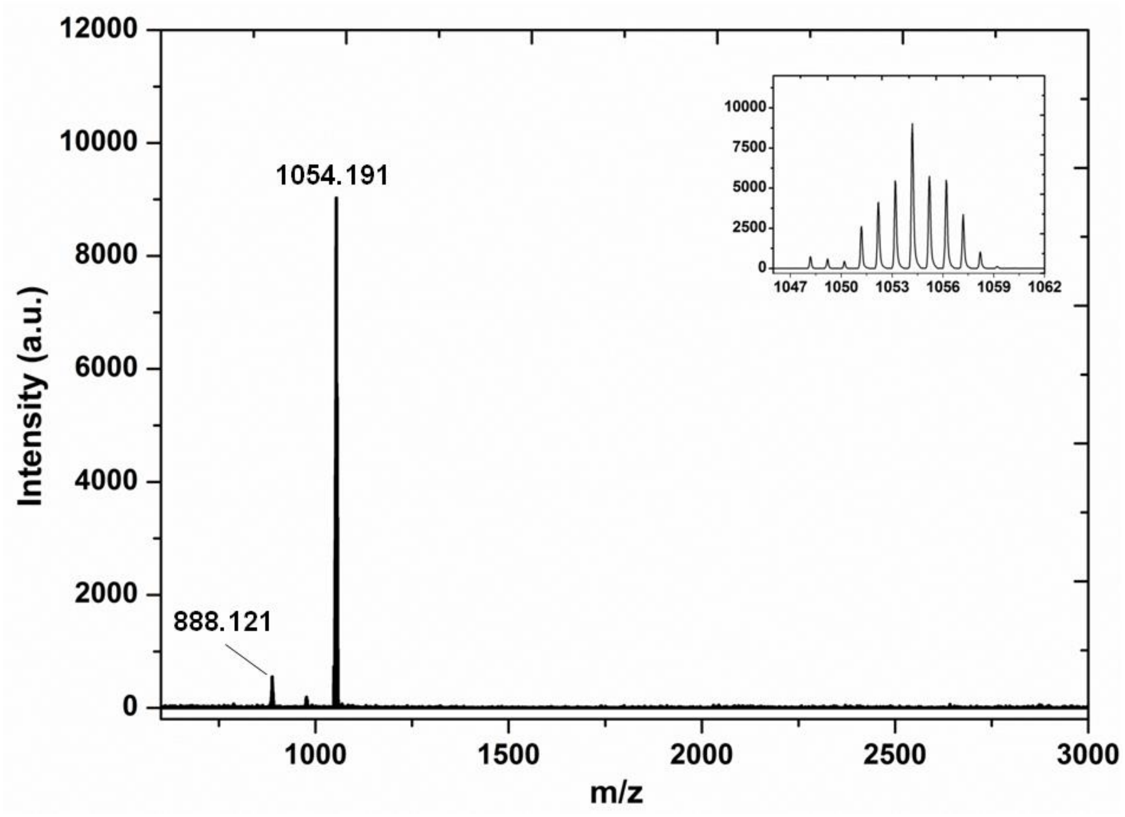

Figure S60 – MALDI-TOF mass spectrum of macrocycle **11**.

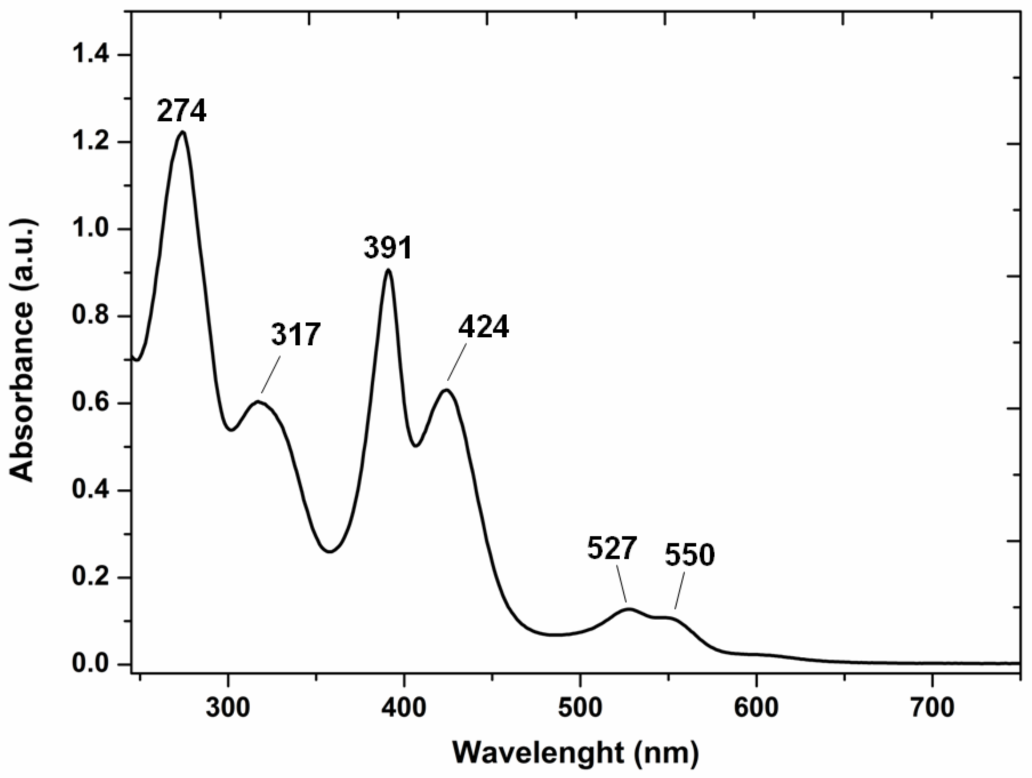

Figure S61 – Ground State UV-Vis absorption spectrum of macrocycle **11**. Conditions: DCM solutions at 10^{-5} M concentration, room temperature.

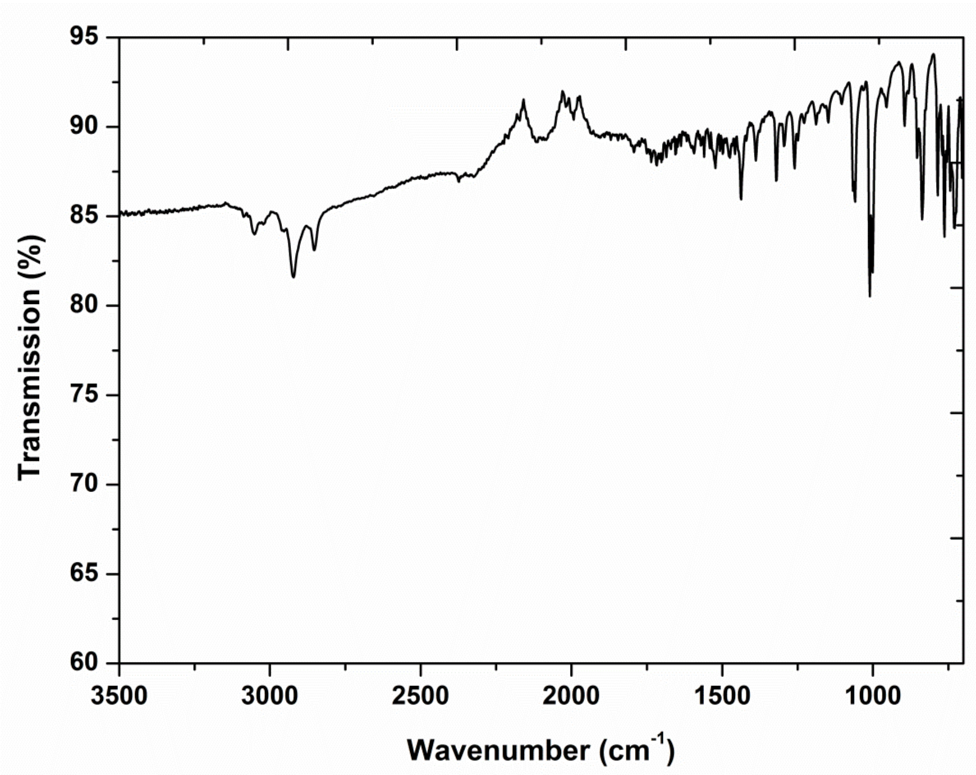

Figure S62 – FTIR spectrum of macrocycle **11**.

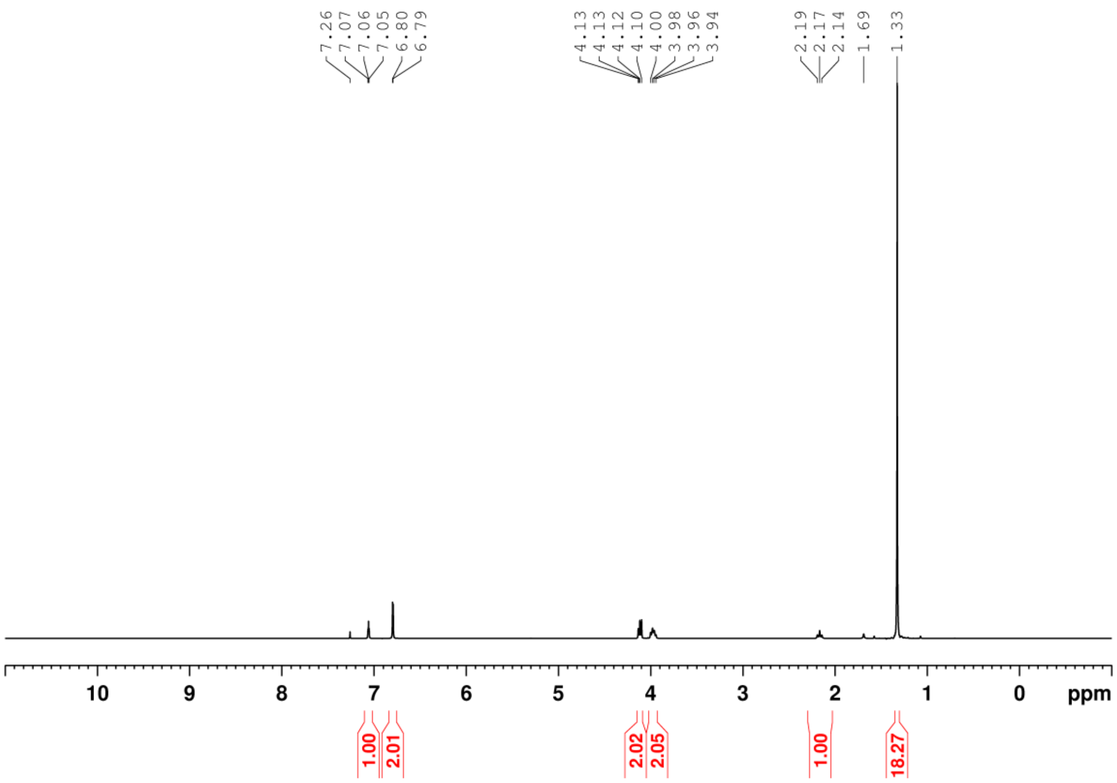

Figure S63 – ^{1}H NMR spectrum of compound **S2** (250 MHz, $CDCl_3$, 298 K).

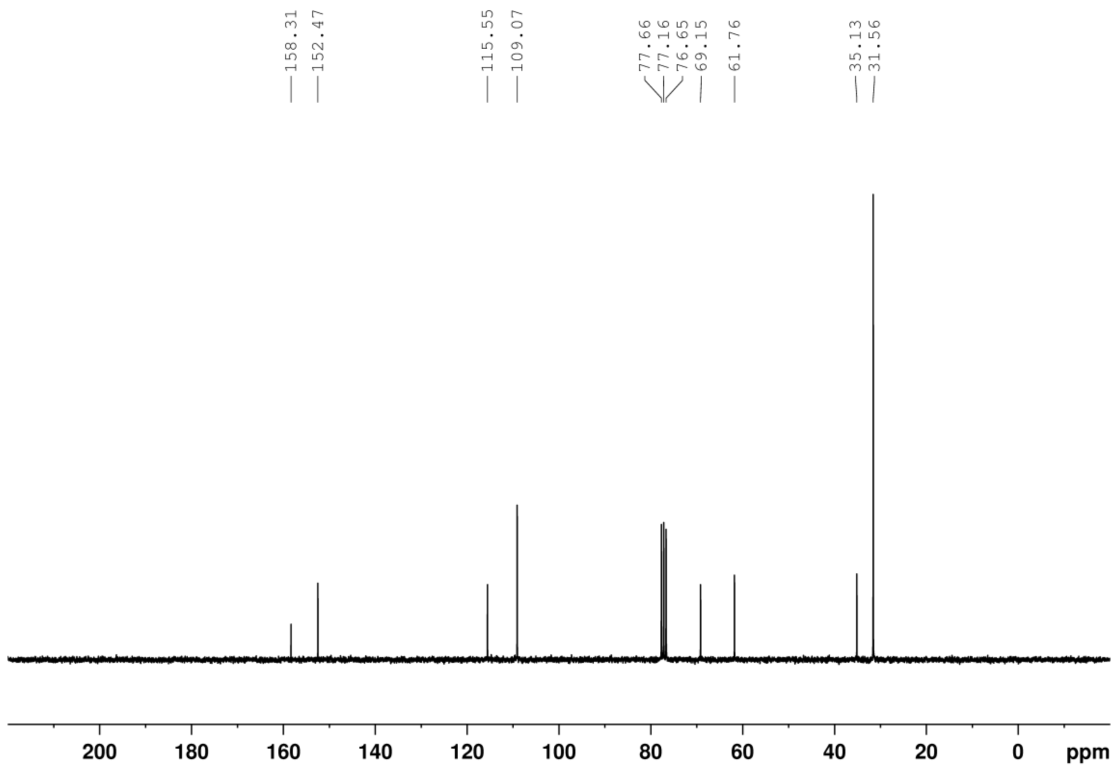

Figure S64 – ^{13}C NMR spectrum of compound **S2** (60 MHz, $CDCl_3$, 298 K).

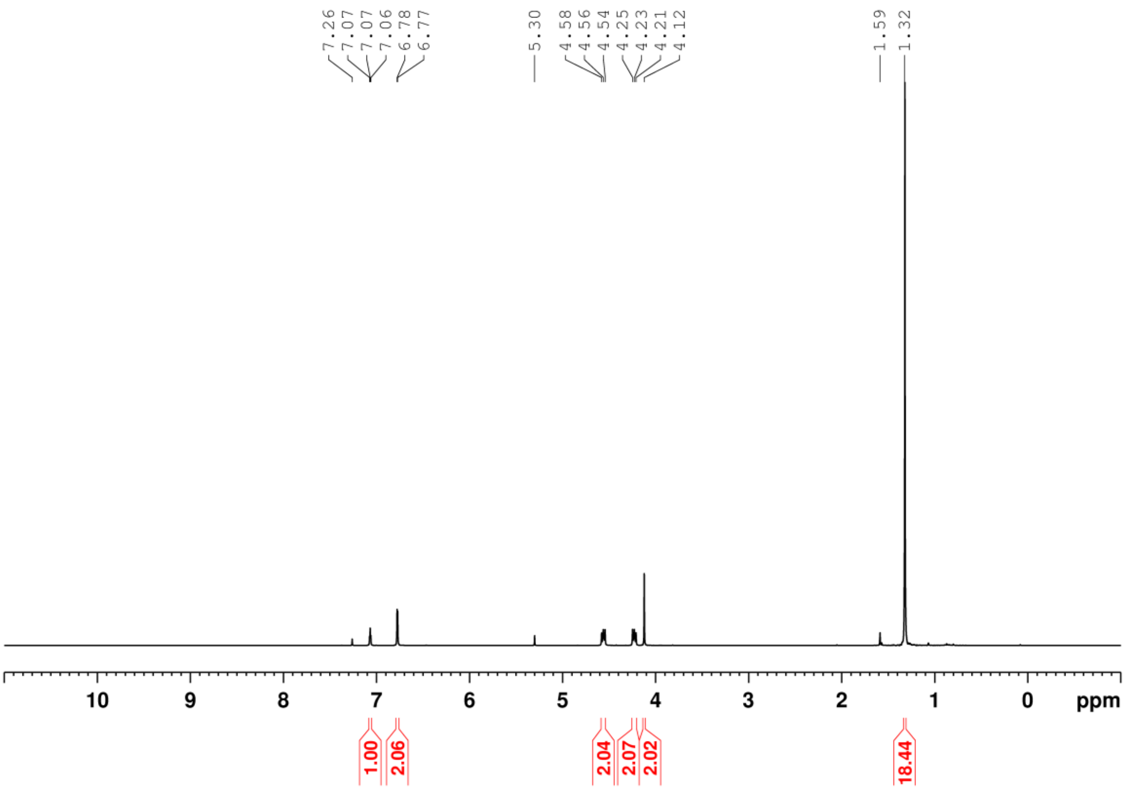

Figure S65 – ^{1}H NMR spectrum of compound **S3** (250 MHz, $CDCl_3$, 298 K).

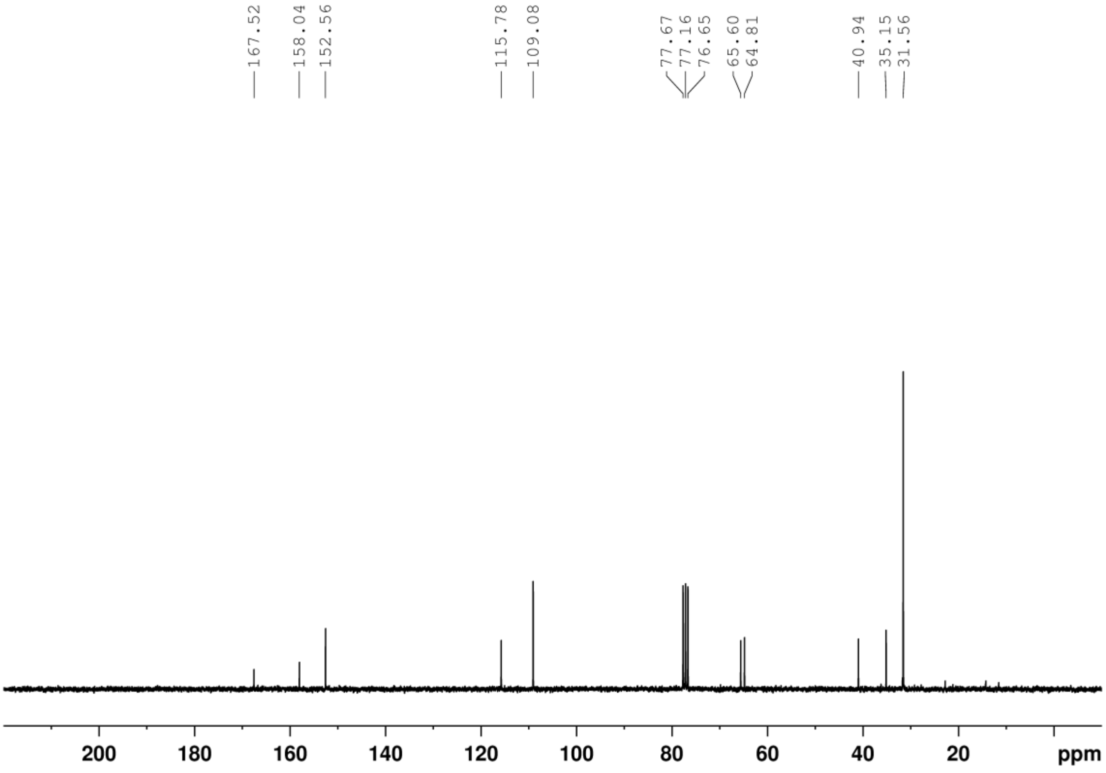

Figure S66 – ^{13}C NMR spectrum of compound **S3** (60 MHz, $CDCl_3$, 298 K).

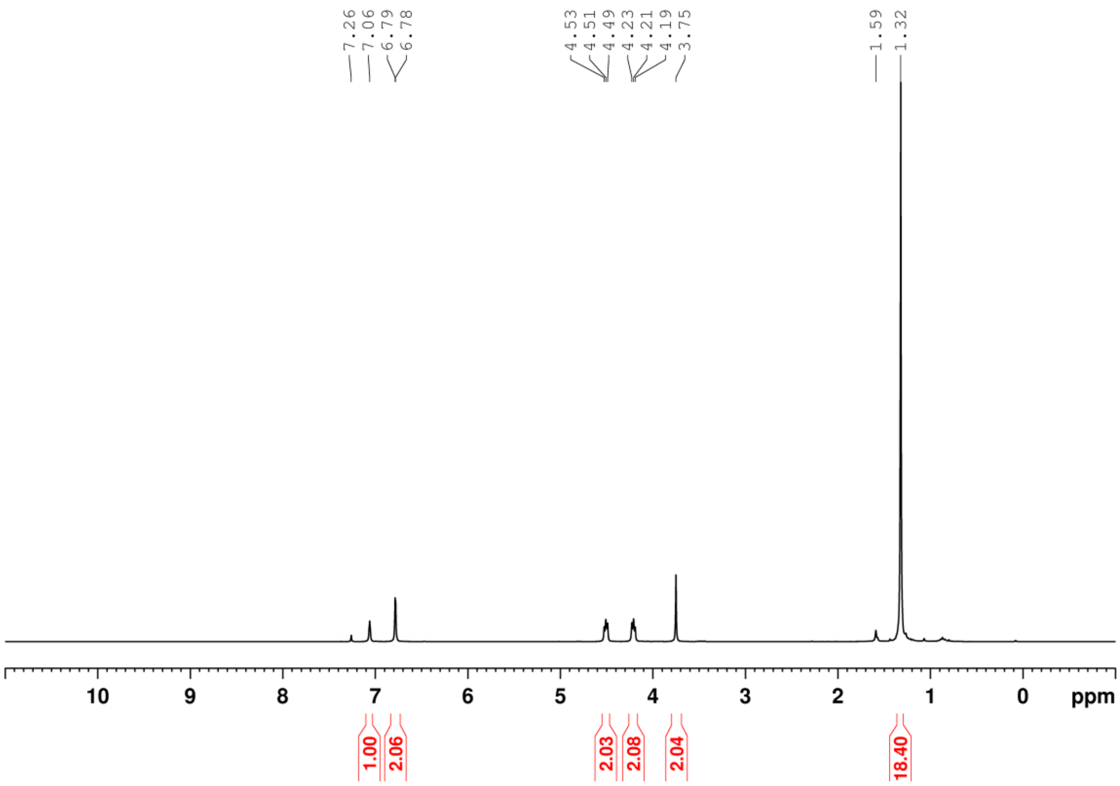

Figure S67 – ^{1}H NMR spectrum of compound **S4** (250 MHz, $CDCl_3$, 298 K).

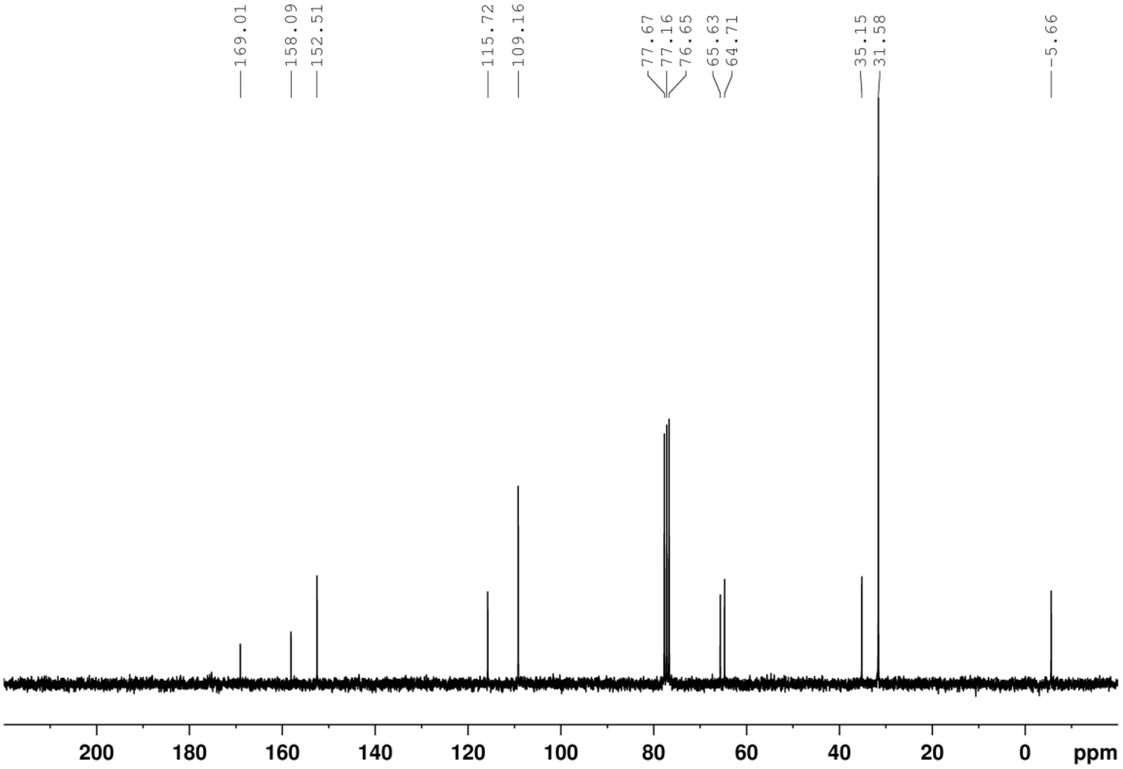

Figure S68 – ^{13}C NMR spectrum of compound **S4** (60 MHz, $CDCl_3$, 298 K).

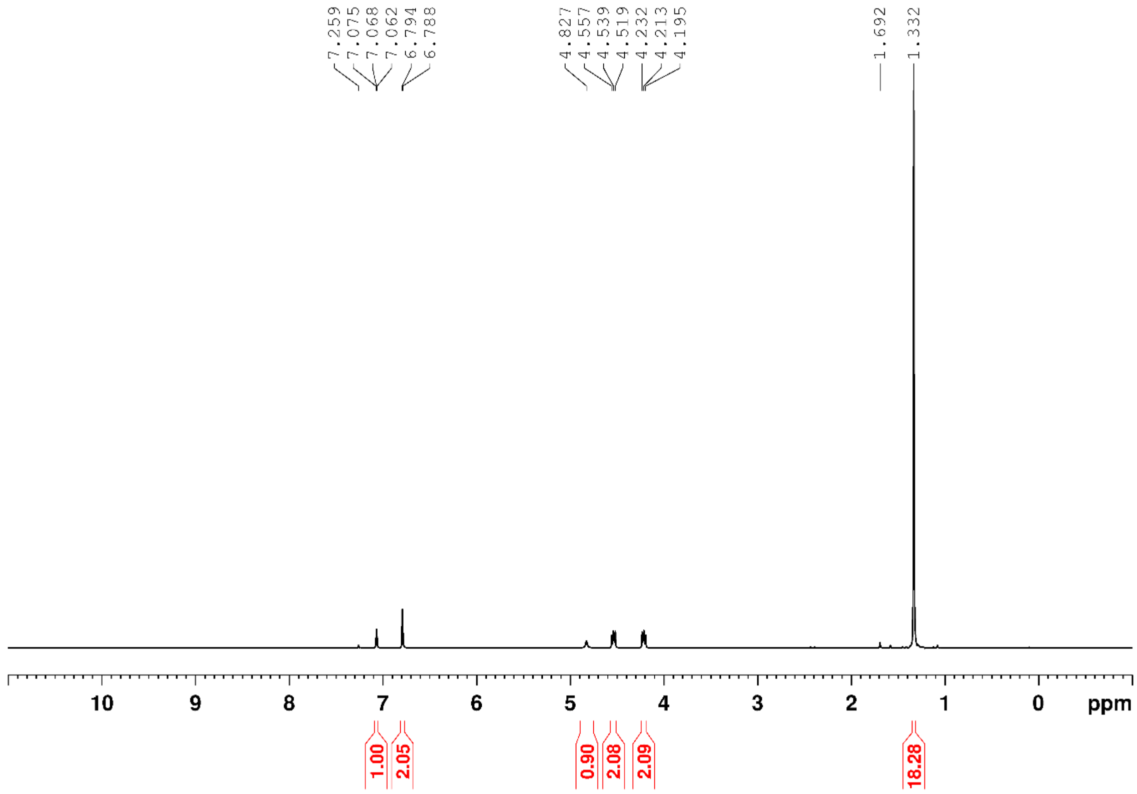

Figure S69 – ^{1}H NMR spectrum of compound **14** (250 MHz, $CDCl_3$, 298 K).

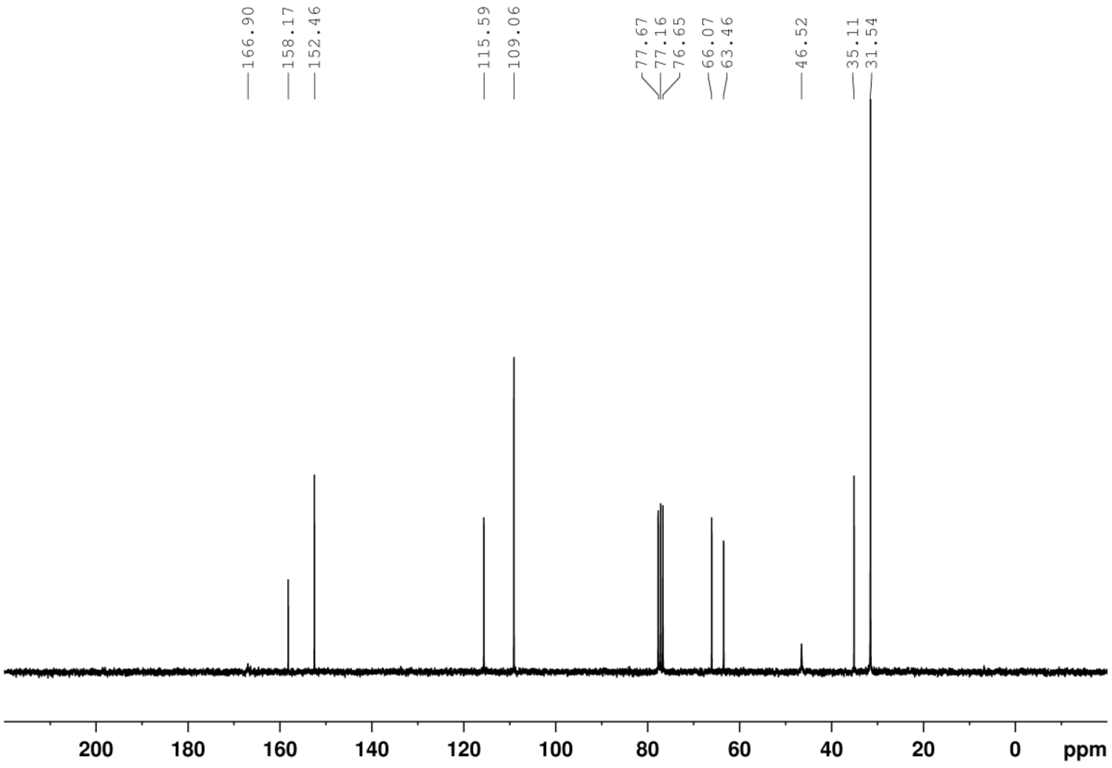

Figure S70 – ^{13}C NMR spectrum of compound **14** (60 MHz, $CDCl_3$, 298 K).

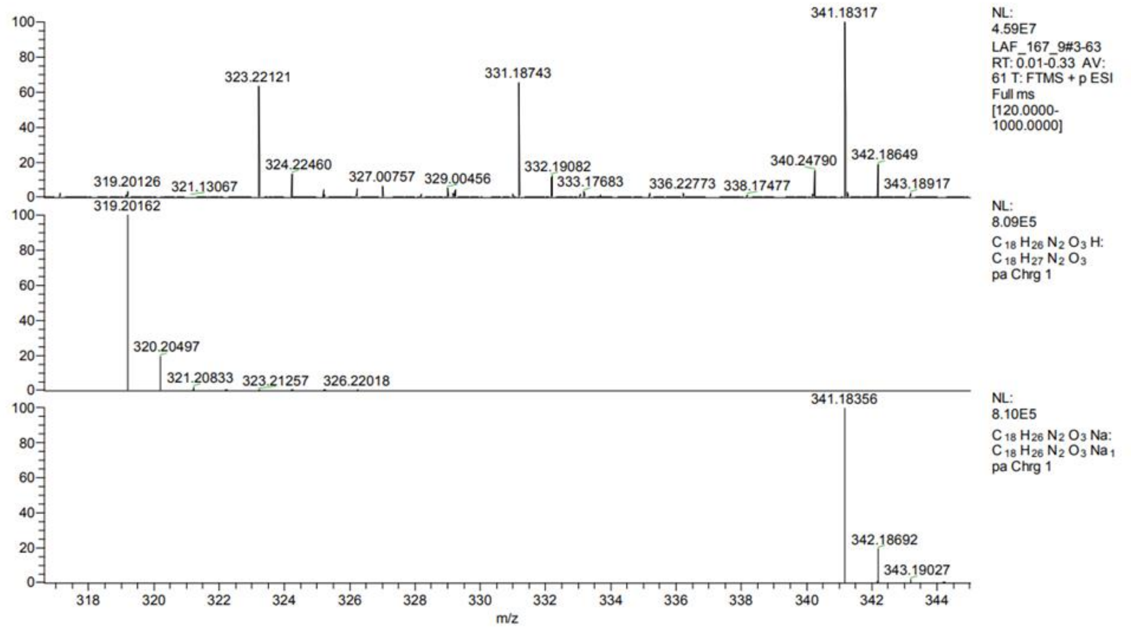

Figure S71 – High-resolution mass spectrum of compound **14**.

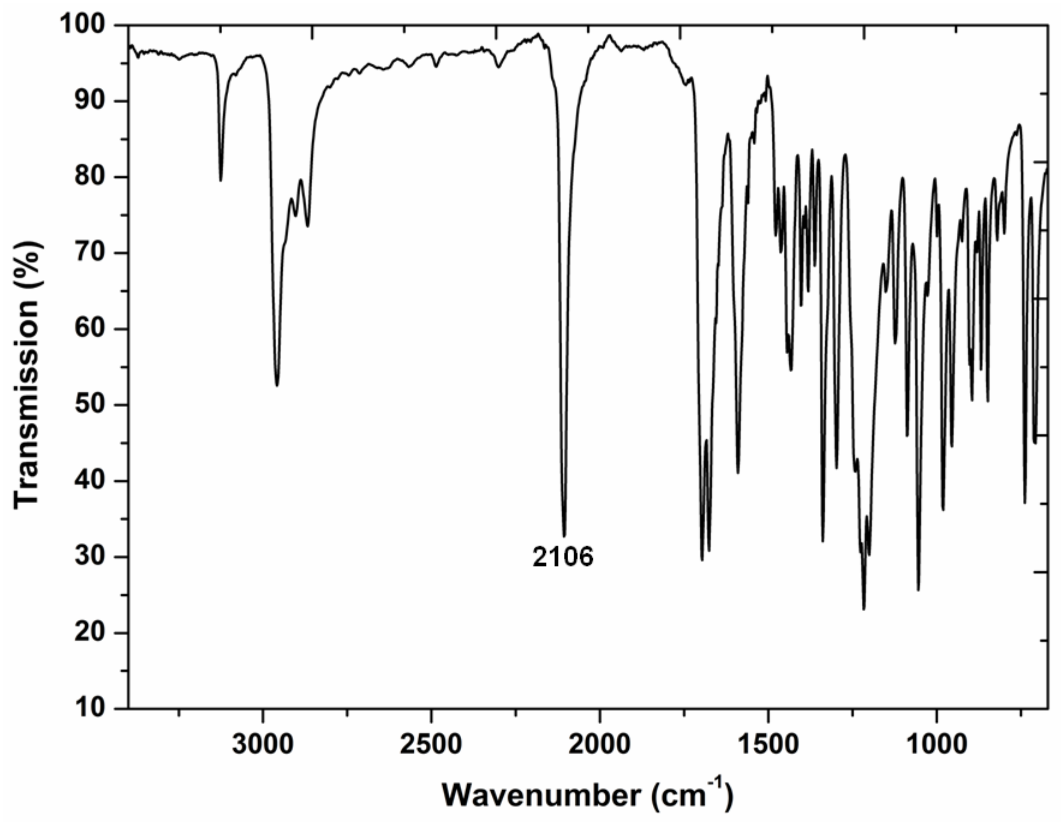

Figure S72 – FTIR spectrum of compound **14**.

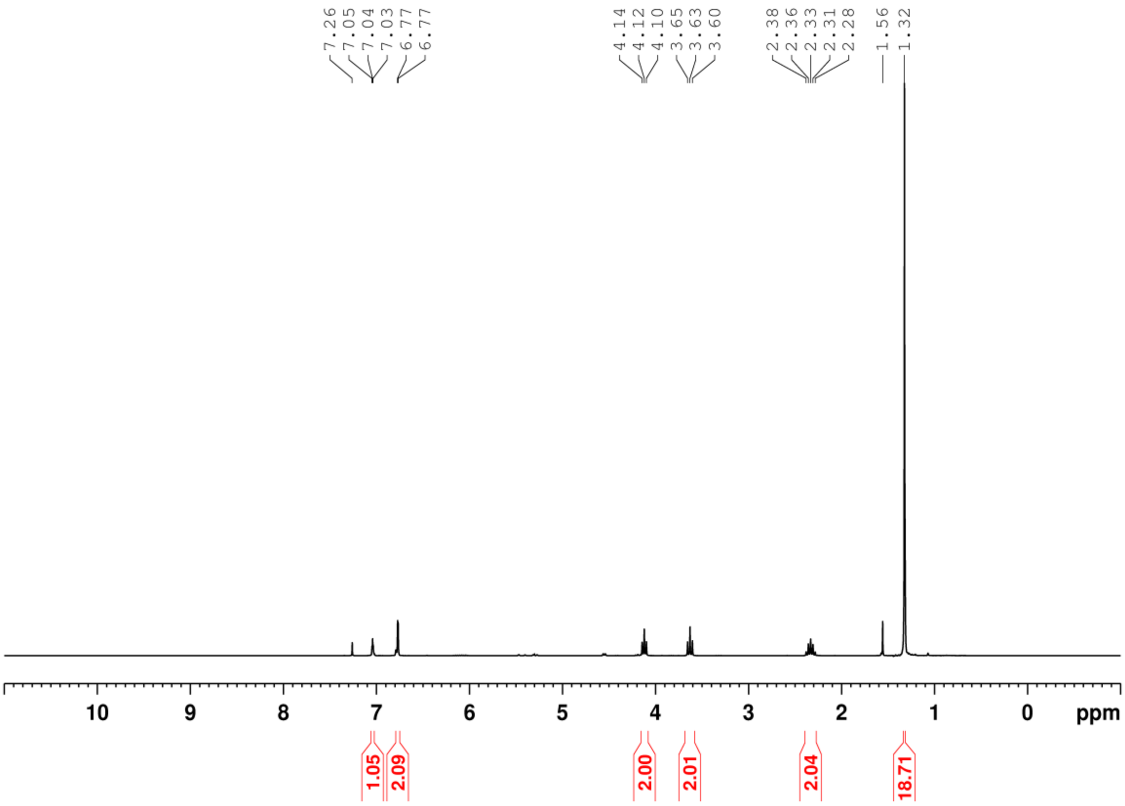

Figure S73 – ^{1}H NMR spectrum of compound **S5** (250 MHz, $CDCl_3$, 298 K).

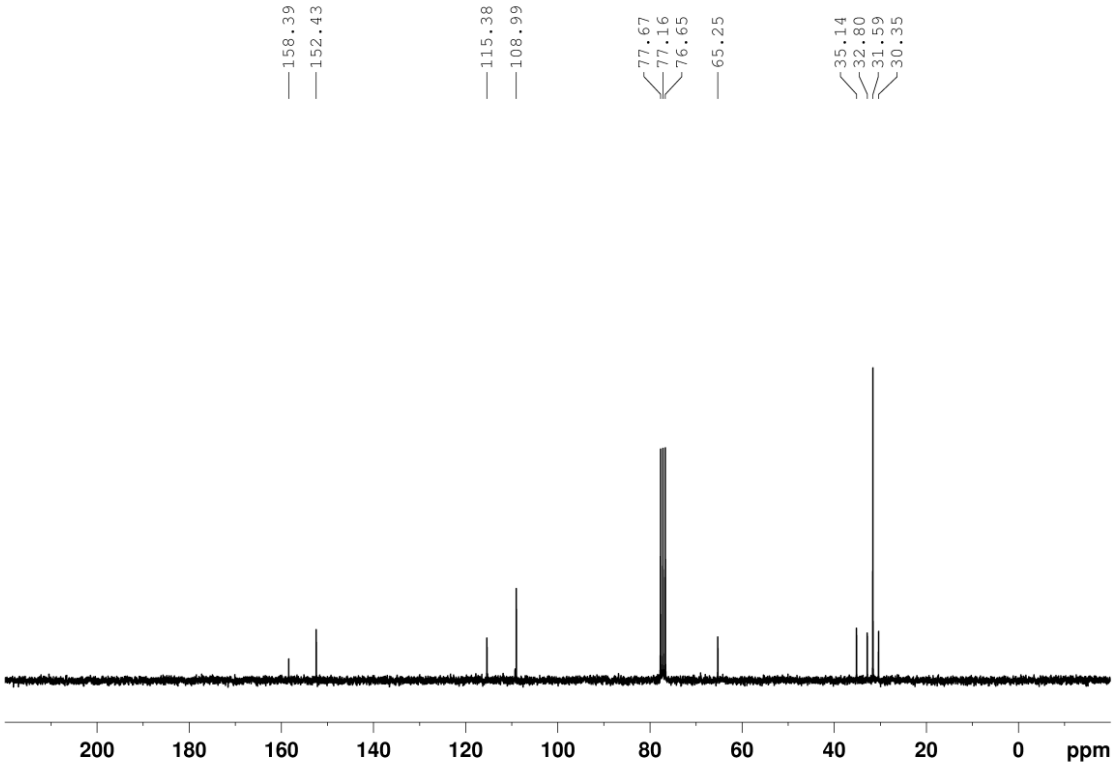

Figure S74 – ^{13}C NMR spectrum of compound **S5** (60 MHz, $CDCl_3$, 298 K).

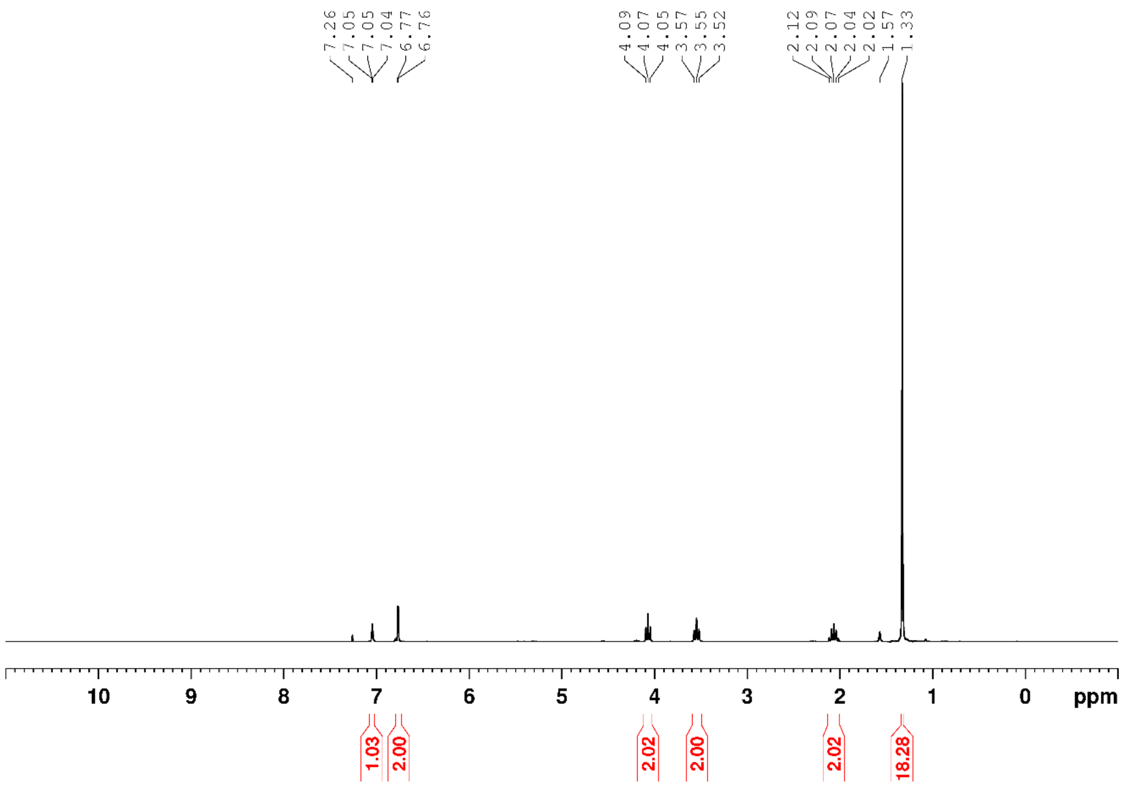

Figure S75 – ^{1}H NMR spectrum of compound **S6** (250 MHz, $CDCl_3$, 298 K).

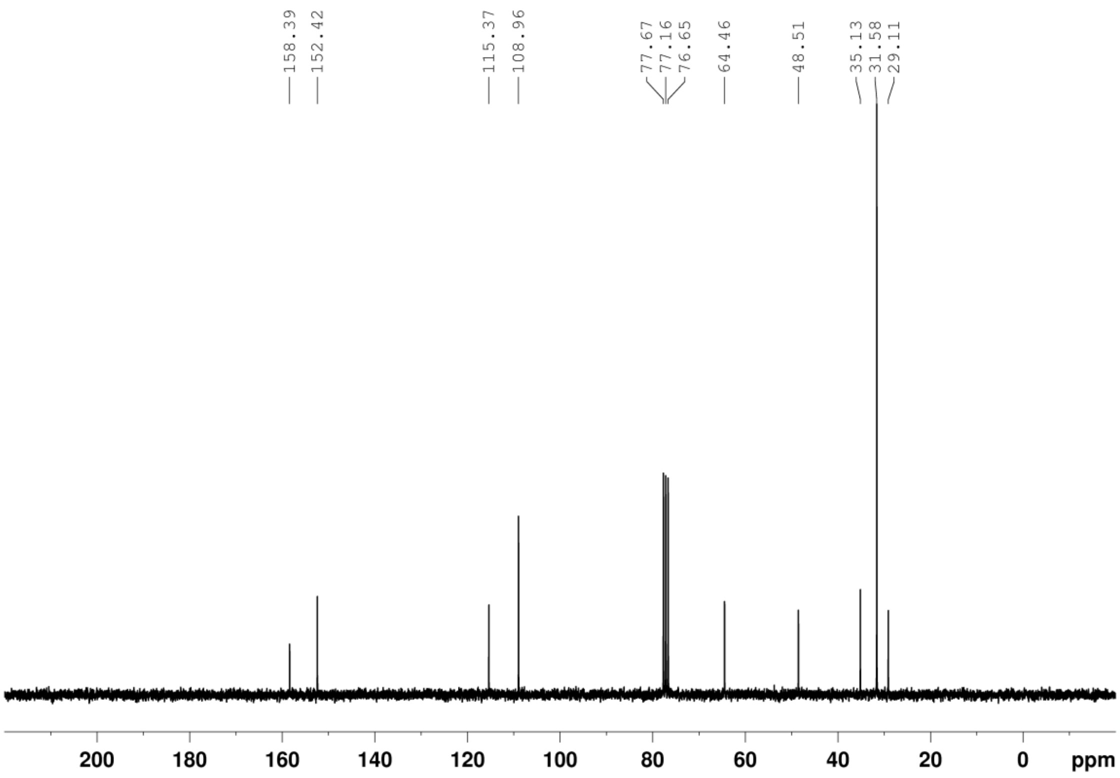

Figure S76 – ^{13}C NMR spectrum of compound **S6** (60 MHz, $CDCl_3$, 298 K).

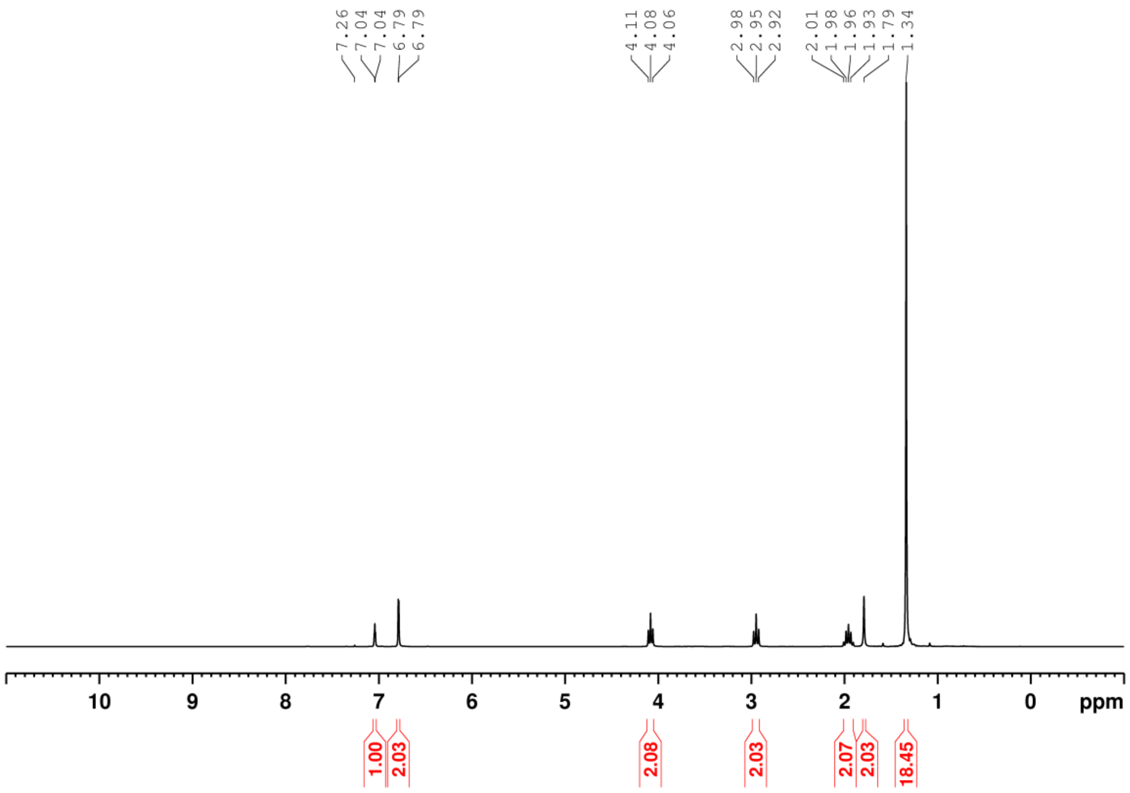

Figure S77 – ^{1}H NMR spectrum of compound **13** (250 MHz, $CDCl_3$, 298 K).

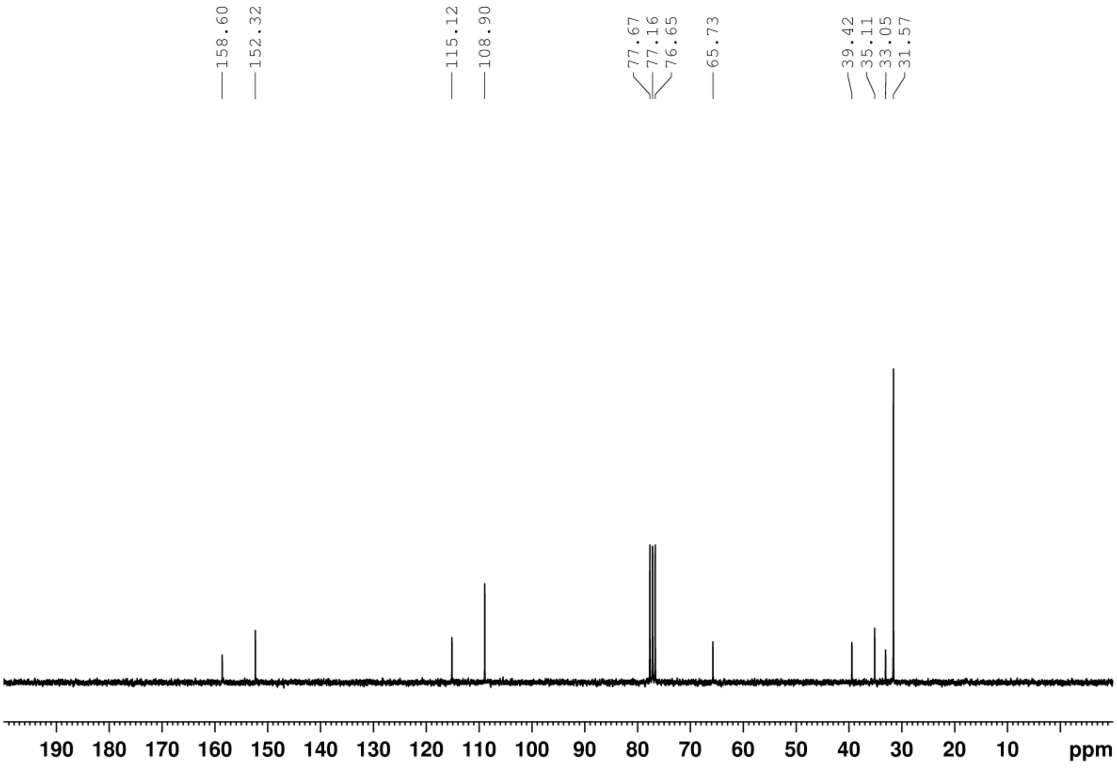

Figure S78 – ^{13}C NMR spectrum of compound **13** (60 MHz, $CDCl_3$, 298 K).

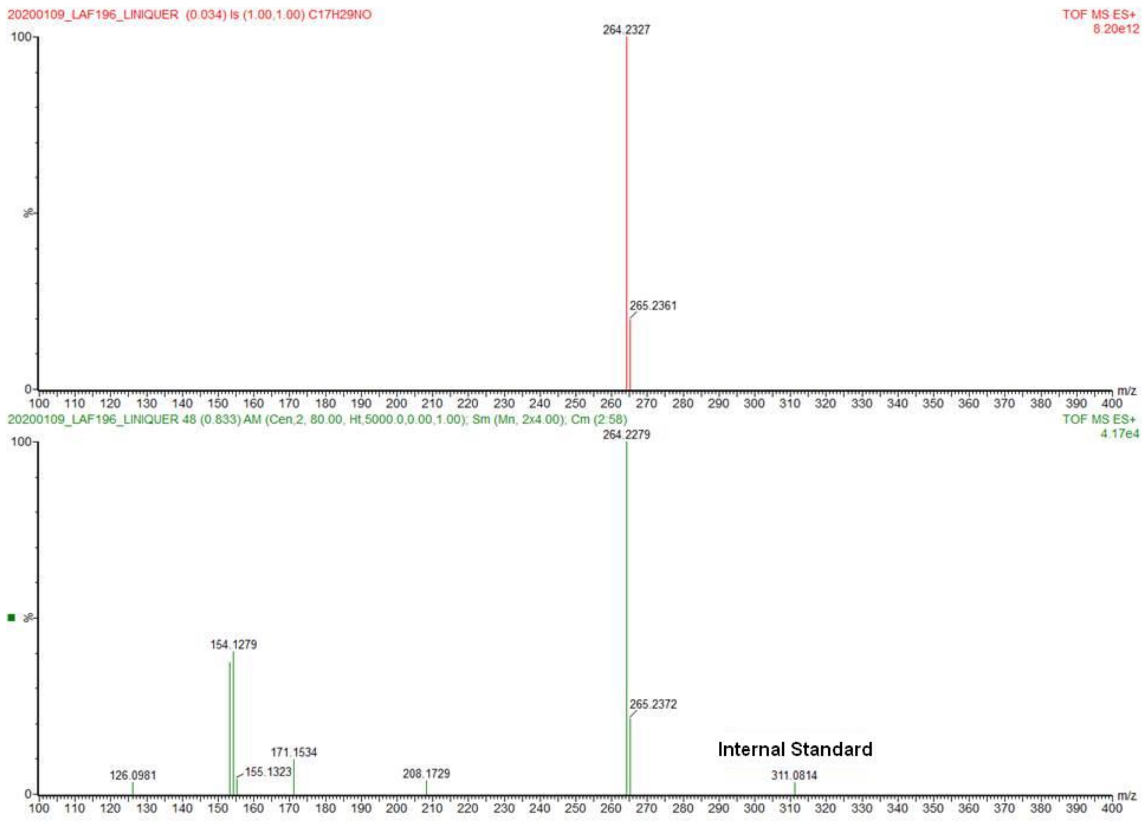

Figure S79 – High-resolution mass spectrum of compound **13**.

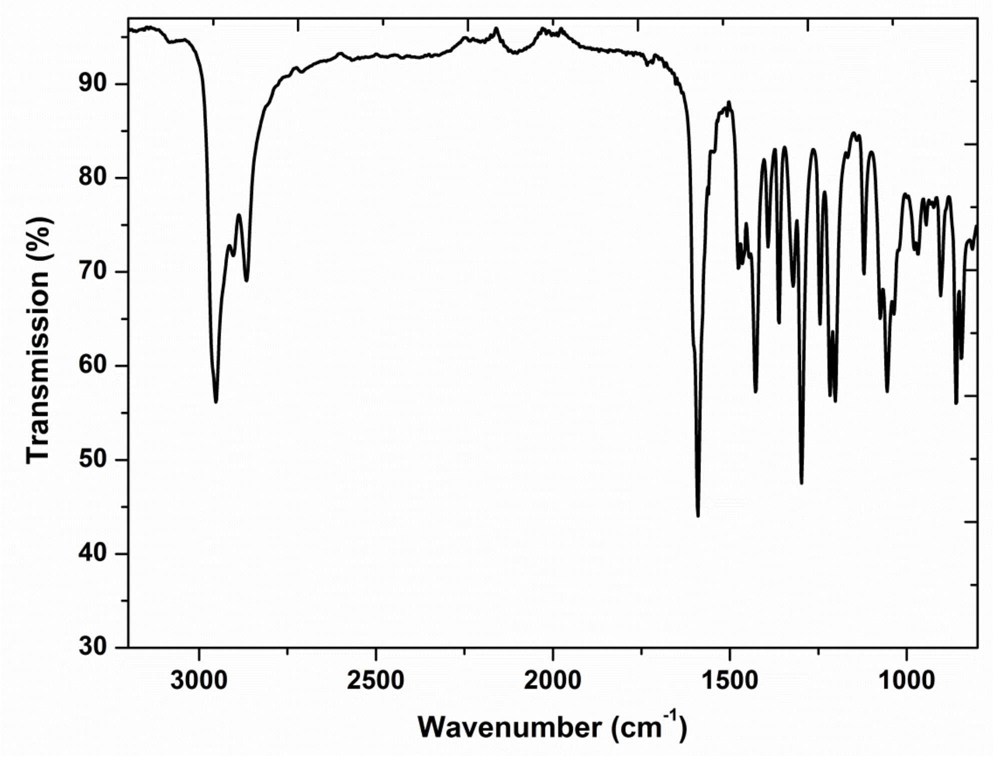

Figure S80 – FTIR spectrum of compound **13**.

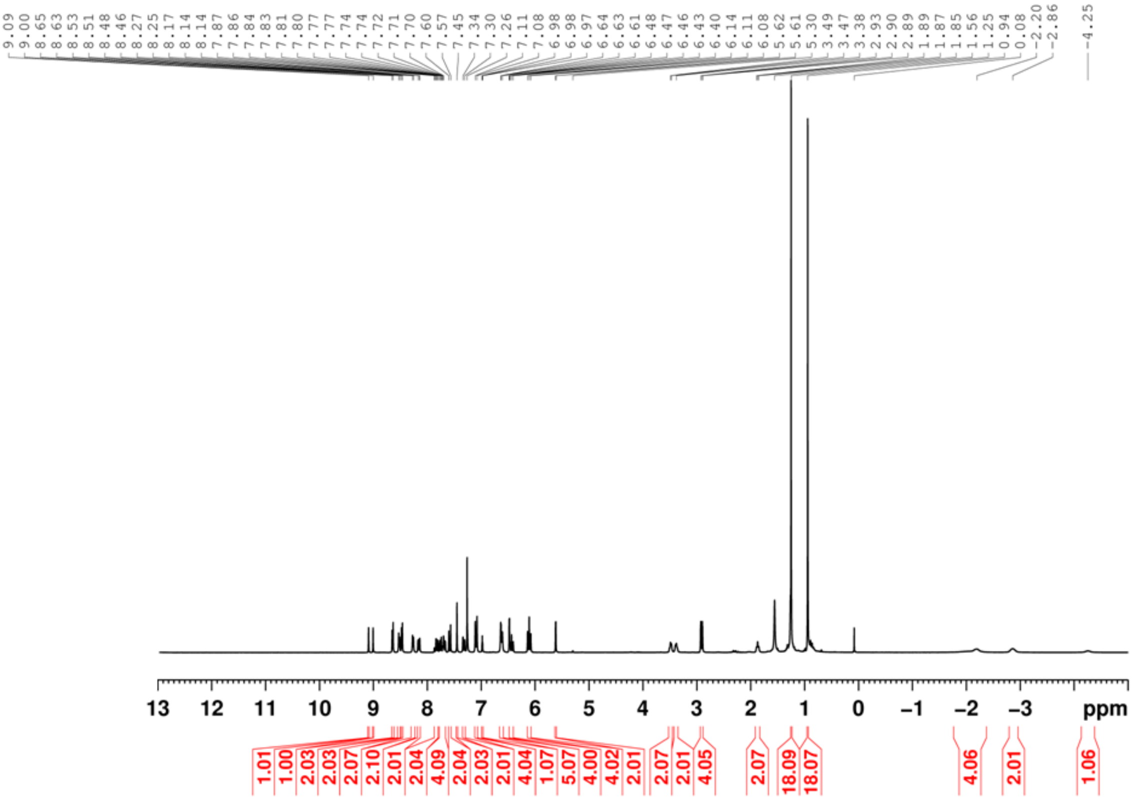

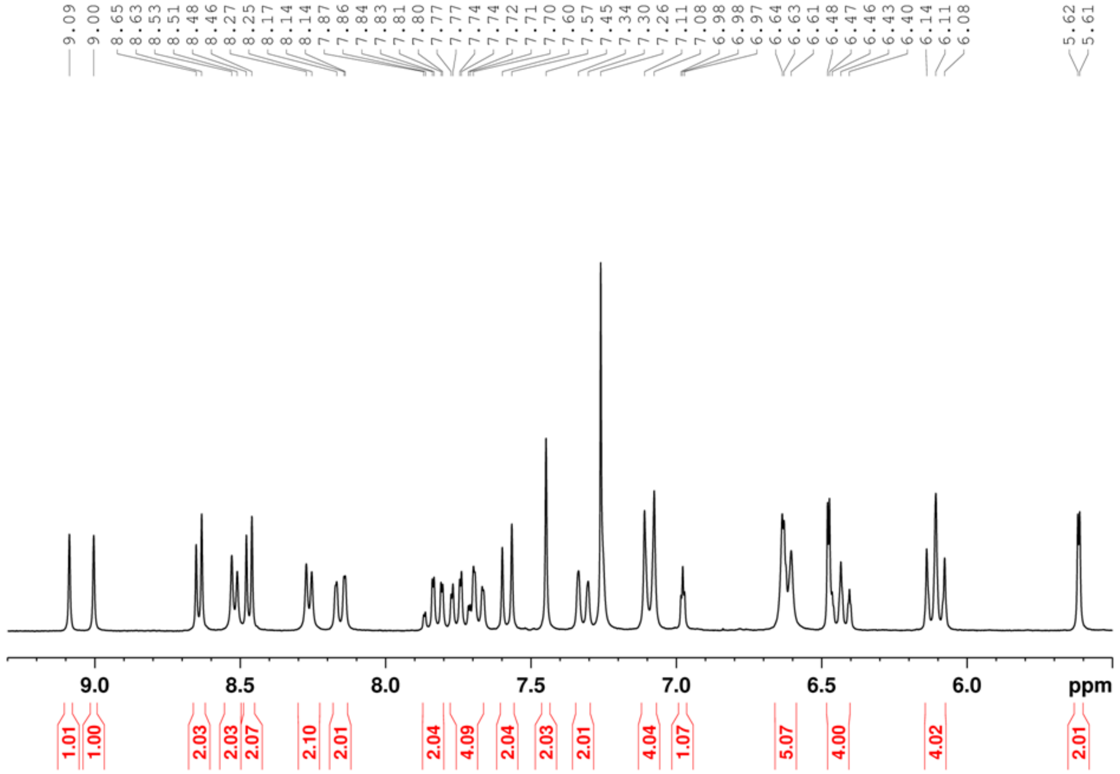

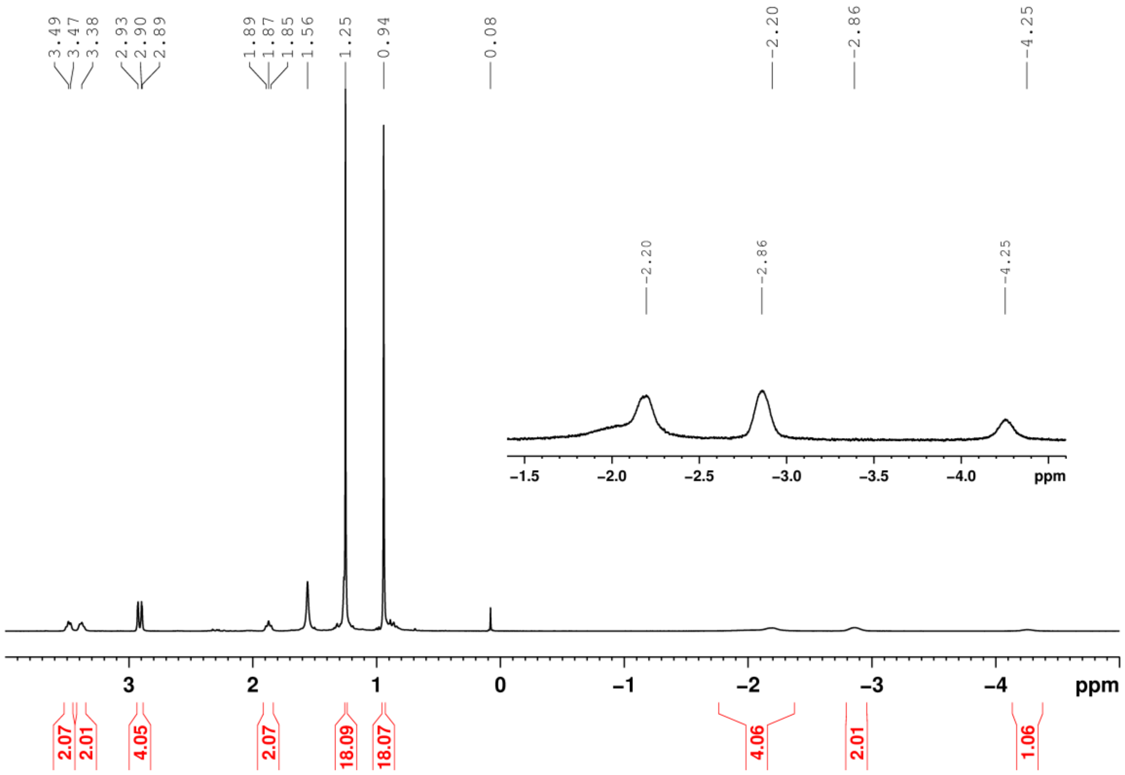

Figure S81 – ^{1}H NMR spectra of rotaxane **15** (250 MHz, $CDCl_3$, 298 K).

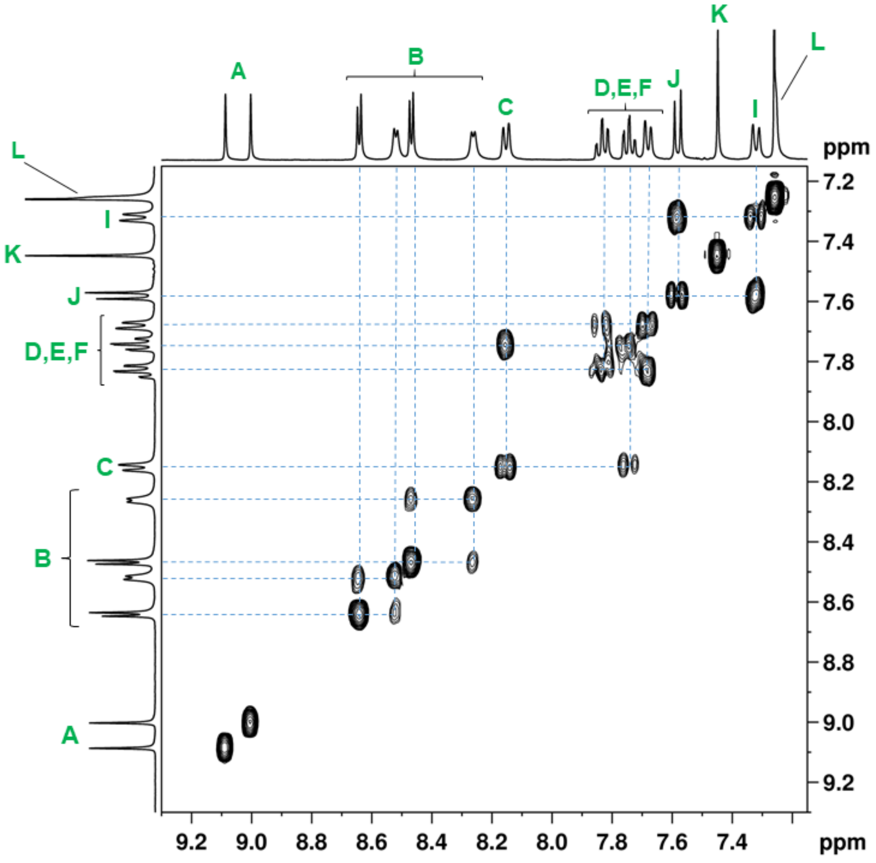

Figure S82 – Two-dimensional (^{1}H-^{1}H) COSY NMR spectrum of rotaxane **15** (400 MHz, $CDCl_3$, 298 K).

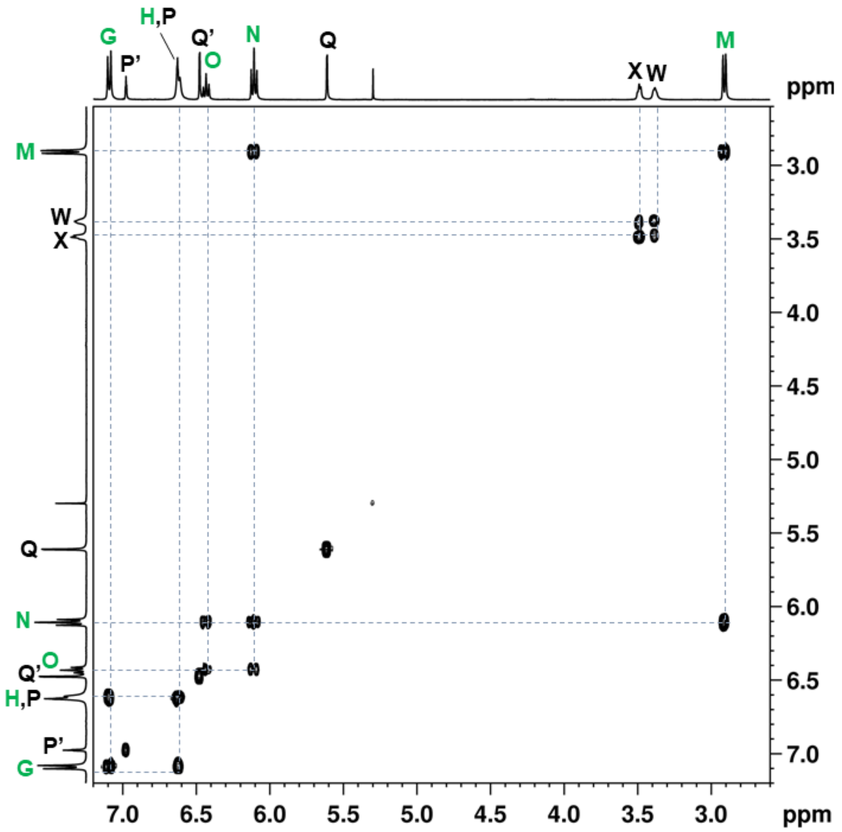

Figure S83 – Two-dimensional (^{1}H-^{1}H) COSY NMR spectrum of rotaxane **15** (400 MHz, $CDCl_3$, 298 K).

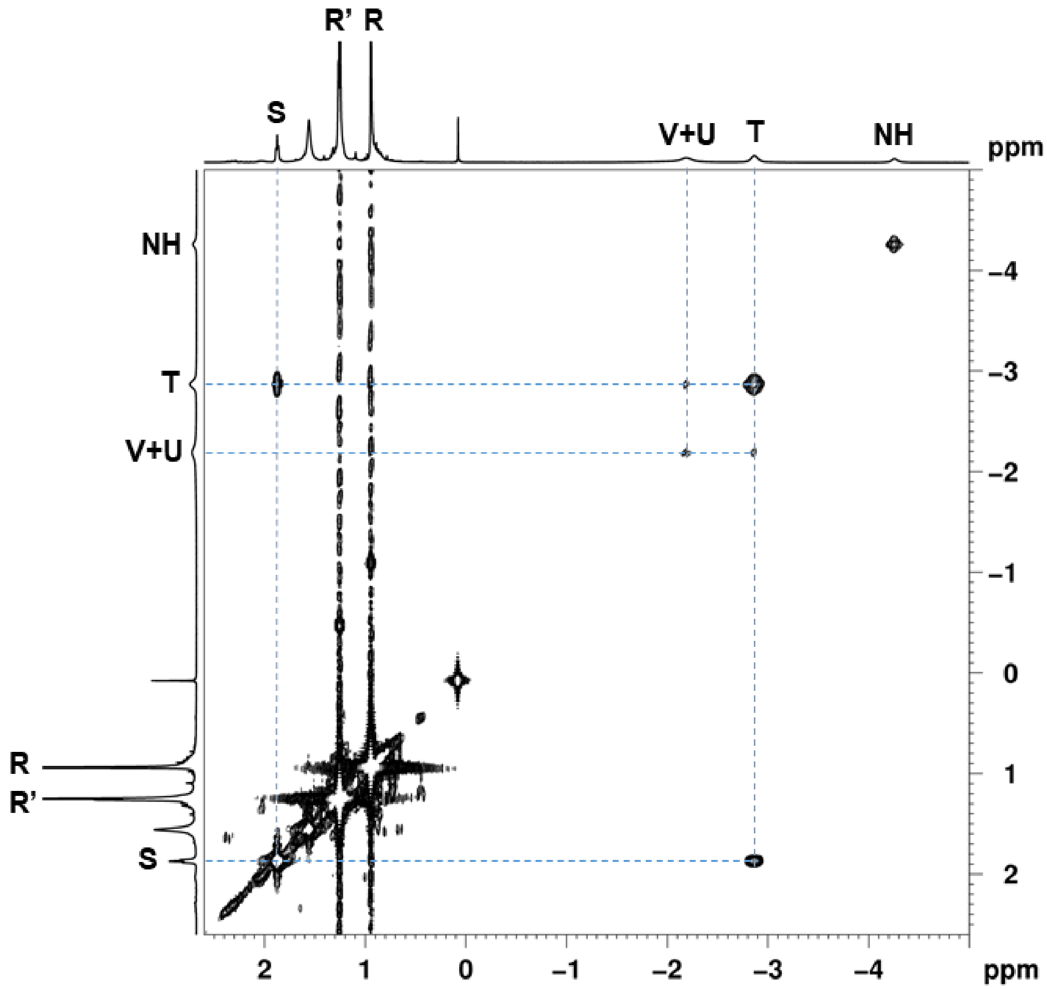

Figure S84 – Two-dimensional (^{1}H-^{1}H) COSY NMR spectrum of rotaxane **15** (400 MHz, $CDCl_3$, 298 K).

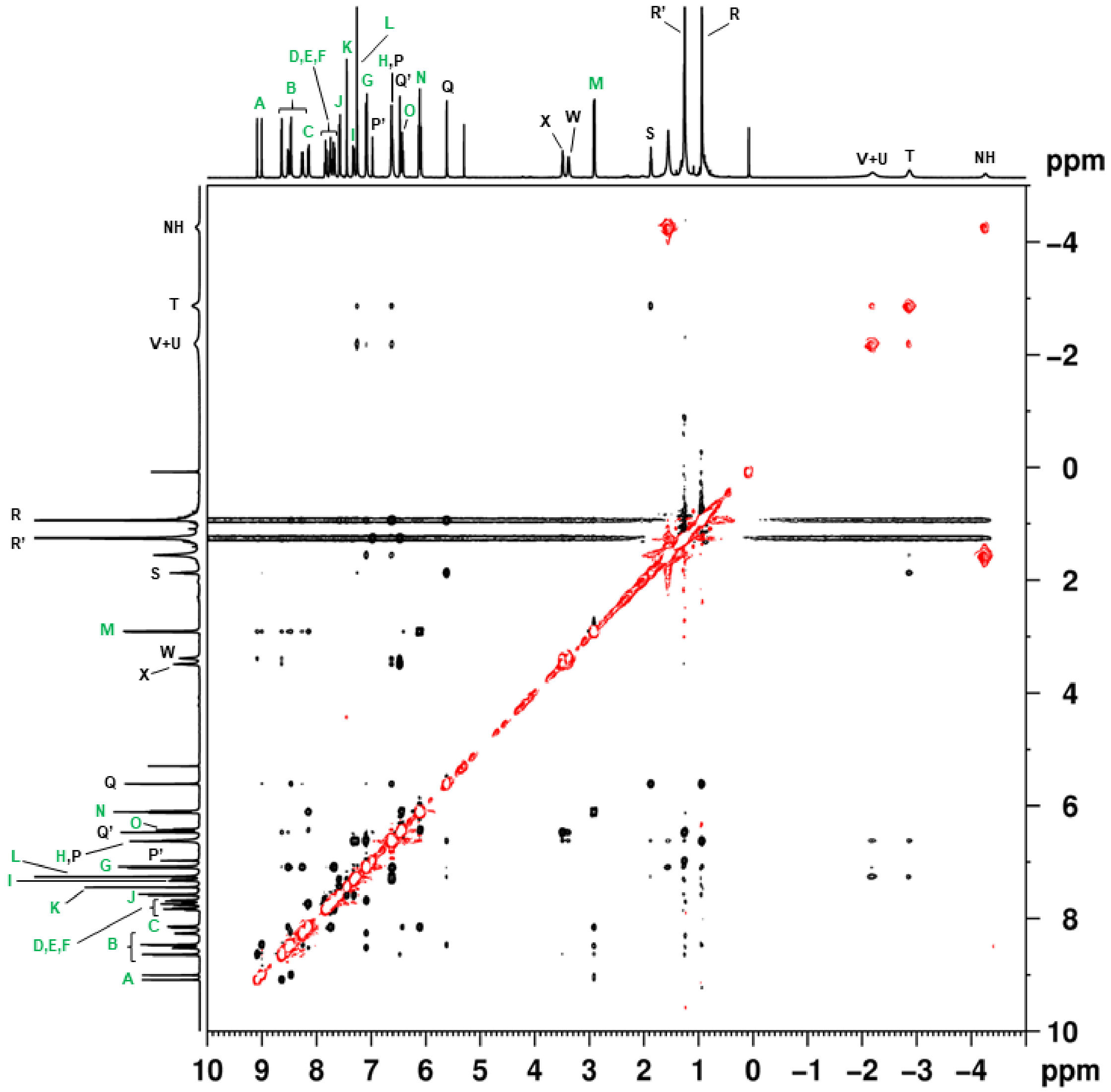

Figure S85 – Two-dimensional (^{1}H-^{1}H) NOESY NMR full spectrum of rotaxane **15** (400 MHz, $CDCl_3$, 298 K).

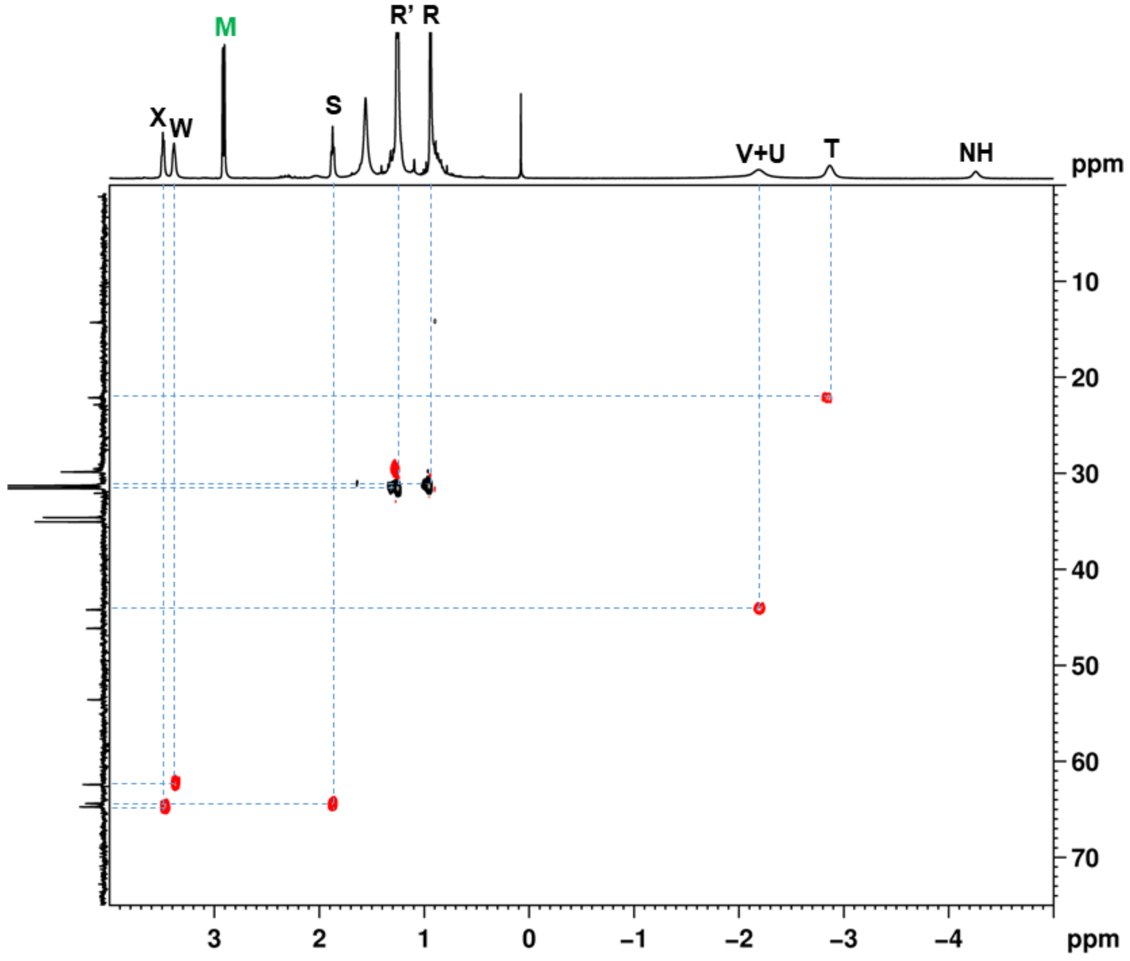

Figure S86 – Two-dimensional (^1H-^{13}C) HSQC NMR spectrum of rotaxane **15** (400 MHz, $CDCl_3$, 298 K). The CH_3 and CH groups are in black while CH_2 moieties are in red.

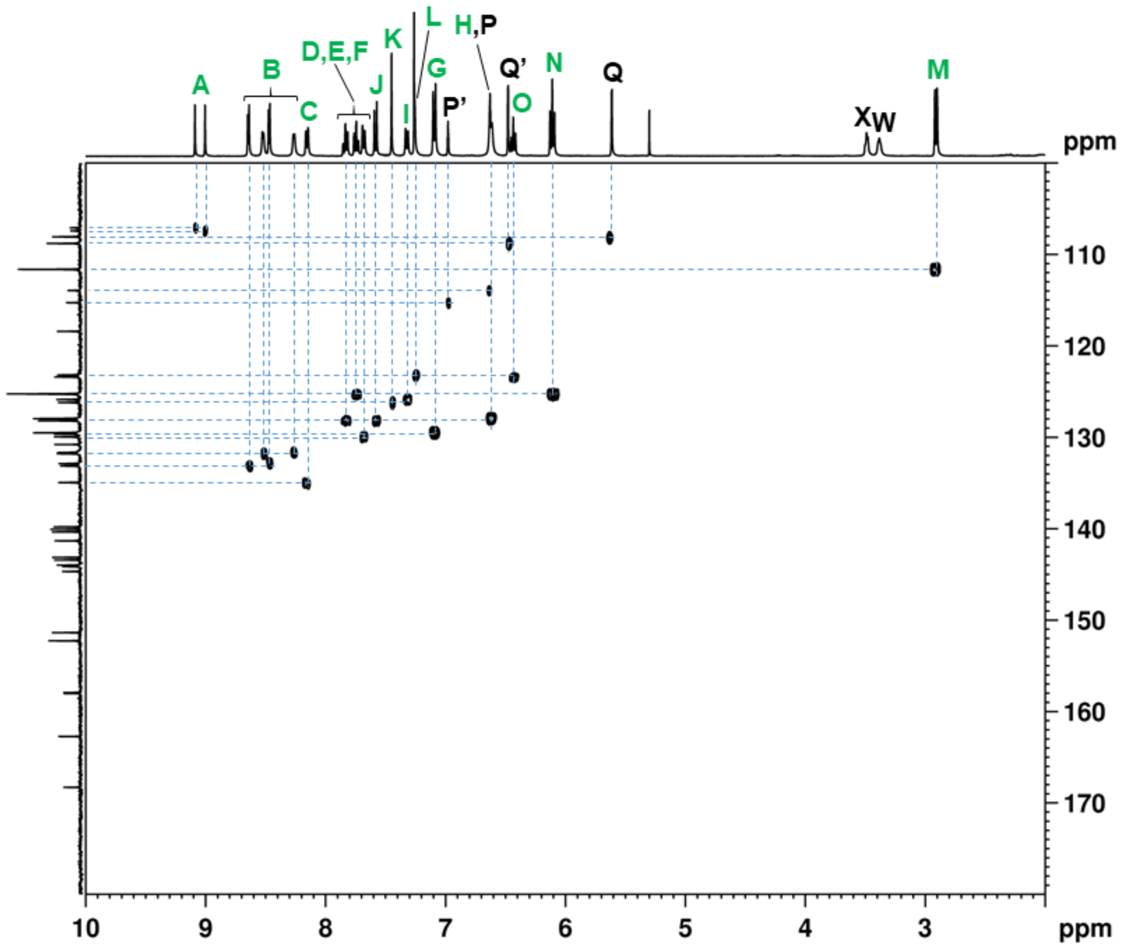

Figure S87 – Two-dimensional (^{1}H-^{13}C) HSQC NMR spectrum of rotaxane **15** (400 MHz, $CDCl_3$, 298 K). The CH_3 and CH groups are in black while the CH_2 moieties are in red.

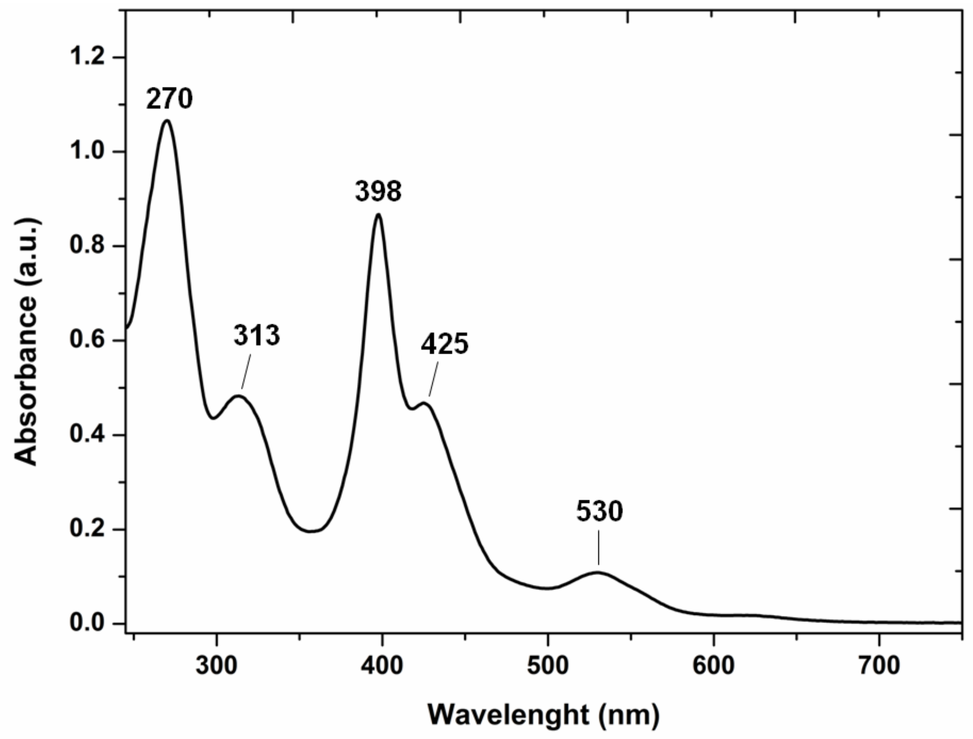

Figure S88 – Ground State UV-Vis absorption spectrum of rotaxane **15**. Conditions: DCM solutions at 10^{-5} M concentration, room temperature.

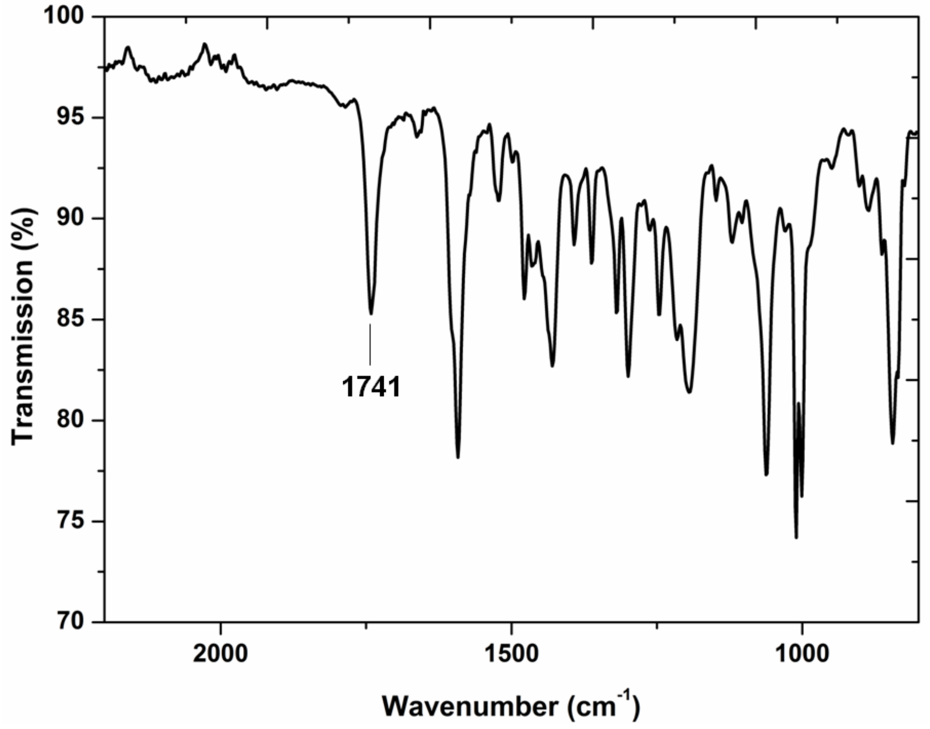

Figure S89 – FTIR spectrum of rotaxane **15.**

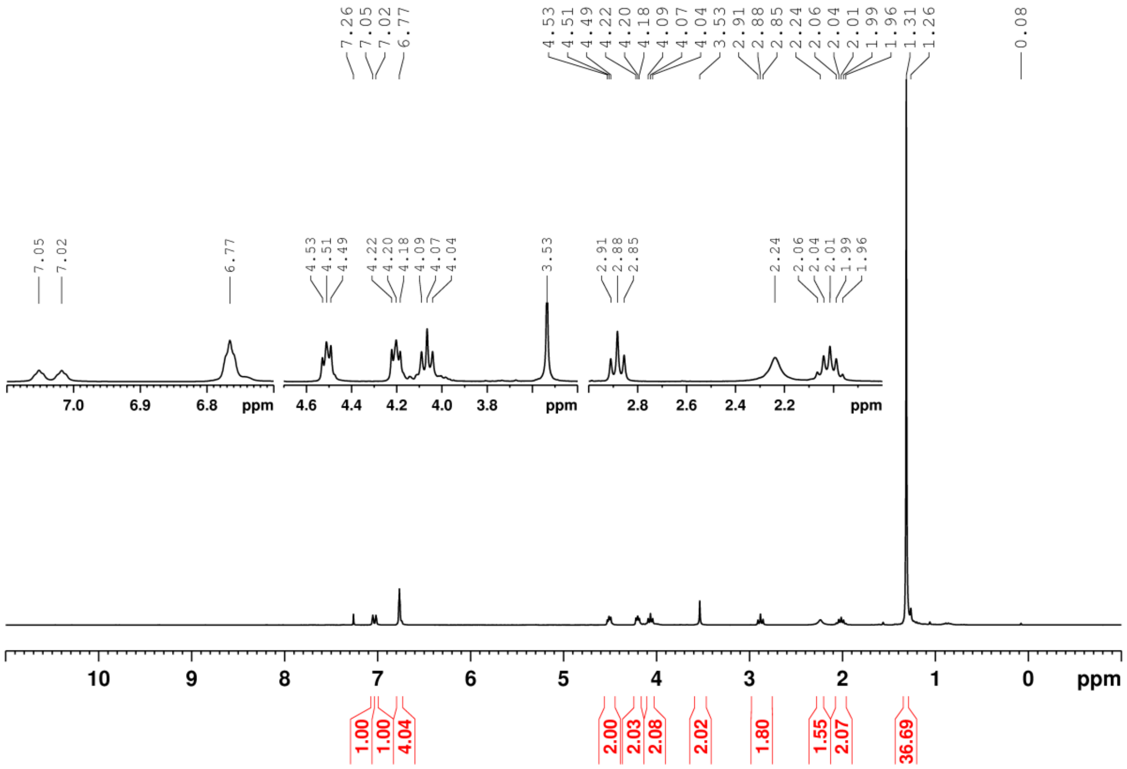

Figure S90 – ^{1}H NMR spectrum of thread **16** (250 MHz, $CDCl_3$, 298 K).

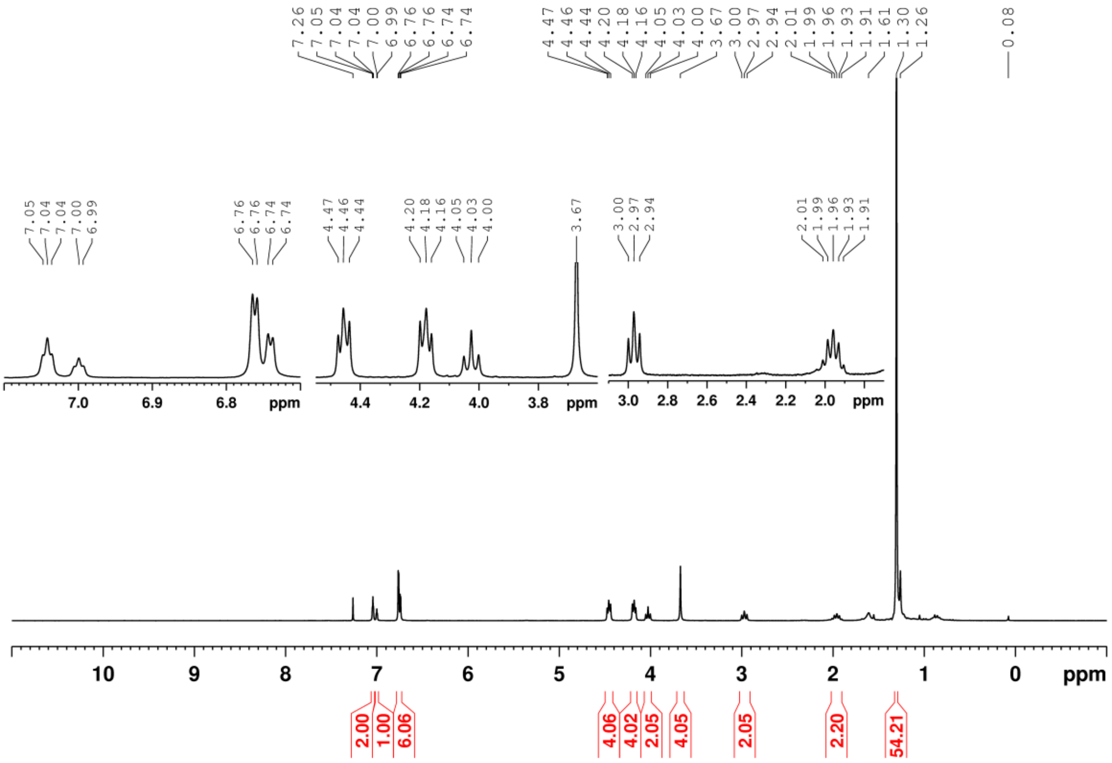

Figure S91 – ^{1}H NMR spectrum of the tertiary amine thread analogue afforded from the double carbene insertion reaction byproduct (250 MHz, $CDCl_3$, 298 K).

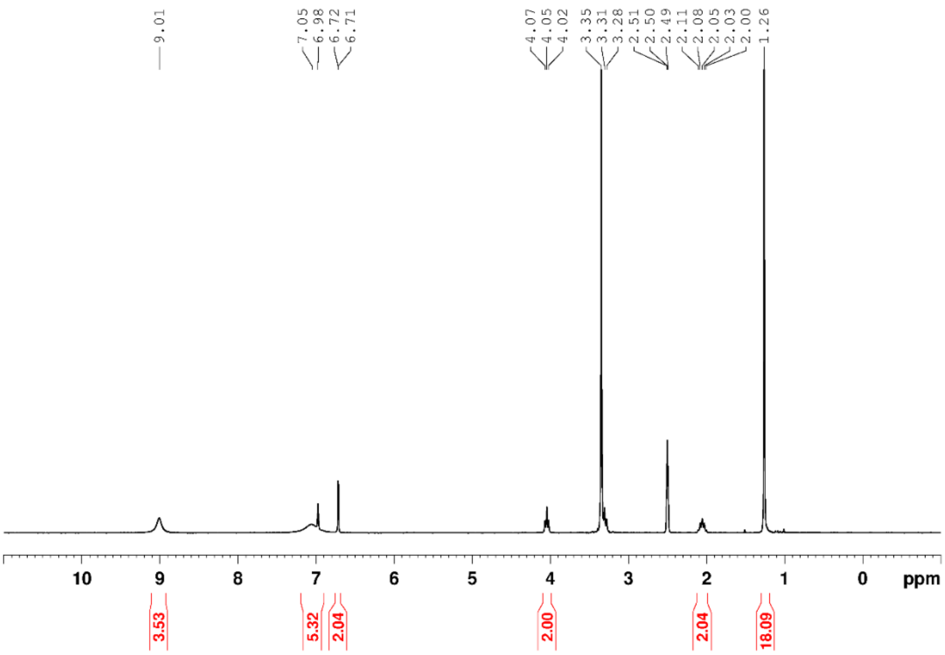

Figure S92 – ^{1}H NMR spectrum of compound **S7** (250 MHz, DMSO-d_6, 298 K).

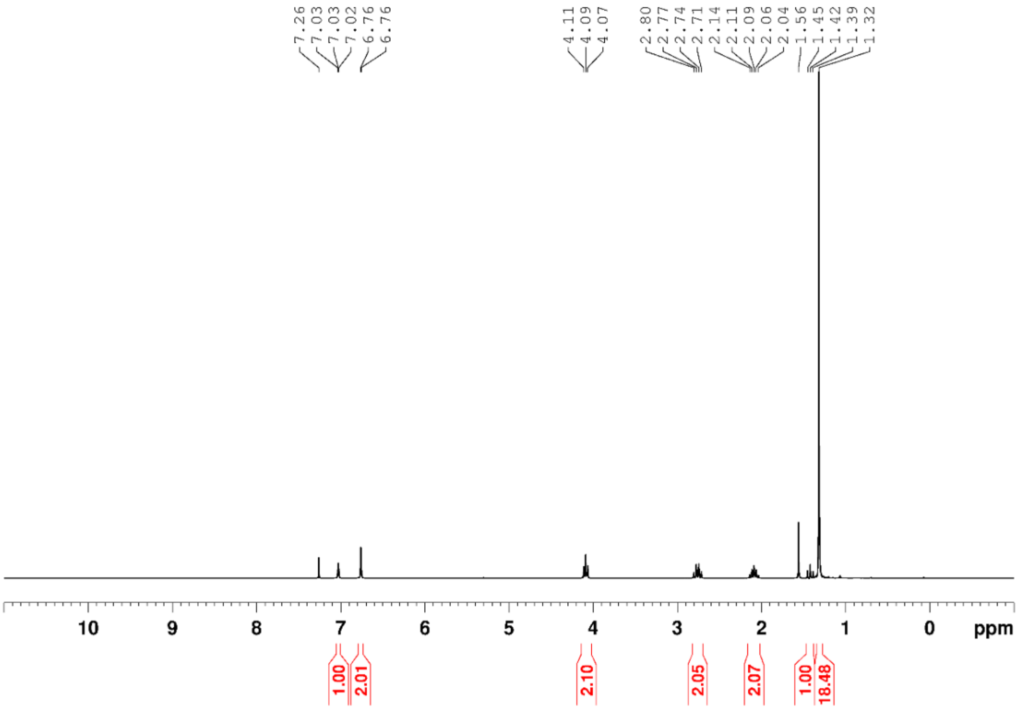

Figure S93 – ^{1}H NMR spectrum of compound **17** (250 MHz, $CDCl_3$, 298 K).

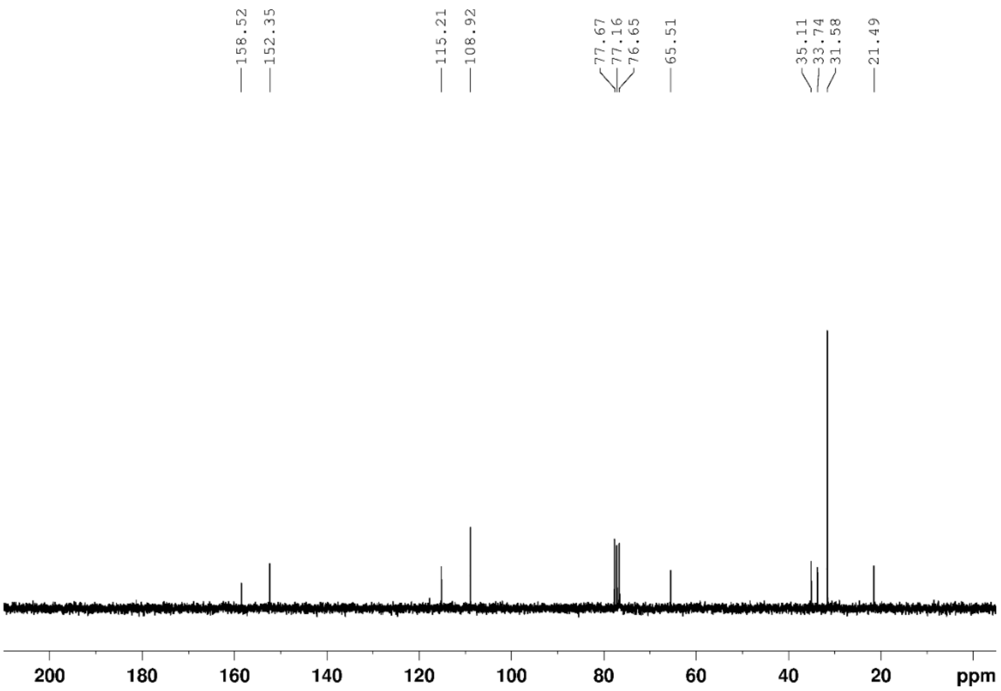

Figure S94 – ^{13}C NMR spectrum of compound **17** (250 MHz, $CDCl_3$, 298 K).

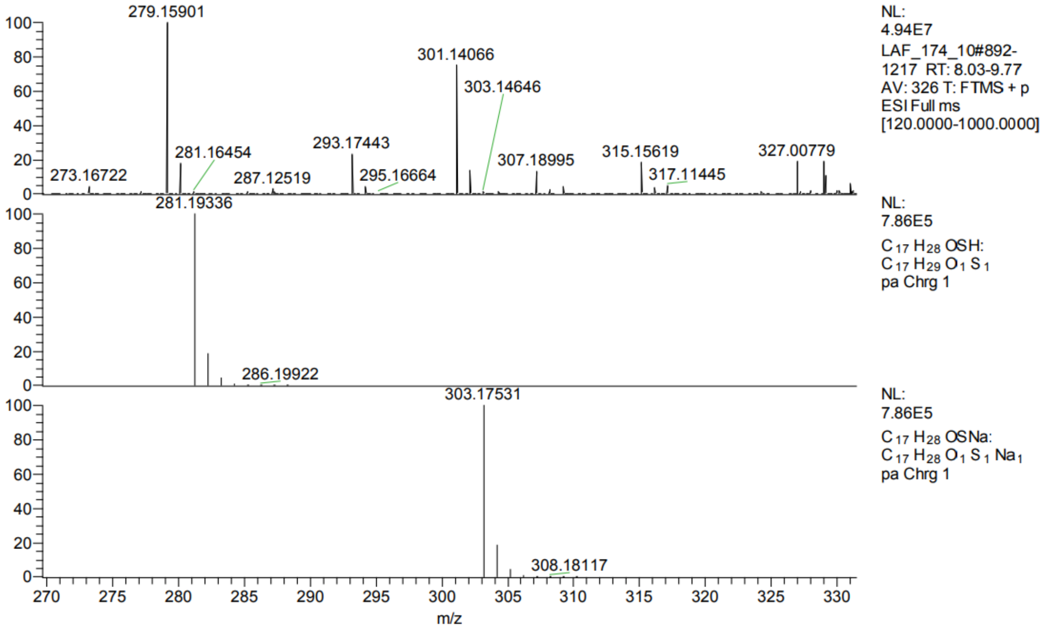

Figure S95 – High-resolution mass spectrum of compound **17**.

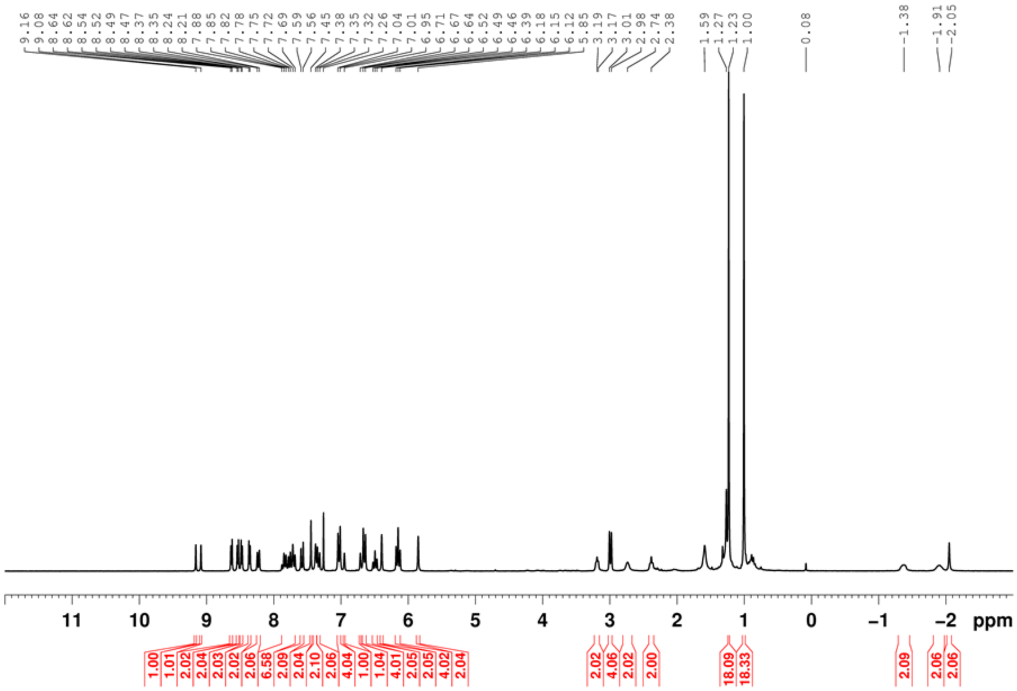

Figure S96 – ^{1}H NMR spectrum of rotaxane **19** (250 MHz, $CDCl_3$, 298 K).

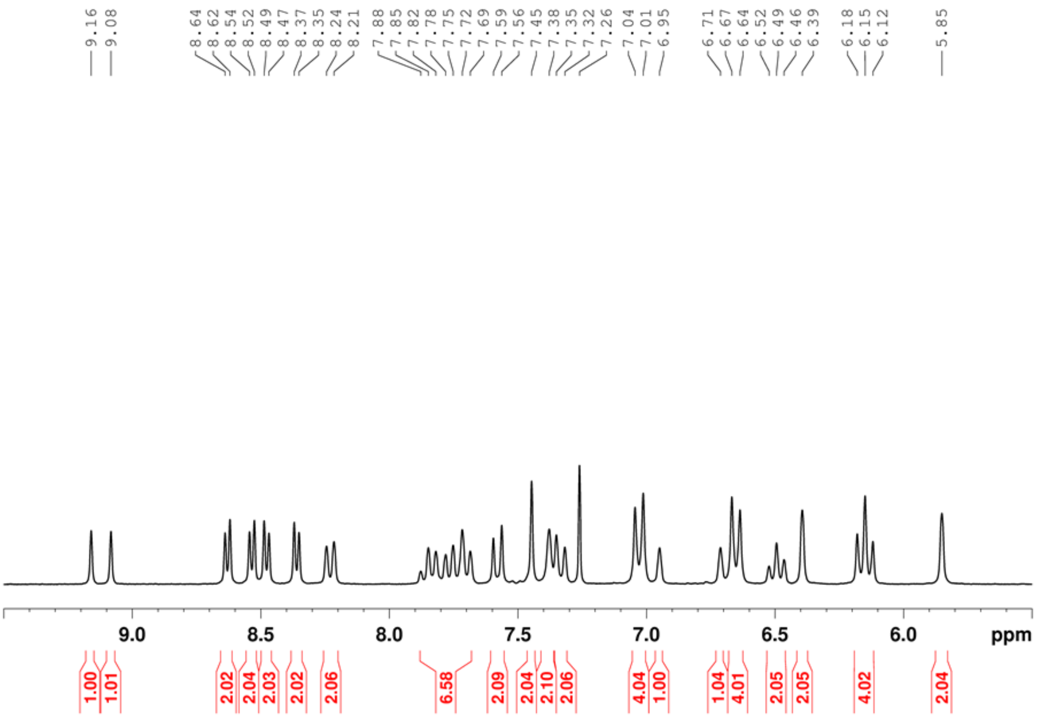

Figure S97 – Aromatic region of the ^{1}H NMR spectrum of rotaxane **19** (250 MHz, $CDCl_3$, 298 K).

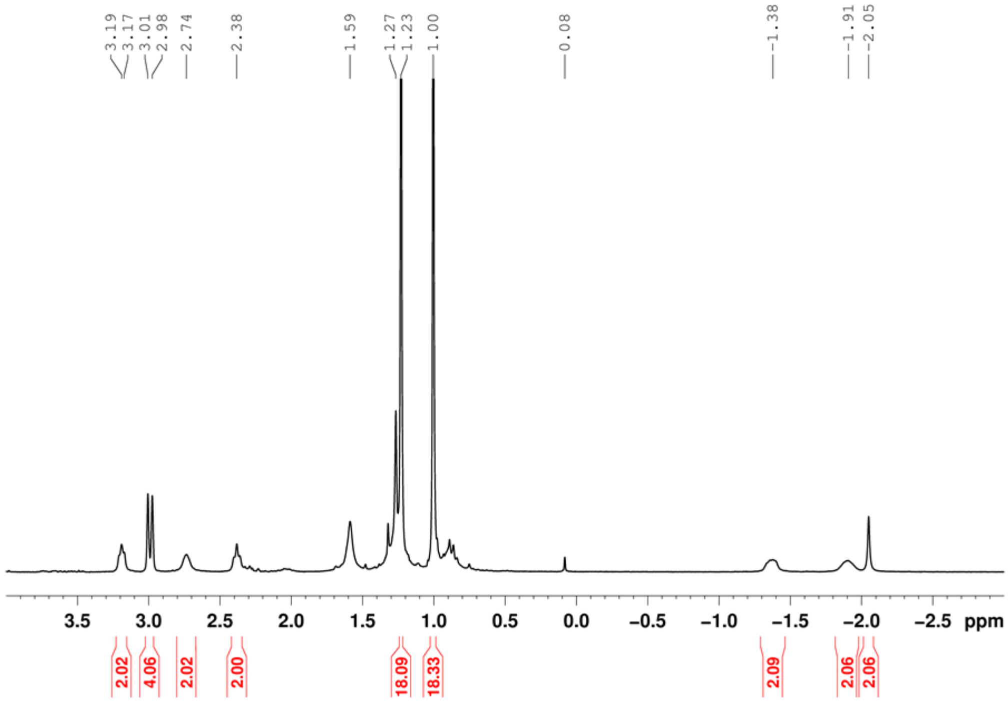

Figure S98 – Aliphatic region of the ^{1}H NMR spectrum of rotaxane **19** (250 MHz, $CDCl_3$, 298 K).

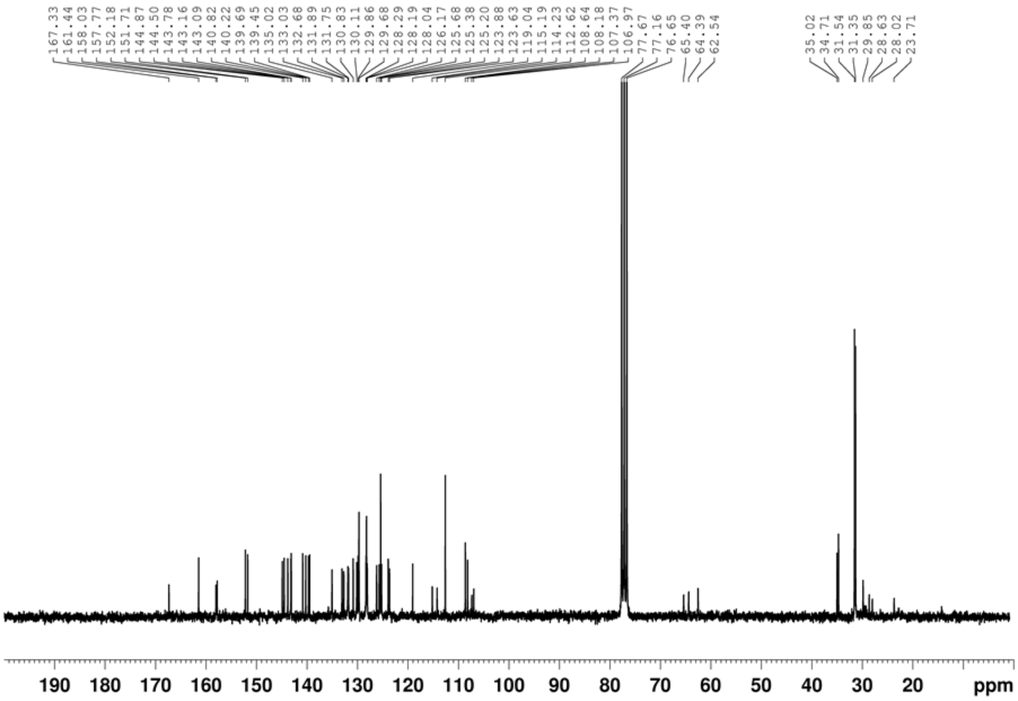

Figure S99 – ^{13}C NMR spectrum of rotaxane **19** (60 MHz, $CDCl_3$, 298 K).

5. Crystal Data

The X-ray diffraction experiments were performed at MX2 beamline.[S9] MX2 is a wiggler beamline dedicated to Macromolecular Crystallography at the UVX synchrotron source at the Brazilian Synchrotron Light Source. It operates on a 2.0 T hybrid 30-pole wiggler and its optical layout includes collimating mirror, Si(111) double-crystal monochromator and toroidal bendable mirror. The MX2 beamline provides wide tunability between 5 and 15 keV with maximum flux at 8.5 keV. The beamline is equipped with PILATUS2M detector from Dectris and a mini-Kappa goniometer from Arinax. For the data collection of the crystal of macrocycle **7**, 360 frames were collected using phi-scans (1°/frame, 30s of exposition for scan, kappa 0°) and additional 180 frames were collected using phi-scans with kappa axis at kappa 45°. The wavelength used for this sample was 0.82665 Å. For the data collection of the crystal of macrocycle **11**, 360 frames were collected using phi-scans with kappa at 0° and 360 additional frames with kappa at 45°. Each frame was acquired within 5s of x-ray exposition with 1°/frame. Both scans were performed with a wavelength of 0.82602 Å. Both crystals were kept at 100 ± 2 K during the x-ray diffraction experiments.

For macrocycle **7**, red needle-like single crystals (growth by slow evaporation of a methanol/CH_2Cl_2/THF solution) of $C_{58}H_{36}N_4$•H_3COH (MM= 820.95 g/mol) with approximate dimensions of 0.025 x 0.01 x 0.05 mm were placed in a Micromount supported with mineral oil. Crystal reflections indexing, unit-cell parameters refinement, integration and corrections were performed by CCP4,[S10] XIA2 0.5.653-g9f819c0c-dials-1.11,[S11] DIALS 1.11.2-g01fb9e997-release.[S12] Data merging and scale were performed using Aimless[S13] and Pointless.[S14] The number of reflections measured for the crystal of macrocycle **7** was 31887 (3.13° ≤ 2Θ ≤ 56.26°), 3996 unique (Rint =0.1775, Rsigma = 0.1186) which were used in all calculations.

For complex **11**, red needle-like single crystals (grown from slow evaporation of a dichloromethane/acetonitrile saturated solution) with approximate dimensions of 0.27 × 0.08 × 0.08 mm were placed in a Micromount supported with mineral oil. The number of reflections measured for the crystal of macrocycle **11** was 56903 (4.14° ≤ 2Θ ≤ 59.68°), 8927 unique (Rint =0.0591, Rsigma = 0.0356) which were used in the calculations. Using Olex2,[S15] the structures were solved with the ShelXT[S16] structure solution program using Intrinsic Phasing and refined with the XL[S17] refinement package using Least Squares minimization. The position of all non-hydrogen atoms was refined anisotropically. The

hydrogen atoms in the compounds were added to the structures in idealized positions and further refined according to the riding model. Uiso(H) = 1.2Ueq(C) for aromatic and N groups. Uiso(H) = 1.5Ueq(C) for methyl groups and O groups. Tables S1, S2 and S3 present crystal data, data collection and refinement data for macrocycle **7**, respectively. For macrocycle **11**, tables S4, S5 and S6 gather the crystal data, data collection and refinement data, respectively.

Table S1 – Sample and crystal data for macrocycle **7**.

$C_{58}H_{36}N_4\,0.5(C_2H_8O_2)\cdot$	D_x = 1.222 Mg m^{-3}
M_r = 820.95	Synchrotron radiation, λ = 0.82665 Å
Orthorhombic, *Ima*2	Cell parameters from 4238 reflections
a = 29.4081 (10) Å	θ = 1.6–28.1°
b = 12.2692 (3) Å	μ = 0.10 mm^{-1}
c = 12.3661 (4) Å	*T* = 100 K
V = 4461.9 (2) Å^3	Needle, red
Z = 4	0.05 × 0.025 × 0.01 mm
F(000) = 1720	CCDC number: 1883084

Table S2 - Data collection for macrocycle **7**.

MX2/LNLS diffractometer	R_{int} = 0.178
Absorption correction: empirical (using intensity measurements) *CCP4* 7.0.066: AIMLESS, version 0.7.3: 15/08/18 Scaling & analysis of unmerged intensities, absorption correction using spherical harmonics	θ_{max} = 28.1°, θ_{min} = 1.6°
T_{min} = 0.986, T_{max} = 1.0	*h* = -33→11
31887 measured reflections	*k* = -15→15
3996 independent reflections	*l* = -15→12
2532 reflections with $I > 2\sigma(I)$	

Table S3 – Refinement of the data of macrocycle **7**.

Refinement on F^2	Hydrogen site location: inferred from neighbouring sites
Least-squares matrix: full	H-atom parameters constrained
$R[F^2 > 2\sigma(F^2)]$ = 0.106	$w = 1/[\sigma^2(F_o^2) + (0.2P)^2]$ where $P = (F_o^2 + 2F_c^2)/3$
$wR(F^2)$ = 0.298	$(\Delta/\sigma)_{max}$ < 0.001
S = 1.05	$\Delta\rangle_{max}$ = 1.17 e Å^{-3}

3996 reflections	$\Delta\rangle_{min}$ = -0.47 e Å^{-3}
296 parameters	Absolute structure: Flack x determined using 847 quotients [(I+)-(I-)]/[(I+)+(I-)] (Parsons and Flack (2004), Acta Cryst. A60, s61).
1 restraint	Absolute structure parameter: -0.8 (10)
Primary atom site location: dual	

Table S4 – Sample and crystal data for macrocycle **11**.

$C_{71}H_{46}N_4ORu\cdot C_2H_3N\cdot CH_2Cl_2$	Z = 2
M_r = 1198.16	F(000) = 1232
Triclinic, $P\overline{1}$	D_x = 1.444 Mg m^{-3}
a = 11.939 (4) Å	Synchrotron radiation, λ = 0.82602 Å
b = 15.318 (3) Å	Cell parameters from 20761 reflections
c = 16.282 (4) Å	θ = 1.5–29.6°
α = 104.491 (12)°	μ = 0.65 mm^{-1}
β = 97.892 (13)°	T = 100 K
γ = 102.523 (12)°	Needle, red
V = 2755.9 (13) Å^3	0.27 × 0.08 × 0.08 mm

Table S5 - Data collection for macrocycle **11**.

MX2 LNLS<br>diffractometer	R_{int} = 0.059
Absorption correction: empirical (using intensity measurements)<br>XDS (Kabsch, 2010)	θ_{max} = 31.4°, θ_{min} = 2.1°
T_{min} = 0.71, T_{max} = 1	h = -12→12
56903 measured reflections	k = -19→19
9235 independent reflections	l = -18→18
7911 reflections with $I > 2\sigma(I)$	

Table S6 – Refinement of the data of macrocycle **11**.

Refinement on F^2	Primary atom site location: dual
Least-squares matrix: full	Hydrogen site location: mixed
$R[F^2 > 2\sigma(F^2)]$ = 0.057	H-atom parameters constrained
$wR(F^2)$ = 0.164	$w = 1/[\sigma^2(F_o^2) + (0.1013P)^2 + 4.1805P]$<br>where $P = (F_o^2 + 2F_c^2)/3$
S = 1.04	$(\Delta/\sigma)_{max}$ < 0.001
9235 reflections	$\Delta\rangle_{max}$ = 1.50 e Å^{-3}
750 parameters	$\Delta\rangle_{min}$ = -1.19 e Å^{-3}
0 restraints	

Table S7 – Main bond lengths (Å) and angles (°) for macrocycle **11**.

Ru1—N1	2.040(4)	C59—Ru1—O1	176.3(2)
Ru1—N2	2.048(3)	N1—Ru1—N2	89.0(1)
Ru1—N3	2.036(4)	N2—Ru1—N3	90.5(1)
Ru1—N4	2.044(3)	N3—Ru1—N4	89.1(1)
Ru1—C59	1.852(4)	N4—Ru1—N1	90.5(1)
Ru1—O1	2.371(3)	C59—Ru1—N1	93.4(2)
		C59—Ru1—N2	91.2(2)
		C59—Ru1—N3	92.3(2)
		C59—Ru1—N4	98.5(2)

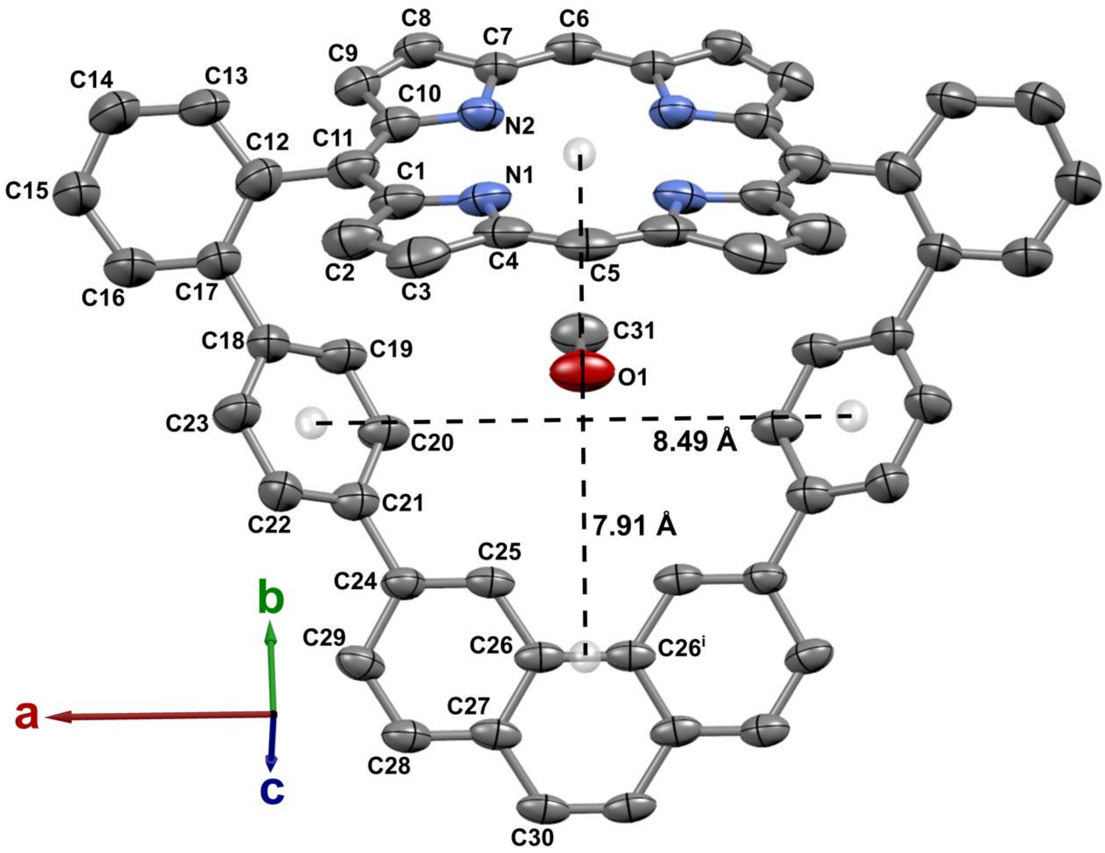

Figure S100 – Estimated cavity size of macrocycle **7** afforded from the crystal structure. Using Mercury software, a centroid at the C26–C26^{i} bond on the phenanthrene moiety was calculated, which along with the centroid calculated from the 24 porphyrin atoms and the two centroids of the phenyl spacers allowed the estimation of the cavity size in **7**. Carbon atoms are shown in grey, nitrogen in blue and oxygen in red. Hydrogen atoms are omitted for clarity purposes. Ellipsoids are drawn at 50% probability levels. Symmetry code: i = 1/2-x, y, z.

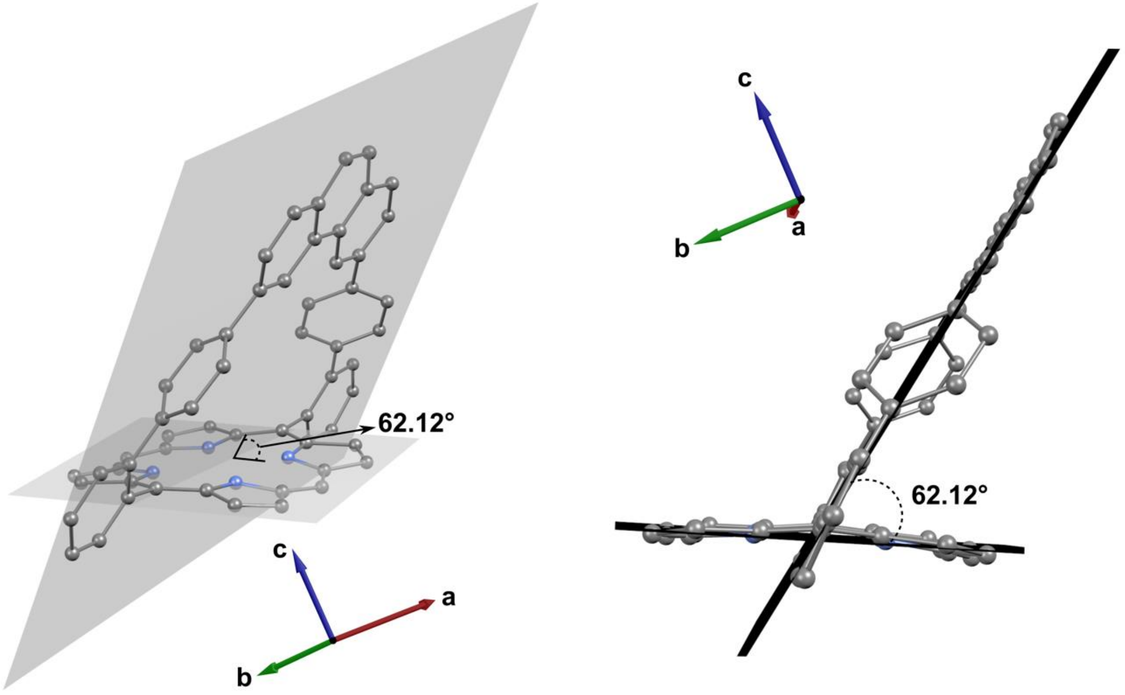

Figure S101 – Macrocycle **7** crystal structure highlighting the porphyrin ring and aromatic molecular loop mean planes with the 62.12º angle between them.

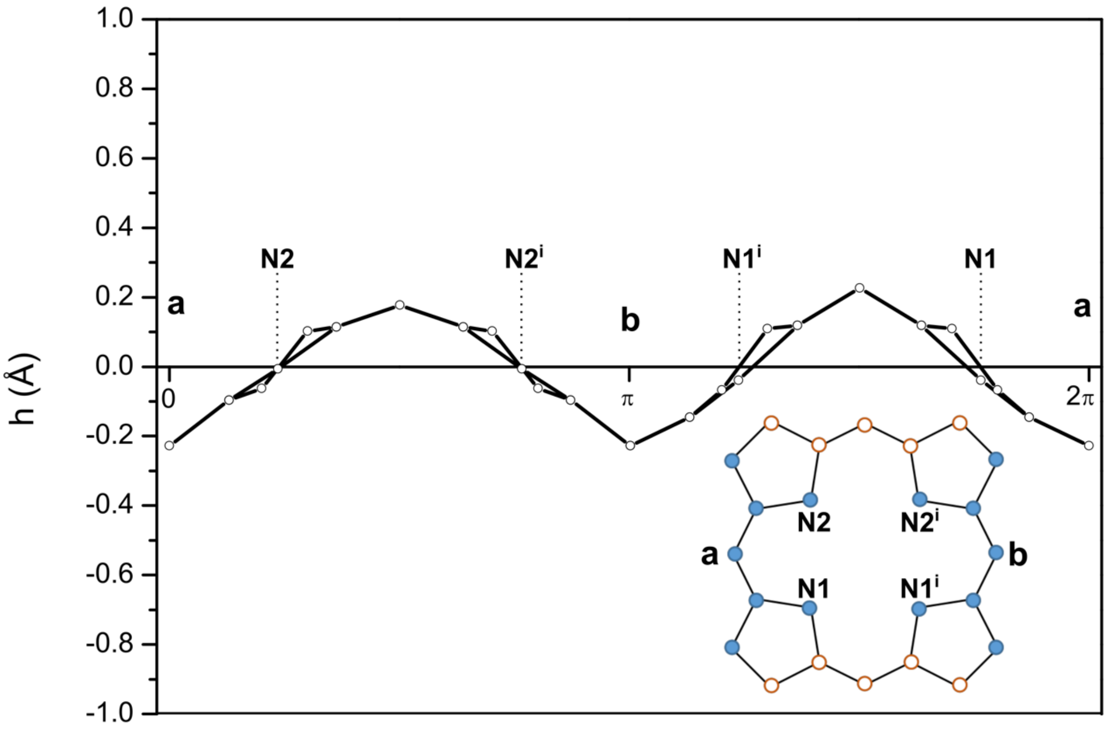

Figure S102 – Cylindrical projection of the porphyrin moiety in **7** with the *z*-coordinate displacement relative to the porphyrin mean plane, on the vertical axis (*h*, in Å), and the azimuthal angle on the horizontal axis. The inset scheme shows the porphyrin-core

conformation with open-orange circles and closed-blue circles representing atoms lying above and below the mean plane, respectively. Although the cylindrical projection of the porphyrin group in **7** reveals a distortion pattern of a ruffled conformation, the very low deviation of the *z*-coordinate displacements for each atom (*h*, in Å) from the porphyrin ring mean plane (confirmed by the low root-mean-square out-of-plane value of 0.124 Å) informs that the porphyrin core is flat in **7**.[S18]

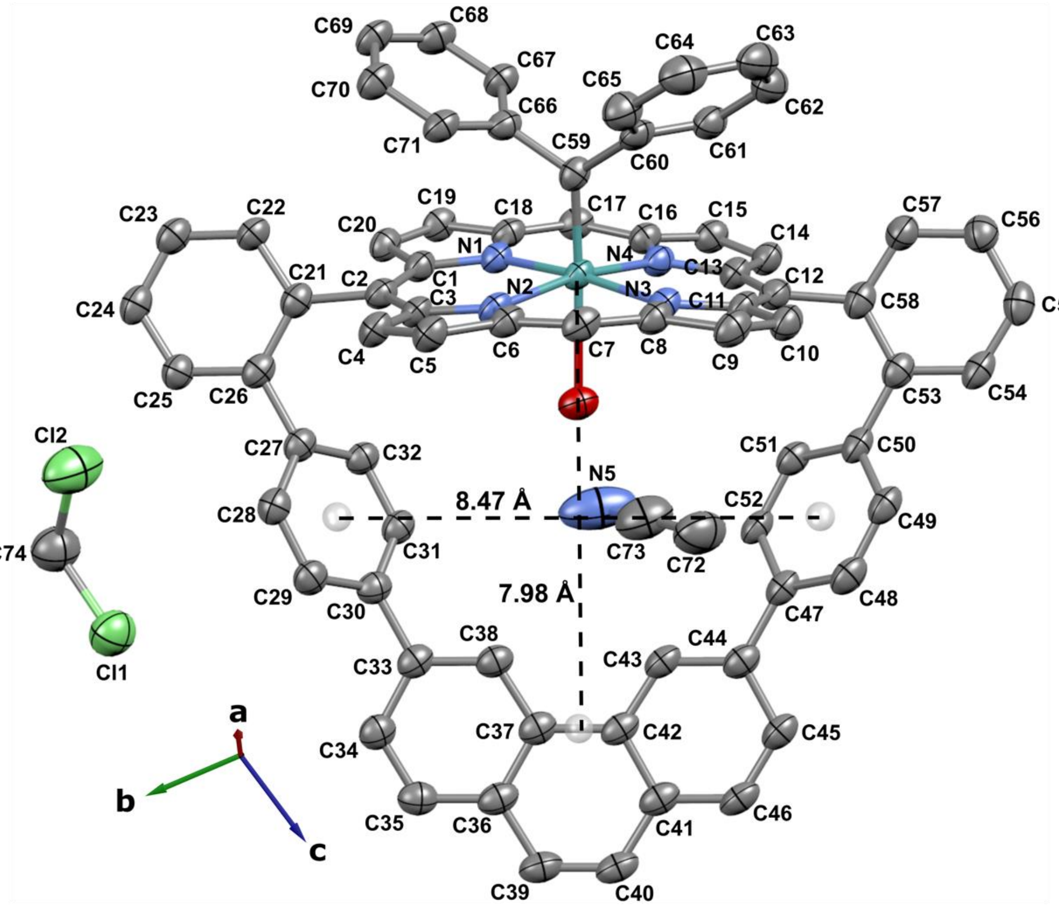

Figure S103 – Estimated cavity size of macrocycle **11** afforded from the crystal structure. Using Mercury software, a centroid at the C37–C42 bond on the phenanthrene moiety was calculated, which along with the centroid calculated from the 24 porphyrin atoms and the two centroids of the phenyl spacers allowed the estimation of the cavity size in **11**. Carbon atoms are shown in grey, nitrogen in blue, oxygen in red, ruthenium in turquoise, chlorine in green. Hydrogen atoms are omitted for clarity purposes. Ellipsoids are drawn at 50% probability levels.

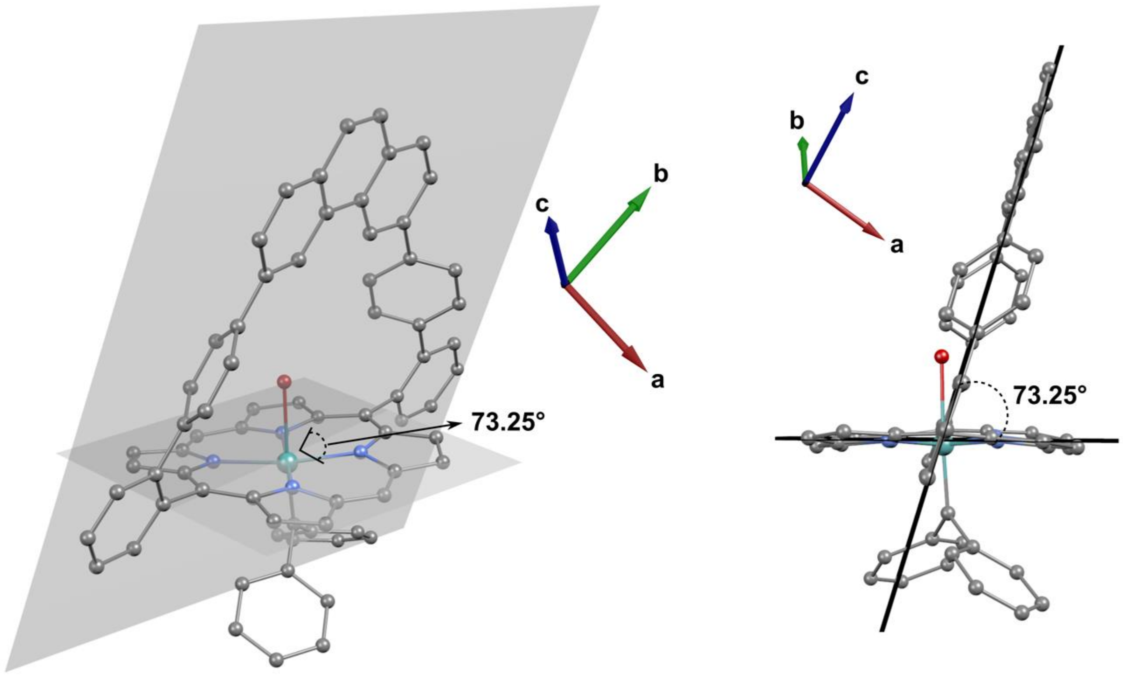

Figure S104 – Macrocycle **11** crystal structure highlighting the porphyrin ring and aromatic molecular loop mean planes with the 73.25º angle between them.

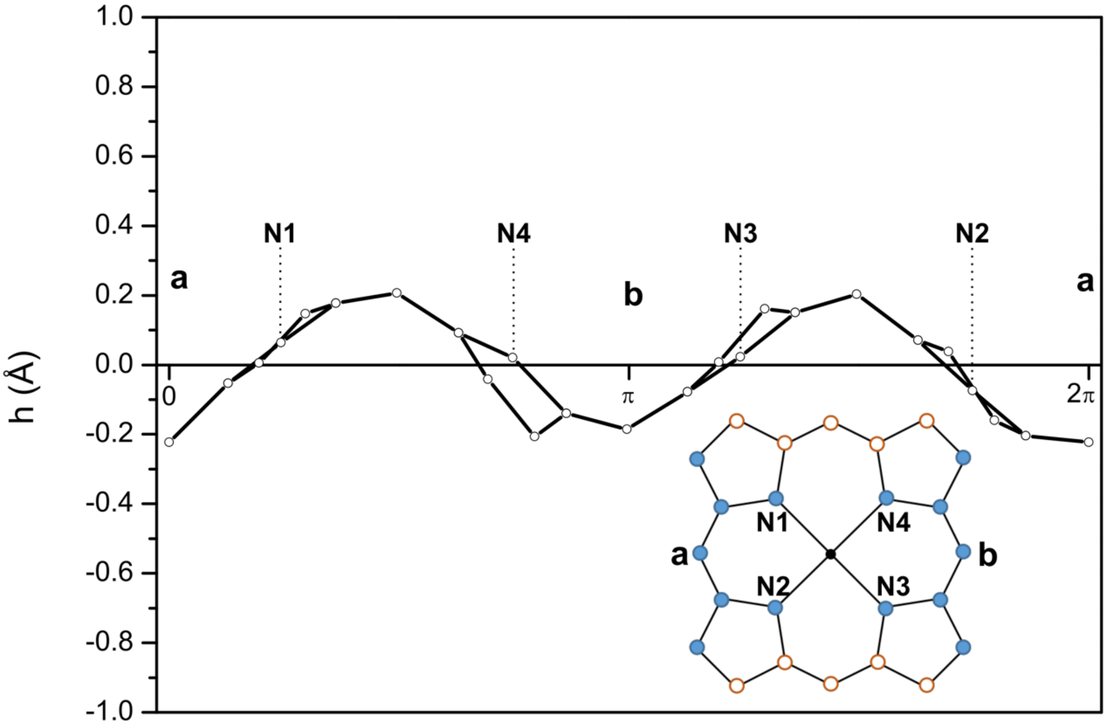

Figure S105 – Cylindrical projection of the porphyrin moiety of **11** with the *z*-coordinate displacement relative to the porphyrin mean plane, on the vertical axis (*h*, in Å), and the azimuthal angle on the horizontal axis. The inset scheme shows the porphyrin-core conformation with open-orange circles and closed-blue circles representing atoms lying

above and below the mean plane, respectively. Although the cylindrical projection of the porphyrin group in **11** reveals a distortion pattern of a ruffled conformation, the very low deviation of the *z*-coordinate displacements for each atom (*h*, in Å) from the porphyrin ring mean plane (confirmed by the low root-mean-square out-of-plane value of 0.135 Å) informs that the porphyrin core is flat in **11**.[S18]

6. References

S1 – Findley, T. W.; Swern, D.; Scanlan, J. T. *J. Am. Chem. Soc.* **1945**, *67*, 412.

S2 – Hu, M.; Ni, C.; Li, L.; Han, Y.; Hu, J. *J. Am. Chem. Soc.* **2015**, *137*, 14496.

S3 – Gottlieb, H. E.; Kotlyar, V.; Nudelman, A. *J. Org. Chem.* **1997**, *62*, 7512.

S4 – Alcântara, A. F. P.; Fontana, L. A.; Rigolin, V. H.; Andrade, Y. F. S.; Ribeiro, M. A.; Barros, W. P.; Ornelas, C.; Megiatto, Jr., J. D. *Angew. Chem. Int. Ed.* **2018**, *57*, 8979.

S5 – Astruc, D. *Organometallic Chemistry and Catalysis*. Springer-Verlag, Berlin, 2007.

S6 – Portela-Cubillo, F.; Scott, J. S.; Walton, J. C. *J. Org. Chem.* **2008**, *73*, 5558.

S6 – Smith, M. K.; Northrop, B. H. *Chem. Mater.* **2014**, *26*, 3781.

S7 – Talele, H. R.; Chaudhary, A. R.; Patel, P. R.; Bedekar, A. V. *ARKIVOC* **2011**, *2011* (IX), 15.

S8 – Lindsey, J. S. Schreiman, I. C.; Hsu, H. C.; Kearney, P. C.; Marguerettaz, A. M. *J. Org. Chem.* **1987**, *52*, 827.

S9 – Guimaraes, B. G., Sanfelici, L., Neuenschwander, R. T., Rodrigues, F., Grizolli, W. C., Raulik, M. A., Piton, J. R., Meyer, B. C., Nascimento, A. S., Polikarpov, I. (2009). *J. Synchrotron Rad.* 16, 69.

S10 – Winn, M. D., Ballard, C. C., Cowtan, K. D., Dodson, E. J., Emsley, P., Evans, P. R., Keegan, R. M., Krissinel, E. B., Leslie, A. G. W., McCoy, A., McNicholas, S. J., Murshudov, G. N., Pannu, N. S., Potterton, E. A., Powell, H. R., Read, R. J., Vagin, A. & Wilson, K. S. (2011). *Acta Cryst. D* 67, 235.

S11 – Winter, G. (2010). *J. Appl. Cryst.* 43, 186.

S12 - Winter G, Waterman DG, Parkhurst JM, Brewster AS, Gildea RJ, Gerstel M, Fuentes-Montero L, Vollmar M, Michels-Clark T, Young ID, Sauter NK, Evans G. *Acta Crystallogr D Struct Biol* **74**, 85.

S13 – Evans, P. R. & Murshudov, G. N. (2013). *Acta Cryst. D* 69, 1204-1214.

S14 – Evans, P. (2006). *Acta Cryst. D* 62, 72.

S15 – Dolomanov, O.V., Bourhis, L.J., Gildea, R.J, Howard, J.A.K., Puschmann, H. (2009), *J. Appl. Cryst.* 42, 339.

S16 – Sheldrick, G.M. (2015). *Acta Cryst. A* 71, 3.

S17 – Sheldrick, G.M. (2015). *Acta Cryst. C* 71, 3.

S18 – Shelnutt, J. A.; Song, X.-Z.; Ma, J.-G.; Jia, S.-L.; Jentzen, W.; Med-forth, C. J.; *Chem. Soc. Rev.* **1998**, *27*, 31.
